# Down‐regulation of *MYH10* driven by chromosome 17p13.1 deletion promotes hepatocellular carcinoma metastasis through activation of the EGFR pathway

**DOI:** 10.1111/jcmm.17036

**Published:** 2021-11-04

**Authors:** Qian Jin, Min Cheng, Xia Xia, Yuqing Han, Jing Zhang, Pengbo Cao, Gangqiao Zhou

**Affiliations:** ^1^ State Key Laboratory of Proteomics National Center for Protein Sciences at Beijing Beijing Institute of Radiation Medicine Beijing China; ^2^ Collaborative Innovation Center for Personalized Cancer Medicine Center for Global Health School of Public Health Nanjing Medical University Nanjing City China; ^3^ State Key Laboratory of Proteomics National Center for Protein Sciences at Beijing Beijing Institute of Lifeomics Beijing China; ^4^ College of Life Sciences Hebei University Baoding City China

**Keywords:** 17p13.1 deletion, EGFR pathway, hepatocellular carcinoma, MYH10

## Abstract

Somatic copy number alterations (CNAs) are a genomic hallmark of cancers. Among them, the chromosome 17p13.1 deletions are recurrent in hepatocellular carcinoma (HCC). Here, utilizing an integrative omics analysis, we screened out a novel tumour suppressor gene within 17p13.1, *myosin heavy chain 10* (*MYH10*). We observed frequent deletions (~38%) and significant down‐regulation of *MYH10* in primary HCC tissues. Deletion or decreased expression of *MYH10* was a potential indicator of poor outcomes in HCC patients. Knockdown of *MYH10* significantly promotes HCC cell migration and invasion in vitro, and overexpression of MYH10 exhibits opposite effects. Further, inhibition of *MYH10* markedly potentiates HCC metastasis in vivo. We preliminarily elucidated the mechanism by which loss of *MYH10* promotes HCC metastasis by facilitating EGFR pathway activation. In conclusion, our study suggests that MYH10, a candidate target gene for 17p13 deletion, acts as a tumour suppressor and may serve as a potential prognostic indicator for HCC patients.

## 1 INTRODUCTION

The global incidence of hepatocellular carcinoma (HCC), the most common primary liver malignancy, will reach a million by 2025.^1^, ^2^ Owing to their high recurrence and metastasis rates, the 5‐year survival rate of HCC patients remains limited. Elucidating the molecular mechanisms of HCC initiation and progression may lead to improved diagnostic and therapeutic strategies.^3^

Somatic chromosome copy number alterations (CNAs), which generally consist of tens or hundreds of genes, are common features of human cancers.^4^, ^5^ Accumulating evidence has indicated that several CNAs functioned as the drivers of human cancers, which occur at the early stages of tumorigenesis.^6^ For instance, chromosome 3p deletions are detected in more than 90% of lung cancers,^7^ and chromosome 8q is frequently amplified in HCCs.^8^ Among them, chromosome 17p13.1 deletions are extremely common in both hematopoietic malignancies and solid cancers, including HCC.^3^, ^9^ It has been proposed that tumour suppressor genes are enriched in cancer‐associated chromosome deletion regions while oncogenes are enriched in chromosome amplicons.^10^ However, given that a large number of genes are involved in each CNA region, it has been challenging to functionally identify the potential tumour suppressor genes or oncogenes for better mechanistic insights of these CNAs. A recent study had assumed that the known function of 17p deletion is for *TP53* loss of heterozygosity (LOH).^11^ It was demonstrated that 17p deletion could promote cancer development through both *TP53*‐dependent and ‐independent mechanisms. However, the additional tumour suppressor genes in this region remain unexplored. There are about tens of genes on chromosome 17p13.1 and a large amount of effort has been made to identify new tumour suppressor genes in this region.^12^ Recently, it has been reported that *EIF5A* and *ALOX15B*, both located within 17p13.1, prevent tumorigenesis by promoting apoptosis of pre‐malignant B cells.^11^, ^12^ These studies imply that potential tumour suppressor genes involved in 17p13.1 have been largely underestimated and emphasize the functional studies for further identification of novel candidates within this region. Currently, the mechanism of action of 17p13.1 deletion in HCC remains unknown, with most studies focusing on multiple myeloma.

MYH10 is a member of non‐muscle myosin II, and influences the cell migration by regulating the localization of the centriole and the Golgi apparatus.^13^, ^14^ It has been found that the dysregulation of MYH10 participated in the invasion of breast cancer cells.^15^ Loss‐of‐function (LOF) mutations in *MYH10* have been proved to promote cell migration and associate with metastasis of nasopharyngeal carcinoma and glioma.^16^, ^17^ However, it lacks evidence for MYH10’s tumour‐suppressive role in HCC progression.

In this study, utilizing an integrative omics analysis in HCC, we screened out *MYH10* as a novel tumour suppressor within the genomic deletion region at chromosome 17p13.1. Consistent with its recurrent copy number loss and down‐regulation in HCC tissues, MYH10 plays a tumour‐suppressive role in HCC by reducing HCC metastasis both in vitro and in vivo. Furthermore, MYH10 exerts its functions through attenuating the EGFR pathway.

## 2 MATERIALS AND METHODS

### 2.1 Genomic CNA, gene expression and clinical relevance analyses based on publicly available data from HCC samples

The genomic copy number data and mRNA expression data of the Cancer Genome Atlas (TCGA)‐liver hepatocellular carcinoma (LIHC) cohort (https://cancergenome.nih.gov/) were applied for genomic characterization of 17p13.1 and screening for novel tumour suppressor gene(s). The relative copy number (log_2_ transformed) >0.3 was defined as genomic amplification, while the relative copy number (log_2_ transformed) <−0.3 was considered as deletion. In addition to the TCGA dataset, the other 11 gene expression profile datasets were obtained from the HCCDB database (http://lifeome.net/database/hccdb), including the International Cancer Genome Consortium Liver Cancer‐RIKEN Japan (ICGC‐LIRI‐JP) cohort, GSE22058, GSE36376, GSE63898, GSE76427, GSE10143, GSE25097, GSE14520, GSE46444, GSE54236 and GSE64041. The difference in *MYH10* expression levels between HCCs and ANTLs was assessed by the Wilcoxon rank‐sum test. *p* < 0.05 and log_2_(fold‐change) <−0.2 was considered to be statistically significant. Survival information in TCGA‐LIHC was used to analyse the clinical relevance of *MYH10* loss or down‐regulation. The PRISMA flow was shown in Figure S1.

### 2.2 HCC patients recruitment and tissue samples collection

A total of 154 pairs of human HCC tissues and ANTLs were collected for this study. The First Affiliated Hospital of Jinling Hospital (Nanjing City, China), Jindu Hospital (Nanjing City, China) and Guangxi Cancer Hospital (Nanning City, China) helped recruit the cohort between 2007 and 2016 (Table S1) as previously described.^18^ The HCC tissues were used to genotype the genomic copy number of *MYH10* by CNVplex assays, and all pairs of HCC tissues and ANTLs were applied for examining the protein expression levels of MYH10 by immunohistochemistry (IHC) assays. These newly diagnosed and untreated (chemotherapy or radiotherapy) HCC samples were pathologically confirmed and tumour free. Consents for sample collection were obtained from the HCC patients or their guardians. This study was performed under the supervision of Medical Ethical Committee of Beijing Institute of Radiation Medicine (Beijing, China).

### 2.3 DNA extraction and CNA analyses by quantitative PCR (qPCR) assays

First, we extracted total DNA from the liver cell lines using Trizol Reagent (15596026, Invitrogen, USA). To determine the DNA copy numbers of *MYH10*, a pair of primers were designed on the basis of the intron sequences of *MYH10*. The average genomic content of three genes, including *LTBP1* (at 2p22.2 locus), *SATB1* (3p24.3) and *ANO3* (11p14.3), which were confirmed to having no copy number alterations in our HCC cohorts (data not shown), was used as the internal reference. All primers were listed in Table S6. The qPCR assays were performed using SYBR FAST qPCR kit (KK4607, Kapa Biosystems, USA) on IQ5 real‐time PCR system (BioRad, USA).

### 2.4 Validation of CNAs by CNVplex assays

The genomic copy number of *MYH10* was profiled by CNVplex^®^ assays (GENESKY, China). The CNVplex assays were performed as previously described.^18^ Briefly, each sample was subjected to capillary electrophoresis after the ligation reaction and PCR amplification. The relative copy number of *MYH10* was normalized to the average copy number of four reference gene loci, including *POLR2A*, *POP1*, *RPP14* and *TBX15*. All probes were listed in Table S6. Clean data were obtained after GeneMapper 4.1 (ABI, USA) analysis.

### 2.5 RNA extraction and real‐time quantitative PCR (RT‐qPCR) assays

Total RNAs were extracted from the cultured cells using RNApure tissue&cell kit (CW0584S, CWBIO, China). The cDNAs were synthesized using PrimeScript RT reagent kit (RR037A, Takara, Japan). The RT‐qPCR assays were performed using SYBR FAST qPCR kit (KK4607, Kapa Biosystems, USA) on IQ5 real‐time PCR system (BioRad, USA). The relative mRNA expression levels of *SPRY2*, *DUSP6*, *DUSP4*, *SPRED1* and *MYH10* were calculated with the 2^−ΔΔCt^ method and normalized to *GAPDH*, respectively. The primer sequences for RT‐qPCR are shown in the Table S6.

### 2.6 Cell lines and plasmids

The human normal liver cell line L‐02 and HCC cell line HepG2 were purchased from the China Center for Type Culture Collection (CCTCC, Wuhan City, China); three HCC cell lines (SMMC7721, HCCLM3 and MHCC97H) were kindly provided by Prof. Shuhan Sun (Navy Military Medical University, Shanghai, China); the HCC cell line Huh7 was a gift from Prof. Chunyan Tian (Beijing Institute of Lifeomics, Beijing, China). All these cells were cultured in Dulbecco's modified Eagle's medium (DMEM) (containing 10% foetal bovine serum, 50 U/mL penicillin and 0.1 mg/mL streptomycin) in a humidified atmosphere (37°C, 5% CO_2_). To stably knock down the expression of *MYH10*, the sequences of short hairpin RNAs (shRNAs) targeting *MYH10* were cloned into the GV248 vector. Sequences of shRNAs are listed in the Table S6. For MYH10 overexpression, the intact sequences of *MYH10* were synthesized by Biomed Co. (Beijing, China) and cloned into the pLV‐Neo‐Flag vector (Clontech, USA). Then, the pLV‐Neo‐Flag and pLV‐Neo‐Flag‐MYH10 vectors were transfected into HCCLM3 and MHCC97H cells using Lipofectamine 2000 (Invitrogen, USA).

### 2.7 Cell proliferation and colony formation assays

The proliferation rate of HCC cells was detected using the Cell Counting Kit‐8 (CCK‐8) (CK04, Dojindo, Japan) according to the manufacturer's instructions.

For colony formation assays, 1000 cells were seeded in 6‐well plates per well. Two—three weeks later, the colonies were stained with 0.5% crystal violet for 25 min at room temperature and then scanned and counted. An average number of colonies were obtained from three replicates. Three replicates were set up for each experiment and three independent experiments were conducted.

### 2.8 Cell migration and invasion assays

A total of 5 × 10^4^ cells (200 μL) were planted on the top chamber of each insert (353097, BD Biosciences, USA) or matrigel invasion chamber (354480, BD Biosciences, USA). Six hundred microlitre DMEM (containing 20% FBS) was injected into the lower chambers. After incubation at 37°C for 24–48 h, cells that migrated to the other side of the insert were stained with 0.5% crystal violet. The inserts were subsequently rinsed clean and counted by the IX71 inverted microscope (Olympus, Japan). An average number of cells were obtained from three random fields of view of the chamber. All experiments were repeated three times.

### 2.9 Nude mice assays

The experiments were carried out in compliance with the requirements of the Institutional Animal Care and Use Committee of the National Center for Protein Sciences at Beijing (Beijing, China). Five‐ to six‐week‐old male nude BALB/c mice were purchased from Vital River Laboratories (Beijing, China). HepG2 cells transfected with scramble control or shRNA (sh*MYH10*‐1 and sh*MYH10*‐2 were pooled as 1:1) were diluted to 2 × 10^7^ per mL. For subcutaneous tumour formation assay, 2 × 10^6^ cells HepG2 cells were injected subcutaneously into each side of the mice back (*n* = 6). The length (L) and width (W) of the lump was measured every 4 days with callipers. The equation V = (1/2) × L × W^2^ was used to calculate the tumour volume. After 68 days, the mice were sacrificed, the tumours were stripped, and tumour weights were measured.

For in vivo metastasis model, SMMC7721 cells stably expressing firefly luciferase (pCMV‐luciferase) were transfected with scramble control or sh*MYH10* (sh*MYH10*‐1 and sh*MYH10*‐2 were pooled as 1:1). These cells (2 × 10^6^) were injected into the lateral tail vein of BALB/c nude mice (*n* = 6). The first test was performed after 24 h with the IVIS@ Lumina II system (Caliper Life Sciences, USA). The luciferase substrate (Gold Biotech, USA) was injected intraperitoneally into the mice 10 min prior to the assay. Thereafter, the test was performed every 7 days. After 4 weeks, all mice were sacrificed, and their lung tissues were applied for haematoxylin and eosin (H&E) staining. The numbers of metastatic tumours were evaluated based on H&E staining.

### 2.10 mRNA expression profiling upon *MYH10* knockdown

Total RNAs were extracted from HepG2 cells stably transfected with scramble control (shCtrl) or sh*MYH10* (sh*MYH10*‐1 and sh*MYH10*‐2 were pooled as 1:1) using RNApure tissue&cell kit (CW0584S, CWBIO, China). Three replicates were available for both the shCtrl and sh*MYH10* groups. The Affymetrix GeneChip Human Gene U133 Array was used for gene expression profiling. The profiling was performed by CapitalBio Corporation (Beijing, China) following the protocol of Affymetrix (USA). Row data were processed by using Robust Multi‐array Average (RMA).^19^ The difference of gene expression levels between sh*MYH10* and shCtrl was assessed by DESeq2.^20^ Adjusted *p* (false discovery rate [FDR]) <0.05 was considered to be statistically significant.

### 2.11 Gene set enrichment analyses

Gene set enrichment analysis (GSEA)^21^ was performed based on the paired groups (sh*MYH10* vs. shCtrl) with the genes ranked according to their log_2_ fold‐changes (sh*MYH10* vs. shCtrl). The significant gene sets were identified by the weighted Kolmogorov–Smirnov test from the MsigDB (v6).^21^ False discovery rate (FDR) was calculated by the Benjamini–Hochberg method. The FDR <0.05 was considered to be statistically significant. Cytoscape and EnrichmentMap^22^ were applied for the visualization and cluster of GSEA results, respectively.

### 2.12 Immunoblotting assays

Cells were lysed in RIPA buffer plus EDTA‐free protease inhibitor cocktail (04693132001, Roche, Germany) and phosphatase inhibitor cocktail (CW2383, CWBIO, China) for 30 min in ice. The cell lysates were centrifuged and boiled to make total cellular protein. The protein samples were subjected to SDS‐PAGE and transblotted onto the BioTrace™ NT Nitrocellulose Transfer Membrane (P/N66485, PALL, USA). Place the membrane in the target primary antibody dilution and incubate overnight at 4°C. After washing the membrane with Tris‐buffered saline/Tween (TBST), incubate the secondary antibody for 1 h at room temperature. The immunobands were detected with Tanon™ Femto‐sig ECL Chemiluminescent Substrate (180‐506, Tanon, China) and gel image analysis system (1600, Tanon, China). The following primary antibodies were used in this study: mouse monoclonal anti‐GAPDH (1:1000; CW0100 M, CWBIO, China), mouse monoclonal anti‐Flag (1:1000; NBP1‐06712, MBL, Japan), rabbit monoclonal anti‐MYH10 (1:1000; 8824, CST, USA), rabbit monoclonal anti‐EGFR (1:1000; 4267, CST, USA), rabbit monoclonal anti‐phosphorylated‐EGFR (p‐EGFR, 1:1000; 3777, CST, USA), mouse monoclonal anti‐ERK1/2 (1:500; 4696, CST, USA), rabbit monoclonal anti‐p‐ERK1/2 (1:500; 4370, CST, USA), rabbit monoclonal anti‐AKT (1:500; 9272, CST, USA) and rabbit monoclonal anti‐p‐AKT (1:500; 4060, CST, USA).

### 2.13 Immunohistochemistry (IHC) assays

The protein expression levels of MYH10 were examined by IHC assays in tumour tissues and adjacent non‐tumour tissues from HCC patients. The following operations were performed on the slides, including de‐paraffinization, dehydration and washing. After incubation with 3% H_2_O_2_ for 10 min, the slides were placed in a citrate buffer (pH = 6.0) at high pressure for 2 min. The slides were then incubated overnight at 4°C in primary antibody dilution (anti‐MYH10; 1:200; ab230823, Abcam, USA). After washing, the slides were treated by the GTVision™ TV Detection System (GK500710, DAKO, Denmark). Then, all the slides were stained with 3, 3‐diaminobenzidine tetra‐hydrochloride (DAB) and haematoxylin. Image information of the slides was captured through the Olympus BX51 microscopic/Digital Camera System (Olympus, Japan). The overall score was obtained by scoring the proportion of positive staining tumour cells (0, none; 1, <1/100; 2, 1/100 to <1/10; 3, 1/10 to <1/3; 4, 1/3 to <2/3; and 5, >2/3) and the intensity of staining (0, none; 1, weak; 2, intermediate; and 3, strong). Statistical differences between HCC and ANTL groups were analysed using a Wilcoxon rank‐sum test.

### 2.14 Statistical analyses

All quantification data are indicated as mean ± standard deviation (SD) from three independent experiments. Statistical differences of multiple group comparisons were analysed using two‐sided Student's *t* test or ANOVA. Disease‐free survival (DFS) ranges from the day of resection to the day of the first HCC recurrence, death or last follow‐up. Overall survival (OS) ranges from the date of the surgery to death or the last follow‐up. Survival analysis was performed by the Kaplan–Meier method and log‐rank test. In all statistical tests, *p* < 0.05 was considered to be significant unless stated otherwise. Statistical analyses were performed using R (version 3.1.2) software.

## 3 RESULTS

### 3.1 Integrative omics analysis prioritizes *MYH10* as a candidate functional target of chromosome 17p13.1 deletion

**FIGURE 1:**
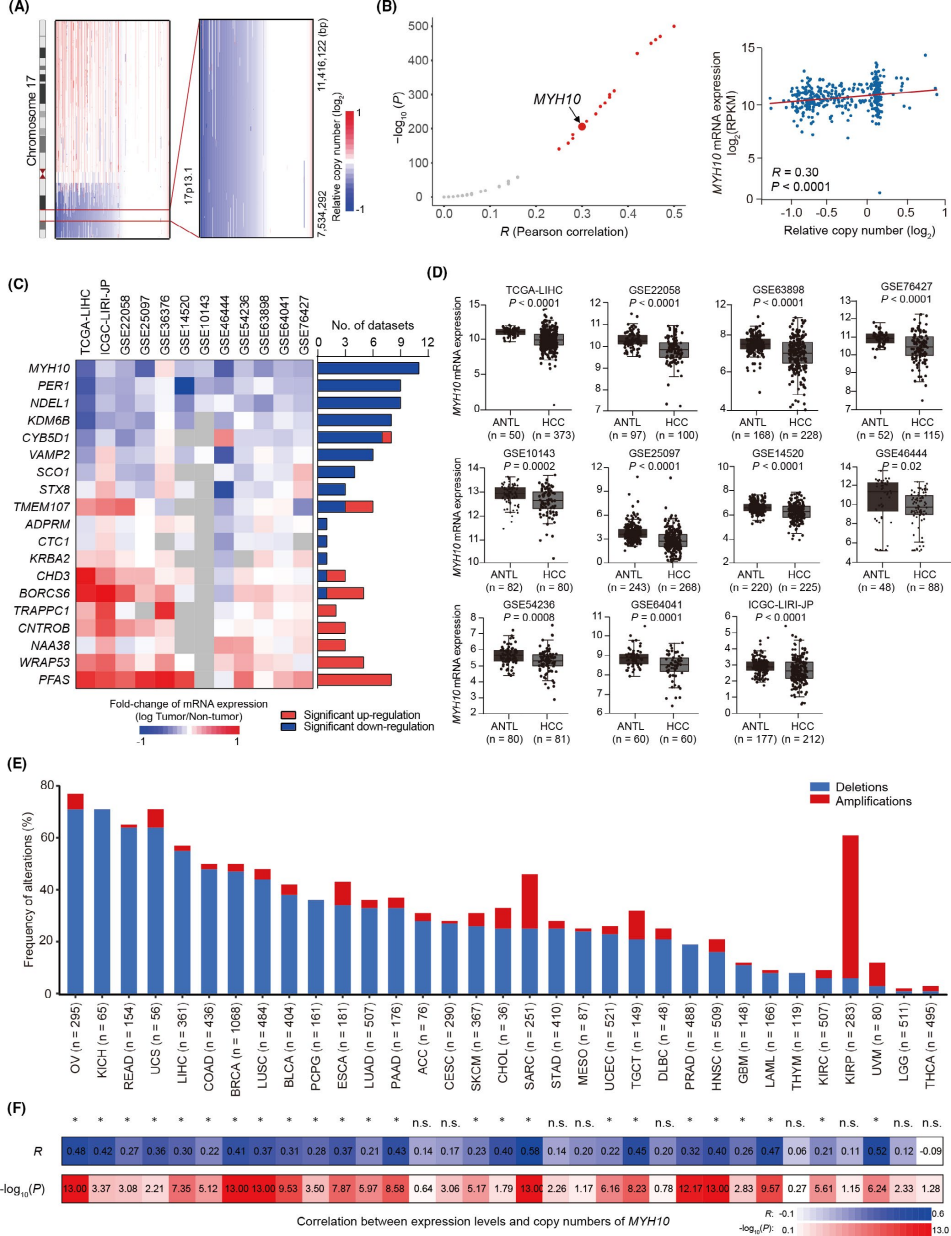
Genomic loss at chromosome 17p13.1 correlates with down‐regulation of *MYH10*. (A) The characteristics of genomic loss at chromosome 17p13.1. The distribution of depletions (in blue) and amplifications (in red) of chromosome 17p13.1 from individual HCC on the basis of the copy number alteration (CNA) dataset of the Cancer Genome Atlas (TCGA)‐liver hepatocellular carcinoma (LIHC) cohort (Materials and Methods**)**. The 17p13.1 cytoband is emphasized by a red line. (B) Left, the rank of Pearson correlations between the mRNA levels of each protein‐coding gene (*n* = 47) within the focally deleted region at 17p13.1 and the copy numbers of 17p13.1. Nineteen (in red) of 47 genes were identified as candidates with significant correlations (*R* > 0.2, *p* < 0.05). Right, the correlations between the mRNA levels of *MYH10* and the copy numbers of 17p13.1. RPKM, reads per kilobase per million mapped reads. (C) Dysregulation of the 19 cis‐regulated genes by 17p13.1 deletions in multiple gene expression datasets of HCC cohorts from the HCCDB database (http://lifeome.net/database/hccdb). The genes with *p* < 0.05 (Wilcoxon rank‐sum test) and log_2_(fold‐change) <−0.2 were considered to be significantly down‐regulated in HCC tissues compared to adjacent non‐tumour liver tissues (ANTLs). (D) Down‐regulation of *MYH10* in 11 independent cohorts of HCC patients (including TCGA‐LIHC, ICGC‐LIRI‐JP, GSE22058, GSE63898, GSE76427, GSE10143, GSE25097, GSE14520, GSE46444, GSE54236 and GSE64041). (E) Genomic alteration frequencies of *MYH10* in 33 types of cancer from the TCGA database. (F) The correlations between the mRNA levels and the copy numbers of *MYH10* in 33 types of cancer from the TCGA database, significant correlation with both *R* > 0.2 and *p* < 0.05; n.s., not significant. The abbreviations of cancer names are described on the TCGA (https://cancergenome.nih.gov/) database

Previous studies have found that losses of chromosome 17p13.1 were common in HCCs.^23^, ^24^ Tens of genes were within 17p13.1, and a large number of efforts have been made to illustrate the novel tumour suppressor gene(s) in this region.^12^ In this study, we were aimed to identify the functional gene(s) within this region through an integrative omics analysis combined with functional validation. First, we profiled the genomic copy number status at 17p13.1 by analysing the genomic data of 373 HCC patients in the TCGA‐LIHC cohort. The results showed that approximately 55% of HCC tumours carry the 17p13.1 deletions (Figure 1A). Then, we examined the cis‐regulation of 17p13.1 deletion on the expression levels of the 47 genes within 17p13.1 using the TCGA‐LIHC dataset. We observed that the expression levels of 19 (40.4%, 19/47) genes are significantly correlated with the copy numbers of 17p13.1 in HCC tumours (*R* > 0.2, *p* < 0.05; Figure 1B and Table S2). Subsequently, we investigated the dysregulation of these 19 candidates in a large collection of mRNA expression profile datasets from multiple cohorts documented in the HCCDB database (including 12 datasets: TCGA‐LIHC, ICGC‐LIRI‐JP, GSE22058, GSE63898, GSE76427, GSE10143, GSE25097, GSE14520, GSE46444, GSE54236, GSE36376 and GSE64041). Notably, it was shown that *MYH10* is consistently down‐regulated (*p* < 0.05, log_2_[fold‐change] <−0.2) in HCC tissues compared to adjacent non‐tumour liver tissues (ANTLs) in 11 out of the 12 datasets (Figure 1C and D, and Figure S1 and Table S3), increasing its candidacy as the functional target within this deleted region. Further, we analysed the prevalence of *MYH10* deletion in 33 types of cancer from TCGA and found that the frequency of *MYH10* deletion was greater than 30% in half of the cancer types (Figure 1E). Moreover, the expression levels of *MYH10* are significantly correlated with the copy numbers in these types of cancer (Figure 1F), suggesting *MYH10* deletion as a trans‐cancer genomic feature.

### 3.2 Genomic deletion or down‐regulation of *MYH10* predicts poor outcomes of HCC patients

**FIGURE 2:**
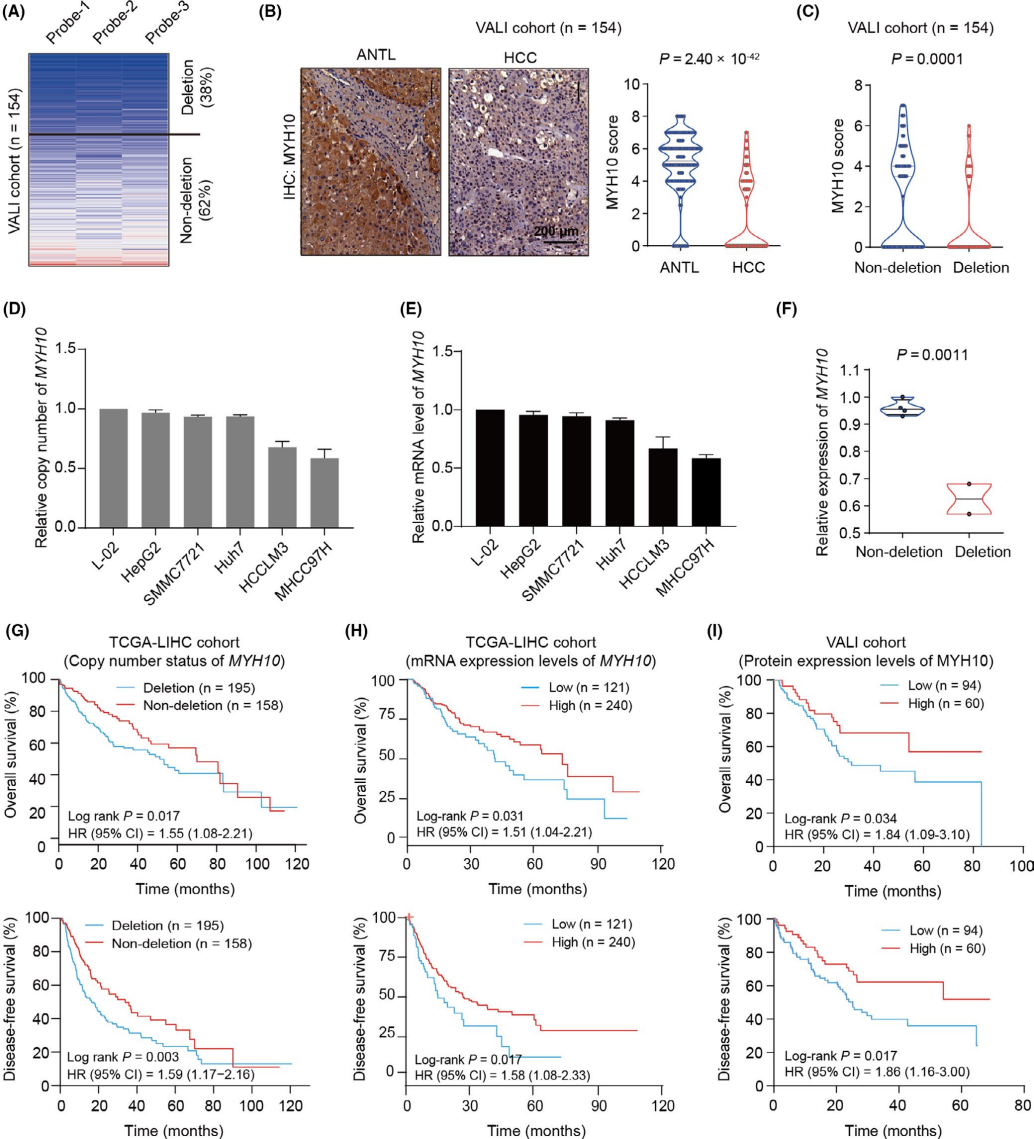
*MYH10* is down‐regulated in HCC tissues, suggesting poor outcomes of HCC patients. (A) *MYH10* is recurrently deleted in HCC tumours from the validation cohort (VALI, *n* = 154) determined by CNVplex assays. Three independent probes were applied to genotype the genomic copy number of *MYH10*. Four genes, including *POLR2A*, *POP1*, *RPP14* and *TBX15*, were applied as the internal references for normalization. (B) Protein levels of MYH10 were analysed by immunohistochemistry (IHC) assays in the VALI cohort. *P* value was calculated by Wilcoxon rank‐sum test. HCC, hepatocellular carcinoma tissue; ANTL, adjacent non‐tumour liver tissue. (C) The protein expression levels of MYH10 in HCC tumours with *MYH10* deletion or those with no deletion. *p* value was obtained by Wilcoxon rank‐sum test. (D) The relative copy numbers of *MYH10* were determined in multiple human hepatocyte cell lines, including one immortalized hepatocyte cell line (L‐02) and five HCC cell lines (HepG2, SMMC7721, Huh7, HCCLM3 and MHCC97H) by quantitative PCR (qPCR) assays. Three genes, including *SATB1*, *ANO3* and *LTBP1*, were used as internal reference for normalization. (E) The mRNA levels of *MYH10* were determined in multiple human hepatocyte cell lines utilizing real‐time quantitative PCR (RT‐qPCR) assays. (F) The mRNA expression levels of *MYH10* in MYH10 copy number deletion group and non‐deletion group cell lines. *p* Value was obtained by Student's *t*‐test. (G) Kaplan–Meier analysis for the overall survival (OS) rate (up) and disease‐free survival (DFS) rate (bottom) of HCC patients in the TCGA‐LIHC cohort. Patients with relative copy number (log_2_) of *MYH10* ≥−0.3 or <−0.3 in primary tumour tissues were designated as non‐deletion or deletion subgroup, respectively. *p* Value was obtained by log‐rank test. HR, hazard ratio; CI, confidence interval. (H) Kaplan–Meier analysis for OS rate (up) and DFS rate (bottom) of HCC patients in the TCGA‐LIHC cohort. The *MYH10* mRNA expression levels were classified by the higher two tertiles in the low expression group versus the lowest tertile representing the high expression group. *p* Value was calculated by log‐rank test. (I) Kaplan–Meier curves for OS rate (up) and DFS rate (bottom) of HCC patients in the VALI cohort. The patients with MYH10 IHC score >3 or ≤3 in primary tumour tissues were designated as high or low expression subgroup, respectively. *p* Value was obtained by log‐rank test

To validate these genomic findings, we first genotyped the copy number of *MYH10* in HCC tissues and ANTLs from a validation cohort consisting of 154 HCC patients (designated as VALI cohort; Table S1) by CNVplex assays.^25^ It was confirmed that ~38% of HCC patients are affected by *MYH10* deletion (Figure 2A). To further examine the dysregulation of MYH10 in HCC, we also detected the protein expression levels of MYH10 in the VALI cohort by IHC assays. MYH10 protein was predominantly located at cytoplasm and significantly decreased in HCC tissues compared to ANTLs (*p* = 2.40 × 10^−42^; Figure 2B), especially in those patients with vascular invasion (*p* = 0.031; Table S1). Again, HCC tumours with *MYH10* deletions also presented lower expression levels of MYH10 compared to those without *MYH10* deletions (*p* = 0.0001; Figure 2C). Consistent with the findings in clinical specimens, using RT‐qPCR assays, we also observed that *MYH10* is globally deleted or down‐regulated in HCC cell lines (including HepG2, SMMC7721, Huh7, HCCLM3 and MHCC97H), especially in those ones with higher metastatic capacity (HCCLM3 and MHCC97H), compared to the human hepatocyte cell line L‐02 (Figure 2D and E). Consistently, the MYH10 CNA‐mRNA cis‐correlation was observed in these types of cell lines (*p* = 0.0011; Figure 2F).

Further, we evaluated the potential of *MYH10* deficiency to predict prognosis in HCC patients. In TCGA‐LIHC cohort, the results showed that the genomic depletion at *MYH10* locus is significantly correlated with decreased overall survival (OS) rate (Log‐rank *p* = 0.017, HR = 1.55; Figure 2G) and disease‐free survival (DFS) rate (Log‐rank *p* = 0.003, HR = 1.59; Figure 2G). Additionally, Kaplan–Meier survival analyses of HCC patients from TCGA‐LIHC cohort based on the *MYH10* mRNA expression suggested worse OS (*p* = 0.031, HR = 1.51; Figure 2H) and DFS (*p* = 0.017, HR = 1.58; Figure 2H) in the patients with lower *MYH10* expression levels than those with high expression levels. Similarly, low expression of MYH10 protein levels suggested shorten OS (*p* = 0.034, HR = 1.84; Figure 2I) and DFS (*p* = 0.017, HR = 1.86; Figure 2I) in HCC patients from the VALI cohort. Taken together, these data enhanced to the persuasiveness of MYH10 as a marker for evaluating the prognosis status of HCC patients.

### 3.3 MYH10 reduces HCC cells migration and invasion in vitro

**FIGURE 3:**
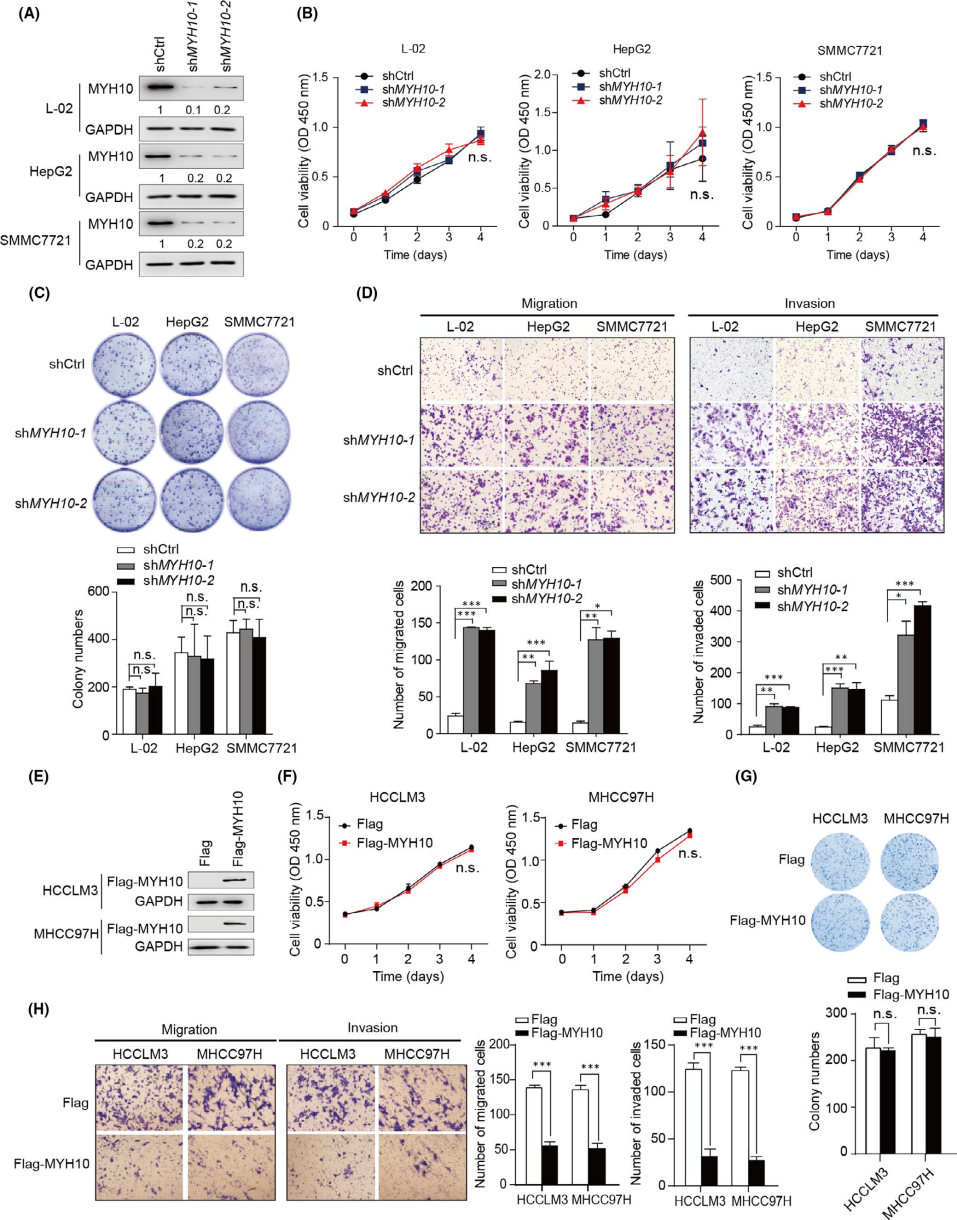
*MYH10* reduces HCC cell migration and invasion in vitro. (A) Stable knockdown of *MYH10* by two independent shRNAs. (B) CCK‐8 assays revealed that knockdown of *MYH10* has no pro‐growth effect on L‐02, HepG2 and SMMC7721 cells. (C) Knockdown of *MYH10* does not affect the plate colony formation abilities of L‐02, HepG2 and SMMC7721 cells. (D) Transwell assays revealed that knockdown of *MYH10* promotes migration and invasion of L‐02, HepG2 and SMMC7721 cells. (E) Construction of HCCLM3 and MHCC97H cells stably expressing exogenous MYH10. (F) CCK‐8 assays revealed that overexpression of MYH10 has no proliferative effect on HCCLM3 and MHCC97H cells. (G) Overexpression of MYH10 does not affect the plate colony formation abilities of HCCLM3 and MHCC97H cells. (H) Transwell assays revealed that overexpression of MYH10 decreases migration and invasion of HCCLM3 and MHCC97H cells. All quantification data are mean ± SD from three independent experiments. ^*^ *p* < 0.05, ^**^ *p* < 0.01 and ^***^ *p* < 0.001 (Student's *t* test). n.s., not significant

Next, we examined the tumorigenic effects of MYH10 on HCC cells. The mRNA expression levels of endogenous MYH10 were higher in L‐02, HepG2 and SMMC7721 cells than in the other HCC cell lines (Figure 2E). Therefore, we constructed L‐02, HepG2 and SMMC7721 cell lines with stable expression of shRNAs targeting *MYH10* (Figure 3A). Knockdown of *MYH10* has no significant effects on the growth of L‐02, HepG2 and SMMC7721 cells (Figure 3B, C). However, transwell assays revealed that knockdown of *MYH10* in L‐02, HepG2 and SMMC7721 cells markedly enhances the abilities of these cells migration and invasion (Figure 3D).

We further examined the effects of MYH10 overexpression on HCC cells. We constructed HCC cell lines HCCLM3 and MHCC97H that stably overexpressed MYH10. (Figure 3E). As expected, overexpression of MYH10 had no significant effect on either proliferation or plate colony formation of HCCLM3 and MHCC97H cells (Figure 3F and G). However, overexpression of MYH10 was able to reduce the migration and invasion abilities of these two types of cells (Figure 3H). Taken together, these results suggested that MYH10 has no effect on HCC cells proliferation, but plays suppressive roles in cells migration and invasion in vitro.

### 3.4 Down‐regulation of *MYH10* facilitates HCC metastasis in vivo

**FIGURE 4:**
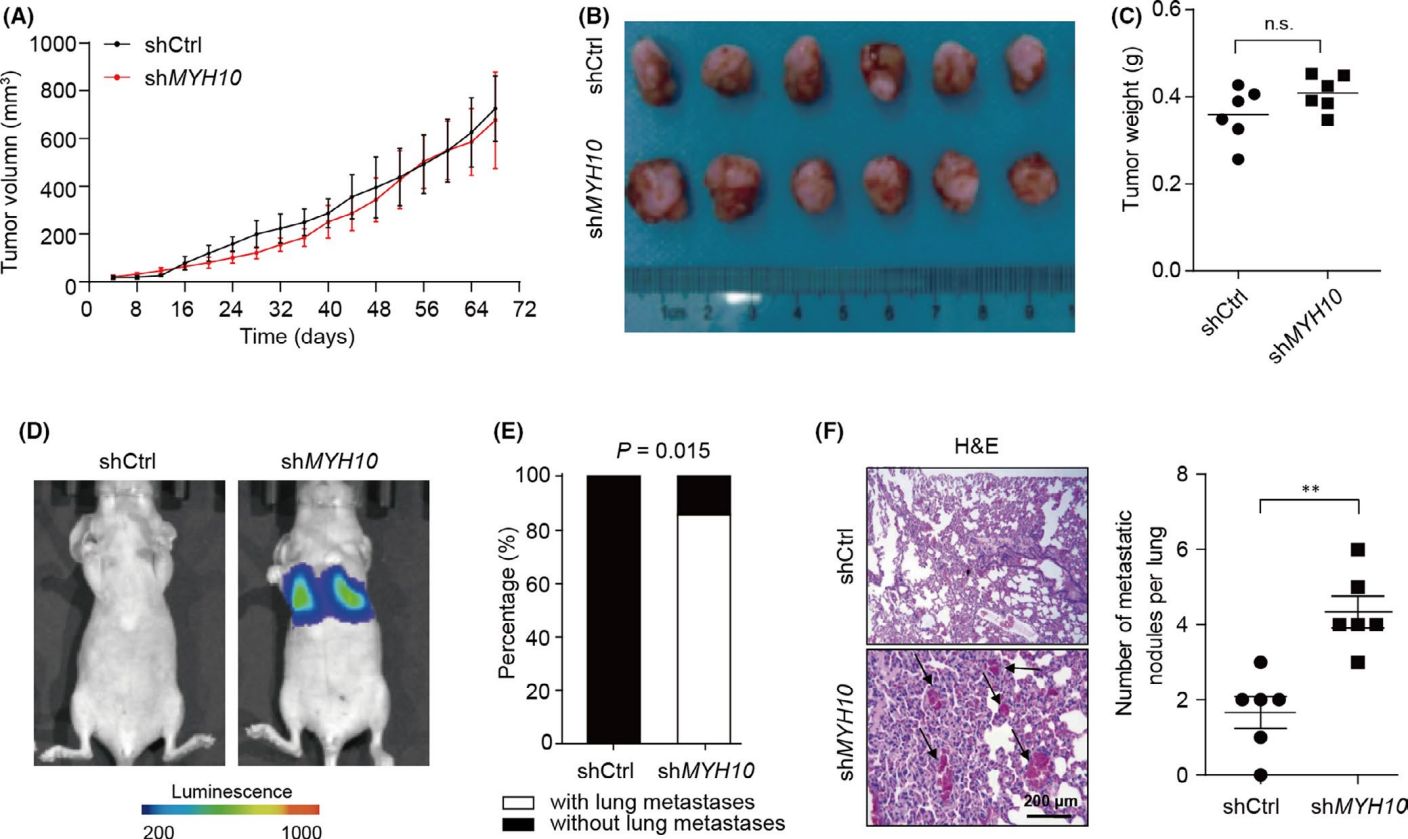
Depletion of *MYH10* promotes HCC metastasis in vivo. (A) The HepG2 cells (2 × 10^6^) stably expressed scramble control or sh*MYH10* were injected subcutaneously into each flank of the male BALB/c nude mice (*n* = 6). The volumes of tumours were measured every 4 days for 68 days. (B) Representative images of the subcutaneous tumour formation assay. All mice were sacrificed and tumours were taken on the 68th day. (C) Tumour volumes in the control and *MYH10*‐depleted groups (*n* = 6). Data are expressed as mean ± SD. (D) Representative images of luciferase signals in pulmonary metastatic luciferase foci were taken on the 28^th^ day. SMMC7721 cells that were engineered to stably express the firefly luciferase (pCMV‐luciferase) were infected with lentiviruses carrying scramble control or sh*MYH10* (sh*MYH10*‐1 and sh*MYH10*‐2 were pooled as 1:1). These cells (2 × 10^6^) were injected into the lateral tail vein of BALB/c nude mice (*n* = 6). The mice were photographed with the IVIS@ Lumina II system (Caliper Life Sciences, USA). (E) The incidence of lung metastasis was determined based on the bioluminescence imaging (BLI) results. The *p* value was calculated by Fisher's exact test. (F) Haematoxylin and eosin (H&E) staining of the lung tissues (left). Scale bars, 200 µm. Knockdown of *MYH10* increases the number of nodules metastasizing in the lungs of nude mice (right; *n* = 6). ^**^ *p* < 0.01 (Student's *t* test). n.s., not significant

We next focused on the tumorigenicity and metastasis function of MYH10 in HCC in vivo. *MYH10*‐depleted HepG2 cells and the control HepG2 cells were used for the subcutaneous xenograft model. The results showed that knockdown of *MYH10* does not affect the tumour growth and tumour weight (Figure 4A–C), indicating that MYH10 is not required for in vivo tumorigenicity. Next, we sought to examine whether depletion of *MYH10* promotes HCC metastasis. The bioluminescence imaging (BLI) results showed that knockdown of endogenous *MYH10* significantly promotes lung metastasis of SMMC7721 cells (Figure 4D): 83% of mice from the *MYH10*‐depleted group developed lung metastases, while no mice in the control group developed such metastases (*p* = 0.015; Figure 4E). Histological analyses confirmed that the mice injected with *MYH10*‐depleted cells had more metastatic nodules in the lungs than the mice from the control group (Figure 4F). Together, these results suggested that loss of *MYH10* facilitates HCC metastasis in vivo.

### 3.5 Depletion of *MYH10* enhances the EGFR pathway

**FIGURE 5:**
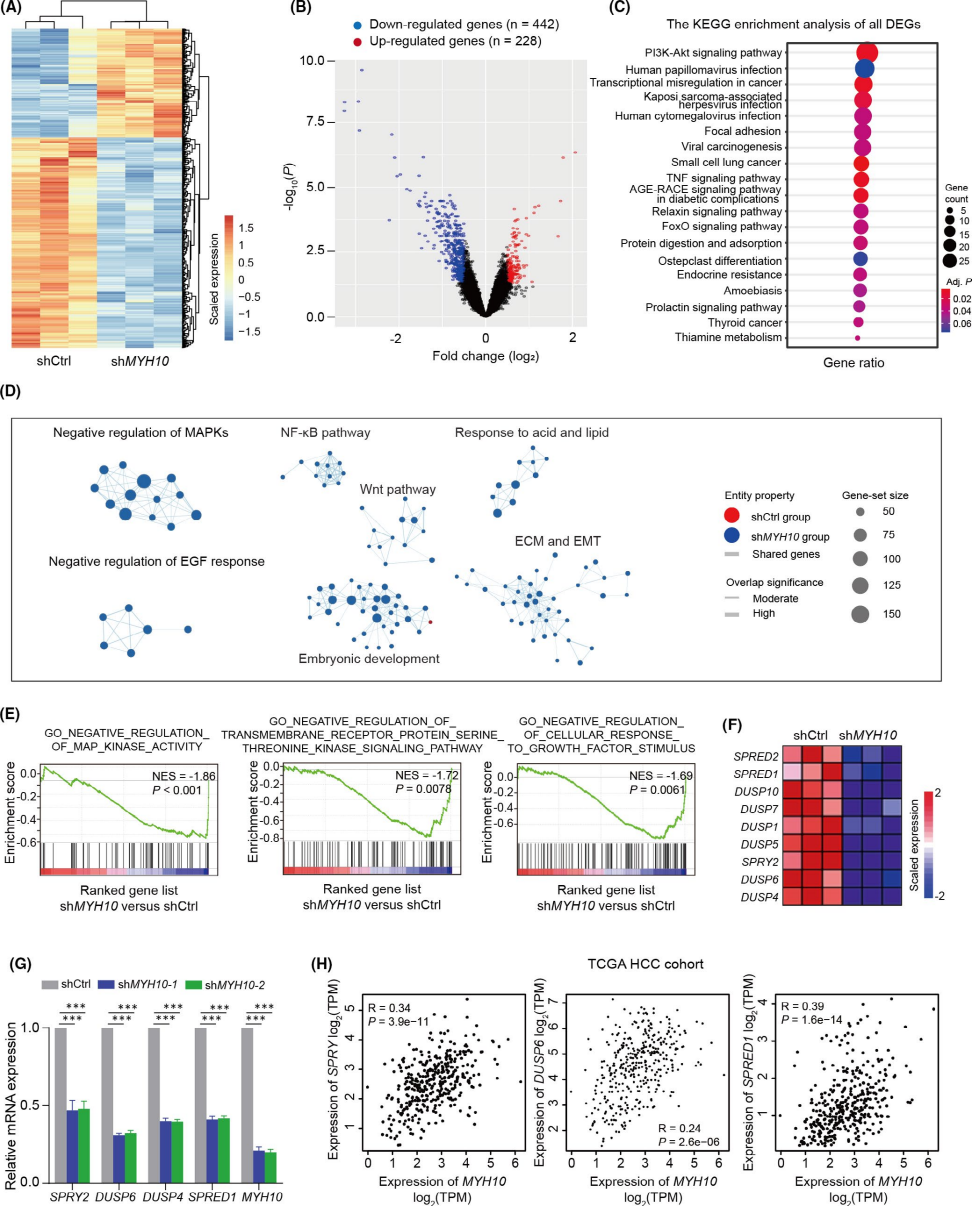
Depletion of *MYH10* induced activation of EGFR pathway. (A and B) The heatmap (A) and volcano plot (B) of the differential expressed genes (DGEs) in *MYH10* knockdown HepG2 cells compared to the control cells. Significantly up‐regulated genes are indicated in red; down‐regulated genes are shown in blue. (C) KEGG pathway that was significantly enriched by DEGs in (B). Adjusted *p* (false discovery rate [FDR]) <0.05 was considered to be statistically significant. (D) The enrichment map of the GSEA results based on the gene expression profiles of *MYH10*‐depleted and control HepG2 cells. Nodes represent significant gene sets (*p* < 0.05), and node size represents the number of genes in the gene set. Edges represent the similarity between gene sets. Edge width represents the number of genes that overlap between a pair of gene sets. (E) GSEA plots of gene sets involved in negative regulation of EGF response/MAPKs subnetworks in (D). NES, normalized enrichment score. (F) The heatmap of dysregulated core genes (negative regulators of EGFR/MAPKs) in the gene sets in (E). (G) Relative mRNA expression levels of the negative regulators of EGFR or MAPKs were confirmed by RT‐qPCR assays in *MYH10*‐depleted HepG2 cells. The quantitative data are mean ± standard deviation (SD) of three independent experiments. ^***^ *p* < 0.001 (Student's *t* test). (H) Pearson correlation analyses of the mRNA levels of *MYH10* and the negative regulators of EGFR or MAPKs in the TCGA‐LIHC cohort. TPM, transcripts per million

Next, we sought to explore the underlying mechanism that MYH10 promotes HCC cells migration, invasion and metastasis. To achieve this, we described by mRNA expression profiles the genes with altered mRNA expression in *MYH10*‐depleted HepG2 cells compared to the control cells (Table S4). We identified a total of 442 down‐regulated genes and 228 up‐regulated genes (Figure 5A and B). KEGG enrichment analyses showed that these differentially expressed genes (DEGs) are significantly represented by the phosphatidylinositol 3‐kinase‐AKT serine/threonine kinase 1 (PI3K‐AKT) pathway, virus infection and focal adhesion (Figure 5C). Additionally, gene set enrichment analysis (GSEA) revealed seven functional subnetworks modulated by *MYH10* depletion, including negative regulation of mitogen‐activated protein kinases (MAPKs), negative regulation of EGF response, ECM and EMT, NF‐κB pathway, Wnt pathway and embryonic development, all of which are pronounced in *MYH10*‐depleted cells (Figure 5D and Table S5). These functional enrichment results are consistent with previous findings that MYH10 affects EMT and cell differentiation.^17^, ^26^, ^27^ Notably, among them, negative regulation of EGF response/MAPKs subnetworks was tensely connected, which exhibited significant association between them (Figure 5D and E).

Indeed, the dysfunction of the EGFR pathway has been shown to involve in the malignant transformation and progression of a broad variety of cancers, including HCC.^28^, ^29^, ^30^ Therefore, we hypothesized that MYH10 exerts its tumour‐suppressive role by reducing the activation of EGFR pathway. Indeed, we observed that several key negative regulators of EGFR or MAPKs are significantly down‐regulated in *MYH10*‐depleted cells (Figure 5F), including *Sprouty RTK signalling antagonist 2* (*SPRY2*),^31^ *dual*‐*specificity phosphatases* (*DUSPs*, such as *DUSP6*, *DUSP4*, *DUSP5*, *DUSP1*, *DUSP7* and *DUSP10*)^32^ and *Sprouty related EVH1 domain containing proteins* (*SPREDs*, such as *SPRED1* and *SPRED2*),^33^ which were confirmed by RT‐qPCR assays (Figure 5G). Further, we confirmed in the TCGA‐LIHC cohort that the mRNA expressions of these genes were positively correlated with MYH10 expression (Figure 5H). Taken together, these data suggested that inhibition of EGFR pathway activation may be responsible for the tumour‐suppressive effect of MYH10.

### 3.6 Inhibition of EGFR was required for MYH10’s tumour‐suppressive function

**FIGURE 6:**
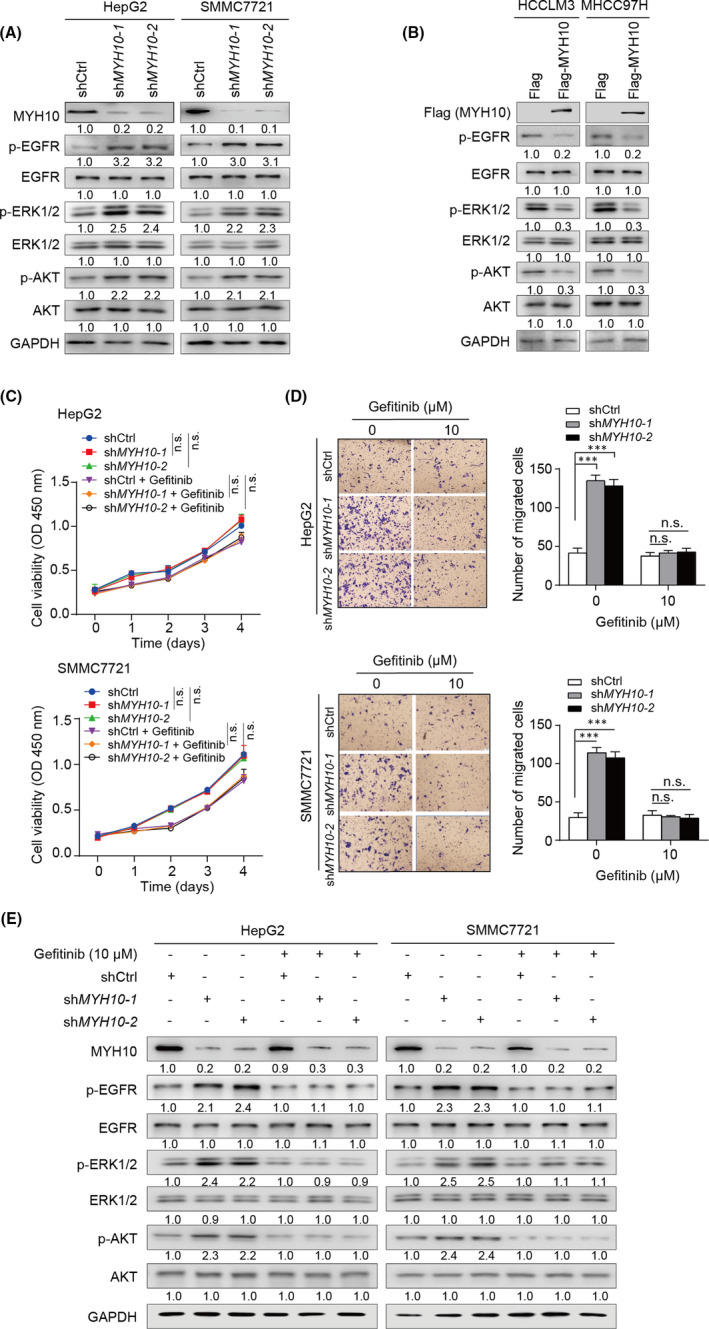
MYH10 exerts its tumour‐suppressive role through inactivation of the EGFR pathway. (A) Knockdown of *MYH10* triggers the activation of the EGFR pathway. The immunobands of EGFR, p‐EGFR (Tyr1068), ERK1/2, p‐ERK1/2 (Thr202/Tyr204), AKT and p‐AKT (Ser473) were detected by immunoblotting assays in L‐02, HepG2 and SMMC7721 cells. (B) Overexpression of MYH10 inhibits EGFR pathway in HCCLM3 and MHCC97H cells. (C) Knockdown of *MYH10* has no pro‐proliferation effect on HepG2 (top) and SMMC7721 (bottom) cells with or without Gefitinib treatment (10 µM). (D) Gefitinib treatment (10 µM) abolishes the pro‐migratory effects of *MYH10* depletion on HepG2 (top) and SMMC7721 (bottom) cells. (E) Gefitinib treatment (10 µM) eliminates the activation of *MYH10* depletion on EGFR pathway in HepG2 (left) and SMMC7721 (right) cells. ^***^ *p* < 0.001 (Student's *t* test). n.s., not significant

We then explored the modulatory effect of MYH10 on the EGFR pathway in HCC cells. Indeed, we observed that knockdown of *MYH10* in HepG2 and SMMC7721 cells increases the phosphorylation levels of EGFR (p‐EGFR) and its major downstream cascades ERK1/2 (p‐ERK1/2) and AKT (p‐AKT) (Figure 6A). In contrast, overexpression of MYH10 in HCCLM3 and MHCC97H cells significantly reduced the levels of p‐EGFR, p‐ERK1/2 and p‐AKT (Figure 6B). These results suggested that MYH10 inhibits EGFR signalling in HCC cells.

We next explored whether the anti‐tumorigenic function of MYH10 is dependent on the EGFR pathway in HCC cells. Therefore, we treated the *MYH10*‐depleted HCC cells with Gefitinib, a kind of EGFR tyrosine kinase inhibitor, to investigate the changes in cell growth and migration. Consistently, *MYH10* depletion exhibited no significant effects on the growth of HepG2 and SMMC7721 cells either with or without Gefitinib treatment (Figure 6C). However, the Gefitinib treatment significantly limited the pro‐migratory effects of *MYH10* depletion on HepG2 and SMMC7721 cells (Figure 6D). Accordingly, knockdown of *MYH10* in HepG2 and SMMC7721 cells was able to increase the levels of p‐EGFR, p‐ERK and p‐AKT (Figure 6E); however, Gefitinib treatment significantly counteracted the activation of the EGFR pathway caused by *MYH10* deletion (Figure 6E). In conclusion, these results suggest that MYH10 is highly likely to affect HCC progression by inhibiting the EGFR pathway.

## 4 DISCUSSION

In the present study, we clarified a novel candidate tumour suppressor gene *MYH10* driven by 17p13.1 deletions in HCC through an integrative omics analysis. In the TCGA‐LIHC and VALI cohort, *MYH10* is down‐regulated in primary HCC tissues. Meanwhile, lower *MYH10* levels predict worse OS and DFS rates in patients with HCC, supporting that *MYH10* may be a prognostic biomarker for HCC worth more investigation. We further revealed that inhibition of *MYH10* markedly potentiates HCC metastasis in vitro and in vivo. Our results also demonstrated that loss of *MYH10* promotes HCC metastasis by enhancing the activation of the EGFR pathway.

Given the high prevalence of CNAs and their pivotal prognostic relevance in human cancers, it is important to dissect the underlying mechanisms of CNAs in cancer progression and treatment. Loss of heterozygosity (LOH) on chromosome 17p13.1was found to be a common phenomenon in HCC.^9^, ^34^ Wang et al.^35^ revealed the highest incidence of genomic imbalance at 17p13 (65%) compared to any other chromosome locations. LOH at 17p13.1 has also been observed in several other types of cancer, likewise in lung cancer and colon cancer.^11^ These studies suggested that 17p13.1 plays an important role in the development of tumours, including HCC. It is well known that the 17p13.1 region contains several tumour suppressor genes, such as *TP53* ^11^ and *PHF23*.^36^ However, several other genes are located within the 17p13.1 region and their expressions are significantly correlated with the copy number of 17p13.1, therefore deserving to be investigated. Here, we prioritized a novel candidate tumour suppressor gene *MYH10* within this depleted region through an integrative omics analysis. To our best knowledge, this is the first study to elaborate on the clinical relevance of MYH10 in HCC. We observed that *MYH10* deletion occurred in ~38% of primary HCCs that drives the down‐regulation of MYH10. Furthermore, down‐regulation of MYH10 was significantly associated with poor outcomes of HCC patients. Our results also indicated that *MYH10* deletion is a trans‐cancer genomic feature and deserves more attention on its biological and clinical relevance in multi types of cancer.

MYH10 is one of the isoforms of non‐muscle myosin II, and has been known to participate in cell adhesion and migration.^14^ Although several studies provide a close linkage between MYH10 and tumorigenesis, the roles of MYH10 are controversial. For instance, MYH10 could promote metastasis through accelerating initial rates of lamellar spreading in breast cancer.^15^ In contrast, in nasopharyngeal carcinoma, MYH10 was down‐regulated by miR‐200a and was shown to inhibit cell migration and invasion.^16^ Here, we revealed the anti‐metastatic role of MYH10 in HCC cells through loss‐of‐ and gain‐of‐function experimental assays. These discordances might be due to tumour heterogeneity that MYH10 exerts its role in a context‐dependent manner. Further studies are warranted to explore the functional consequence of MYH10 in different types of cancer. Our findings highlighted that MYH10 is a novel tumour suppressor of HCC, providing an important supplement to the mechanism of HCC development and metastasis.

It is widely accepted that the EGFR pathway, as well as downstream networks involving MEK‐ERK and PI3K‐AKT, was hyperactivated and played important roles in promoting tumour metastasis of multiple cancers, including HCC.^29^, ^37^, ^38^ Here, the EGFR pathway was identified as the leading pathway modulated by MYH10. Further, we revealed that several negative regulators of EGFR or MAPKs, including SPRY2, DUSPs and SPREDs,^31^, ^32^, ^33^ are down‐regulated by *MYH10* depletion. These negative regulators acted at different cascades of the EGFR signalling pathway. For instance, SPRY2 can antagonize EGFR activity by inhibiting downstream ERK activation.^39^ DUSPs often inhibit EGFR activation by blocking the PI3K‐AKT signalling.^40^ SPRED proteins modulate EGFR signalling by inhibiting the RAS/ERK pathway.^41^ However, the aberrant expression of these negative regulators in HCC is largely unknown. Our findings suggested that MYH10 is a novel repressor of the EGFR pathway through inducing the expression of these negative regulators, thus possessing a novel therapeutic vulnerability in HCCs with EGFR hypoactivation.

## 5 CONCLUSIONS

In conclusion, the survey revealed that, for the first time, MYH10 functions as a promising tumour suppressor driven by copy number deletion at 17p13.1 in the development of HCC. Depletion or down‐regulation of *MYH10* suggests worse outcomes in HCC patients. Depletion of *MYH10* triggered activation of the EGFR pathway, which in turn promoted metastasis of HCC cells. More evidence is necessary to elucidate the mechanism of MYH10 in HCC, which may be beneficial to develop a novel treatment strategy for this malignancy.

## CONFLICT OF INTERESTS

No competing financial interests exist.

## AUTHOR CONTRIBUTIONS

**Qian Jin:** Validation (equal); Visualization (equal); Writing‐original draft (equal). **Min Cheng:** Data curation (equal). **Xia Xia:** Data curation (equal). **Yuqing Han:** Validation (equal). **Jing Zhang:** Investigation (equal). **Pengbo Cao:** Conceptualization (equal); Visualization (equal); Writing‐original draft (equal). **Gangqiao Zhou:** Conceptualization (equal); Funding acquisition (equal).

## ETHICAL APPROVAL

The studies involving human participants were reviewed and approved by the Medical Ethical Committee of Beijing Institute of Radiation Medicine (Beijing, China). The animal study was reviewed and approved by the Animal Ethics Committee of Beijing Institute of Radiation Medicine (Beijing, China).

## Supporting information

Supplementary Material

## Data Availability

The gene expression profile datasets of *MYH10*‐depleted HepG2 cells and control HepG2 cells have been deposited in GEO (Accession number GSE109358). All the other data that support the findings of this study are available upon request from the corresponding author.
