# Supplementary material for: Down‐regulation of MYH10 driven by chromosome 17p13.1 deletion promotes hepatocellular carcinoma metastasis through activation of the EGFR pathway
Source: J Cell Mol Med. 2021 Nov 4;25(24):11142–56. doi: 10.1111/jcmm.17036 (PMC8650048; doi:10.1111/jcmm.17036)
Supplement: Supplementary file 1 — Supplementary Material [file JCMM-25-11142-s001.docx]

**Supplementary Figure S1: The PRISMA flow of the screening of datasets from the HCCDB database for use as MYH10 differential expression.**

**
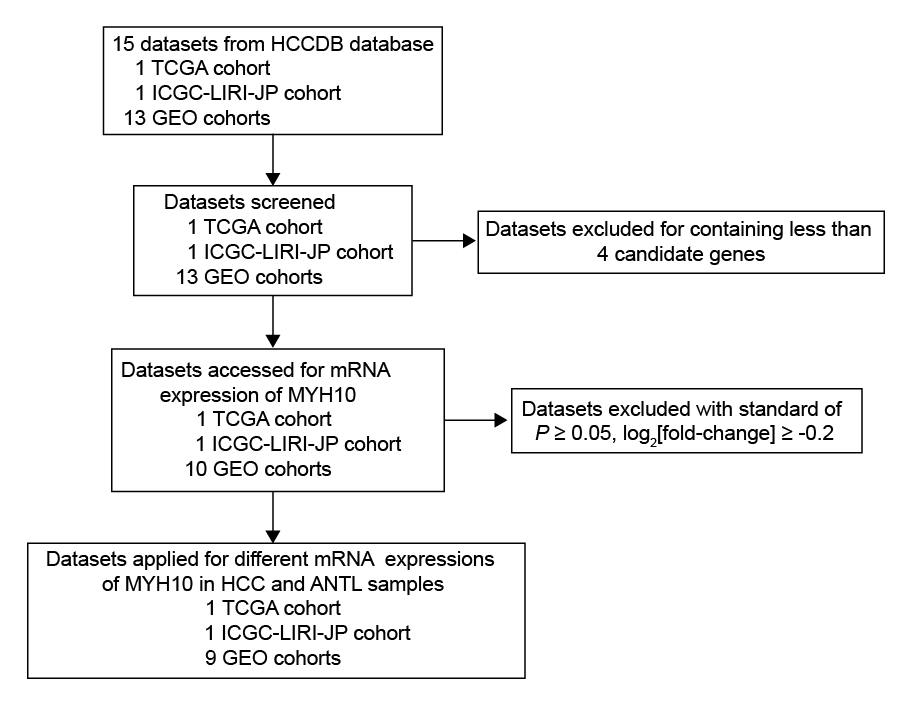
**

**Supplementary Table S1: Comparisons of pathological parameters between the groups of "high" and "low" MYH10 expression in HCC patients from the validation cohort.**

| **Variables** | **Protein levels of MYH10** | | ***P*^a^** |
| --- | --- | --- | --- |
|  | **High (n = 60)** | **Low (n = 94)** |  |
|  | **No. of patients (%)** | **No. of patients (%)** |  |
| **Sex** |  |  |  |
| Male | 51 (85.00) | 78 (82.98) | 0.74 |
| Female | 9 (15.00) | 16 (17.02) |  |
| **Age**, **years** |  |  |  |
| Old (> 50) | 30 (50.00) | 47 (50.00) | 1.00 |
| Young (≤ 50) | 30 (50.00) | 47 (50.00) |  |
| **ALT, U/L** |  |  |  |
| High (> 50) | 24 (40.00) | 30 (31.91) | 0.23 |
| Low (≤ 50) | 27 (45.00) | 52 (55.32) |  |
| NA | 9 (15.00) | 12 (12.77) |  |
| **AFP, ng/mL** |  |  |  |
| High (> 200) | 17 (28.33) | 40 (42.55) | 0.14 |
| Low (≤ 200) | 37 (61.67) | 51 (54.26) |  |
| NA | 6 (10.00) | 3 (3.19) |  |
| **Cirrhosis** |  |  |  |
| Positive | 29 (48.33) | 38 (40.43) | 0.26 |
| Negative | 27 (45.00) | 52 (55.32) |  |
| NA | 4 (6.67) | 4 (4.26) |  |
| **Tumor size, cm** |  |  |  |
| > 5 | 26 (43.33) | 49 (52.13) | 0.62 |
| ≤ 5 | 21 (35.00) | 33 (35.11) |  |
| NA | 13 (21.67) | 12 (12.77) |  |
| **No. of nodules** |  |  |  |
| Multiple | 9 (15.00) | 21 (22.34) | 0.36 |
| Single | 35 (58.33) | 54 (57.45) |  |
| NA | 16 (26.67) | 19 (20.21) |  |
| **TNM stage** |  |  |  |
| III-IV | 10 (16.67) | 19 (20.21) | 0.55 |
| I-II | 43 (71.67) | 63 (67.02) |  |
| NA | 7 (11.67) | 12 (12.77) |  |
| **Tumour capsule** |  |  |  |
| Positive | 27 (45.00) | 48 (51.06) | 0.49 |
| Negative | 15 (25.00) | 20 (21.28) |  |
| NA | 18 (30.00) | 26 (27.66) |  |
| **Vascular invasion** |  |  |  |
| Positive | 10 (16.67) | 32 (34.04) | **0.031** |
| Negative | 43 (71.67) | 57 (60.64) |  |
| NA | 7 (11.67) | 5 (5.32) |  |
| ^a^*P* values were caculated by *χ*^2^ test. *P* < 0.05 was considered to be statistically significant. The immunohistochemistry score for MYH10 greater than 3 were considered as “High” MYH10 expression (n = 60), whereas the others were “Low” MYH10 expression (n = 94). AFP, alpha-fetoprotein; ALT, alanine transaminase; TNM, tumor-node-metastasis; NA, not available. | | | |

**Supplementary Table S2: Pearson correlations between the mRNA levels of each down-regulated gene and the copy numbers of 17p13.1.**

| **Genes** | ***R*** | ***P*** |
| --- | --- | --- |
| *SCO1* | 0.5 | 1E-500 |
| *CNTROB* | 0.47 | 1E-470 |
| *CYB5D1* | 0.46 | 1E-460 |
| *TMEM107* | 0.46 | 1E-461 |
| *STX8* | 0.45 | 1E-450 |
| *BORCS6* | 0.42 | 1E-420 |
| *TRAPPC1* | 0.42 | 1E-421 |
| *CTC1* | 0.37 | 8.35E-312 |
| *KDM6B* | 0.36 | 3.82E-293 |
| *NDEL1* | 0.36 | 3.76E-300 |
| *PFAS* | 0.35 | 5.54E-276 |
| *WRAP53* | 0.34 | 8.74E-266 |
| *KRBA2* | 0.33 | 2.43E-244 |
| *ADPRM* | 0.31 | 1.93E-222 |
| *MYH10* | 0.3 | 1.97E-208 |
| *CHD3* | 0.28 | 1.42E-183 |
| *PER1* | 0.28 | 3.44E-172 |
| *VAMP2* | 0.27 | 9.53E-159 |
| *LINC00324* | 0.25 | 8.28E-142 |
| *NAA38* | 0.25 | 5.57E-140 |
| *KCNAB3* | 0.16 | 1.40E-58 |
| *NTN1* | 0.16 | 4.16E-59 |
| *EFNB3* | 0.14 | 3.74E-45 |
| *HES7* | 0.14 | 1.08E-39 |
| *TMEM220* | 0.14 | 8.95E-47 |
| *AURKB* | 0.12 | 1.03E-30 |
| *GAS7* | 0.12 | 1.48E-30 |
| *PIK3R5* | 0.12 | 1.32E-32 |
| *ARHGEF15* | 0.09 | 3.05E-19 |
| *SLC25A35* | 0.08 | 6.94E-17 |
| *ALOXE3* | 0.06 | 5.31E-09 |
| *GUCY2D* | 0.06 | 1.17E-10 |
| *CFAP52* | 0.06 | 3.07E-08 |
| *ALOX12B* | 0.05 | 1.71E-06 |
| *DNAH2* | 0.05 | 7.80E-08 |
| *CCDC42* | 0.04 | 0.000559 |
| *GLP2R* | 0.04 | 8.31E-05 |
| *MFSD6L* | 0.04 | 4.80E-05 |
| *MYH13* | 0.04 | 0.00011 |
| *ALOX15B* | 0.03 | 0.00061 |
| *MYH1* | 0.03 | 0.0049 |
| *MYH2* | 0.02 | 0.11 |
| *MYH4* | 0.01 | 0.54 |
| *MYH8* | 0.01 | 0.52 |
| *RCVRN* | 0.01 | 0.48 |
| *MYH3* | 0 | 0.93 |
| *ODF4* | 0 | 0.98 |
| The rank of Pearson correlations between the mRNA levels of each protein-coding genes (n = 47) within the focally deleted region at 17p13.1 and the copy numbers of 17p13.1. *R* values were calculated by Pearson correlation coefficient t test. *R* < 0.2 was considered to be statistically significant. | | |

**Supplementary Table S3: The dysregulation of expressions of 19 cis-regulated genes by 17p13.1 deletions in HCC cohorts.**

| **Genes** | **TCGA-LIHC** | | **ICGC-LIRI-JP** | | **GSE**  **22058** | | **GSE**  **25097** | | **GSE**  **36376** | | **GSE**  **14520** | | **GSE**  **10143** | | **GSE**  **46444** | | **GSE**  **54236** | | **GSE**  **63898** | | **GSE**  **64041** | | **GSE**  **76427** | |
| --- | --- | --- | --- | --- | --- | --- | --- | --- | --- | --- | --- | --- | --- | --- | --- | --- | --- | --- | --- | --- | --- | --- | --- | --- |
|  | **LogFC** | ***P*** | **LogFC** | ***P*** | **LogFC** | ***P*** | **LogFC** | ***P*** | **LogFC** | ***P*** | **LogFC** | ***P*** | **LogFC** | ***P*** | **LogFC** | ***P*** | **LogFC** | ***P*** | **LogFC** | ***P*** | **LogFC** | ***P*** | **LogFC** | ***P*** |
| *MYH10* | -9E-01 | 4E-20 | -3E-01 | 6E-05 | -5E-01 | 4E-10 | -9E-01 | 2E-17 | 8E-02 | 2E-01 | -4E-01 | 3E-09 | -3E-01 | 2E-04 | -9E-01 | 2E-02 | -4E-01 | 8E-04 | -6E-01 | 3E-15 | -4E-01 | 1E-04 | -5E-01 | 3E-07 |
| *PER1* | -1E+00 | 7E-12 | -4E-01 | 2E-06 | -5E-01 | 3E-13 | -2E-01 | 5E-22 | -5E-02 | 1E-02 | -2E+00 | 3E-96 | -5E-01 | 4E-05 | -6E-01 | 7E-03 | -2E-01 | 3E-02 | -5E-02 | 7E-02 | -4E-01 | 2E-04 | -4E-01 | 1E-08 |
| *NDEL1* | -9E-01 | 7E-24 | -4E-01 | 3E-13 | -5E-01 | 2E-13 | -3E-01 | 2E-55 | -5E-02 | 8E-02 | -7E-01 | 2E-43 | -2E-01 | 7E-04 | -5E-02 | 9E-01 | -4E-01 | 5E-09 | -6E-01 | 6E-39 | -2E-01 | 2E-04 | -4E-01 | 2E-07 |
| *KDM6B* | -1E+00 | 2E-16 | -6E-01 | 4E-16 | -5E-01 | 8E-16 | -6E-01 | 3E-23 | 6E-03 | 7E-01 | -6E-01 | 3E-43 | NA | NA | -5E-01 | 2E-02 | -1E-02 | 1E+00 | -3E-01 | 6E-19 | -8E-02 | 1E-02 | -2E-01 | 7E-06 |
| *CYB5D1* | -7E-01 | 1E-24 | -2E-01 | 4E-08 | -7E-01 | 3E-17 | -4E-01 | 7E-28 | 3E-02 | 5E-01 | NA | NA | NA | NA | 3E-01 | 2E-01 | -2E-01 | 4E-02 | -2E-01 | 1E-14 | -3E-01 | 2E-07 | -2E-01 | 9E-04 |
| *ADPRM* | -2E-01 | 9E-03 | 7E-02 | 7E-02 | -8E-02 | 5E-01 | 3E-03 | 2E-01 | 8E-02 | 2E-02 | 4E-02 | 4E-01 | NA | NA | -3E-01 | 3E-01 | -2E-01 | 4E-02 | 7E-02 | 2E-01 | -6E-02 | 4E-01 | 1E-02 | 9E-01 |
| *SCO1* | -4E-01 | 4E-10 | 1E-01 | 2E-02 | -4E-01 | 6E-11 | -5E-01 | 1E-14 | 1E-01 | 3E-02 | NA | NA | NA | NA | -6E-02 | 8E-01 | -4E-01 | 2E-08 | -7E-02 | 2E-01 | -1E-01 | 4E-02 | 2E-01 | 9E-03 |
| *CNTROB* | 2E-01 | 2E-06 | 5E-01 | 1E-21 | 3E-01 | 2E-06 | 2E-01 | 5E-08 | 1E-01 | 1E-08 | NA | NA | NA | NA | -1E-02 | 9E-01 | 4E-02 | 5E-01 | -2E-02 | 2E-01 | 5E-02 | 2E-01 | 7E-02 | 2E-02 |
| *TMEM107* | 3E-01 | 9E-04 | 3E-01 | 6E-14 | 4E-01 | 7E-05 | 1E-02 | 2E-11 | -3E-01 | 2E-11 | NA | NA | NA | NA | -5E-01 | 2E-02 | -1E-01 | 3E-01 | 1E-02 | 7E-01 | 3E-02 | 6E-01 | -7E-01 | 2E-08 |
| *STX8* | -1E-01 | 2E-01 | 2E-01 | 8E-05 | -2E-01 | 1E-03 | -3E-01 | 4E-05 | 1E-01 | 2E-02 | -1E-01 | 6E-02 | NA | NA | -1E+00 | 3E-04 | -2E-01 | 3E-02 | -2E-01 | 2E-08 | -2E-02 | 8E-01 | 1E-01 | 4E-02 |
| *BORCS6* | 6E-01 | 3E-14 | 7E-01 | 5E-33 | 4E-01 | 3E-12 | 2E-01 | 2E-17 | 3E-01 | 9E-33 | -8E-02 | 3E-05 | NA | NA | -4E-01 | 1E-01 | 1E-01 | 4E-02 | 2E-01 | 2E-09 | 6E-02 | 2E-01 | 8E-02 | 4E-02 |
| *TRAPPC1* | 2E-01 | 3E-03 | 5E-01 | 2E-17 | -5E-02 | 4E-01 | NA | NA | 6E-01 | 3E-31 | NA | NA | NA | NA | -1E-01 | 9E-01 | -1E-02 | 9E-01 | 1E-01 | 4E-03 | -1E-02 | 8E-01 | 3E-02 | 6E-01 |
| *CTC1* | -2E-01 | 2E-02 | 1E-01 | 4E-02 | -1E-01 | 6E-02 | -4E-03 | 6E-01 | NA | NA | -1E-01 | 2E-03 | NA | NA | -6E-01 | 2E-02 | -8E-02 | 2E-01 | -1E-01 | 6E-04 | -1E-01 | 1E-02 | NA | NA |
| *PFAS* | 5E-01 | 8E-15 | 5E-01 | 2E-30 | 4E-01 | 8E-11 | 6E-01 | 5E-22 | 6E-01 | 4E-43 | 5E-01 | 1E-20 | NA | NA | -9E-02 | 7E-01 | 4E-01 | 2E-05 | 2E-02 | 4E-01 | 2E-01 | 2E-04 | 3E-01 | 1E-05 |
| *WRAP53* | 3E-01 | 3E-07 | 4E-01 | 2E-23 | 2E-01 | 9E-08 | 4E-02 | 8E-12 | 3E-01 | 7E-21 | 2E-03 | 9E-01 | NA | NA | 1E-01 | 6E-01 | 2E-01 | 1E-02 | 1E-01 | 1E-05 | 7E-02 | 7E-02 | -1E-01 | 4E-02 |
| *KRBA2* | 1E-01 | 4E-01 | 1E-01 | 3E-08 | 5E-02 | 3E-01 | 9E-04 | 2E-01 | 7E-02 | 4E-07 | NA | NA | NA | NA | -4E-01 | 3E-01 | NA | NA | 1E-01 | 1E-05 | -4E-02 | 2E-01 | 1E-01 | 6E-06 |
| *CHD3* | 6E-01 | 2E-06 | 3E-01 | 7E-05 | 2E-01 | 1E-01 | 2E-01 | 2E-05 | 4E-02 | 5E-03 | 8E-02 | 1E-02 | NA | NA | -2E-01 | 2E-02 | 7E-02 | 5E-01 | 0E+00 | 1E+00 | 7E-03 | 9E-01 | -5E-02 | 3E-02 |
| *VAMP2* | -6E-01 | 5E-17 | 1E-01 | 3E-02 | -3E-01 | 2E-07 | -3E-01 | 2E-11 | 4E-02 | 3E-01 | -4E-01 | 3E-23 | -2E-02 | 8E-01 | -1E+00 | 1E-04 | -2E-01 | 3E-02 | 2E-02 | 5E-01 | -2E-01 | 2E-03 | -2E-02 | 7E-01 |
| *NAA38* | -1E-01 | 2E-01 | 2E-01 | 3E-05 | -2E-01 | 2E-06 | -4E-02 | 4E-09 | 6E-02 | 2E-01 | NA | NA | NA | NA | 2E-01 | 5E-01 | 2E-01 | 2E-02 | 3E-02 | 6E-01 | -6E-02 | 1E-01 | 9E-02 | 3E-01 |
| The mRNA levels of 19 cis-regulated genes by 17p13.1 deletions in HCC tissues and adjacent non-tumor tissues across 12 cohorts were obtained from the HCCDB database (http://lifeome.net/database/hccdb). LogFC, log2-transformed fold change of gene expression levels in HCC tissues relative to adjacent non-tumor tissues. *P* values were calculated by Student's *t* test. *P* < 0.05 was considered to be statistically significant. NA, not available. | | | | | | | | | | | | | | | | | | | | | | | | |

**Supplementary Table S4: The list of dysregulated genes in HepG2 cells upon knockdown of MYH10.**

| **Genes** | **shCtrl-rep1** | **shCtrl-rep2** | **shCtrl-rep3** | **sh*MYH10***  **-rep1** | **sh*MYH10***  **-rep2** | **sh*MYH10***  **-rep3** | **LogFC** | ***P*** | **Adjusted *P*** |
| --- | --- | --- | --- | --- | --- | --- | --- | --- | --- |
| *MYH10* | 1059.21 | 1149.39 | 1102.92 | 133.12 | 146.03 | 148.31 | -2.86E+00 | 1.46E-14 | 2.94E-10 |
| *SERPINE2* | 894.30 | 848.91 | 779.61 | 88.08 | 113.95 | 108.13 | -2.94E+00 | 4.81E-13 | 4.85E-09 |
| *COL5A2* | 102.73 | 111.30 | 100.07 | 10.79 | 9.02 | 11.84 | -3.26E+00 | 7.61E-13 | 5.12E-09 |
| *DUSP4* | 159.64 | 194.88 | 168.56 | 14.38 | 19.21 | 19.37 | -3.26E+00 | 2.19E-12 | 1.10E-08 |
| *FZD10* | 74.38 | 71.88 | 58.52 | 9.31 | 7.41 | 9.31 | -2.92E+00 | 1.59E-11 | 6.43E-08 |
| *ABCC3* | 390.06 | 500.60 | 452.27 | 94.11 | 80.27 | 101.60 | -2.17E+00 | 2.71E-11 | 9.11E-08 |
| *NTS* | 584.47 | 613.66 | 612.96 | 2908.66 | 1996.73 | 2565.17 | 2.07E+00 | 1.57E-10 | 4.52E-07 |
| *ASNS* | 920.51 | 955.78 | 943.39 | 337.34 | 356.94 | 284.94 | -1.44E+00 | 2.71E-10 | 6.83E-07 |
| *CGA* | 50.83 | 51.58 | 56.72 | 192.26 | 160.76 | 171.93 | 1.79E+00 | 3.51E-10 | 7.09E-07 |
| *LAMP3* | 128.96 | 140.48 | 131.58 | 27.14 | 36.94 | 26.79 | -2.10E+00 | 3.25E-10 | 7.09E-07 |
| *GRB10* | 170.23 | 220.24 | 217.55 | 40.02 | 51.19 | 56.42 | -1.97E+00 | 1.75E-09 | 3.21E-06 |
| *SDR16C5* | 103.50 | 116.41 | 95.78 | 35.90 | 33.11 | 36.07 | -1.54E+00 | 2.12E-09 | 3.57E-06 |
| *COL15A1* | 816.66 | 725.04 | 738.36 | 125.54 | 184.96 | 215.60 | -2.05E+00 | 2.39E-09 | 3.71E-06 |
| *ARNT2* | 56.72 | 81.75 | 65.74 | 16.67 | 20.23 | 20.23 | -1.82E+00 | 8.29E-09 | 1.20E-05 |
| *TSPAN8* | 97.10 | 127.98 | 132.04 | 38.97 | 28.71 | 34.38 | -1.75E+00 | 1.01E-08 | 1.35E-05 |
| *NUPR1* | 264.36 | 317.34 | 304.68 | 101.44 | 120.77 | 88.25 | -1.39E+00 | 1.32E-08 | 1.66E-05 |
| *ASS1* | 2070.13 | 2263.18 | 2160.24 | 1148.90 | 1219.81 | 1236.18 | -8.30E-01 | 1.71E-08 | 2.03E-05 |
| *HPD* | 91.94 | 92.10 | 76.27 | 31.78 | 29.97 | 33.35 | -1.41E+00 | 2.10E-08 | 2.36E-05 |
| *ABCG2* | 237.54 | 263.94 | 270.77 | 125.17 | 119.39 | 113.10 | -9.76E-01 | 3.90E-08 | 3.39E-05 |
| *ADM* | 521.60 | 601.53 | 562.33 | 222.20 | 277.44 | 272.23 | -1.01E+00 | 4.29E-08 | 3.39E-05 |
| *COL4A1* | 154.11 | 164.61 | 159.88 | 64.38 | 71.53 | 77.27 | -1.06E+00 | 4.37E-08 | 3.39E-05 |
| *GPR87* | 16.50 | 22.20 | 17.55 | 6.87 | 6.64 | 6.39 | -1.52E+00 | 3.49E-08 | 3.39E-05 |
| *HTRA1* | 798.65 | 904.37 | 887.58 | 459.85 | 469.72 | 459.60 | -8.12E-01 | 3.48E-08 | 3.39E-05 |
| *IER3* | 1558.52 | 2017.88 | 1873.45 | 640.27 | 867.76 | 725.77 | -1.25E+00 | 4.27E-08 | 3.39E-05 |
| *NUAK1* | 232.02 | 283.26 | 261.85 | 110.91 | 125.84 | 107.51 | -1.04E+00 | 3.78E-08 | 3.39E-05 |
| *PCK1* | 24.90 | 34.59 | 30.59 | 84.31 | 92.51 | 120.43 | 1.73E+00 | 4.21E-08 | 3.39E-05 |
| *IL13RA1* | 275.05 | 328.16 | 308.90 | 144.18 | 146.30 | 155.71 | -8.85E-01 | 5.56E-08 | 4.15E-05 |
| *PHGDH* | 932.74 | 1066.55 | 986.85 | 565.37 | 557.62 | 550.13 | -7.72E-01 | 5.76E-08 | 4.15E-05 |
| *INHBE* | 27.38 | 37.75 | 30.88 | 9.78 | 11.61 | 11.73 | -1.56E+00 | 6.19E-08 | 4.30E-05 |
| *USP46* | 88.39 | 91.61 | 77.63 | 28.04 | 32.77 | 36.27 | -1.38E+00 | 6.40E-08 | 4.30E-05 |
| *FHL2* | 1378.46 | 1413.40 | 1365.49 | 800.71 | 839.29 | 710.80 | -7.76E-01 | 7.65E-08 | 4.98E-05 |
| *PTGS2* | 37.84 | 45.57 | 39.27 | 14.30 | 11.93 | 17.15 | -1.54E+00 | 8.01E-08 | 5.05E-05 |
| *VAPA* | 3289.19 | 3402.90 | 3559.63 | 2169.66 | 2157.89 | 2014.70 | -7.16E-01 | 8.52E-08 | 5.21E-05 |
| *BIRC3* | 58.92 | 49.55 | 52.68 | 25.02 | 20.80 | 20.67 | -1.30E+00 | 9.40E-08 | 5.27E-05 |
| *SLC12A7* | 112.35 | 104.95 | 97.42 | 238.79 | 193.11 | 252.55 | 1.22E+00 | 8.99E-08 | 5.27E-05 |
| *STC2* | 59.21 | 76.26 | 72.78 | 27.94 | 27.05 | 30.70 | -1.26E+00 | 9.20E-08 | 5.27E-05 |
| *EPAS1* | 658.28 | 650.07 | 665.98 | 271.20 | 364.79 | 292.55 | -9.93E-01 | 1.03E-07 | 5.48E-05 |
| *MAFB* | 67.77 | 77.36 | 69.43 | 25.37 | 19.76 | 30.35 | -1.52E+00 | 1.03E-07 | 5.48E-05 |
| *TENM2* | 26.31 | 29.31 | 31.88 | 11.19 | 10.76 | 13.08 | -1.37E+00 | 1.13E-07 | 5.84E-05 |
| *IGFBP7* | 1774.72 | 2177.82 | 2050.76 | 1030.71 | 1112.29 | 906.80 | -9.43E-01 | 1.20E-07 | 5.90E-05 |
| *KLRC3* | 135.92 | 150.60 | 132.86 | 302.57 | 238.43 | 310.04 | 1.14E+00 | 1.17E-07 | 5.90E-05 |
| *PDIA6* | 2573.60 | 2376.01 | 2487.92 | 1356.44 | 1587.37 | 1475.96 | -7.49E-01 | 1.28E-07 | 6.13E-05 |
| *CARD6* | 93.42 | 126.72 | 99.04 | 46.39 | 43.48 | 47.19 | -1.15E+00 | 1.46E-07 | 6.83E-05 |
| *H2BC12* | 1122.28 | 1194.53 | 1183.10 | 1935.10 | 1764.29 | 1740.27 | 6.60E-01 | 1.52E-07 | 6.90E-05 |
| *YARS1* | 1277.43 | 1369.47 | 1281.72 | 723.03 | 820.05 | 771.76 | -7.04E-01 | 1.54E-07 | 6.90E-05 |
| *SLC35F3* | 38.69 | 37.21 | 35.44 | 17.11 | 18.69 | 17.38 | -1.12E+00 | 2.04E-07 | 8.95E-05 |
| *AMIGO2* | 82.94 | 74.92 | 70.45 | 25.52 | 32.25 | 33.20 | -1.32E+00 | 2.13E-07 | 9.16E-05 |
| *DERL1* | 68.27 | 58.35 | 66.38 | 28.77 | 31.31 | 27.81 | -1.13E+00 | 2.35E-07 | 9.77E-05 |
| *PPDPFL* | 78.29 | 88.87 | 70.37 | 173.10 | 137.36 | 169.40 | 1.11E+00 | 2.37E-07 | 9.77E-05 |
| *C3* | 116.27 | 128.92 | 147.40 | 55.11 | 64.40 | 56.40 | -1.06E+00 | 2.53E-07 | 1.02E-04 |
| *CCDC113* | 74.84 | 93.51 | 93.72 | 36.81 | 40.02 | 41.74 | -1.10E+00 | 2.65E-07 | 1.05E-04 |
| *SLCO4A1* | 489.32 | 515.60 | 510.70 | 254.22 | 260.96 | 300.29 | -7.73E-01 | 2.80E-07 | 1.07E-04 |
| *SPRED2* | 112.16 | 108.77 | 104.98 | 51.08 | 55.24 | 55.54 | -9.35E-01 | 2.77E-07 | 1.07E-04 |
| *TGM2* | 134.52 | 129.30 | 130.08 | 65.40 | 49.36 | 59.52 | -1.09E+00 | 2.91E-07 | 1.09E-04 |
| *CASP4* | 106.42 | 133.97 | 128.35 | 60.53 | 60.85 | 60.99 | -9.12E-01 | 3.28E-07 | 1.18E-04 |
| *RPIA* | 244.43 | 239.25 | 239.06 | 116.07 | 129.90 | 131.79 | -8.05E-01 | 3.24E-07 | 1.18E-04 |
| *COL12A1* | 1080.29 | 1178.89 | 1106.33 | 510.61 | 674.58 | 598.82 | -8.64E-01 | 3.77E-07 | 1.32E-04 |
| *DUSP6* | 177.44 | 170.27 | 143.39 | 73.83 | 75.97 | 76.05 | -9.95E-01 | 3.80E-07 | 1.32E-04 |
| *DUSP1* | 2207.88 | 2445.03 | 2566.21 | 1421.69 | 1503.61 | 1471.81 | -6.99E-01 | 3.95E-07 | 1.33E-04 |
| *GARS1* | 2065.49 | 2120.27 | 2086.27 | 1248.74 | 1455.07 | 1267.23 | -6.41E-01 | 3.88E-07 | 1.33E-04 |
| *PPP2R1B* | 743.21 | 809.42 | 763.57 | 425.37 | 481.27 | 461.28 | -6.74E-01 | 4.07E-07 | 1.35E-04 |
| *COL4A2* | 292.14 | 370.09 | 338.73 | 142.13 | 170.70 | 176.72 | -8.95E-01 | 4.41E-07 | 1.43E-04 |
| *SLC26A2* | 240.10 | 254.88 | 220.55 | 115.29 | 133.78 | 114.69 | -8.49E-01 | 4.73E-07 | 1.51E-04 |
| *CXCL8* | 16.65 | 19.05 | 19.05 | 7.74 | 9.60 | 7.75 | -1.19E+00 | 5.05E-07 | 1.59E-04 |
| *HSPB8* | 358.94 | 313.78 | 323.74 | 629.79 | 518.27 | 625.93 | 9.38E-01 | 5.13E-07 | 1.59E-04 |
| *DIO2* | 162.10 | 175.60 | 191.56 | 263.32 | 268.19 | 271.03 | 7.41E-01 | 5.77E-07 | 1.76E-04 |
| *ASZ1* | 99.77 | 56.03 | 52.87 | 19.34 | 10.55 | 13.82 | -2.23E+00 | 6.33E-07 | 1.88E-04 |
| *SELENOW* | 395.70 | 392.77 | 399.45 | 647.66 | 568.60 | 597.60 | 6.99E-01 | 6.30E-07 | 1.88E-04 |
| *WFDC1* | 1412.61 | 1584.12 | 1513.90 | 490.92 | 818.22 | 462.63 | -1.34E+00 | 6.69E-07 | 1.96E-04 |
| *GPRC5A* | 471.57 | 516.66 | 518.28 | 271.45 | 312.60 | 273.30 | -6.94E-01 | 6.88E-07 | 1.98E-04 |
| *LRATD2* | 111.77 | 92.45 | 92.82 | 43.53 | 46.28 | 47.33 | -1.05E+00 | 6.97E-07 | 1.98E-04 |
| *PKIB* | 40.77 | 41.31 | 39.27 | 22.70 | 20.94 | 21.20 | -9.51E-01 | 7.07E-07 | 1.98E-04 |
| *MFAP5* | 832.09 | 963.27 | 955.12 | 1559.27 | 1397.27 | 1296.27 | 6.75E-01 | 7.48E-07 | 2.04E-04 |
| *OSTM1* | 145.88 | 145.82 | 129.85 | 68.10 | 75.94 | 69.47 | -8.68E-01 | 7.41E-07 | 2.04E-04 |
| *TGFBI* | 155.46 | 227.84 | 213.93 | 74.92 | 91.85 | 88.90 | -1.10E+00 | 7.77E-07 | 2.09E-04 |
| *CREG2* | 39.20 | 49.94 | 47.88 | 23.21 | 22.71 | 18.03 | -1.13E+00 | 8.35E-07 | 2.22E-04 |
| *HOXA10* | 165.77 | 195.04 | 220.10 | 95.10 | 99.27 | 93.03 | -8.85E-01 | 9.65E-07 | 2.53E-04 |
| *SERPINB4* | 7.72 | 7.14 | 5.89 | 3.42 | 3.38 | 3.18 | -1.01E+00 | 9.78E-07 | 2.53E-04 |
| *ATP1B3* | 4725.63 | 4809.84 | 4465.52 | 3252.27 | 3582.24 | 3341.69 | -5.08E-01 | 1.02E-06 | 2.57E-04 |
| *CEBPB* | 1307.74 | 1574.38 | 1547.33 | 808.39 | 915.37 | 894.26 | -7.20E-01 | 1.01E-06 | 2.57E-04 |
| *EPHA2* | 218.95 | 251.92 | 238.11 | 128.71 | 135.77 | 140.77 | -6.72E-01 | 1.13E-06 | 2.82E-04 |
| *EMC7* | 789.55 | 856.67 | 837.93 | 525.92 | 546.71 | 476.56 | -6.08E-01 | 1.18E-06 | 2.90E-04 |
| *HEXB* | 1531.69 | 1835.01 | 1860.57 | 970.77 | 1106.61 | 1071.01 | -6.99E-01 | 1.21E-06 | 2.90E-04 |
| *SEL1L3* | 80.91 | 101.14 | 93.48 | 48.93 | 49.95 | 48.15 | -8.36E-01 | 1.21E-06 | 2.90E-04 |
| *CBX1* | 1291.12 | 1269.15 | 1191.41 | 754.26 | 857.75 | 782.60 | -5.99E-01 | 1.27E-06 | 2.94E-04 |
| *TTC13* | 226.06 | 259.36 | 224.36 | 135.25 | 133.44 | 130.98 | -6.92E-01 | 1.25E-06 | 2.94E-04 |
| *UCA1* | 38.39 | 41.43 | 49.61 | 22.95 | 20.94 | 20.88 | -1.03E+00 | 1.25E-06 | 2.94E-04 |
| *FAM133A* | 24.65 | 24.75 | 23.90 | 14.58 | 12.16 | 12.37 | -9.83E-01 | 1.34E-06 | 3.08E-04 |
| *TXNDC12* | 530.66 | 530.02 | 512.96 | 321.70 | 318.52 | 283.36 | -6.55E-01 | 1.38E-06 | 3.13E-04 |
| *GLCE* | 34.30 | 41.93 | 42.39 | 21.62 | 18.49 | 19.99 | -1.03E+00 | 1.43E-06 | 3.21E-04 |
| *PTPRO* | 24.74 | 26.09 | 25.13 | 11.99 | 10.99 | 14.36 | -1.10E+00 | 1.47E-06 | 3.23E-04 |
| *SOWAHC* | 150.83 | 176.62 | 185.70 | 78.77 | 95.03 | 92.76 | -8.28E-01 | 1.49E-06 | 3.23E-04 |
| *TNFRSF21* | 19.84 | 22.54 | 23.42 | 9.34 | 12.05 | 10.56 | -1.11E+00 | 1.48E-06 | 3.23E-04 |
| *PARP12* | 62.09 | 75.86 | 65.13 | 35.64 | 35.64 | 37.26 | -8.77E-01 | 1.54E-06 | 3.30E-04 |
| *USH1C* | 146.63 | 201.75 | 179.10 | 93.42 | 83.03 | 91.08 | -8.44E-01 | 1.72E-06 | 3.65E-04 |
| *CRYBG1* | 36.65 | 34.12 | 43.88 | 18.11 | 21.26 | 17.43 | -1.06E+00 | 1.86E-06 | 3.84E-04 |
| *DRAM1* | 369.00 | 402.27 | 392.19 | 229.60 | 242.60 | 242.59 | -5.86E-01 | 1.89E-06 | 3.84E-04 |
| *H2BS1* | 384.97 | 416.23 | 383.02 | 617.68 | 556.31 | 540.25 | 6.32E-01 | 1.87E-06 | 3.84E-04 |
| *TAP1* | 219.15 | 256.57 | 245.68 | 128.85 | 145.57 | 136.46 | -6.75E-01 | 1.83E-06 | 3.84E-04 |
| *NUDT6* | 98.85 | 106.74 | 113.76 | 62.26 | 55.14 | 55.14 | -8.00E-01 | 1.94E-06 | 3.92E-04 |
| *AKR1B1* | 1524.87 | 1764.05 | 1720.86 | 1119.27 | 1134.05 | 1052.79 | -5.66E-01 | 2.01E-06 | 4.01E-04 |
| *SERPINB3* | 21.53 | 29.67 | 29.81 | 13.88 | 11.16 | 11.12 | -1.20E+00 | 2.03E-06 | 4.02E-04 |
| *CEMIP2* | 188.92 | 194.51 | 185.87 | 109.77 | 106.94 | 111.57 | -6.58E-01 | 2.08E-06 | 4.04E-04 |
| *RPRM* | 210.37 | 270.07 | 274.92 | 370.95 | 388.71 | 381.94 | 7.29E-01 | 2.08E-06 | 4.04E-04 |
| *CACHD1* | 28.12 | 29.30 | 31.50 | 16.93 | 14.67 | 16.99 | -9.40E-01 | 2.54E-06 | 4.87E-04 |
| *IRF2BPL* | 1273.88 | 1267.62 | 1287.75 | 760.30 | 793.44 | 892.17 | -5.97E-01 | 2.63E-06 | 5.01E-04 |
| *GADD45B* | 176.44 | 217.58 | 199.81 | 103.81 | 116.35 | 116.86 | -6.86E-01 | 2.78E-06 | 5.24E-04 |
| *H2BC5* | 380.99 | 469.08 | 467.83 | 705.56 | 628.24 | 628.60 | 6.63E-01 | 3.01E-06 | 5.58E-04 |
| *OGT* | 225.22 | 244.11 | 269.61 | 133.73 | 122.29 | 148.91 | -7.38E-01 | 3.02E-06 | 5.58E-04 |
| *MSMO1* | 385.45 | 373.59 | 335.96 | 551.98 | 554.94 | 499.94 | 6.56E-01 | 3.09E-06 | 5.67E-04 |
| *LRIG1* | 49.35 | 53.46 | 51.06 | 18.33 | 25.43 | 27.29 | -1.15E+00 | 3.14E-06 | 5.70E-04 |
| *SLC7A6* | 71.20 | 82.37 | 77.51 | 38.08 | 46.61 | 39.98 | -8.51E-01 | 3.19E-06 | 5.74E-04 |
| *EGR1* | 459.39 | 459.39 | 445.02 | 246.77 | 253.17 | 294.97 | -6.58E-01 | 3.22E-06 | 5.75E-04 |
| *LOC100506100* | 247.60 | 306.87 | 274.05 | 497.83 | 385.31 | 425.24 | 7.66E-01 | 3.27E-06 | 5.79E-04 |
| *SLU7* | 110.02 | 109.83 | 102.55 | 58.45 | 60.07 | 65.09 | -7.29E-01 | 3.43E-06 | 6.02E-04 |
| *ETV5* | 61.39 | 73.04 | 64.43 | 32.47 | 39.88 | 29.36 | -9.53E-01 | 3.55E-06 | 6.18E-04 |
| *GNG11* | 1468.70 | 1354.32 | 1364.56 | 933.61 | 970.80 | 928.56 | -5.14E-01 | 3.62E-06 | 6.19E-04 |
| *PMEPA1* | 71.31 | 91.49 | 82.13 | 41.73 | 48.05 | 39.51 | -8.71E-01 | 3.59E-06 | 6.19E-04 |
| *OAS1* | 81.16 | 88.67 | 92.32 | 48.64 | 49.94 | 52.45 | -7.35E-01 | 3.67E-06 | 6.22E-04 |
| *TNC* | 26.80 | 12.55 | 15.90 | 6.62 | 5.47 | 6.56 | -1.53E+00 | 3.72E-06 | 6.25E-04 |
| *HACD2* | 1353.12 | 1105.60 | 1124.22 | 653.71 | 753.94 | 707.25 | -7.01E-01 | 3.81E-06 | 6.35E-04 |
| *ALDOC* | 164.09 | 179.48 | 168.18 | 236.63 | 226.08 | 253.40 | 6.11E-01 | 3.93E-06 | 6.51E-04 |
| *ALG2* | 369.96 | 471.22 | 434.77 | 239.97 | 260.94 | 249.53 | -6.39E-01 | 4.05E-06 | 6.54E-04 |
| *UBLCP1* | 129.09 | 127.96 | 119.09 | 73.10 | 68.33 | 73.16 | -7.02E-01 | 4.03E-06 | 6.54E-04 |
| *WWC3* | 452.47 | 526.48 | 500.29 | 291.96 | 300.54 | 322.38 | -5.77E-01 | 4.02E-06 | 6.54E-04 |
| *UCP2* | 23.78 | 20.77 | 21.61 | 39.53 | 49.27 | 44.45 | 9.36E-01 | 4.12E-06 | 6.59E-04 |
| *IL20RB* | 460.94 | 534.56 | 540.27 | 321.43 | 293.43 | 320.09 | -6.04E-01 | 4.27E-06 | 6.78E-04 |
| *NEBL* | 56.64 | 68.39 | 55.00 | 32.35 | 28.42 | 34.64 | -9.09E-01 | 4.47E-06 | 7.04E-04 |
| *ZCCHC24* | 111.80 | 140.94 | 140.69 | 178.28 | 200.12 | 207.15 | 7.11E-01 | 4.55E-06 | 7.11E-04 |
| *JAK1* | 159.93 | 200.96 | 207.12 | 90.04 | 111.47 | 87.63 | -8.50E-01 | 4.77E-06 | 7.41E-04 |
| *PHLDA1* | 175.89 | 112.58 | 107.24 | 44.16 | 45.22 | 50.19 | -1.40E+00 | 4.86E-06 | 7.49E-04 |
| *SPINK13* | 583.27 | 604.00 | 631.54 | 1385.39 | 878.44 | 1251.44 | 9.87E-01 | 4.92E-06 | 7.53E-04 |
| *BCAR3* | 243.77 | 182.96 | 172.72 | 89.97 | 91.87 | 87.63 | -1.01E+00 | 5.19E-06 | 7.65E-04 |
| *MFSD12* | 615.57 | 774.76 | 746.89 | 401.44 | 443.91 | 441.46 | -6.46E-01 | 5.19E-06 | 7.65E-04 |
| *NELL2* | 42.59 | 53.90 | 57.73 | 29.18 | 20.88 | 24.92 | -1.06E+00 | 5.15E-06 | 7.65E-04 |
| *TUBB2A* | 195.27 | 206.77 | 196.72 | 287.60 | 339.09 | 265.10 | 6.96E-01 | 5.17E-06 | 7.65E-04 |
| *VMA21* | 655.52 | 700.55 | 707.73 | 445.52 | 465.13 | 457.63 | -5.07E-01 | 5.11E-06 | 7.65E-04 |
| *TBRG4* | 70.93 | 73.10 | 65.14 | 37.05 | 30.95 | 41.98 | -9.17E-01 | 5.23E-06 | 7.65E-04 |
| *IER2* | 1675.04 | 1911.76 | 1797.70 | 1098.36 | 1284.48 | 1193.99 | -5.56E-01 | 5.28E-06 | 7.65E-04 |
| *SOCS2* | 8.28 | 8.93 | 8.14 | 5.09 | 4.83 | 3.88 | -8.96E-01 | 5.31E-06 | 7.65E-04 |
| *CARS1* | 150.44 | 186.27 | 176.44 | 91.86 | 103.60 | 81.60 | -7.68E-01 | 5.46E-06 | 7.81E-04 |
| *H1-2* | 763.97 | 874.93 | 844.87 | 1386.03 | 1105.28 | 1336.92 | 6.69E-01 | 5.57E-06 | 7.84E-04 |
| *PEX6* | 164.78 | 187.77 | 177.76 | 97.10 | 87.60 | 111.44 | -7.14E-01 | 5.55E-06 | 7.84E-04 |
| *TNFSF10* | 31.77 | 38.38 | 40.77 | 113.92 | 78.98 | 167.46 | 1.67E+00 | 5.59E-06 | 7.84E-04 |
| *CH17-340M24.3* | 145.06 | 136.59 | 131.82 | 204.37 | 201.33 | 188.19 | 6.55E-01 | 6.01E-06 | 8.36E-04 |
| *PROCR* | 104.72 | 105.83 | 101.86 | 156.24 | 146.38 | 143.89 | 6.33E-01 | 6.22E-06 | 8.59E-04 |
| *H2BC9* | 111.22 | 101.39 | 93.01 | 150.21 | 144.99 | 158.47 | 6.96E-01 | 6.88E-06 | 9.45E-04 |
| *FBXL17* | 116.98 | 126.10 | 122.93 | 71.00 | 62.65 | 77.27 | -6.98E-01 | 7.80E-06 | 1.06E-03 |
| *C3orf14* | 40.22 | 53.84 | 46.09 | 26.37 | 27.27 | 22.23 | -9.03E-01 | 8.05E-06 | 1.09E-03 |
| *JUNB* | 224.88 | 257.44 | 233.08 | 129.69 | 156.09 | 140.61 | -6.13E-01 | 8.16E-06 | 1.10E-03 |
| *S100P* | 5817.73 | 6357.08 | 6153.27 | 4773.21 | 3849.32 | 4252.60 | -5.33E-01 | 8.25E-06 | 1.10E-03 |
| *ERRFI1* | 231.02 | 281.33 | 251.88 | 146.09 | 144.83 | 166.22 | -6.06E-01 | 8.43E-06 | 1.12E-03 |
| *LPCAT2* | 21.02 | 16.41 | 19.33 | 10.81 | 10.79 | 11.15 | -8.64E-01 | 8.78E-06 | 1.16E-03 |
| *SELENOP* | 33.78 | 37.76 | 28.31 | 17.57 | 18.03 | 18.93 | -9.24E-01 | 8.85E-06 | 1.16E-03 |
| *ZC3H6* | 63.34 | 74.32 | 70.44 | 40.80 | 43.54 | 40.74 | -7.06E-01 | 8.88E-06 | 1.16E-03 |
| *ID2* | 730.36 | 635.76 | 605.57 | 1249.30 | 912.38 | 1201.66 | 8.30E-01 | 9.29E-06 | 1.20E-03 |
| *C6orf120* | 173.95 | 167.83 | 190.11 | 98.17 | 108.41 | 102.97 | -6.50E-01 | 9.44E-06 | 1.20E-03 |
| *MAMLD1* | 125.00 | 142.56 | 137.94 | 78.57 | 82.91 | 85.60 | -5.96E-01 | 9.47E-06 | 1.20E-03 |
| *PLAAT3* | 1072.70 | 1197.61 | 1118.38 | 798.93 | 777.94 | 766.77 | -4.78E-01 | 9.37E-06 | 1.20E-03 |
| *GPAT3* | 15.27 | 21.66 | 17.67 | 9.03 | 9.62 | 9.48 | -1.01E+00 | 9.60E-06 | 1.21E-03 |
| *CCDC71L* | 50.55 | 47.23 | 47.46 | 22.77 | 20.18 | 30.28 | -1.03E+00 | 1.00E-05 | 1.25E-03 |
| *SCIN* | 105.48 | 97.42 | 96.00 | 42.12 | 25.66 | 52.02 | -1.34E+00 | 1.00E-05 | 1.25E-03 |
| *NPR3* | 91.99 | 89.29 | 92.84 | 133.33 | 136.60 | 123.50 | 6.33E-01 | 1.02E-05 | 1.26E-03 |
| *NRCAM* | 53.41 | 72.00 | 65.00 | 36.03 | 29.10 | 36.25 | -8.94E-01 | 1.02E-05 | 1.26E-03 |
| *LYRM1* | 276.18 | 283.43 | 296.21 | 185.11 | 141.74 | 155.05 | -7.02E-01 | 1.06E-05 | 1.29E-03 |
| *CCN2* | 1088.44 | 1179.39 | 984.60 | 598.40 | 752.35 | 661.38 | -6.30E-01 | 1.08E-05 | 1.31E-03 |
| *TOX2* | 42.90 | 52.83 | 43.02 | 24.89 | 24.10 | 28.67 | -8.67E-01 | 1.09E-05 | 1.32E-03 |
| *DDA1* | 128.24 | 154.37 | 148.02 | 81.63 | 92.52 | 79.90 | -6.42E-01 | 1.19E-05 | 1.42E-03 |
| *PMAIP1* | 712.13 | 683.84 | 646.94 | 439.67 | 463.09 | 378.66 | -5.87E-01 | 1.18E-05 | 1.42E-03 |
| *COL7A1* | 167.74 | 222.30 | 205.06 | 107.63 | 114.27 | 122.82 | -6.54E-01 | 1.20E-05 | 1.42E-03 |
| *OPN3* | 1378.02 | 1558.14 | 1516.25 | 836.10 | 1105.70 | 851.66 | -6.46E-01 | 1.21E-05 | 1.43E-03 |
| *ALPI* | 492.21 | 656.57 | 643.06 | 266.25 | 326.89 | 367.19 | -7.98E-01 | 1.25E-05 | 1.46E-03 |
| *CUL4A* | 504.92 | 637.63 | 601.51 | 362.26 | 373.26 | 364.75 | -5.62E-01 | 1.25E-05 | 1.46E-03 |
| *CTSL* | 651.27 | 772.52 | 716.11 | 442.33 | 502.95 | 441.45 | -5.40E-01 | 1.27E-05 | 1.48E-03 |
| *DTX4* | 144.32 | 187.84 | 130.38 | 78.44 | 83.52 | 83.46 | -7.83E-01 | 1.31E-05 | 1.51E-03 |
| *UTP18* | 1882.01 | 1901.15 | 1736.02 | 1412.75 | 1292.89 | 1260.07 | -4.50E-01 | 1.33E-05 | 1.53E-03 |
| *ASAH1* | 220.45 | 281.27 | 274.44 | 158.84 | 150.75 | 140.46 | -6.45E-01 | 1.35E-05 | 1.54E-03 |
| *MDFIC* | 160.90 | 189.49 | 169.47 | 288.60 | 228.76 | 248.46 | 6.78E-01 | 1.42E-05 | 1.61E-03 |
| *ARHGAP12* | 120.27 | 111.70 | 129.13 | 73.09 | 68.84 | 69.82 | -6.66E-01 | 1.44E-05 | 1.62E-03 |
| *E2F8* | 69.82 | 56.02 | 63.78 | 92.86 | 98.48 | 114.27 | 7.68E-01 | 1.45E-05 | 1.63E-03 |
| *BRI3BP* | 502.53 | 481.87 | 479.55 | 267.04 | 329.34 | 312.29 | -5.75E-01 | 1.50E-05 | 1.66E-03 |
| *LAMB3* | 120.68 | 149.53 | 125.53 | 61.28 | 70.47 | 84.21 | -7.72E-01 | 1.49E-05 | 1.66E-03 |
| *RAP1A* | 187.47 | 163.25 | 169.04 | 83.41 | 98.71 | 103.77 | -7.40E-01 | 1.50E-05 | 1.66E-03 |
| *NBEA* | 82.82 | 79.94 | 67.33 | 41.16 | 43.06 | 46.82 | -7.70E-01 | 1.53E-05 | 1.68E-03 |
| *CAPN2* | 1865.21 | 2161.35 | 2115.34 | 1287.04 | 1559.72 | 1270.77 | -5.64E-01 | 1.58E-05 | 1.71E-03 |
| *PCDHB14* | 34.32 | 27.90 | 26.77 | 14.45 | 15.46 | 18.49 | -9.44E-01 | 1.59E-05 | 1.71E-03 |
| *SPRR4* | 84.22 | 70.83 | 68.49 | 123.26 | 118.41 | 107.54 | 7.42E-01 | 1.59E-05 | 1.71E-03 |
| *MYLK3* | 182.24 | 166.96 | 170.20 | 305.60 | 236.30 | 254.36 | 7.35E-01 | 1.61E-05 | 1.72E-03 |
| *MTCP1* | 28.93 | 36.59 | 29.91 | 19.85 | 19.10 | 16.23 | -8.51E-01 | 1.62E-05 | 1.73E-03 |
| *GAMT* | 103.76 | 87.84 | 107.77 | 143.94 | 157.28 | 143.14 | 6.93E-01 | 1.66E-05 | 1.75E-03 |
| *PJVK* | 37.03 | 55.48 | 51.02 | 19.59 | 17.57 | 26.99 | -1.19E+00 | 1.65E-05 | 1.75E-03 |
| *TUBA1A* | 185.78 | 207.74 | 183.77 | 260.12 | 240.53 | 275.61 | 5.63E-01 | 1.70E-05 | 1.79E-03 |
| *SH3RF3* | 60.46 | 60.64 | 61.28 | 32.72 | 39.89 | 36.27 | -7.37E-01 | 1.72E-05 | 1.79E-03 |
| *AMDHD1* | 17.96 | 22.21 | 21.85 | 13.08 | 11.43 | 11.74 | -8.54E-01 | 1.75E-05 | 1.82E-03 |
| *IGIP* | 118.12 | 139.45 | 150.80 | 69.67 | 75.69 | 86.33 | -7.06E-01 | 1.75E-05 | 1.82E-03 |
| *1-Mar* | 1232.52 | 1476.84 | 1372.40 | 917.02 | 991.77 | 874.42 | -5.02E-01 | 1.84E-05 | 1.89E-03 |
| *LINC00857* | 64.59 | 79.42 | 76.28 | 115.12 | 97.90 | 116.53 | 6.83E-01 | 1.95E-05 | 2.00E-03 |
| *DTL* | 1398.58 | 1513.48 | 1520.76 | 1063.88 | 872.94 | 1030.87 | -5.35E-01 | 2.01E-05 | 2.04E-03 |
| *LYPD8* | 58.31 | 63.59 | 56.10 | 34.76 | 32.04 | 39.75 | -7.42E-01 | 2.03E-05 | 2.04E-03 |
| *SLIT2* | 72.34 | 93.20 | 80.67 | 36.13 | 46.66 | 48.96 | -8.59E-01 | 2.02E-05 | 2.04E-03 |
| *SLC38A1* | 1803.33 | 1949.26 | 1869.69 | 1358.78 | 1387.80 | 1408.01 | -4.18E-01 | 2.08E-05 | 2.09E-03 |
| *CTSC* | 206.93 | 219.50 | 221.50 | 279.49 | 278.78 | 269.95 | 4.88E-01 | 2.09E-05 | 2.09E-03 |
| *EPHX1* | 119.69 | 141.94 | 122.13 | 80.63 | 71.85 | 80.85 | -5.99E-01 | 2.14E-05 | 2.13E-03 |
| *SLC16A9* | 44.68 | 46.52 | 35.11 | 25.90 | 22.43 | 23.81 | -8.40E-01 | 2.21E-05 | 2.18E-03 |
| *TRRAP* | 292.91 | 366.05 | 342.27 | 174.80 | 205.43 | 219.29 | -6.13E-01 | 2.21E-05 | 2.18E-03 |
| *LRRC43* | 175.97 | 119.00 | 124.75 | 242.74 | 243.82 | 222.27 | 9.05E-01 | 2.23E-05 | 2.19E-03 |
| *CRTC3* | 1581.39 | 1831.77 | 1799.86 | 1064.67 | 1254.77 | 1231.22 | -5.14E-01 | 2.25E-05 | 2.19E-03 |
| *ZNF703* | 116.45 | 138.79 | 144.30 | 75.22 | 80.90 | 84.92 | -6.10E-01 | 2.29E-05 | 2.22E-03 |
| *LRP1B* | 5.65 | 4.86 | 4.50 | 3.24 | 2.82 | 2.79 | -7.16E-01 | 2.33E-05 | 2.24E-03 |
| *PHF10* | 320.44 | 393.43 | 393.66 | 219.56 | 242.73 | 232.51 | -5.51E-01 | 2.32E-05 | 2.24E-03 |
| *ZFYVE26* | 129.62 | 156.02 | 145.03 | 86.64 | 89.73 | 94.15 | -5.48E-01 | 2.35E-05 | 2.25E-03 |
| *AJUBA* | 348.80 | 333.39 | 354.24 | 199.67 | 235.61 | 181.32 | -6.26E-01 | 2.40E-05 | 2.27E-03 |
| *IFITM1* | 485.29 | 559.28 | 518.10 | 351.60 | 301.04 | 351.95 | -5.36E-01 | 2.39E-05 | 2.27E-03 |
| *TCIM* | 214.44 | 137.48 | 168.47 | 58.77 | 80.62 | 38.66 | -1.48E+00 | 2.41E-05 | 2.27E-03 |
| *MAGEA11* | 12.06 | 20.77 | 16.78 | 6.01 | 8.18 | 7.81 | -1.20E+00 | 2.47E-05 | 2.32E-03 |
| *ZNF547* | 8.43 | 7.82 | 7.37 | 5.24 | 4.51 | 4.95 | -7.09E-01 | 2.53E-05 | 2.36E-03 |
| *COQ9* | 577.66 | 716.03 | 684.70 | 419.40 | 460.87 | 405.03 | -5.36E-01 | 2.55E-05 | 2.37E-03 |
| *FAM171A1* | 82.60 | 112.79 | 102.40 | 56.54 | 61.53 | 51.00 | -7.33E-01 | 2.65E-05 | 2.43E-03 |
| *PLEKHB2* | 439.50 | 473.19 | 463.07 | 263.20 | 319.87 | 309.45 | -5.13E-01 | 2.64E-05 | 2.43E-03 |
| *RRAGD* | 12.94 | 12.68 | 15.84 | 6.83 | 7.60 | 8.85 | -9.00E-01 | 2.65E-05 | 2.43E-03 |
| *SETD7* | 605.59 | 682.52 | 735.52 | 391.01 | 479.60 | 422.88 | -5.68E-01 | 2.68E-05 | 2.45E-03 |
| *FRMD3* | 35.17 | 38.44 | 31.77 | 20.27 | 22.94 | 21.26 | -7.60E-01 | 2.80E-05 | 2.55E-03 |
| *SLC7A11* | 36.53 | 32.46 | 37.65 | 22.85 | 23.93 | 20.21 | -7.31E-01 | 2.82E-05 | 2.56E-03 |
| *E2F7* | 461.22 | 459.47 | 455.39 | 243.52 | 281.97 | 320.01 | -5.87E-01 | 2.84E-05 | 2.56E-03 |
| *RNF145* | 634.17 | 711.68 | 696.07 | 434.61 | 445.92 | 492.03 | -4.83E-01 | 2.85E-05 | 2.56E-03 |
| *MSRB1* | 220.84 | 273.88 | 241.31 | 321.61 | 342.10 | 302.87 | 5.21E-01 | 2.93E-05 | 2.62E-03 |
| *RBM47* | 87.05 | 101.81 | 96.63 | 50.33 | 64.77 | 51.54 | -7.12E-01 | 3.04E-05 | 2.70E-03 |
| *VASN* | 124.76 | 124.36 | 116.73 | 77.58 | 71.81 | 79.47 | -5.69E-01 | 3.11E-05 | 2.75E-03 |
| *MGME1* | 675.32 | 708.15 | 622.59 | 420.42 | 414.14 | 478.07 | -5.22E-01 | 3.15E-05 | 2.78E-03 |
| *TMEM117* | 123.20 | 160.71 | 170.29 | 76.92 | 90.07 | 89.59 | -6.97E-01 | 3.21E-05 | 2.81E-03 |
| *ADGRG2* | 17.62 | 24.78 | 22.45 | 13.69 | 10.80 | 11.33 | -9.21E-01 | 3.23E-05 | 2.82E-03 |
| *FGF2* | 109.36 | 126.41 | 127.07 | 71.82 | 80.95 | 69.57 | -5.98E-01 | 3.30E-05 | 2.87E-03 |
| *RPN2* | 2264.30 | 2531.93 | 2309.98 | 1703.28 | 1870.48 | 1607.30 | -4.41E-01 | 3.35E-05 | 2.90E-03 |
| *AP1M2* | 155.96 | 158.27 | 129.97 | 219.53 | 246.62 | 190.20 | 6.92E-01 | 3.38E-05 | 2.92E-03 |
| *CLGN* | 70.77 | 64.21 | 66.77 | 45.44 | 35.30 | 40.15 | -7.19E-01 | 3.43E-05 | 2.92E-03 |
| *SARS1* | 660.44 | 721.77 | 733.81 | 506.60 | 506.43 | 464.99 | -4.42E-01 | 3.44E-05 | 2.92E-03 |
| *TCAIM* | 73.83 | 78.45 | 66.68 | 45.64 | 45.40 | 47.06 | -6.20E-01 | 3.42E-05 | 2.92E-03 |
| *SQSTM1* | 961.67 | 926.59 | 935.03 | 650.10 | 710.84 | 625.68 | -4.40E-01 | 3.46E-05 | 2.93E-03 |
| *IFNGR1* | 252.17 | 287.39 | 274.44 | 185.48 | 175.04 | 168.29 | -4.94E-01 | 3.49E-05 | 2.94E-03 |
| *OLFML2A* | 113.12 | 121.61 | 125.07 | 69.15 | 72.11 | 82.54 | -5.83E-01 | 3.61E-05 | 3.03E-03 |
| *COL4A4* | 37.80 | 43.21 | 38.97 | 24.07 | 18.67 | 26.42 | -8.53E-01 | 3.66E-05 | 3.05E-03 |
| *FLJ32255* | 76.36 | 83.66 | 85.05 | 43.44 | 44.06 | 56.84 | -7.25E-01 | 3.66E-05 | 3.05E-03 |
| *E2F5* | 148.59 | 163.11 | 166.54 | 102.23 | 105.29 | 95.29 | -5.29E-01 | 3.70E-05 | 3.07E-03 |
| *SPRY2* | 213.60 | 198.89 | 185.63 | 102.41 | 126.27 | 123.95 | -6.29E-01 | 3.71E-05 | 3.07E-03 |
| *ACSL3* | 632.25 | 665.53 | 731.80 | 441.66 | 458.49 | 471.57 | -4.73E-01 | 3.74E-05 | 3.08E-03 |
| *SMOC1* | 420.80 | 482.44 | 475.07 | 214.54 | 316.69 | 271.77 | -6.79E-01 | 3.76E-05 | 3.08E-03 |
| *ITGA2* | 75.33 | 61.92 | 64.23 | 40.46 | 42.82 | 36.34 | -7.24E-01 | 3.82E-05 | 3.12E-03 |
| *EFNA1* | 248.83 | 287.95 | 253.44 | 159.58 | 181.52 | 143.58 | -5.82E-01 | 3.84E-05 | 3.12E-03 |
| *TBC1D2* | 30.44 | 38.98 | 35.98 | 13.49 | 20.05 | 20.87 | -1.02E+00 | 3.92E-05 | 3.17E-03 |
| *ITPR1* | 711.86 | 675.91 | 738.93 | 513.86 | 461.00 | 474.53 | -4.70E-01 | 3.95E-05 | 3.18E-03 |
| *MLEC* | 889.65 | 1077.78 | 1067.61 | 619.18 | 740.92 | 649.36 | -5.40E-01 | 3.98E-05 | 3.19E-03 |
| *NUS1* | 222.15 | 236.82 | 263.16 | 135.07 | 153.49 | 160.61 | -5.51E-01 | 3.99E-05 | 3.19E-03 |
| *GALNT1* | 351.67 | 378.26 | 385.66 | 252.26 | 250.40 | 219.24 | -5.06E-01 | 4.16E-05 | 3.31E-03 |
| *SLC35D1* | 147.03 | 179.03 | 172.77 | 88.77 | 98.00 | 114.27 | -6.08E-01 | 4.21E-05 | 3.35E-03 |
| *DCN* | 67.54 | 74.97 | 75.27 | 110.32 | 92.61 | 107.06 | 6.05E-01 | 4.50E-05 | 3.52E-03 |
| *PLCL2* | 396.95 | 490.92 | 527.94 | 285.29 | 294.52 | 302.32 | -5.56E-01 | 4.46E-05 | 3.52E-03 |
| *PROSER2* | 564.82 | 598.71 | 578.77 | 401.88 | 417.15 | 412.23 | -4.08E-01 | 4.47E-05 | 3.52E-03 |
| *TRIM2* | 126.60 | 163.18 | 160.19 | 85.04 | 88.77 | 98.46 | -5.94E-01 | 4.50E-05 | 3.52E-03 |
| *ACER3* | 51.41 | 58.13 | 57.13 | 32.70 | 34.13 | 38.67 | -6.67E-01 | 4.57E-05 | 3.54E-03 |
| *SLC22A15* | 69.06 | 90.08 | 78.49 | 49.96 | 46.97 | 49.48 | -6.29E-01 | 4.57E-05 | 3.54E-03 |
| *SLC30A7* | 189.32 | 176.43 | 176.14 | 92.76 | 111.44 | 118.59 | -6.24E-01 | 4.59E-05 | 3.54E-03 |
| *SLC3A2* | 738.27 | 712.85 | 645.77 | 456.94 | 500.78 | 467.60 | -4.63E-01 | 4.69E-05 | 3.61E-03 |
| *MAP2K6* | 218.73 | 283.40 | 278.86 | 163.90 | 162.40 | 147.84 | -5.78E-01 | 4.72E-05 | 3.61E-03 |
| *UXS1* | 169.90 | 191.19 | 179.11 | 109.39 | 121.00 | 121.25 | -4.84E-01 | 4.72E-05 | 3.61E-03 |
| *CCND1* | 100.59 | 99.86 | 94.16 | 54.22 | 68.05 | 49.77 | -7.10E-01 | 4.78E-05 | 3.64E-03 |
| *CYP20A1* | 47.25 | 48.77 | 50.64 | 31.15 | 33.26 | 32.06 | -6.17E-01 | 4.85E-05 | 3.66E-03 |
| *PCM1* | 599.24 | 598.28 | 611.20 | 385.08 | 432.74 | 429.75 | -4.42E-01 | 4.86E-05 | 3.66E-03 |
| *PPARG* | 147.08 | 194.27 | 200.57 | 107.77 | 110.21 | 94.10 | -6.62E-01 | 4.85E-05 | 3.66E-03 |
| *PRR34-AS1* | 608.41 | 629.48 | 628.00 | 812.09 | 781.97 | 749.24 | 4.01E-01 | 4.92E-05 | 3.69E-03 |
| *GRN* | 532.39 | 605.60 | 586.93 | 366.80 | 369.24 | 423.68 | -4.76E-01 | 4.97E-05 | 3.71E-03 |
| *FOXE1* | 35.10 | 42.33 | 38.23 | 22.04 | 21.66 | 26.58 | -7.66E-01 | 5.07E-05 | 3.77E-03 |
| *RAB31* | 381.76 | 466.34 | 427.86 | 263.65 | 265.87 | 302.34 | -4.92E-01 | 5.09E-05 | 3.77E-03 |
| *PXDN* | 1151.23 | 1363.76 | 1239.42 | 832.01 | 955.70 | 846.12 | -4.63E-01 | 5.12E-05 | 3.78E-03 |
| *NLRC5* | 45.39 | 53.28 | 69.28 | 30.07 | 30.48 | 32.37 | -8.42E-01 | 5.15E-05 | 3.80E-03 |
| *ANKRD50* | 248.29 | 268.61 | 244.25 | 160.26 | 137.10 | 175.34 | -5.63E-01 | 5.18E-05 | 3.80E-03 |
| *FAAH2* | 76.33 | 87.26 | 86.38 | 53.41 | 56.61 | 50.67 | -5.71E-01 | 5.23E-05 | 3.81E-03 |
| *LARP6* | 91.47 | 102.60 | 104.24 | 60.12 | 63.28 | 67.95 | -5.56E-01 | 5.25E-05 | 3.81E-03 |
| *LINC00323* | 12.36 | 18.97 | 16.11 | 8.49 | 7.38 | 9.19 | -9.68E-01 | 5.24E-05 | 3.81E-03 |
| *NIBAN2* | 285.81 | 323.81 | 335.17 | 195.29 | 222.24 | 194.46 | -4.92E-01 | 5.28E-05 | 3.82E-03 |
| *LGSN* | 95.83 | 87.08 | 90.49 | 56.62 | 60.69 | 55.37 | -5.93E-01 | 5.41E-05 | 3.90E-03 |
| *FXYD5* | 604.00 | 677.66 | 687.43 | 403.31 | 495.74 | 410.49 | -5.04E-01 | 5.52E-05 | 3.95E-03 |
| *ZNF598* | 174.27 | 220.04 | 213.67 | 118.29 | 134.19 | 128.99 | -5.33E-01 | 5.50E-05 | 3.95E-03 |
| *CTNNAL1* | 910.63 | 912.58 | 883.11 | 1177.35 | 1099.10 | 1143.43 | 3.88E-01 | 5.57E-05 | 3.96E-03 |
| *DLGAP1-AS2* | 25.98 | 23.25 | 24.94 | 18.11 | 15.48 | 15.82 | -6.75E-01 | 5.56E-05 | 3.96E-03 |
| *INSIG1* | 150.64 | 142.92 | 136.44 | 183.20 | 188.88 | 186.62 | 5.06E-01 | 5.73E-05 | 4.04E-03 |
| *LRRC57* | 139.67 | 136.59 | 145.39 | 74.96 | 92.61 | 91.48 | -5.93E-01 | 5.72E-05 | 4.04E-03 |
| *MYL6B* | 324.70 | 432.84 | 391.36 | 520.43 | 498.96 | 496.27 | 5.09E-01 | 5.91E-05 | 4.15E-03 |
| *SLC16A6* | 304.12 | 401.38 | 387.20 | 240.78 | 205.40 | 212.93 | -6.01E-01 | 5.99E-05 | 4.20E-03 |
| *SLC7A5* | 2467.81 | 2582.22 | 2462.94 | 1755.92 | 2051.16 | 1903.74 | -4.05E-01 | 6.01E-05 | 4.20E-03 |
| *DUSP5* | 83.07 | 99.20 | 103.60 | 65.36 | 51.74 | 53.70 | -6.67E-01 | 6.36E-05 | 4.40E-03 |
| *OTUD1* | 218.90 | 250.39 | 242.44 | 138.19 | 159.57 | 166.09 | -4.89E-01 | 6.37E-05 | 4.40E-03 |
| *PIMREG* | 329.78 | 348.07 | 321.22 | 396.44 | 426.35 | 415.98 | 4.27E-01 | 6.35E-05 | 4.40E-03 |
| *CHN1* | 285.94 | 326.41 | 307.30 | 376.77 | 425.10 | 361.52 | 4.65E-01 | 6.45E-05 | 4.44E-03 |
| *IL17RD* | 34.75 | 31.81 | 37.64 | 19.94 | 18.23 | 24.76 | -8.02E-01 | 6.59E-05 | 4.52E-03 |
| *PITPNA* | 346.27 | 386.77 | 369.71 | 249.93 | 251.55 | 255.33 | -4.14E-01 | 6.66E-05 | 4.56E-03 |
| *RIMKLB* | 593.09 | 633.04 | 662.92 | 968.79 | 792.39 | 798.48 | 5.01E-01 | 6.82E-05 | 4.65E-03 |
| *DAAM2* | 36.77 | 39.44 | 36.94 | 63.99 | 55.77 | 54.26 | 6.23E-01 | 6.94E-05 | 4.71E-03 |
| *PLAC8* | 1295.90 | 1507.03 | 1491.19 | 2123.77 | 1929.19 | 1717.59 | 4.52E-01 | 6.99E-05 | 4.73E-03 |
| *TESC* | 74.10 | 88.57 | 85.84 | 41.23 | 58.42 | 41.95 | -7.71E-01 | 7.07E-05 | 4.77E-03 |
| *HSD17B7* | 302.25 | 320.54 | 338.77 | 367.33 | 438.29 | 426.47 | 4.78E-01 | 7.10E-05 | 4.78E-03 |
| *ZNF615* | 26.65 | 20.54 | 23.56 | 45.04 | 36.66 | 40.46 | 7.24E-01 | 7.18E-05 | 4.81E-03 |
| *ADARB1* | 321.64 | 386.80 | 352.34 | 198.41 | 242.96 | 242.59 | -5.13E-01 | 7.25E-05 | 4.83E-03 |
| *SLC25A36* | 1112.45 | 1344.27 | 1297.00 | 859.07 | 801.44 | 914.55 | -4.86E-01 | 7.24E-05 | 4.83E-03 |
| *LOC102546294* | 53.21 | 61.95 | 59.89 | 100.23 | 78.10 | 85.31 | 6.56E-01 | 7.29E-05 | 4.83E-03 |
| *TPBG* | 495.73 | 628.58 | 607.60 | 339.23 | 381.74 | 403.12 | -5.24E-01 | 7.33E-05 | 4.85E-03 |
| *RNF217* | 146.35 | 145.10 | 149.51 | 98.62 | 92.33 | 94.49 | -4.98E-01 | 7.36E-05 | 4.85E-03 |
| *VWA5A* | 86.22 | 114.02 | 112.94 | 68.10 | 61.76 | 57.15 | -6.47E-01 | 7.61E-05 | 5.00E-03 |
| *DANCR* | 1079.99 | 1172.49 | 1085.79 | 1561.31 | 1427.31 | 1316.61 | 3.97E-01 | 7.75E-05 | 5.08E-03 |
| *EVA1C* | 214.60 | 288.62 | 275.34 | 157.44 | 143.21 | 170.44 | -5.80E-01 | 8.20E-05 | 5.35E-03 |
| *KDM7A* | 53.00 | 65.01 | 65.86 | 34.09 | 36.41 | 42.56 | -6.93E-01 | 8.33E-05 | 5.40E-03 |
| *OGFRP1* | 58.53 | 43.35 | 46.99 | 85.06 | 77.79 | 71.65 | 7.15E-01 | 8.31E-05 | 5.40E-03 |
| *SUPT7L* | 78.47 | 88.94 | 89.53 | 55.87 | 56.62 | 57.77 | -5.23E-01 | 8.61E-05 | 5.55E-03 |
| *TSSK3* | 86.34 | 62.39 | 62.30 | 111.24 | 107.36 | 104.28 | 7.18E-01 | 8.58E-05 | 5.55E-03 |
| *CDKN1A* | 266.97 | 338.60 | 280.52 | 175.09 | 176.44 | 206.14 | -5.29E-01 | 8.67E-05 | 5.55E-03 |
| *CLDN1* | 16.88 | 11.01 | 12.92 | 7.80 | 8.63 | 7.86 | -7.98E-01 | 8.65E-05 | 5.55E-03 |
| *FOS* | 346.47 | 417.57 | 399.32 | 183.98 | 280.50 | 200.45 | -7.04E-01 | 8.75E-05 | 5.57E-03 |
| *MGST3* | 1991.51 | 2137.94 | 2091.40 | 1730.24 | 1464.02 | 1476.49 | -4.02E-01 | 8.78E-05 | 5.57E-03 |
| *OGFRL1* | 100.87 | 92.39 | 83.69 | 115.32 | 134.33 | 130.44 | 5.72E-01 | 8.79E-05 | 5.57E-03 |
| *LINC00319* | 62.52 | 73.84 | 68.10 | 35.38 | 41.77 | 47.81 | -6.98E-01 | 8.88E-05 | 5.60E-03 |
| *PTGES* | 126.10 | 130.44 | 123.02 | 77.78 | 78.12 | 90.18 | -5.12E-01 | 8.87E-05 | 5.60E-03 |
| *NEK9* | 412.36 | 523.42 | 539.23 | 311.89 | 305.85 | 329.77 | -5.13E-01 | 8.98E-05 | 5.64E-03 |
| *CXCL16* | 89.97 | 95.28 | 85.50 | 60.20 | 61.21 | 58.64 | -5.10E-01 | 9.16E-05 | 5.74E-03 |
| *CSRP1* | 870.78 | 940.04 | 877.20 | 597.57 | 710.90 | 559.88 | -4.61E-01 | 9.25E-05 | 5.78E-03 |
| *ZIC2* | 320.44 | 384.37 | 374.71 | 236.71 | 223.95 | 256.00 | -4.69E-01 | 9.31E-05 | 5.80E-03 |
| *ZNF30* | 74.94 | 95.06 | 83.17 | 56.05 | 55.10 | 52.45 | -5.53E-01 | 9.86E-05 | 6.12E-03 |
| *MPC2* | 788.84 | 815.98 | 786.94 | 1031.06 | 954.66 | 985.91 | 3.71E-01 | 9.96E-05 | 6.15E-03 |
| *PSMB9* | 40.49 | 42.51 | 49.36 | 29.57 | 30.02 | 22.45 | -7.21E-01 | 9.96E-05 | 6.15E-03 |
| *HOTAIR* | 42.49 | 54.07 | 47.29 | 27.98 | 25.13 | 33.81 | -7.49E-01 | 1.02E-04 | 6.29E-03 |
| *MAGED1* | 100.45 | 98.98 | 84.73 | 66.24 | 51.56 | 51.29 | -6.75E-01 | 1.04E-04 | 6.38E-03 |
| *GNG4* | 69.64 | 69.10 | 72.01 | 91.27 | 103.83 | 91.38 | 5.25E-01 | 1.05E-04 | 6.39E-03 |
| *SHMT2* | 227.66 | 257.58 | 263.59 | 140.54 | 174.02 | 169.69 | -4.99E-01 | 1.05E-04 | 6.41E-03 |
| *SORBS1* | 226.79 | 191.75 | 205.35 | 249.89 | 286.92 | 301.44 | 5.63E-01 | 1.05E-04 | 6.41E-03 |
| *ATP6V1G3* | 22.77 | 25.85 | 23.65 | 17.61 | 13.94 | 9.63 | -9.20E-01 | 1.07E-04 | 6.46E-03 |
| *RNF38* | 149.89 | 168.51 | 119.55 | 73.82 | 79.93 | 94.66 | -6.98E-01 | 1.07E-04 | 6.49E-03 |
| *ATF4* | 2290.66 | 2307.76 | 2364.19 | 1612.49 | 1968.03 | 1653.42 | -4.17E-01 | 1.09E-04 | 6.57E-03 |
| *TMEM47* | 78.66 | 86.40 | 83.42 | 102.19 | 105.03 | 122.23 | 5.11E-01 | 1.10E-04 | 6.61E-03 |
| *DDIT4* | 1473.40 | 1619.80 | 1601.25 | 1053.21 | 1219.67 | 1195.27 | -4.02E-01 | 1.15E-04 | 6.87E-03 |
| *LOC102724880* | 28.15 | 30.68 | 35.90 | 21.89 | 20.44 | 20.44 | -6.57E-01 | 1.16E-04 | 6.90E-03 |
| *FSTL3* | 253.02 | 306.49 | 294.50 | 158.29 | 206.86 | 177.39 | -5.24E-01 | 1.16E-04 | 6.92E-03 |
| *LINC00205* | 30.23 | 38.20 | 33.47 | 17.11 | 21.71 | 22.90 | -7.83E-01 | 1.17E-04 | 6.92E-03 |
| *LBR* | 1204.50 | 856.22 | 806.79 | 524.72 | 561.85 | 564.12 | -7.02E-01 | 1.17E-04 | 6.93E-03 |
| *GALNT12* | 55.44 | 36.78 | 36.77 | 22.03 | 15.44 | 25.97 | -1.08E+00 | 1.20E-04 | 7.10E-03 |
| *MSRB2* | 624.27 | 782.83 | 775.26 | 459.48 | 453.45 | 525.29 | -5.15E-01 | 1.21E-04 | 7.11E-03 |
| *SETD5* | 65.07 | 42.92 | 49.78 | 92.57 | 84.27 | 77.67 | 7.64E-01 | 1.22E-04 | 7.13E-03 |
| *TAGAP* | 84.71 | 57.16 | 64.60 | 109.18 | 104.91 | 101.60 | 7.14E-01 | 1.22E-04 | 7.13E-03 |
| *CUTC* | 326.46 | 341.37 | 342.71 | 432.44 | 412.70 | 391.34 | 4.03E-01 | 1.23E-04 | 7.18E-03 |
| *OXER1* | 117.85 | 94.38 | 104.27 | 145.44 | 158.83 | 136.02 | 6.01E-01 | 1.23E-04 | 7.18E-03 |
| *ERMP1* | 535.64 | 584.95 | 610.72 | 393.06 | 409.98 | 425.58 | -4.00E-01 | 1.25E-04 | 7.24E-03 |
| *CD55* | 6585.41 | 6869.44 | 6918.12 | 5563.27 | 5424.56 | 5773.13 | -3.02E-01 | 1.26E-04 | 7.25E-03 |
| *STEAP1* | 55.63 | 70.08 | 68.24 | 41.17 | 37.73 | 44.81 | -6.22E-01 | 1.26E-04 | 7.25E-03 |
| *C1QTNF6* | 100.70 | 107.94 | 106.81 | 62.43 | 74.16 | 70.27 | -5.15E-01 | 1.29E-04 | 7.42E-03 |
| *AKR1C2* | 1079.76 | 1357.63 | 1325.97 | 920.94 | 806.94 | 809.48 | -5.03E-01 | 1.30E-04 | 7.47E-03 |
| *COL3A1* | 326.86 | 425.40 | 423.07 | 211.60 | 235.46 | 274.77 | -5.80E-01 | 1.31E-04 | 7.48E-03 |
| *PIH1D2* | 11.56 | 11.64 | 14.07 | 8.60 | 7.58 | 8.42 | -6.66E-01 | 1.31E-04 | 7.48E-03 |
| *BAG1* | 663.62 | 732.00 | 720.11 | 833.46 | 885.52 | 894.57 | 3.66E-01 | 1.32E-04 | 7.48E-03 |
| *ACAT2* | 337.89 | 321.94 | 307.83 | 408.57 | 474.09 | 381.94 | 4.98E-01 | 1.33E-04 | 7.51E-03 |
| *TTC30A* | 8.36 | 9.21 | 9.18 | 5.16 | 5.88 | 6.46 | -6.67E-01 | 1.33E-04 | 7.51E-03 |
| *TRIP11* | 57.94 | 59.60 | 62.60 | 36.49 | 42.94 | 40.89 | -5.75E-01 | 1.34E-04 | 7.54E-03 |
| *ST6GAL1* | 45.74 | 44.36 | 48.28 | 62.82 | 64.04 | 69.77 | 5.32E-01 | 1.35E-04 | 7.56E-03 |
| *ITGB1* | 1353.79 | 1408.89 | 1289.08 | 1022.29 | 1073.93 | 1012.02 | -3.44E-01 | 1.35E-04 | 7.56E-03 |
| *L3MBTL3* | 72.58 | 72.92 | 75.96 | 50.52 | 38.74 | 50.30 | -6.32E-01 | 1.35E-04 | 7.56E-03 |
| *HYOU1* | 380.56 | 399.44 | 414.51 | 254.10 | 301.21 | 245.28 | -4.68E-01 | 1.37E-04 | 7.59E-03 |
| *LINC02274* | 11.84 | 16.58 | 10.83 | 8.20 | 7.90 | 5.90 | -8.88E-01 | 1.37E-04 | 7.59E-03 |
| *SCG5* | 20.73 | 33.32 | 33.06 | 17.05 | 14.60 | 15.53 | -9.13E-01 | 1.37E-04 | 7.59E-03 |
| *COX7B* | 4674.35 | 4469.50 | 4511.38 | 6119.30 | 5804.54 | 5291.31 | 3.16E-01 | 1.38E-04 | 7.62E-03 |
| *UBQLN4* | 87.31 | 80.35 | 82.70 | 103.65 | 107.96 | 118.64 | 5.02E-01 | 1.38E-04 | 7.62E-03 |
| *AARS1* | 839.28 | 955.68 | 980.68 | 638.32 | 711.98 | 647.08 | -4.09E-01 | 1.40E-04 | 7.69E-03 |
| *ARFGEF3* | 14.76 | 24.86 | 20.08 | 11.01 | 10.30 | 11.28 | -9.12E-01 | 1.41E-04 | 7.71E-03 |
| *LOC102606465* | 119.64 | 120.61 | 132.60 | 85.62 | 79.09 | 81.75 | -4.84E-01 | 1.41E-04 | 7.73E-03 |
| *PCDH7* | 61.23 | 69.26 | 55.16 | 28.56 | 37.92 | 41.94 | -7.82E-01 | 1.42E-04 | 7.74E-03 |
| *IL7* | 3.95 | 4.19 | 4.12 | 2.79 | 2.98 | 2.61 | -5.15E-01 | 1.45E-04 | 7.87E-03 |
| *ANKLE2* | 131.31 | 127.11 | 117.95 | 81.44 | 83.29 | 83.86 | -4.87E-01 | 1.45E-04 | 7.88E-03 |
| *RBM19* | 39.67 | 36.44 | 36.74 | 26.77 | 27.48 | 23.30 | -5.93E-01 | 1.47E-04 | 7.95E-03 |
| *MT2A* | 3590.99 | 3702.28 | 3568.18 | 2814.27 | 3184.99 | 2942.78 | -3.33E-01 | 1.50E-04 | 8.05E-03 |
| *TAGLN2* | 882.19 | 902.77 | 817.47 | 1023.78 | 1142.76 | 1061.44 | 3.66E-01 | 1.49E-04 | 8.05E-03 |
| *SLC44A2* | 74.85 | 73.79 | 64.05 | 88.51 | 100.15 | 100.14 | 5.27E-01 | 1.51E-04 | 8.10E-03 |
| *AOC3* | 17.19 | 17.49 | 14.53 | 9.68 | 11.79 | 10.91 | -6.88E-01 | 1.57E-04 | 8.36E-03 |
| *MCM2* | 1342.51 | 1476.27 | 1292.48 | 981.05 | 956.27 | 1090.15 | -4.01E-01 | 1.56E-04 | 8.36E-03 |
| *C3P1* | 25.20 | 25.40 | 26.94 | 38.40 | 40.77 | 40.31 | 5.64E-01 | 1.58E-04 | 8.39E-03 |
| *PXN* | 160.26 | 199.53 | 175.52 | 117.80 | 119.23 | 120.96 | -4.40E-01 | 1.58E-04 | 8.39E-03 |
| *TOGARAM2* | 26.16 | 34.96 | 34.02 | 19.79 | 22.24 | 15.88 | -7.79E-01 | 1.58E-04 | 8.39E-03 |
| *RASSF9* | 106.86 | 132.60 | 126.44 | 166.18 | 146.38 | 153.05 | 4.84E-01 | 1.60E-04 | 8.45E-03 |
| *PAX8* | 122.78 | 102.00 | 99.77 | 159.57 | 148.52 | 138.48 | 5.83E-01 | 1.60E-04 | 8.45E-03 |
| *GUSB* | 721.53 | 885.09 | 842.26 | 541.84 | 543.67 | 614.62 | -4.51E-01 | 1.63E-04 | 8.54E-03 |
| *RNF144B* | 149.66 | 164.60 | 160.00 | 107.12 | 111.34 | 103.89 | -4.27E-01 | 1.63E-04 | 8.54E-03 |
| *ZNF225* | 16.88 | 12.11 | 18.80 | 8.62 | 9.72 | 10.26 | -8.02E-01 | 1.65E-04 | 8.64E-03 |
| *H2BC6* | 94.60 | 87.12 | 88.15 | 137.22 | 110.98 | 121.10 | 5.61E-01 | 1.68E-04 | 8.66E-03 |
| *KLHDC8B* | 152.19 | 169.68 | 158.03 | 183.44 | 214.84 | 193.32 | 4.33E-01 | 1.66E-04 | 8.66E-03 |
| *L1CAM* | 1287.86 | 1354.74 | 1290.39 | 849.24 | 955.03 | 1051.42 | -4.17E-01 | 1.68E-04 | 8.66E-03 |
| *STEAP4* | 40.96 | 44.00 | 38.57 | 21.87 | 31.77 | 19.94 | -8.14E-01 | 1.67E-04 | 8.66E-03 |
| *ZNF385D* | 10.81 | 12.15 | 14.36 | 7.08 | 8.58 | 8.03 | -7.18E-01 | 1.67E-04 | 8.66E-03 |
| *FA2H* | 72.64 | 79.60 | 88.18 | 37.69 | 46.38 | 57.07 | -7.43E-01 | 1.72E-04 | 8.85E-03 |
| *CFAP44* | 12.45 | 13.43 | 14.45 | 7.64 | 8.69 | 9.81 | -7.01E-01 | 1.74E-04 | 8.89E-03 |
| *SENP5* | 223.62 | 254.23 | 261.94 | 169.10 | 170.03 | 165.48 | -4.17E-01 | 1.73E-04 | 8.89E-03 |
| *SNX9* | 126.21 | 132.76 | 123.40 | 82.18 | 88.91 | 87.70 | -4.47E-01 | 1.74E-04 | 8.89E-03 |
| *IPMK* | 35.96 | 29.38 | 26.02 | 21.16 | 18.69 | 19.33 | -6.92E-01 | 1.75E-04 | 8.91E-03 |
| *PSMB10* | 106.47 | 126.38 | 112.79 | 148.37 | 182.11 | 137.06 | 5.58E-01 | 1.76E-04 | 8.91E-03 |
| *SLC7A8* | 96.79 | 76.62 | 79.45 | 121.42 | 116.82 | 110.54 | 5.79E-01 | 1.76E-04 | 8.91E-03 |
| *NLK* | 266.44 | 284.94 | 263.88 | 300.52 | 365.41 | 378.52 | 4.69E-01 | 1.79E-04 | 9.00E-03 |
| *ORC5* | 358.10 | 417.97 | 391.58 | 271.01 | 280.92 | 244.55 | -4.29E-01 | 1.79E-04 | 9.00E-03 |
| *TFAP2C* | 144.02 | 176.10 | 156.50 | 186.13 | 191.20 | 211.00 | 4.39E-01 | 1.79E-04 | 9.00E-03 |
| *SLC9A6* | 304.20 | 353.77 | 333.82 | 216.99 | 240.88 | 231.75 | -3.95E-01 | 1.80E-04 | 9.02E-03 |
| *PCK2* | 226.17 | 286.09 | 266.85 | 174.87 | 172.58 | 176.47 | -4.35E-01 | 1.80E-04 | 9.03E-03 |
| *KCNN4* | 210.63 | 273.95 | 272.88 | 154.80 | 172.11 | 157.92 | -4.99E-01 | 1.81E-04 | 9.04E-03 |
| *TIMP3* | 326.44 | 357.46 | 358.80 | 403.44 | 441.68 | 528.73 | 5.04E-01 | 1.83E-04 | 9.10E-03 |
| *TLL1* | 31.43 | 41.71 | 37.03 | 25.47 | 24.46 | 23.00 | -6.36E-01 | 1.83E-04 | 9.11E-03 |
| *BAIAP2-DT* | 101.86 | 112.27 | 101.31 | 141.10 | 127.92 | 129.10 | 4.57E-01 | 1.86E-04 | 9.24E-03 |
| *BFSP1* | 22.91 | 31.42 | 30.41 | 19.73 | 16.00 | 17.51 | -7.30E-01 | 1.87E-04 | 9.24E-03 |
| *THAP2* | 25.77 | 30.31 | 23.62 | 16.63 | 11.65 | 18.34 | -8.58E-01 | 1.87E-04 | 9.24E-03 |
| *PACS2* | 456.77 | 293.53 | 329.59 | 599.27 | 542.83 | 514.60 | 7.51E-01 | 1.93E-04 | 9.51E-03 |
| *2-Mar* | 170.96 | 216.93 | 197.06 | 128.62 | 129.44 | 134.06 | -4.35E-01 | 1.97E-04 | 9.61E-03 |
| *ABCC4* | 53.44 | 61.77 | 49.18 | 31.73 | 34.04 | 39.94 | -6.42E-01 | 1.96E-04 | 9.61E-03 |
| *DDAH2* | 124.08 | 132.51 | 139.80 | 160.89 | 164.38 | 157.55 | 4.21E-01 | 1.97E-04 | 9.61E-03 |
| *EPB41L4B* | 249.23 | 315.66 | 295.10 | 184.44 | 194.64 | 199.39 | -4.29E-01 | 1.98E-04 | 9.61E-03 |
| *EXT1* | 152.74 | 206.14 | 190.74 | 104.23 | 126.66 | 116.51 | -5.29E-01 | 1.98E-04 | 9.61E-03 |
| *UFC1* | 775.68 | 819.87 | 807.71 | 1036.60 | 971.93 | 927.97 | 3.49E-01 | 1.99E-04 | 9.67E-03 |
| *DR1* | 74.07 | 84.41 | 79.59 | 38.41 | 54.26 | 52.26 | -6.78E-01 | 2.00E-04 | 9.68E-03 |
| *MYO5C* | 674.01 | 826.60 | 844.46 | 556.32 | 510.18 | 557.83 | -4.48E-01 | 2.01E-04 | 9.68E-03 |
| *ARHGEF4* | 90.05 | 119.90 | 107.47 | 64.46 | 66.45 | 73.97 | -5.34E-01 | 2.02E-04 | 9.71E-03 |
| *ARIH1* | 48.66 | 59.37 | 55.86 | 37.23 | 35.64 | 38.85 | -5.49E-01 | 2.03E-04 | 9.71E-03 |
| *DUBR* | 27.94 | 36.27 | 38.16 | 19.40 | 22.70 | 22.40 | -7.18E-01 | 2.03E-04 | 9.71E-03 |
| *PRKACB* | 454.63 | 518.88 | 499.12 | 328.73 | 322.97 | 375.78 | -4.09E-01 | 2.03E-04 | 9.71E-03 |
| *LZTFL1* | 146.25 | 164.74 | 128.62 | 98.45 | 77.65 | 93.83 | -5.78E-01 | 2.06E-04 | 9.81E-03 |
| *RPUSD3* | 189.30 | 197.54 | 224.11 | 241.52 | 266.01 | 249.46 | 4.41E-01 | 2.07E-04 | 9.84E-03 |
| *WFDC21P* | 553.28 | 633.10 | 617.26 | 461.99 | 413.75 | 382.68 | -4.31E-01 | 2.07E-04 | 9.84E-03 |
| *CHST2* | 38.92 | 49.01 | 45.60 | 32.30 | 22.74 | 28.43 | -7.07E-01 | 2.08E-04 | 9.84E-03 |
| *HRAS* | 229.60 | 268.26 | 262.91 | 293.94 | 350.70 | 300.15 | 4.35E-01 | 2.09E-04 | 9.87E-03 |
| *CCNE2* | 500.64 | 384.53 | 333.58 | 700.13 | 534.96 | 647.18 | 7.34E-01 | 2.11E-04 | 9.90E-03 |
| *CIB2* | 50.00 | 50.15 | 48.69 | 69.24 | 64.28 | 70.72 | 4.90E-01 | 2.11E-04 | 9.90E-03 |
| *NPC1* | 348.90 | 507.66 | 465.90 | 244.65 | 292.37 | 276.71 | -5.65E-01 | 2.15E-04 | 1.01E-02 |
| *COTL1* | 41.94 | 47.60 | 47.94 | 31.15 | 32.53 | 31.83 | -5.39E-01 | 2.16E-04 | 1.01E-02 |
| *NCOR2* | 636.95 | 725.03 | 736.07 | 437.57 | 510.32 | 522.83 | -4.30E-01 | 2.16E-04 | 1.01E-02 |
| *ACTA2* | 70.13 | 73.06 | 66.92 | 44.74 | 47.73 | 51.27 | -5.09E-01 | 2.18E-04 | 1.01E-02 |
| *ALDH2* | 304.01 | 379.24 | 371.81 | 236.38 | 234.92 | 247.16 | -4.29E-01 | 2.18E-04 | 1.01E-02 |
| *SGPP1* | 310.42 | 268.40 | 244.57 | 172.52 | 166.24 | 180.91 | -5.24E-01 | 2.18E-04 | 1.01E-02 |
| *ALPP* | 2634.20 | 2671.23 | 2781.14 | 1552.31 | 2219.90 | 1917.86 | -5.42E-01 | 2.21E-04 | 1.02E-02 |
| *PURA* | 418.26 | 458.19 | 464.60 | 297.71 | 337.33 | 314.90 | -3.81E-01 | 2.22E-04 | 1.02E-02 |
| *MFAP3* | 52.27 | 59.38 | 58.60 | 42.50 | 38.12 | 36.65 | -5.26E-01 | 2.24E-04 | 1.03E-02 |
| *TSPAN12* | 229.26 | 127.06 | 132.36 | 295.59 | 256.21 | 273.17 | 9.42E-01 | 2.24E-04 | 1.03E-02 |
| *ZNF622* | 672.11 | 737.58 | 706.26 | 533.03 | 545.38 | 511.36 | -3.42E-01 | 2.25E-04 | 1.03E-02 |
| *CHAC2* | 609.72 | 421.44 | 322.81 | 773.71 | 738.82 | 728.74 | 8.70E-01 | 2.27E-04 | 1.04E-02 |
| *ATG14* | 199.51 | 236.60 | 237.33 | 160.67 | 137.14 | 147.48 | -4.63E-01 | 2.27E-04 | 1.04E-02 |
| *CEP55* | 640.28 | 560.75 | 555.72 | 764.44 | 759.88 | 705.02 | 4.22E-01 | 2.28E-04 | 1.04E-02 |
| *RBM3* | 661.15 | 422.44 | 417.42 | 774.54 | 791.51 | 768.13 | 7.58E-01 | 2.30E-04 | 1.04E-02 |
| *USH1G* | 93.10 | 64.52 | 76.27 | 116.54 | 115.34 | 106.32 | 6.47E-01 | 2.30E-04 | 1.04E-02 |
| *SLC25A35* | 131.26 | 130.46 | 120.93 | 167.73 | 149.62 | 159.68 | 4.52E-01 | 2.33E-04 | 1.05E-02 |
| *AQP3* | 364.84 | 390.25 | 344.76 | 455.60 | 429.31 | 445.60 | 3.86E-01 | 2.34E-04 | 1.06E-02 |
| *HOMER1* | 72.27 | 84.62 | 85.02 | 46.47 | 56.21 | 55.93 | -5.53E-01 | 2.36E-04 | 1.06E-02 |
| *ETNPPL* | 126.81 | 147.48 | 138.56 | 95.71 | 94.75 | 93.07 | -4.17E-01 | 2.37E-04 | 1.06E-02 |
| *NQO1* | 771.80 | 797.87 | 739.72 | 606.91 | 559.50 | 500.11 | -3.93E-01 | 2.37E-04 | 1.06E-02 |
| *IFNE* | 8.23 | 10.43 | 9.54 | 6.59 | 6.05 | 6.38 | -6.27E-01 | 2.39E-04 | 1.07E-02 |
| *KNSTRN* | 312.44 | 312.05 | 260.94 | 408.71 | 386.31 | 344.94 | 4.86E-01 | 2.39E-04 | 1.07E-02 |
| *LOC100996506* | 67.82 | 47.19 | 55.55 | 91.39 | 82.80 | 79.57 | 6.51E-01 | 2.42E-04 | 1.07E-02 |
| *MYOF* | 1266.29 | 1440.05 | 1444.57 | 929.55 | 1046.29 | 1100.76 | -4.01E-01 | 2.42E-04 | 1.07E-02 |
| *RAMAC* | 139.33 | 164.37 | 165.44 | 107.77 | 106.18 | 92.10 | -4.83E-01 | 2.42E-04 | 1.07E-02 |
| *APIP* | 351.02 | 360.16 | 347.12 | 539.60 | 397.70 | 461.02 | 5.03E-01 | 2.44E-04 | 1.08E-02 |
| *CA11* | 57.49 | 60.80 | 44.27 | 79.60 | 72.74 | 82.11 | 5.95E-01 | 2.45E-04 | 1.08E-02 |
| *NTAN1* | 167.46 | 175.62 | 158.90 | 204.86 | 201.59 | 196.45 | 4.03E-01 | 2.46E-04 | 1.08E-02 |
| *GAS1RR* | 17.69 | 19.30 | 21.19 | 13.84 | 11.65 | 13.99 | -6.50E-01 | 2.48E-04 | 1.09E-02 |
| *PIN1* | 293.87 | 283.85 | 266.94 | 324.55 | 362.10 | 341.43 | 4.04E-01 | 2.48E-04 | 1.09E-02 |
| *SLC1A3* | 24.10 | 25.77 | 23.24 | 17.73 | 18.07 | 16.90 | -5.64E-01 | 2.50E-04 | 1.09E-02 |
| *TMEM158* | 195.16 | 234.06 | 218.59 | 261.01 | 246.33 | 276.58 | 4.11E-01 | 2.50E-04 | 1.09E-02 |
| *NMI* | 39.43 | 38.73 | 43.53 | 26.77 | 29.50 | 28.63 | -5.58E-01 | 2.51E-04 | 1.10E-02 |
| *MCUB* | 145.93 | 144.37 | 124.73 | 183.40 | 164.93 | 175.76 | 4.72E-01 | 2.53E-04 | 1.10E-02 |
| *WARS1* | 159.26 | 176.77 | 166.38 | 100.97 | 126.64 | 100.77 | -4.92E-01 | 2.54E-04 | 1.10E-02 |
| *KLF4* | 34.06 | 42.28 | 39.06 | 28.47 | 23.60 | 26.07 | -6.03E-01 | 2.56E-04 | 1.11E-02 |
| *MMP14* | 69.62 | 52.83 | 63.58 | 89.07 | 84.29 | 86.12 | 5.62E-01 | 2.59E-04 | 1.12E-02 |
| *NMRAL2P* | 25.59 | 25.38 | 30.09 | 21.02 | 18.50 | 16.00 | -6.27E-01 | 2.59E-04 | 1.12E-02 |
| *TMEM120A* | 156.50 | 164.23 | 146.44 | 196.11 | 191.77 | 180.80 | 4.19E-01 | 2.62E-04 | 1.13E-02 |
| *H2BC4* | 39.30 | 48.03 | 49.33 | 66.42 | 60.10 | 74.24 | 5.87E-01 | 2.65E-04 | 1.14E-02 |
| *MRPS18A* | 307.39 | 376.03 | 360.53 | 219.58 | 256.44 | 235.27 | -4.25E-01 | 2.66E-04 | 1.14E-02 |
| *ATXN7L3B* | 281.50 | 366.19 | 353.53 | 190.89 | 216.69 | 241.81 | -5.00E-01 | 2.68E-04 | 1.14E-02 |
| *FAM167B* | 50.36 | 62.96 | 48.27 | 34.72 | 35.73 | 37.04 | -5.79E-01 | 2.67E-04 | 1.14E-02 |
| *SLC30A3* | 115.98 | 103.89 | 106.33 | 136.46 | 132.60 | 141.77 | 4.57E-01 | 2.68E-04 | 1.14E-02 |
| *NHSL1* | 50.25 | 69.30 | 62.56 | 33.60 | 41.34 | 40.07 | -6.44E-01 | 2.70E-04 | 1.14E-02 |
| *N4BP3* | 35.05 | 39.96 | 32.79 | 24.30 | 26.40 | 23.86 | -5.79E-01 | 2.71E-04 | 1.15E-02 |
| *MSANTD3* | 301.27 | 314.45 | 264.62 | 340.48 | 370.60 | 360.93 | 4.10E-01 | 2.75E-04 | 1.16E-02 |
| *IQCK* | 41.40 | 43.08 | 44.11 | 30.20 | 26.54 | 32.14 | -5.64E-01 | 2.76E-04 | 1.16E-02 |
| *LHX2* | 42.52 | 59.60 | 53.94 | 70.94 | 75.05 | 100.29 | 7.04E-01 | 2.87E-04 | 1.20E-02 |
| *SCN1A* | 21.34 | 20.77 | 24.74 | 38.81 | 32.84 | 50.27 | 7.74E-01 | 2.86E-04 | 1.20E-02 |
| *PRPH* | 65.39 | 50.91 | 46.40 | 84.89 | 76.87 | 76.43 | 6.26E-01 | 2.89E-04 | 1.21E-02 |
| *PUDP* | 368.29 | 465.77 | 428.10 | 287.17 | 307.36 | 272.19 | -4.17E-01 | 2.90E-04 | 1.21E-02 |
| *SPINK6* | 18.27 | 18.88 | 17.81 | 33.39 | 26.94 | 28.77 | 5.87E-01 | 2.92E-04 | 1.22E-02 |
| *ANHX* | 82.52 | 66.93 | 78.71 | 109.44 | 99.76 | 99.30 | 5.35E-01 | 2.94E-04 | 1.22E-02 |
| *THRA* | 128.45 | 120.26 | 140.23 | 148.17 | 170.16 | 172.30 | 4.66E-01 | 2.95E-04 | 1.22E-02 |
| *MSRA* | 215.98 | 226.30 | 201.75 | 147.62 | 153.35 | 135.67 | -4.22E-01 | 3.00E-04 | 1.24E-02 |
| *FBXL18* | 91.54 | 58.17 | 72.23 | 117.78 | 111.98 | 104.59 | 7.01E-01 | 3.01E-04 | 1.24E-02 |
| *NT5E* | 63.84 | 63.41 | 63.74 | 39.05 | 48.88 | 40.83 | -5.50E-01 | 3.01E-04 | 1.24E-02 |
| *TOP1* | 772.84 | 773.48 | 801.80 | 515.23 | 634.87 | 552.19 | -3.98E-01 | 3.04E-04 | 1.25E-02 |
| *TUBB3* | 362.00 | 318.90 | 304.78 | 397.42 | 454.91 | 401.35 | 4.68E-01 | 3.03E-04 | 1.25E-02 |
| *DERA* | 463.25 | 484.59 | 473.94 | 607.15 | 541.71 | 571.56 | 3.61E-01 | 3.06E-04 | 1.25E-02 |
| *FSTL1* | 1009.94 | 1149.43 | 1107.12 | 696.22 | 866.07 | 807.65 | -4.12E-01 | 3.07E-04 | 1.25E-02 |
| *P3H1* | 123.03 | 150.53 | 131.48 | 84.38 | 96.91 | 92.37 | -4.43E-01 | 3.05E-04 | 1.25E-02 |
| *RPRD1B* | 132.70 | 140.75 | 139.34 | 84.93 | 97.30 | 99.18 | -4.32E-01 | 3.07E-04 | 1.25E-02 |
| *MYH3* | 10.49 | 14.45 | 14.45 | 9.50 | 7.77 | 7.36 | -7.40E-01 | 3.12E-04 | 1.27E-02 |
| *LINC01085* | 10.45 | 8.23 | 6.68 | 18.98 | 13.10 | 19.98 | 8.84E-01 | 3.15E-04 | 1.28E-02 |
| *AVPR1B* | 75.00 | 54.68 | 54.04 | 104.60 | 91.18 | 83.12 | 6.82E-01 | 3.16E-04 | 1.28E-02 |
| *SLC41A3* | 227.33 | 297.16 | 234.70 | 173.09 | 161.67 | 167.65 | -4.55E-01 | 3.18E-04 | 1.29E-02 |
| *SLC30A1* | 1388.75 | 1390.39 | 1357.38 | 936.70 | 1149.65 | 1041.66 | -3.61E-01 | 3.20E-04 | 1.29E-02 |
| *GGCX* | 117.76 | 149.03 | 127.62 | 83.06 | 95.19 | 85.49 | -4.60E-01 | 3.25E-04 | 1.31E-02 |
| *OAZ2* | 258.59 | 281.94 | 263.10 | 333.09 | 332.33 | 295.02 | 3.80E-01 | 3.25E-04 | 1.31E-02 |
| *MAD1L1* | 46.58 | 60.81 | 58.25 | 35.93 | 40.44 | 32.65 | -5.94E-01 | 3.32E-04 | 1.33E-02 |
| *SYCP2* | 6.71 | 6.74 | 8.88 | 5.06 | 5.06 | 4.94 | -5.92E-01 | 3.33E-04 | 1.33E-02 |
| *ORAI2* | 190.08 | 172.13 | 182.43 | 199.46 | 275.95 | 264.23 | 5.66E-01 | 3.35E-04 | 1.34E-02 |
| *QSOX2* | 173.50 | 252.05 | 241.03 | 137.44 | 139.72 | 137.94 | -5.33E-01 | 3.35E-04 | 1.34E-02 |
| *SPATA32* | 108.44 | 78.86 | 81.46 | 133.62 | 122.18 | 121.62 | 6.15E-01 | 3.36E-04 | 1.34E-02 |
| *CNTD2* | 97.94 | 69.62 | 66.14 | 129.73 | 110.44 | 108.44 | 6.95E-01 | 3.39E-04 | 1.35E-02 |
| *ELOVL6* | 534.33 | 463.33 | 432.72 | 622.91 | 595.02 | 594.04 | 4.42E-01 | 3.40E-04 | 1.35E-02 |
| *POPDC3* | 78.77 | 79.57 | 82.60 | 105.58 | 98.68 | 98.68 | 4.30E-01 | 3.40E-04 | 1.35E-02 |
| *PRTFDC1* | 28.77 | 49.85 | 40.64 | 22.00 | 17.11 | 25.38 | -9.13E-01 | 3.43E-04 | 1.35E-02 |
| *MIR1915HG* | 110.18 | 98.44 | 85.80 | 149.31 | 127.61 | 124.31 | 5.68E-01 | 3.48E-04 | 1.37E-02 |
| *BACE1* | 107.42 | 132.75 | 134.74 | 76.63 | 73.60 | 92.19 | -5.14E-01 | 3.50E-04 | 1.38E-02 |
| *TRIM29* | 1346.82 | 1556.77 | 1444.10 | 1075.16 | 1181.38 | 1108.03 | -3.42E-01 | 3.51E-04 | 1.38E-02 |
| *STX17-AS1* | 349.29 | 207.92 | 243.18 | 450.85 | 391.95 | 396.83 | 7.78E-01 | 3.53E-04 | 1.38E-02 |
| *LINC00621* | 380.10 | 389.60 | 373.84 | 272.97 | 285.10 | 257.89 | -3.69E-01 | 3.54E-04 | 1.38E-02 |
| *ATN1* | 101.76 | 95.58 | 94.36 | 127.40 | 128.32 | 114.40 | 4.56E-01 | 3.56E-04 | 1.39E-02 |
| *C1orf226* | 46.63 | 57.28 | 49.35 | 28.21 | 32.77 | 38.78 | -6.43E-01 | 3.58E-04 | 1.39E-02 |
| *MAP4K5* | 265.48 | 162.11 | 167.60 | 293.99 | 294.53 | 316.73 | 7.82E-01 | 3.57E-04 | 1.39E-02 |
| *PAPPA2* | 59.66 | 44.66 | 46.60 | 74.97 | 71.62 | 70.16 | 5.78E-01 | 3.58E-04 | 1.39E-02 |
| *ETV4* | 57.13 | 60.00 | 64.78 | 44.96 | 40.94 | 30.60 | -6.33E-01 | 3.61E-04 | 1.40E-02 |
| *SPACA9* | 35.17 | 40.83 | 37.44 | 29.08 | 23.43 | 26.35 | -5.67E-01 | 3.63E-04 | 1.40E-02 |
| *ADAMTSL2* | 37.04 | 47.46 | 43.10 | 28.78 | 26.35 | 31.03 | -5.90E-01 | 3.64E-04 | 1.40E-02 |
| *INTU* | 29.86 | 44.27 | 35.05 | 25.98 | 21.07 | 22.44 | -6.81E-01 | 3.72E-04 | 1.43E-02 |
| *SP1* | 38.45 | 46.37 | 41.77 | 28.95 | 29.94 | 30.35 | -5.30E-01 | 3.74E-04 | 1.44E-02 |
| *LOC400684* | 32.05 | 36.82 | 37.31 | 24.94 | 27.34 | 21.39 | -5.85E-01 | 3.75E-04 | 1.44E-02 |
| *SAMD9* | 22.89 | 27.20 | 25.37 | 19.65 | 17.64 | 16.46 | -5.76E-01 | 3.75E-04 | 1.44E-02 |
| *GPR27* | 93.10 | 76.37 | 72.24 | 116.31 | 103.61 | 107.23 | 5.42E-01 | 3.81E-04 | 1.45E-02 |
| *ITGB5* | 112.25 | 152.39 | 115.80 | 84.60 | 74.01 | 84.64 | -5.17E-01 | 3.80E-04 | 1.45E-02 |
| *SLC16A4* | 5.95 | 5.99 | 6.97 | 3.65 | 4.09 | 4.88 | -6.04E-01 | 3.80E-04 | 1.45E-02 |
| *FAM83A* | 27.70 | 33.84 | 30.56 | 19.39 | 24.44 | 18.11 | -6.45E-01 | 3.84E-04 | 1.46E-02 |
| *ASB10* | 128.22 | 97.26 | 112.59 | 165.87 | 152.57 | 141.85 | 5.73E-01 | 3.85E-04 | 1.46E-02 |
| *FAM111A* | 42.27 | 50.26 | 47.07 | 29.31 | 34.61 | 32.43 | -5.49E-01 | 3.89E-04 | 1.47E-02 |
| *NLGN2* | 87.07 | 73.30 | 75.27 | 111.27 | 102.58 | 98.30 | 5.08E-01 | 3.92E-04 | 1.48E-02 |
| *CHST11* | 49.43 | 55.85 | 54.47 | 30.72 | 42.39 | 32.14 | -6.17E-01 | 3.99E-04 | 1.50E-02 |
| *FAM20C* | 120.56 | 147.60 | 161.46 | 83.17 | 96.61 | 97.56 | -5.04E-01 | 3.99E-04 | 1.50E-02 |
| *ARSG* | 33.17 | 35.27 | 34.67 | 23.77 | 19.59 | 26.70 | -6.28E-01 | 4.02E-04 | 1.51E-02 |
| *CENPV* | 510.17 | 551.01 | 531.69 | 594.57 | 710.69 | 631.53 | 3.61E-01 | 4.03E-04 | 1.51E-02 |
| *CLPB* | 131.26 | 157.00 | 142.92 | 176.87 | 173.13 | 164.78 | 3.90E-01 | 4.01E-04 | 1.51E-02 |
| *EEF1A2* | 74.15 | 83.33 | 94.72 | 114.94 | 102.10 | 109.56 | 4.87E-01 | 4.02E-04 | 1.51E-02 |
| *C14orf180* | 65.05 | 50.09 | 51.60 | 73.10 | 82.54 | 78.87 | 5.58E-01 | 4.05E-04 | 1.51E-02 |
| *TSPAN14* | 174.56 | 224.39 | 189.01 | 132.93 | 129.34 | 136.50 | -4.14E-01 | 4.07E-04 | 1.52E-02 |
| *NUFIP2* | 311.46 | 325.37 | 322.94 | 226.68 | 216.86 | 240.63 | -3.62E-01 | 4.09E-04 | 1.52E-02 |
| *TPK1* | 26.96 | 32.34 | 30.59 | 21.77 | 15.00 | 21.56 | -7.11E-01 | 4.09E-04 | 1.52E-02 |
| *UBE2F* | 115.60 | 115.59 | 103.30 | 75.01 | 76.16 | 78.96 | -4.33E-01 | 4.10E-04 | 1.52E-02 |
| *PSCA* | 65.19 | 93.10 | 75.27 | 51.76 | 32.77 | 48.67 | -7.71E-01 | 4.18E-04 | 1.55E-02 |
| *CENPL* | 126.38 | 134.49 | 133.92 | 149.07 | 162.82 | 155.47 | 3.75E-01 | 4.23E-04 | 1.56E-02 |
| *DDX5* | 190.05 | 226.28 | 219.21 | 155.12 | 130.77 | 145.08 | -4.30E-01 | 4.24E-04 | 1.56E-02 |
| *LRRK1* | 79.16 | 93.10 | 91.95 | 51.82 | 56.78 | 67.01 | -5.24E-01 | 4.28E-04 | 1.57E-02 |
| *LMX1A* | 4.00 | 3.44 | 3.21 | 5.97 | 5.54 | 5.14 | 5.22E-01 | 4.37E-04 | 1.60E-02 |
| *ASPH* | 62.33 | 79.39 | 76.06 | 47.98 | 51.22 | 49.10 | -4.91E-01 | 4.38E-04 | 1.60E-02 |
| *BRWD1* | 37.16 | 38.03 | 44.59 | 27.93 | 29.43 | 27.42 | -5.30E-01 | 4.46E-04 | 1.63E-02 |
| *HIVEP3* | 78.68 | 75.86 | 82.60 | 102.70 | 98.06 | 97.10 | 4.25E-01 | 4.48E-04 | 1.63E-02 |
| *IMPA1* | 993.29 | 1025.24 | 966.35 | 1352.54 | 1170.07 | 1148.31 | 3.38E-01 | 4.48E-04 | 1.63E-02 |
| *CETN3* | 439.51 | 347.75 | 330.06 | 535.10 | 479.69 | 483.44 | 5.37E-01 | 4.53E-04 | 1.65E-02 |
| *DTX2P1-UPK3BP1-PMS2P11* | 178.74 | 124.52 | 130.03 | 214.16 | 197.00 | 193.84 | 6.31E-01 | 4.55E-04 | 1.65E-02 |
| *SKIL* | 20.79 | 25.47 | 17.64 | 12.99 | 13.91 | 14.96 | -6.81E-01 | 4.56E-04 | 1.65E-02 |
| *SLC27A2* | 393.20 | 346.00 | 322.77 | 237.34 | 241.54 | 230.70 | -4.48E-01 | 4.56E-04 | 1.65E-02 |
| *ZNF512B* | 30.18 | 31.23 | 30.17 | 21.98 | 17.90 | 23.83 | -6.07E-01 | 4.56E-04 | 1.65E-02 |
| *PAM* | 831.52 | 967.50 | 886.28 | 677.54 | 626.88 | 694.38 | -3.52E-01 | 4.57E-04 | 1.65E-02 |
| *CCRL2* | 29.82 | 41.50 | 45.60 | 27.72 | 24.10 | 20.23 | -7.30E-01 | 4.62E-04 | 1.66E-02 |
| *CYP1A2* | 44.57 | 28.39 | 28.89 | 51.05 | 57.17 | 58.40 | 7.18E-01 | 4.64E-04 | 1.66E-02 |
| *RCC2* | 923.10 | 1065.17 | 976.38 | 654.51 | 777.96 | 766.77 | -3.74E-01 | 4.64E-04 | 1.66E-02 |
| *ENO2* | 60.94 | 96.27 | 86.38 | 103.36 | 113.54 | 117.07 | 5.82E-01 | 4.65E-04 | 1.66E-02 |
| *FLJ42351* | 36.55 | 43.36 | 48.35 | 30.62 | 27.68 | 29.44 | -5.65E-01 | 4.67E-04 | 1.67E-02 |
| *ANO7* | 112.34 | 115.32 | 116.61 | 141.51 | 139.78 | 131.65 | 3.91E-01 | 4.70E-04 | 1.67E-02 |
| *GTF2A2* | 893.54 | 900.59 | 860.83 | 1210.31 | 1092.47 | 988.82 | 3.57E-01 | 4.70E-04 | 1.67E-02 |
| *RNF157* | 48.43 | 28.03 | 33.94 | 65.08 | 56.45 | 58.36 | 7.33E-01 | 4.71E-04 | 1.67E-02 |
| *LINC02035* | 61.43 | 60.27 | 62.51 | 35.97 | 34.61 | 49.10 | -6.28E-01 | 4.72E-04 | 1.68E-02 |
| *ERLEC1* | 859.67 | 1060.20 | 1017.41 | 704.66 | 739.11 | 731.05 | -3.68E-01 | 4.74E-04 | 1.68E-02 |
| *MAD2L2* | 454.88 | 477.38 | 451.25 | 524.60 | 580.61 | 532.35 | 3.36E-01 | 4.77E-04 | 1.68E-02 |
| *CKAP4* | 670.50 | 795.03 | 793.95 | 501.43 | 582.15 | 557.16 | -3.87E-01 | 4.78E-04 | 1.69E-02 |
| *MAGEA12* | 127.43 | 139.88 | 115.46 | 88.88 | 88.79 | 82.02 | -4.38E-01 | 4.79E-04 | 1.69E-02 |
| *PLK2* | 360.46 | 402.12 | 371.56 | 276.94 | 280.43 | 250.33 | -3.68E-01 | 4.79E-04 | 1.69E-02 |
| *NUDT5* | 337.02 | 293.94 | 328.40 | 459.59 | 376.00 | 395.13 | 4.70E-01 | 4.82E-04 | 1.69E-02 |
| *ABL1* | 313.31 | 355.49 | 351.54 | 232.49 | 244.37 | 256.72 | -3.47E-01 | 4.83E-04 | 1.69E-02 |
| *TMEM254* | 32.34 | 27.14 | 27.67 | 23.93 | 19.01 | 17.74 | -6.06E-01 | 4.84E-04 | 1.69E-02 |
| *CCDC152* | 454.63 | 422.10 | 419.13 | 500.48 | 531.48 | 633.23 | 4.46E-01 | 4.86E-04 | 1.69E-02 |
| *PTH2* | 67.75 | 51.22 | 52.94 | 90.57 | 73.83 | 81.08 | 5.86E-01 | 4.86E-04 | 1.69E-02 |
| *APOA5* | 82.73 | 76.97 | 74.12 | 107.49 | 111.10 | 91.47 | 4.97E-01 | 4.90E-04 | 1.71E-02 |
| *KRTCAP2* | 1260.51 | 1282.02 | 1295.53 | 930.03 | 1086.75 | 985.32 | -3.17E-01 | 4.97E-04 | 1.73E-02 |
| *SIGLEC17P* | 8.87 | 7.55 | 5.73 | 14.22 | 13.94 | 11.30 | 7.09E-01 | 5.00E-04 | 1.73E-02 |
| *AVIL* | 37.34 | 49.31 | 45.60 | 31.09 | 29.94 | 30.10 | -5.44E-01 | 5.01E-04 | 1.74E-02 |
| *CAPN3* | 36.43 | 48.94 | 44.27 | 31.00 | 28.39 | 29.37 | -5.59E-01 | 5.02E-04 | 1.74E-02 |
| *ND6* | 828.34 | 568.39 | 549.85 | 819.11 | 1117.70 | 999.77 | 6.82E-01 | 5.09E-04 | 1.75E-02 |
| *RRNAD1* | 70.80 | 100.19 | 103.51 | 57.27 | 56.02 | 58.80 | -5.76E-01 | 5.08E-04 | 1.75E-02 |
| *DECR1* | 299.35 | 341.04 | 325.50 | 240.77 | 209.27 | 226.77 | -3.81E-01 | 5.12E-04 | 1.75E-02 |
| *KIAA1549* | 190.94 | 195.87 | 195.87 | 116.49 | 145.12 | 135.52 | -4.20E-01 | 5.12E-04 | 1.75E-02 |
| *PPP1R26-AS1* | 14.85 | 11.89 | 18.97 | 10.97 | 9.74 | 8.42 | -7.14E-01 | 5.10E-04 | 1.75E-02 |
| *KRTAP5-8* | 94.60 | 71.81 | 84.77 | 118.58 | 115.49 | 104.83 | 5.41E-01 | 5.19E-04 | 1.77E-02 |
| *MAPK12* | 71.26 | 58.43 | 69.63 | 98.52 | 91.69 | 82.43 | 5.30E-01 | 5.22E-04 | 1.78E-02 |
| *CDKN1C* | 75.03 | 86.49 | 78.06 | 52.75 | 49.76 | 61.58 | -4.86E-01 | 5.24E-04 | 1.79E-02 |
| *PCYT2* | 64.98 | 67.91 | 63.44 | 84.87 | 89.69 | 77.87 | 4.41E-01 | 5.27E-04 | 1.79E-02 |
| *EXOC3L2* | 75.15 | 63.60 | 75.25 | 103.89 | 91.47 | 89.88 | 5.03E-01 | 5.31E-04 | 1.80E-02 |
| *ADAMTS9-AS2* | 19.66 | 18.05 | 18.93 | 30.60 | 28.04 | 27.36 | 5.03E-01 | 5.33E-04 | 1.81E-02 |
| *SEC62* | 8.67 | 9.59 | 9.39 | 6.82 | 4.99 | 6.96 | -6.36E-01 | 5.34E-04 | 1.81E-02 |
| *PLPP3* | 51.76 | 56.17 | 49.80 | 35.03 | 40.90 | 26.28 | -6.47E-01 | 5.36E-04 | 1.81E-02 |
| *C11orf86* | 128.34 | 145.58 | 140.36 | 163.15 | 172.90 | 154.77 | 3.80E-01 | 5.38E-04 | 1.82E-02 |
| *AMOTL2* | 563.68 | 511.90 | 524.59 | 354.91 | 408.44 | 377.08 | -3.86E-01 | 5.47E-04 | 1.83E-02 |
| *FAM81A* | 86.39 | 103.87 | 108.64 | 73.73 | 63.63 | 59.62 | -5.06E-01 | 5.47E-04 | 1.83E-02 |
| *ID3* | 1346.62 | 1590.89 | 1544.08 | 2021.73 | 1727.14 | 2124.68 | 4.01E-01 | 5.52E-04 | 1.83E-02 |
| *KLHL13* | 397.42 | 319.94 | 353.44 | 428.10 | 481.54 | 448.04 | 4.61E-01 | 5.50E-04 | 1.83E-02 |
| *PFKP* | 1004.99 | 1300.30 | 1079.06 | 769.39 | 863.93 | 820.03 | -4.01E-01 | 5.46E-04 | 1.83E-02 |
| *SAMD13* | 32.88 | 31.25 | 39.77 | 24.70 | 15.90 | 24.52 | -7.57E-01 | 5.52E-04 | 1.83E-02 |
| *SSH3* | 101.00 | 91.93 | 97.74 | 129.68 | 118.74 | 116.77 | 4.41E-01 | 5.43E-04 | 1.83E-02 |
| *STAC2* | 74.56 | 74.23 | 74.96 | 95.67 | 89.57 | 93.20 | 4.04E-01 | 5.49E-04 | 1.83E-02 |
| *TOR1B* | 252.73 | 312.09 | 292.21 | 191.92 | 196.01 | 213.11 | -3.72E-01 | 5.44E-04 | 1.83E-02 |
| *TVP23A* | 17.21 | 17.20 | 20.27 | 14.48 | 13.36 | 9.93 | -6.45E-01 | 5.48E-04 | 1.83E-02 |
| *WDR47* | 163.77 | 144.73 | 152.23 | 204.00 | 175.40 | 193.93 | 4.54E-01 | 5.50E-04 | 1.83E-02 |
| *PSAT1* | 409.72 | 467.87 | 364.19 | 286.85 | 285.12 | 220.98 | -5.26E-01 | 5.53E-04 | 1.83E-02 |
| *PRICKLE2* | 6.96 | 6.15 | 6.00 | 9.98 | 10.73 | 9.30 | 5.16E-01 | 5.55E-04 | 1.83E-02 |
| *DNAJC6* | 377.77 | 362.10 | 341.88 | 422.11 | 417.84 | 473.94 | 3.93E-01 | 5.59E-04 | 1.84E-02 |
| *BMP6* | 98.59 | 111.24 | 109.06 | 65.27 | 80.63 | 70.97 | -4.56E-01 | 5.62E-04 | 1.84E-02 |
| *EIF5A* | 1291.51 | 1180.04 | 1070.16 | 1418.30 | 1551.89 | 1439.23 | 3.49E-01 | 5.60E-04 | 1.84E-02 |
| *SLC5A5* | 56.26 | 43.82 | 48.94 | 78.44 | 65.06 | 68.27 | 5.51E-01 | 5.61E-04 | 1.84E-02 |
| *ERLIN2* | 68.18 | 82.02 | 70.66 | 46.98 | 53.59 | 52.95 | -4.71E-01 | 5.67E-04 | 1.86E-02 |
| *MANBAL* | 330.84 | 422.47 | 411.12 | 276.81 | 269.09 | 259.87 | -3.99E-01 | 5.70E-04 | 1.87E-02 |
| *CD70* | 445.27 | 496.91 | 483.44 | 538.10 | 561.24 | 582.00 | 3.21E-01 | 5.72E-04 | 1.87E-02 |
| *MAN2A1* | 260.59 | 324.28 | 319.13 | 194.76 | 202.41 | 224.86 | -3.99E-01 | 5.73E-04 | 1.87E-02 |
| *DBF4* | 459.69 | 378.04 | 364.57 | 543.98 | 505.28 | 487.24 | 4.68E-01 | 5.75E-04 | 1.87E-02 |
| *VEGFC* | 21.71 | 32.44 | 24.18 | 17.27 | 16.21 | 17.48 | -6.72E-01 | 5.75E-04 | 1.87E-02 |
| *DUSP14* | 422.27 | 435.96 | 400.32 | 498.05 | 492.76 | 479.15 | 3.24E-01 | 5.76E-04 | 1.87E-02 |
| *XAGE2* | 93.94 | 112.81 | 94.55 | 137.90 | 160.44 | 112.56 | 5.59E-01 | 5.80E-04 | 1.88E-02 |
| *MGC16275* | 10.69 | 13.48 | 14.79 | 7.65 | 8.19 | 9.58 | -6.87E-01 | 5.83E-04 | 1.88E-02 |
| *FOXN3* | 62.82 | 41.99 | 50.93 | 80.05 | 73.03 | 73.01 | 6.03E-01 | 5.87E-04 | 1.90E-02 |
| *PCDHB15* | 10.70 | 10.95 | 11.12 | 7.28 | 7.66 | 8.67 | -5.51E-01 | 5.91E-04 | 1.90E-02 |
| *SLC38A9* | 49.88 | 49.94 | 45.35 | 35.41 | 33.66 | 35.83 | -4.77E-01 | 5.91E-04 | 1.90E-02 |
| *CYP2D6* | 69.10 | 50.81 | 56.96 | 83.95 | 76.79 | 82.79 | 5.36E-01 | 5.94E-04 | 1.90E-02 |
| *EEF2* | 4060.93 | 4509.88 | 4305.18 | 3370.66 | 3464.49 | 3716.01 | -3.17E-01 | 5.94E-04 | 1.90E-02 |
| *DNAAF4* | 26.10 | 29.27 | 26.74 | 21.84 | 17.65 | 19.94 | -5.49E-01 | 5.97E-04 | 1.91E-02 |
| *BCL2L11* | 88.82 | 74.27 | 61.52 | 99.43 | 102.22 | 104.20 | 5.50E-01 | 6.02E-04 | 1.92E-02 |
| *CDC42EP3* | 59.42 | 57.11 | 52.69 | 37.52 | 32.33 | 44.10 | -5.77E-01 | 6.00E-04 | 1.92E-02 |
| *UQCC1* | 135.10 | 143.95 | 125.18 | 95.97 | 94.31 | 91.37 | -3.99E-01 | 6.01E-04 | 1.92E-02 |
| *MAGEA1* | 32.55 | 33.94 | 33.66 | 23.45 | 24.10 | 26.09 | -5.06E-01 | 6.05E-04 | 1.93E-02 |
| *GKN1* | 19.28 | 24.01 | 19.76 | 14.45 | 14.16 | 15.90 | -5.88E-01 | 6.09E-04 | 1.93E-02 |
| *MASTL* | 202.19 | 184.79 | 192.10 | 228.29 | 223.63 | 245.78 | 3.90E-01 | 6.11E-04 | 1.94E-02 |
| *SPG21* | 102.78 | 91.03 | 113.83 | 116.27 | 139.74 | 158.56 | 5.44E-01 | 6.11E-04 | 1.94E-02 |
| *PLAG1* | 39.94 | 37.21 | 34.31 | 25.77 | 24.61 | 28.89 | -5.42E-01 | 6.14E-04 | 1.94E-02 |
| *SYT15* | 90.29 | 74.10 | 82.43 | 123.37 | 101.50 | 105.94 | 5.26E-01 | 6.13E-04 | 1.94E-02 |
| *AK1* | 320.56 | 396.89 | 348.72 | 396.37 | 443.82 | 436.58 | 3.69E-01 | 6.23E-04 | 1.96E-02 |
| *GAS6* | 125.04 | 110.62 | 110.62 | 145.91 | 134.45 | 149.11 | 4.36E-01 | 6.33E-04 | 1.99E-02 |
| *KEL* | 25.70 | 21.90 | 23.96 | 36.60 | 37.67 | 32.51 | 5.07E-01 | 6.33E-04 | 1.99E-02 |
| *MAP3K14* | 72.13 | 88.33 | 79.93 | 51.50 | 59.24 | 56.49 | -4.55E-01 | 6.34E-04 | 1.99E-02 |
| *FEM1C* | 67.44 | 62.01 | 68.91 | 47.15 | 43.18 | 49.44 | -4.72E-01 | 6.37E-04 | 1.99E-02 |
| *TOMM40L* | 107.58 | 123.07 | 114.94 | 127.15 | 145.57 | 138.94 | 3.80E-01 | 6.44E-04 | 2.01E-02 |
| *TAOK1* | 88.03 | 100.60 | 116.46 | 115.82 | 131.54 | 143.92 | 4.81E-01 | 6.47E-04 | 2.02E-02 |
| *CHMP4C* | 255.26 | 262.34 | 278.97 | 184.70 | 192.23 | 195.66 | -3.43E-01 | 6.49E-04 | 2.02E-02 |
| *CIAO2A* | 1650.59 | 1322.60 | 1399.05 | 1899.89 | 1769.61 | 1813.30 | 3.67E-01 | 6.51E-04 | 2.03E-02 |
| *SUMF1* | 496.81 | 517.63 | 523.27 | 377.65 | 376.64 | 395.63 | -3.14E-01 | 6.53E-04 | 2.03E-02 |
| *OR7E104P* | 226.88 | 156.10 | 199.06 | 296.92 | 247.68 | 263.10 | 6.18E-01 | 6.55E-04 | 2.03E-02 |
| *BICC1* | 499.87 | 448.25 | 474.84 | 314.43 | 346.30 | 352.26 | -3.76E-01 | 6.57E-04 | 2.04E-02 |
| *HOXC6* | 265.70 | 278.19 | 282.09 | 289.79 | 341.43 | 347.21 | 3.68E-01 | 6.70E-04 | 2.07E-02 |
| *LOC101927166* | 122.57 | 93.93 | 95.94 | 122.94 | 159.44 | 144.81 | 5.67E-01 | 6.69E-04 | 2.07E-02 |
| *GCA* | 19.70 | 18.18 | 14.45 | 28.26 | 26.65 | 29.04 | 5.88E-01 | 6.71E-04 | 2.07E-02 |
| *MYBL1* | 392.79 | 323.86 | 332.21 | 460.99 | 400.34 | 498.72 | 4.99E-01 | 6.72E-04 | 2.07E-02 |
| *MCC* | 42.27 | 31.43 | 36.99 | 55.77 | 50.71 | 54.54 | 5.33E-01 | 6.78E-04 | 2.08E-02 |
| *PCNX1* | 88.28 | 116.01 | 113.43 | 68.02 | 73.15 | 73.30 | -4.60E-01 | 6.79E-04 | 2.08E-02 |
| *SNRNP25* | 969.33 | 1090.84 | 1034.90 | 1228.27 | 1307.03 | 1190.96 | 3.01E-01 | 6.79E-04 | 2.08E-02 |
| *MICALL1* | 217.77 | 213.70 | 209.26 | 246.43 | 263.10 | 239.70 | 3.59E-01 | 6.82E-04 | 2.09E-02 |
| *ME3* | 35.02 | 39.10 | 38.28 | 29.60 | 28.07 | 24.41 | -5.01E-01 | 6.87E-04 | 2.10E-02 |
| *EIF4G2* | 119.88 | 88.10 | 87.70 | 149.02 | 128.42 | 130.43 | 5.91E-01 | 6.89E-04 | 2.10E-02 |
| *ITGA1* | 11.10 | 13.42 | 14.88 | 9.28 | 9.49 | 8.78 | -5.87E-01 | 6.97E-04 | 2.12E-02 |
| *HIKESHI* | 296.35 | 270.35 | 252.26 | 353.39 | 307.04 | 342.27 | 4.15E-01 | 7.02E-04 | 2.14E-02 |
| *C17orf82* | 74.94 | 52.68 | 45.47 | 87.88 | 82.36 | 85.09 | 6.55E-01 | 7.10E-04 | 2.14E-02 |
| *CPD* | 255.26 | 283.53 | 259.10 | 176.79 | 193.23 | 202.96 | -3.50E-01 | 7.06E-04 | 2.14E-02 |
| *DHRS7B* | 210.97 | 261.10 | 222.55 | 166.33 | 153.31 | 166.50 | -3.80E-01 | 7.10E-04 | 2.14E-02 |
| *FRG1HP* | 57.94 | 69.76 | 70.11 | 47.35 | 44.51 | 48.24 | -4.62E-01 | 7.11E-04 | 2.14E-02 |
| *FZD2* | 254.53 | 300.31 | 313.50 | 151.83 | 210.78 | 202.58 | -4.89E-01 | 7.05E-04 | 2.14E-02 |
| *GDPD5* | 112.18 | 142.50 | 136.92 | 157.44 | 159.73 | 151.17 | 3.95E-01 | 7.08E-04 | 2.14E-02 |
| *KIF21B* | 37.01 | 42.83 | 36.08 | 30.55 | 23.40 | 27.10 | -5.54E-01 | 7.08E-04 | 2.14E-02 |
| *NRGN* | 50.87 | 57.67 | 51.34 | 78.14 | 67.72 | 67.18 | 4.61E-01 | 7.13E-04 | 2.14E-02 |
| *OR1G1* | 29.88 | 24.30 | 28.59 | 40.23 | 39.58 | 40.77 | 4.94E-01 | 7.14E-04 | 2.14E-02 |
| *SOX4* | 44.57 | 30.85 | 28.03 | 57.90 | 50.92 | 53.94 | 6.63E-01 | 7.23E-04 | 2.17E-02 |
| *ZNF395* | 148.23 | 204.84 | 191.16 | 92.10 | 111.10 | 134.07 | -5.67E-01 | 7.24E-04 | 2.17E-02 |
| *BRD4* | 732.75 | 938.61 | 1012.68 | 536.09 | 627.92 | 652.91 | -4.91E-01 | 7.27E-04 | 2.17E-02 |
| *COA5* | 237.98 | 227.79 | 242.22 | 168.42 | 157.47 | 173.06 | -3.72E-01 | 7.27E-04 | 2.17E-02 |
| *FYCO1* | 58.97 | 48.97 | 48.97 | 71.32 | 69.81 | 68.98 | 4.71E-01 | 7.32E-04 | 2.18E-02 |
| *AK8* | 42.02 | 31.47 | 31.20 | 50.04 | 52.40 | 51.70 | 5.52E-01 | 7.38E-04 | 2.19E-02 |
| *CTPS1* | 424.00 | 467.43 | 463.23 | 325.68 | 358.06 | 313.86 | -3.30E-01 | 7.37E-04 | 2.19E-02 |
| *HPDL* | 220.06 | 244.39 | 224.39 | 252.97 | 265.05 | 269.30 | 3.25E-01 | 7.38E-04 | 2.19E-02 |
| *LOC105371967* | 86.74 | 101.31 | 92.56 | 60.83 | 72.39 | 59.39 | -4.56E-01 | 7.36E-04 | 2.19E-02 |
| *TARBP1* | 323.03 | 384.84 | 376.80 | 232.07 | 257.29 | 275.38 | -3.73E-01 | 7.40E-04 | 2.19E-02 |
| *SLC25A20* | 145.33 | 151.21 | 147.77 | 185.39 | 175.64 | 163.87 | 3.73E-01 | 7.41E-04 | 2.19E-02 |
| *NNMT* | 1489.37 | 1827.00 | 1811.47 | 1245.02 | 1354.63 | 1295.40 | -3.53E-01 | 7.50E-04 | 2.21E-02 |
| *ZCCHC14* | 380.15 | 452.20 | 453.41 | 267.99 | 321.03 | 319.27 | -3.82E-01 | 7.51E-04 | 2.21E-02 |
| *FRMD6* | 115.85 | 162.52 | 136.88 | 90.77 | 71.61 | 98.46 | -5.46E-01 | 7.54E-04 | 2.22E-02 |
| *CCDC82* | 17.01 | 17.72 | 17.83 | 14.48 | 11.37 | 12.94 | -5.43E-01 | 7.57E-04 | 2.22E-02 |
| *DPYSL2* | 894.36 | 839.52 | 791.77 | 572.75 | 614.03 | 683.61 | -3.68E-01 | 7.57E-04 | 2.22E-02 |
| *GPAT2* | 17.27 | 20.25 | 23.73 | 15.33 | 12.03 | 14.37 | -6.35E-01 | 7.60E-04 | 2.23E-02 |
| *RGMB* | 99.08 | 131.66 | 121.87 | 73.64 | 81.70 | 82.80 | -4.42E-01 | 7.62E-04 | 2.23E-02 |
| *SQOR* | 235.75 | 307.94 | 287.10 | 198.16 | 185.79 | 189.81 | -3.90E-01 | 7.66E-04 | 2.24E-02 |
| *SLC2A5* | 39.41 | 26.97 | 28.47 | 52.29 | 50.80 | 43.60 | 6.19E-01 | 7.70E-04 | 2.24E-02 |
| *TWSG1* | 46.41 | 55.35 | 54.81 | 36.32 | 40.43 | 35.07 | -4.83E-01 | 7.71E-04 | 2.24E-02 |
| *DHRS7* | 538.10 | 681.41 | 669.65 | 442.94 | 486.52 | 419.47 | -3.95E-01 | 7.77E-04 | 2.26E-02 |
| *YTHDF3* | 58.66 | 65.67 | 64.20 | 30.54 | 47.58 | 42.37 | -6.53E-01 | 7.80E-04 | 2.26E-02 |
| *BCL6* | 446.84 | 540.77 | 548.50 | 302.15 | 336.23 | 407.43 | -4.56E-01 | 7.84E-04 | 2.27E-02 |
| *BTNL9* | 47.80 | 32.47 | 38.50 | 60.11 | 56.31 | 57.98 | 5.68E-01 | 7.83E-04 | 2.27E-02 |
| *SLC22A25* | 91.90 | 55.07 | 52.87 | 108.43 | 97.20 | 100.82 | 7.41E-01 | 7.86E-04 | 2.27E-02 |
| *ETS2* | 277.33 | 312.48 | 303.66 | 208.32 | 221.07 | 221.76 | -3.20E-01 | 7.90E-04 | 2.28E-02 |
| *TRG-AS1* | 6.14 | 4.96 | 4.92 | 8.55 | 8.26 | 8.11 | 5.14E-01 | 7.89E-04 | 2.28E-02 |
| *COMMD8* | 891.79 | 567.27 | 500.98 | 1000.95 | 942.94 | 1026.60 | 7.19E-01 | 7.91E-04 | 2.28E-02 |
| *MMP28* | 15.89 | 15.10 | 16.34 | 11.97 | 12.82 | 11.04 | -5.01E-01 | 7.95E-04 | 2.28E-02 |
| *FGD2* | 240.14 | 154.01 | 165.27 | 270.17 | 255.43 | 258.13 | 6.52E-01 | 8.00E-04 | 2.29E-02 |
| *SNTA1* | 85.45 | 93.25 | 85.04 | 107.36 | 101.96 | 107.72 | 3.72E-01 | 8.01E-04 | 2.29E-02 |
| *TM9SF4* | 75.61 | 91.16 | 92.10 | 58.01 | 62.96 | 60.95 | -4.30E-01 | 8.04E-04 | 2.30E-02 |
| *ASB16* | 55.82 | 35.20 | 39.00 | 77.23 | 64.53 | 60.79 | 6.80E-01 | 8.11E-04 | 2.31E-02 |
| *CDKN1B* | 700.23 | 496.61 | 495.42 | 703.68 | 786.12 | 881.11 | 5.87E-01 | 8.13E-04 | 2.31E-02 |
| *GLP2R* | 39.57 | 41.58 | 46.24 | 32.20 | 28.49 | 31.75 | -4.85E-01 | 8.13E-04 | 2.31E-02 |
| *LINC00491* | 52.19 | 29.98 | 35.72 | 68.98 | 59.70 | 58.99 | 7.07E-01 | 8.13E-04 | 2.31E-02 |
| *PI3* | 78.24 | 83.43 | 88.56 | 63.86 | 48.94 | 57.78 | -4.91E-01 | 8.20E-04 | 2.33E-02 |
| *BDH1* | 29.55 | 26.94 | 25.17 | 42.56 | 37.92 | 38.29 | 4.85E-01 | 8.28E-04 | 2.34E-02 |
| *C5* | 15.25 | 18.57 | 20.55 | 11.84 | 11.61 | 13.79 | -6.32E-01 | 8.30E-04 | 2.34E-02 |
| *ZC4H2* | 37.12 | 27.68 | 30.98 | 49.90 | 45.85 | 44.22 | 5.27E-01 | 8.34E-04 | 2.35E-02 |
| *C9orf72* | 32.09 | 38.54 | 37.51 | 27.75 | 22.12 | 26.83 | -5.49E-01 | 8.35E-04 | 2.35E-02 |
| *LRRC52* | 28.20 | 27.87 | 26.50 | 47.72 | 43.43 | 34.73 | 5.54E-01 | 8.37E-04 | 2.35E-02 |
| *SLC39A11* | 81.76 | 81.60 | 87.87 | 54.28 | 62.84 | 60.77 | -4.35E-01 | 8.36E-04 | 2.35E-02 |
| *DDRGK1* | 206.58 | 270.36 | 257.25 | 162.07 | 179.07 | 160.78 | -4.07E-01 | 8.41E-04 | 2.35E-02 |
| *LOC220077* | 60.69 | 47.84 | 54.46 | 76.75 | 73.11 | 69.44 | 4.86E-01 | 8.41E-04 | 2.35E-02 |
| *C14orf178* | 56.46 | 46.17 | 44.30 | 72.72 | 68.97 | 62.32 | 5.17E-01 | 8.43E-04 | 2.35E-02 |
| *POLRMT* | 156.49 | 179.94 | 159.33 | 120.66 | 116.91 | 114.87 | -3.57E-01 | 8.46E-04 | 2.36E-02 |
| *DPCD* | 226.22 | 274.08 | 316.87 | 186.07 | 187.20 | 167.42 | -4.56E-01 | 8.51E-04 | 2.37E-02 |
| *PGBD2* | 58.31 | 75.27 | 71.37 | 49.61 | 48.71 | 45.42 | -4.66E-01 | 8.49E-04 | 2.37E-02 |
| *PPFIBP2* | 14.12 | 20.77 | 18.79 | 12.78 | 11.49 | 11.66 | -6.51E-01 | 8.52E-04 | 2.37E-02 |
| *PPL* | 170.25 | 223.17 | 226.94 | 137.20 | 129.88 | 149.13 | -4.34E-01 | 8.52E-04 | 2.37E-02 |
| *CROCCP2* | 80.76 | 77.16 | 79.94 | 100.00 | 95.10 | 96.46 | 3.89E-01 | 8.57E-04 | 2.37E-02 |
| *HES4* | 89.94 | 105.86 | 99.41 | 115.75 | 115.96 | 119.81 | 3.63E-01 | 8.57E-04 | 2.37E-02 |
| *DLX2* | 42.47 | 41.94 | 42.79 | 56.73 | 54.16 | 69.85 | 5.04E-01 | 8.63E-04 | 2.38E-02 |
| *WASHC2C* | 539.44 | 626.98 | 570.41 | 412.92 | 458.65 | 433.97 | -3.17E-01 | 8.64E-04 | 2.38E-02 |
| *BBOF1* | 42.80 | 40.89 | 39.31 | 33.35 | 28.17 | 29.21 | -4.77E-01 | 8.67E-04 | 2.39E-02 |
| *SLC2A3* | 155.07 | 179.75 | 158.33 | 116.94 | 120.54 | 110.76 | -3.64E-01 | 8.66E-04 | 2.39E-02 |
| *HMGB2* | 3052.01 | 2289.56 | 2238.97 | 3510.89 | 3221.35 | 3473.19 | 4.22E-01 | 8.70E-04 | 2.39E-02 |
| *HHLA1* | 91.61 | 80.75 | 74.60 | 116.06 | 114.09 | 96.10 | 5.03E-01 | 8.74E-04 | 2.39E-02 |
| *IGFBP4* | 39.56 | 37.66 | 42.55 | 29.14 | 29.17 | 30.55 | -4.69E-01 | 8.74E-04 | 2.39E-02 |
| *THUMPD3-AS1* | 29.31 | 36.44 | 29.66 | 24.99 | 20.73 | 22.44 | -5.46E-01 | 8.74E-04 | 2.39E-02 |
| *FBXO21* | 605.23 | 722.70 | 698.52 | 488.27 | 496.25 | 529.95 | -3.37E-01 | 8.77E-04 | 2.39E-02 |
| *SLC25A29* | 83.08 | 103.51 | 88.36 | 115.51 | 110.45 | 107.97 | 3.96E-01 | 8.77E-04 | 2.39E-02 |
| *NOP14* | 307.39 | 395.60 | 390.03 | 240.78 | 260.80 | 261.42 | -3.88E-01 | 8.82E-04 | 2.41E-02 |
| *MYO1E* | 278.16 | 311.24 | 312.95 | 227.44 | 213.04 | 207.73 | -3.38E-01 | 8.84E-04 | 2.41E-02 |
| *MGLL* | 343.27 | 475.77 | 424.03 | 283.13 | 269.37 | 294.55 | -4.16E-01 | 8.91E-04 | 2.42E-02 |
| *NFKB1* | 149.26 | 204.42 | 181.96 | 116.94 | 121.95 | 126.46 | -4.07E-01 | 8.96E-04 | 2.43E-02 |
| *CTTNBP2* | 31.26 | 34.10 | 29.68 | 48.18 | 40.49 | 54.60 | 5.55E-01 | 8.98E-04 | 2.44E-02 |
| *BABAM2-AS1* | 25.80 | 31.17 | 33.63 | 23.17 | 22.36 | 18.38 | -5.66E-01 | 9.08E-04 | 2.44E-02 |
| *CHAMP1* | 436.92 | 437.59 | 438.54 | 300.79 | 333.97 | 339.28 | -3.25E-01 | 9.07E-04 | 2.44E-02 |
| *DNAH17-AS1* | 82.60 | 106.77 | 89.23 | 50.15 | 61.44 | 70.50 | -5.49E-01 | 9.10E-04 | 2.44E-02 |
| *FAM53A* | 50.74 | 59.04 | 51.62 | 37.61 | 40.32 | 39.92 | -4.47E-01 | 9.10E-04 | 2.44E-02 |
| *GCFC2* | 301.05 | 192.40 | 184.74 | 351.02 | 304.59 | 318.91 | 6.93E-01 | 9.04E-04 | 2.44E-02 |
| *LRRN2* | 100.08 | 107.38 | 92.01 | 121.07 | 115.60 | 121.74 | 3.78E-01 | 9.08E-04 | 2.44E-02 |
| *MCCC1* | 172.89 | 201.96 | 180.19 | 135.62 | 130.47 | 127.59 | -3.52E-01 | 9.11E-04 | 2.44E-02 |
| *MUC1* | 32.87 | 37.88 | 33.97 | 20.77 | 26.81 | 25.96 | -5.70E-01 | 9.08E-04 | 2.44E-02 |
| *KRTAP11-1* | 35.94 | 28.76 | 30.21 | 45.68 | 47.99 | 42.67 | 4.96E-01 | 9.16E-04 | 2.45E-02 |
| *RHOQ* | 45.78 | 49.94 | 40.18 | 60.77 | 58.12 | 64.92 | 4.59E-01 | 9.19E-04 | 2.46E-02 |
| *BTD* | 77.88 | 85.54 | 72.58 | 59.49 | 54.40 | 54.67 | -4.17E-01 | 9.22E-04 | 2.46E-02 |
| *EIF4B* | 2184.26 | 2590.76 | 2621.99 | 1945.01 | 1828.75 | 2021.17 | -3.52E-01 | 9.30E-04 | 2.47E-02 |
| *FEM1B* | 185.15 | 184.37 | 194.43 | 125.37 | 136.10 | 141.10 | -3.54E-01 | 9.31E-04 | 2.47E-02 |
| *TMEM242* | 43.32 | 44.45 | 43.33 | 31.54 | 33.16 | 33.32 | -4.43E-01 | 9.29E-04 | 2.47E-02 |
| *ZNF84* | 129.44 | 157.29 | 165.03 | 101.95 | 103.60 | 107.38 | -3.91E-01 | 9.28E-04 | 2.47E-02 |
| *TRAPPC1* | 140.46 | 139.77 | 151.16 | 163.77 | 171.17 | 165.79 | 3.52E-01 | 9.44E-04 | 2.51E-02 |
| *C4orf36* | 4.42 | 7.69 | 7.30 | 3.69 | 4.01 | 3.89 | -7.09E-01 | 9.50E-04 | 2.51E-02 |
| *PER2* | 103.45 | 127.42 | 98.63 | 72.30 | 59.34 | 83.14 | -5.33E-01 | 9.51E-04 | 2.51E-02 |
| *PHOSPHO2* | 101.88 | 91.71 | 87.27 | 66.71 | 63.02 | 66.71 | -4.33E-01 | 9.52E-04 | 2.51E-02 |
| *RESF1* | 93.31 | 109.20 | 97.98 | 70.54 | 71.45 | 74.41 | -3.76E-01 | 9.49E-04 | 2.51E-02 |
| *ALDH3A1* | 51.45 | 68.79 | 55.95 | 40.20 | 41.63 | 42.27 | -4.81E-01 | 9.60E-04 | 2.52E-02 |
| *ANTXR1* | 138.66 | 173.86 | 147.55 | 103.46 | 112.75 | 105.34 | -3.79E-01 | 9.59E-04 | 2.52E-02 |
| *TDRD6* | 18.44 | 21.78 | 22.99 | 11.79 | 12.84 | 17.47 | -6.92E-01 | 9.58E-04 | 2.52E-02 |
| *CXCL2* | 12.47 | 12.00 | 13.01 | 9.48 | 10.37 | 8.10 | -5.13E-01 | 9.63E-04 | 2.53E-02 |
| *LINC01210* | 176.61 | 101.60 | 104.35 | 227.16 | 176.97 | 188.77 | 7.95E-01 | 9.64E-04 | 2.53E-02 |
| *BEST1* | 99.23 | 74.49 | 87.99 | 115.14 | 110.21 | 113.10 | 4.82E-01 | 9.68E-04 | 2.53E-02 |
| *LOC101926967* | 58.50 | 67.04 | 65.97 | 45.30 | 44.07 | 49.45 | -4.31E-01 | 9.71E-04 | 2.53E-02 |
| *PALM3* | 46.10 | 44.72 | 41.65 | 66.77 | 54.15 | 61.28 | 4.73E-01 | 9.70E-04 | 2.53E-02 |
| *TAC3* | 34.44 | 25.77 | 25.98 | 48.71 | 41.77 | 40.30 | 5.63E-01 | 9.70E-04 | 2.53E-02 |
| *DNAJC3* | 50.03 | 48.59 | 56.27 | 29.89 | 36.50 | 40.29 | -5.59E-01 | 9.74E-04 | 2.54E-02 |
| *TAMM41* | 108.44 | 125.31 | 101.16 | 79.02 | 81.27 | 72.05 | -4.17E-01 | 9.76E-04 | 2.54E-02 |
| *ACP6* | 310.34 | 359.51 | 342.64 | 246.42 | 253.21 | 240.62 | -3.18E-01 | 9.81E-04 | 2.55E-02 |
| *AMPD3* | 16.72 | 22.10 | 20.72 | 13.04 | 15.01 | 13.98 | -5.87E-01 | 9.87E-04 | 2.56E-02 |
| *PM20D2* | 511.02 | 346.99 | 353.88 | 550.60 | 521.77 | 596.77 | 5.89E-01 | 9.88E-04 | 2.56E-02 |
| *RNF165* | 13.55 | 14.91 | 11.98 | 10.67 | 9.50 | 9.51 | -5.32E-01 | 9.88E-04 | 2.56E-02 |
| *FOXO1* | 64.83 | 76.97 | 80.69 | 47.32 | 54.02 | 54.34 | -4.61E-01 | 9.90E-04 | 2.56E-02 |
| *GOT1* | 268.66 | 284.94 | 254.49 | 298.14 | 321.77 | 308.54 | 3.22E-01 | 9.96E-04 | 2.57E-02 |
| *LOC100506411* | 16.11 | 12.49 | 12.76 | 25.71 | 20.51 | 20.51 | 5.66E-01 | 9.96E-04 | 2.57E-02 |
| *OR10J1* | 54.71 | 41.84 | 40.79 | 72.12 | 61.71 | 62.10 | 5.44E-01 | 9.97E-04 | 2.57E-02 |
| *PXMP4* | 50.90 | 36.98 | 36.72 | 60.39 | 63.81 | 56.53 | 5.57E-01 | 9.97E-04 | 2.57E-02 |
| *DENND5A* | 464.88 | 591.18 | 594.88 | 382.58 | 381.69 | 418.10 | -3.82E-01 | 1.00E-03 | 2.57E-02 |
| *EGFLAM* | 109.80 | 119.64 | 128.42 | 88.18 | 84.32 | 80.32 | -3.87E-01 | 1.01E-03 | 2.57E-02 |
| *HBB* | 65.07 | 47.56 | 56.27 | 72.62 | 79.37 | 77.49 | 5.06E-01 | 1.00E-03 | 2.57E-02 |
| *LINC01963* | 21.71 | 29.43 | 26.32 | 16.69 | 20.16 | 17.05 | -5.93E-01 | 1.01E-03 | 2.57E-02 |
| *NOG* | 51.29 | 49.08 | 46.59 | 73.22 | 59.04 | 93.25 | 6.29E-01 | 1.00E-03 | 2.57E-02 |
| *RAB27B* | 13.17 | 14.20 | 17.20 | 12.04 | 10.67 | 8.37 | -6.16E-01 | 1.00E-03 | 2.57E-02 |
| *SC5D* | 396.58 | 338.50 | 336.51 | 488.85 | 437.19 | 408.27 | 4.26E-01 | 1.01E-03 | 2.57E-02 |
| *BCORL1* | 162.88 | 152.79 | 135.08 | 161.35 | 209.75 | 195.57 | 4.63E-01 | 1.01E-03 | 2.57E-02 |
| *CDC14B* | 64.99 | 86.98 | 84.64 | 53.00 | 52.21 | 56.82 | -4.70E-01 | 1.02E-03 | 2.57E-02 |
| *CTSB* | 301.27 | 316.01 | 291.94 | 224.76 | 214.76 | 222.17 | -3.23E-01 | 1.01E-03 | 2.57E-02 |
| *HAGHL* | 83.18 | 79.65 | 78.01 | 96.86 | 94.82 | 101.30 | 3.79E-01 | 1.02E-03 | 2.57E-02 |
| *MTR* | 383.08 | 338.22 | 350.76 | 402.85 | 434.25 | 445.92 | 3.71E-01 | 1.02E-03 | 2.57E-02 |
| *MYC* | 2591.53 | 2944.16 | 2966.66 | 2167.36 | 2193.08 | 2451.23 | -3.41E-01 | 1.02E-03 | 2.57E-02 |
| *PICART1* | 7.34 | 7.10 | 6.44 | 11.28 | 9.81 | 11.67 | 5.13E-01 | 1.02E-03 | 2.57E-02 |
| *VSIR* | 20.90 | 21.12 | 21.00 | 14.45 | 15.68 | 17.13 | -5.12E-01 | 1.01E-03 | 2.57E-02 |
| *ATPAF1* | 134.73 | 141.80 | 126.53 | 98.66 | 87.60 | 97.22 | -3.85E-01 | 1.02E-03 | 2.57E-02 |
| *H1-4* | 57.55 | 55.87 | 50.43 | 71.10 | 66.93 | 75.26 | 4.28E-01 | 1.02E-03 | 2.57E-02 |
| *SAMD8* | 75.51 | 77.24 | 89.37 | 58.38 | 58.09 | 56.47 | -4.22E-01 | 1.02E-03 | 2.57E-02 |
| *ZBTB26* | 38.25 | 39.68 | 33.64 | 29.78 | 21.78 | 26.69 | -5.63E-01 | 1.02E-03 | 2.57E-02 |
| *BMP2* | 1278.76 | 1081.54 | 1015.36 | 669.50 | 921.96 | 774.77 | -4.62E-01 | 1.03E-03 | 2.57E-02 |
| *EIF1B-AS1* | 46.20 | 37.21 | 36.96 | 56.48 | 60.93 | 52.85 | 5.01E-01 | 1.03E-03 | 2.57E-02 |
| *APOBEC3B* | 481.38 | 307.83 | 418.76 | 537.41 | 575.72 | 575.99 | 6.06E-01 | 1.03E-03 | 2.58E-02 |
| *NME4* | 147.77 | 176.90 | 162.35 | 180.02 | 201.41 | 183.46 | 3.46E-01 | 1.03E-03 | 2.58E-02 |
| *MCMBP* | 257.06 | 302.15 | 280.61 | 202.21 | 192.43 | 212.75 | -3.31E-01 | 1.04E-03 | 2.59E-02 |
| *SRGAP2C* | 363.48 | 427.69 | 401.10 | 422.69 | 491.81 | 526.62 | 3.77E-01 | 1.04E-03 | 2.59E-02 |
| *KLK4* | 110.77 | 72.44 | 89.18 | 119.81 | 123.45 | 125.95 | 5.67E-01 | 1.04E-03 | 2.59E-02 |
| *CDH4* | 9.80 | 8.51 | 8.32 | 14.91 | 14.81 | 12.10 | 5.16E-01 | 1.05E-03 | 2.61E-02 |
| *LIF* | 55.96 | 61.68 | 63.79 | 45.05 | 45.85 | 42.08 | -4.20E-01 | 1.05E-03 | 2.61E-02 |
| *PTGER1* | 53.18 | 40.49 | 50.44 | 80.44 | 63.50 | 64.02 | 5.65E-01 | 1.05E-03 | 2.61E-02 |
| *ARHGAP18* | 116.41 | 163.90 | 142.55 | 90.75 | 84.22 | 104.80 | -4.67E-01 | 1.05E-03 | 2.61E-02 |
| *PRR29* | 49.19 | 41.58 | 50.78 | 82.60 | 58.53 | 67.02 | 5.80E-01 | 1.07E-03 | 2.64E-02 |
| *MAP3K5* | 82.07 | 107.02 | 105.57 | 66.18 | 67.22 | 70.46 | -4.33E-01 | 1.07E-03 | 2.65E-02 |
| *H2AJ* | 31.77 | 40.40 | 27.94 | 19.00 | 16.35 | 26.40 | -7.61E-01 | 1.08E-03 | 2.67E-02 |
| *DSCR10* | 87.09 | 68.63 | 79.06 | 96.10 | 101.63 | 101.06 | 4.48E-01 | 1.08E-03 | 2.67E-02 |
| *PAQR5* | 41.78 | 32.85 | 34.36 | 26.90 | 24.69 | 26.99 | -5.21E-01 | 1.09E-03 | 2.68E-02 |
| *PHF20* | 60.08 | 54.12 | 48.36 | 39.01 | 39.01 | 38.42 | -4.72E-01 | 1.09E-03 | 2.68E-02 |
| *LINC02097* | 11.27 | 8.46 | 7.72 | 14.97 | 16.91 | 13.69 | 6.04E-01 | 1.09E-03 | 2.68E-02 |
| *USP34* | 251.32 | 231.97 | 268.56 | 267.39 | 315.56 | 363.95 | 4.55E-01 | 1.09E-03 | 2.68E-02 |
| *PKIA* | 79.88 | 71.77 | 70.11 | 97.67 | 86.20 | 98.12 | 4.29E-01 | 1.10E-03 | 2.69E-02 |
| *ATP5F1E* | 5451.99 | 4051.77 | 3899.94 | 6013.97 | 5807.53 | 5685.59 | 3.82E-01 | 1.10E-03 | 2.69E-02 |
| *RECQL* | 274.21 | 154.10 | 130.39 | 327.87 | 261.87 | 307.71 | 8.88E-01 | 1.10E-03 | 2.69E-02 |
| *TMEM37* | 99.61 | 74.16 | 81.38 | 126.93 | 112.68 | 103.56 | 5.38E-01 | 1.10E-03 | 2.70E-02 |
| *LPIN2* | 92.67 | 129.24 | 118.51 | 77.27 | 71.12 | 80.59 | -4.50E-01 | 1.11E-03 | 2.71E-02 |
| *ANO2* | 113.83 | 125.29 | 115.11 | 65.04 | 95.64 | 54.98 | -6.57E-01 | 1.12E-03 | 2.72E-02 |
| *C1QTNF1-AS1* | 84.07 | 94.20 | 102.21 | 57.03 | 57.91 | 74.60 | -4.97E-01 | 1.13E-03 | 2.72E-02 |
| *GINS2* | 60.10 | 43.40 | 46.64 | 70.19 | 74.26 | 65.45 | 5.33E-01 | 1.12E-03 | 2.72E-02 |
| *GORASP2* | 663.53 | 721.05 | 747.59 | 510.85 | 579.88 | 545.15 | -3.15E-01 | 1.12E-03 | 2.72E-02 |
| *PRMT7* | 125.26 | 156.95 | 158.34 | 93.54 | 104.70 | 106.30 | -3.98E-01 | 1.12E-03 | 2.72E-02 |
| *GABRA3* | 14.63 | 15.48 | 17.00 | 12.32 | 9.95 | 12.24 | -5.44E-01 | 1.13E-03 | 2.74E-02 |
| *STAT3* | 953.55 | 961.23 | 1009.94 | 645.78 | 813.72 | 746.27 | -3.47E-01 | 1.14E-03 | 2.76E-02 |
| *ASRGL1* | 165.50 | 206.92 | 188.28 | 212.60 | 217.63 | 213.88 | 3.36E-01 | 1.14E-03 | 2.76E-02 |
| *OR10H3* | 14.13 | 10.57 | 12.18 | 19.82 | 19.11 | 18.38 | 5.12E-01 | 1.14E-03 | 2.76E-02 |
| *ZNF302* | 17.74 | 14.26 | 14.71 | 11.69 | 10.80 | 12.32 | -5.20E-01 | 1.15E-03 | 2.76E-02 |
| *PGD* | 18.97 | 15.25 | 15.49 | 26.85 | 26.38 | 23.16 | 5.05E-01 | 1.15E-03 | 2.77E-02 |
| *REEP1* | 80.88 | 99.21 | 93.89 | 59.53 | 68.48 | 66.10 | -4.14E-01 | 1.15E-03 | 2.77E-02 |
| *CRLF3* | 491.25 | 479.19 | 489.55 | 558.84 | 547.47 | 587.43 | 3.04E-01 | 1.16E-03 | 2.78E-02 |
| *RPL31* | 422.27 | 478.92 | 462.70 | 366.10 | 265.05 | 309.10 | -4.31E-01 | 1.16E-03 | 2.78E-02 |
| *VMP1* | 101.32 | 116.95 | 132.18 | 81.82 | 79.35 | 81.72 | -4.11E-01 | 1.16E-03 | 2.78E-02 |
| *ERVH-1* | 89.80 | 75.91 | 83.20 | 103.62 | 100.94 | 103.77 | 4.08E-01 | 1.17E-03 | 2.78E-02 |
| *NPTXR* | 19.79 | 24.90 | 30.57 | 16.20 | 16.78 | 17.68 | -6.30E-01 | 1.17E-03 | 2.78E-02 |
| *REPS2* | 51.27 | 55.36 | 53.15 | 40.65 | 38.57 | 40.79 | -4.10E-01 | 1.17E-03 | 2.78E-02 |
| *C9orf64* | 259.02 | 241.22 | 285.54 | 278.82 | 327.08 | 352.09 | 4.09E-01 | 1.17E-03 | 2.78E-02 |
| *CREB3L2* | 726.05 | 766.91 | 733.49 | 500.08 | 605.34 | 585.11 | -3.30E-01 | 1.18E-03 | 2.80E-02 |
| *HYMAI* | 44.00 | 56.39 | 51.60 | 37.15 | 35.94 | 37.10 | -4.57E-01 | 1.19E-03 | 2.80E-02 |
| *KLHL42* | 492.07 | 518.03 | 484.98 | 318.83 | 327.10 | 415.00 | -3.97E-01 | 1.18E-03 | 2.80E-02 |
| *SYNGR3* | 16.25 | 20.23 | 18.94 | 35.72 | 24.90 | 31.44 | 6.25E-01 | 1.19E-03 | 2.80E-02 |
| *CKMT2-AS1* | 71.60 | 65.92 | 66.23 | 47.25 | 44.82 | 53.79 | -4.48E-01 | 1.19E-03 | 2.81E-02 |
| *ATL1* | 123.12 | 117.60 | 140.07 | 162.77 | 150.49 | 146.95 | 4.04E-01 | 1.20E-03 | 2.82E-02 |
| *DNAJB6* | 94.91 | 110.97 | 108.04 | 67.12 | 77.14 | 77.91 | -3.97E-01 | 1.20E-03 | 2.82E-02 |
| *LOC101929398* | 29.62 | 23.82 | 25.97 | 37.06 | 38.69 | 39.55 | 4.76E-01 | 1.20E-03 | 2.82E-02 |
| *TTLL1* | 28.82 | 39.34 | 27.46 | 23.71 | 19.32 | 22.11 | -6.02E-01 | 1.20E-03 | 2.82E-02 |
| *LCN15* | 45.49 | 32.77 | 34.91 | 59.60 | 50.94 | 53.70 | 5.42E-01 | 1.21E-03 | 2.83E-02 |
| *WDFY3* | 22.87 | 30.87 | 27.94 | 19.29 | 18.34 | 20.63 | -5.55E-01 | 1.21E-03 | 2.83E-02 |
| *SYNPO2* | 16.19 | 11.82 | 12.17 | 23.25 | 21.82 | 19.02 | 5.55E-01 | 1.21E-03 | 2.83E-02 |
| *DHRS9* | 71.30 | 61.71 | 59.39 | 89.36 | 82.64 | 77.18 | 4.53E-01 | 1.21E-03 | 2.83E-02 |
| *MICB* | 726.54 | 655.75 | 585.01 | 748.16 | 849.61 | 819.28 | 3.77E-01 | 1.22E-03 | 2.84E-02 |
| *APCDD1L-DT* | 102.27 | 105.77 | 87.29 | 122.94 | 118.33 | 116.27 | 3.94E-01 | 1.22E-03 | 2.85E-02 |
| *HS6ST2* | 26.08 | 19.22 | 21.86 | 15.19 | 18.51 | 14.58 | -5.65E-01 | 1.23E-03 | 2.86E-02 |
| *RAB22A* | 157.60 | 205.77 | 201.76 | 124.86 | 114.69 | 142.01 | -4.31E-01 | 1.23E-03 | 2.86E-02 |
| *MMP19* | 66.44 | 63.07 | 57.44 | 81.44 | 83.10 | 73.73 | 4.19E-01 | 1.24E-03 | 2.88E-02 |
| *CDADC1* | 18.69 | 19.15 | 26.24 | 14.54 | 13.16 | 16.38 | -6.18E-01 | 1.25E-03 | 2.89E-02 |
| *CYP26B1* | 71.14 | 84.77 | 76.35 | 49.23 | 58.18 | 57.56 | -4.31E-01 | 1.25E-03 | 2.89E-02 |
| *LOC102723409* | 9.23 | 10.69 | 7.98 | 6.44 | 6.54 | 7.00 | -5.40E-01 | 1.25E-03 | 2.89E-02 |
| *DEF8* | 99.87 | 110.38 | 109.84 | 129.91 | 149.12 | 115.65 | 4.25E-01 | 1.26E-03 | 2.90E-02 |
| *NOP14-AS1* | 40.18 | 53.45 | 56.97 | 34.08 | 36.50 | 32.76 | -5.35E-01 | 1.26E-03 | 2.92E-02 |
| *ISG20* | 37.53 | 56.62 | 49.42 | 33.30 | 29.53 | 33.24 | -5.70E-01 | 1.27E-03 | 2.92E-02 |
| *TSPAN31* | 86.21 | 85.77 | 97.90 | 103.44 | 110.10 | 112.87 | 3.82E-01 | 1.27E-03 | 2.92E-02 |
| *IL1R1* | 7.53 | 6.77 | 8.18 | 5.22 | 4.88 | 6.19 | -5.14E-01 | 1.28E-03 | 2.94E-02 |
| *RABL6* | 199.98 | 257.82 | 259.18 | 295.09 | 288.76 | 267.69 | 3.83E-01 | 1.28E-03 | 2.95E-02 |
| *FKTN* | 100.77 | 109.72 | 111.45 | 69.22 | 72.26 | 85.74 | -4.08E-01 | 1.29E-03 | 2.95E-02 |
| *HIVEP2* | 42.27 | 58.19 | 50.46 | 32.33 | 31.98 | 39.52 | -5.44E-01 | 1.30E-03 | 2.95E-02 |
| *LOC101927513* | 19.17 | 13.69 | 14.13 | 26.20 | 23.02 | 25.26 | 5.58E-01 | 1.30E-03 | 2.95E-02 |
| *MICOS13* | 515.94 | 627.94 | 589.85 | 765.36 | 704.44 | 635.32 | 3.54E-01 | 1.29E-03 | 2.95E-02 |
| *NHS* | 166.78 | 205.69 | 191.22 | 114.06 | 126.48 | 149.47 | -4.06E-01 | 1.30E-03 | 2.95E-02 |
| *NR1H2* | 57.97 | 64.20 | 53.76 | 40.84 | 44.42 | 43.32 | -4.30E-01 | 1.30E-03 | 2.95E-02 |
| *PLA2G2A* | 27.94 | 25.81 | 27.54 | 38.64 | 38.96 | 36.27 | 4.31E-01 | 1.29E-03 | 2.95E-02 |
| *SLC2A13* | 3.41 | 3.62 | 3.63 | 2.82 | 2.57 | 2.63 | -3.78E-01 | 1.29E-03 | 2.95E-02 |
| *UTS2R* | 130.17 | 90.23 | 84.77 | 152.98 | 134.97 | 133.69 | 6.08E-01 | 1.29E-03 | 2.95E-02 |
| *CCDC33* | 95.68 | 69.84 | 75.27 | 109.77 | 103.46 | 101.81 | 5.00E-01 | 1.31E-03 | 2.95E-02 |
| *CDK7* | 284.18 | 340.56 | 316.95 | 245.23 | 212.91 | 201.13 | -3.81E-01 | 1.30E-03 | 2.95E-02 |
| *CHAC1* | 35.95 | 38.61 | 37.66 | 23.25 | 32.54 | 23.27 | -5.70E-01 | 1.30E-03 | 2.95E-02 |
| *PRRT3-AS1* | 167.39 | 143.92 | 148.32 | 179.71 | 212.68 | 176.02 | 4.34E-01 | 1.31E-03 | 2.95E-02 |
| *ARMC4* | 60.46 | 46.60 | 53.36 | 74.18 | 71.40 | 67.73 | 4.69E-01 | 1.31E-03 | 2.96E-02 |
| *HSPA4L* | 225.34 | 201.24 | 213.95 | 231.67 | 263.28 | 259.75 | 3.71E-01 | 1.32E-03 | 2.97E-02 |
| *CREG1* | 982.06 | 843.58 | 871.43 | 670.71 | 710.35 | 674.06 | -3.24E-01 | 1.32E-03 | 2.98E-02 |
| *ST3GAL5* | 73.60 | 74.98 | 76.06 | 56.64 | 53.66 | 56.13 | -3.74E-01 | 1.33E-03 | 2.98E-02 |
| *ZBTB1* | 21.07 | 22.75 | 26.87 | 16.87 | 18.39 | 17.15 | -5.14E-01 | 1.33E-03 | 2.98E-02 |
| *MAP3K8* | 21.42 | 21.51 | 18.34 | 14.94 | 13.09 | 16.90 | -5.49E-01 | 1.33E-03 | 2.99E-02 |
| *LINC00587* | 6.14 | 6.37 | 6.42 | 4.80 | 5.40 | 4.15 | -4.56E-01 | 1.34E-03 | 3.00E-02 |
| *NPW* | 9.15 | 10.56 | 10.35 | 6.97 | 8.29 | 7.09 | -5.08E-01 | 1.34E-03 | 3.00E-02 |
| *TPP2* | 18.70 | 30.23 | 28.94 | 16.67 | 16.91 | 16.77 | -6.72E-01 | 1.34E-03 | 3.00E-02 |
| *ATP10D* | 89.46 | 92.38 | 88.80 | 63.75 | 61.44 | 71.21 | -3.85E-01 | 1.35E-03 | 3.00E-02 |
| *MSN* | 1168.21 | 900.47 | 974.30 | 658.07 | 785.33 | 716.46 | -4.13E-01 | 1.35E-03 | 3.00E-02 |
| *FANCF* | 130.92 | 175.46 | 165.48 | 101.85 | 107.29 | 116.11 | -3.97E-01 | 1.36E-03 | 3.02E-02 |
| *POM121L12* | 130.38 | 70.10 | 75.27 | 141.95 | 137.13 | 132.60 | 7.45E-01 | 1.35E-03 | 3.02E-02 |
| *RAD51AP1* | 778.05 | 429.10 | 416.77 | 916.96 | 728.22 | 913.94 | 8.01E-01 | 1.36E-03 | 3.02E-02 |
| *WAKMAR2* | 19.07 | 19.46 | 15.97 | 16.55 | 9.02 | 10.30 | -7.36E-01 | 1.36E-03 | 3.02E-02 |
| *GEMIN8* | 43.10 | 48.14 | 48.39 | 32.16 | 35.10 | 36.27 | -4.44E-01 | 1.37E-03 | 3.03E-02 |
| *KCTD17* | 56.48 | 39.77 | 37.16 | 62.93 | 68.48 | 61.69 | 5.76E-01 | 1.37E-03 | 3.03E-02 |
| *AHRR* | 1168.04 | 1234.33 | 1376.28 | 887.96 | 986.26 | 1015.01 | -3.31E-01 | 1.38E-03 | 3.04E-02 |
| *CTSD* | 84.83 | 92.75 | 98.93 | 55.91 | 67.45 | 70.25 | -4.44E-01 | 1.38E-03 | 3.04E-02 |
| *MEST* | 306.83 | 350.21 | 356.05 | 386.84 | 421.26 | 364.49 | 3.26E-01 | 1.38E-03 | 3.05E-02 |
| *CCN5* | 975.33 | 1044.86 | 1046.89 | 770.81 | 584.81 | 836.58 | -4.42E-01 | 1.38E-03 | 3.05E-02 |
| *TMEM87B* | 45.12 | 47.34 | 41.77 | 31.26 | 25.03 | 36.89 | -5.72E-01 | 1.39E-03 | 3.05E-02 |
| *BBS1* | 121.23 | 142.97 | 132.10 | 97.13 | 83.98 | 98.93 | -3.77E-01 | 1.39E-03 | 3.06E-02 |
| *HVCN1* | 69.10 | 47.60 | 43.22 | 76.69 | 72.64 | 77.72 | 5.87E-01 | 1.40E-03 | 3.08E-02 |
| *TFF1* | 18.28 | 15.42 | 15.43 | 27.52 | 23.92 | 23.27 | 4.84E-01 | 1.40E-03 | 3.08E-02 |
| *H2AC14* | 167.53 | 184.94 | 168.27 | 214.99 | 191.56 | 195.67 | 3.41E-01 | 1.41E-03 | 3.10E-02 |
| *FMR1* | 221.44 | 227.83 | 235.69 | 159.66 | 164.87 | 175.93 | -3.17E-01 | 1.42E-03 | 3.10E-02 |
| *LOC389765* | 32.04 | 37.03 | 40.18 | 26.76 | 26.25 | 27.89 | -4.80E-01 | 1.41E-03 | 3.10E-02 |
| *ZBTB8OS* | 250.54 | 250.78 | 236.12 | 310.68 | 274.13 | 272.09 | 3.46E-01 | 1.42E-03 | 3.11E-02 |
| *PARN* | 364.05 | 365.57 | 348.67 | 267.72 | 252.05 | 271.61 | -3.12E-01 | 1.42E-03 | 3.11E-02 |
| *CDC42SE2* | 126.46 | 140.72 | 141.77 | 103.16 | 95.71 | 96.34 | -3.46E-01 | 1.44E-03 | 3.13E-02 |
| *EXT2* | 245.78 | 321.71 | 265.28 | 192.19 | 200.71 | 198.35 | -3.50E-01 | 1.44E-03 | 3.13E-02 |
| *JUP* | 1313.21 | 1405.20 | 1466.66 | 1056.48 | 1034.82 | 1197.59 | -3.18E-01 | 1.44E-03 | 3.13E-02 |
| *KIR2DL5A* | 12.13 | 9.01 | 9.59 | 16.22 | 16.33 | 15.13 | 5.16E-01 | 1.45E-03 | 3.13E-02 |
| *MYT1L* | 34.18 | 41.04 | 38.73 | 28.89 | 30.32 | 25.40 | -4.69E-01 | 1.45E-03 | 3.13E-02 |
| *PNPO* | 178.56 | 169.29 | 166.10 | 197.86 | 200.21 | 192.52 | 3.41E-01 | 1.44E-03 | 3.13E-02 |
| *RNF187* | 53.69 | 44.49 | 55.09 | 72.96 | 69.71 | 62.60 | 4.65E-01 | 1.45E-03 | 3.13E-02 |
| *SIX4* | 131.02 | 154.81 | 146.03 | 95.24 | 102.11 | 111.67 | -3.55E-01 | 1.44E-03 | 3.13E-02 |
| *THAP10* | 140.35 | 138.56 | 136.70 | 92.61 | 107.08 | 97.08 | -3.64E-01 | 1.45E-03 | 3.13E-02 |
| *HERC2* | 238.07 | 314.54 | 290.62 | 157.44 | 191.94 | 216.23 | -4.43E-01 | 1.47E-03 | 3.17E-02 |
| *LINC01278* | 110.52 | 129.08 | 107.30 | 83.06 | 79.53 | 86.00 | -3.65E-01 | 1.47E-03 | 3.17E-02 |
| *PKMYT1* | 217.77 | 220.45 | 223.44 | 242.31 | 247.18 | 256.27 | 3.02E-01 | 1.48E-03 | 3.19E-02 |
| *GLI2* | 42.10 | 50.82 | 46.81 | 30.47 | 35.92 | 35.18 | -4.73E-01 | 1.48E-03 | 3.19E-02 |
| *POGLUT1* | 240.10 | 299.06 | 283.19 | 203.24 | 197.10 | 189.02 | -3.36E-01 | 1.48E-03 | 3.19E-02 |
| *UBE3B* | 126.75 | 153.34 | 146.69 | 96.45 | 100.27 | 108.66 | -3.51E-01 | 1.48E-03 | 3.19E-02 |
| *SALL2* | 44.06 | 33.88 | 34.84 | 60.10 | 51.66 | 49.33 | 5.17E-01 | 1.49E-03 | 3.19E-02 |
| *ZNF491* | 20.08 | 14.58 | 15.72 | 25.01 | 27.00 | 25.69 | 5.19E-01 | 1.49E-03 | 3.20E-02 |
| *SELENOS* | 683.36 | 828.28 | 775.21 | 575.64 | 621.87 | 545.37 | -3.18E-01 | 1.50E-03 | 3.20E-02 |
| *CSRNP2* | 75.83 | 94.43 | 79.60 | 56.56 | 64.34 | 56.81 | -4.15E-01 | 1.50E-03 | 3.21E-02 |
| *CPNE3* | 244.33 | 208.56 | 208.74 | 242.12 | 271.38 | 273.34 | 3.94E-01 | 1.51E-03 | 3.21E-02 |
| *LINC00293* | 20.27 | 19.20 | 13.47 | 31.07 | 26.12 | 28.18 | 6.08E-01 | 1.51E-03 | 3.21E-02 |
| *SUSD5* | 12.81 | 13.40 | 12.94 | 9.76 | 9.57 | 10.64 | -4.73E-01 | 1.52E-03 | 3.24E-02 |
| *XKR8* | 234.70 | 191.60 | 201.52 | 266.36 | 241.93 | 254.83 | 4.17E-01 | 1.52E-03 | 3.24E-02 |
| *EHBP1* | 156.16 | 183.11 | 188.63 | 118.45 | 132.65 | 125.90 | -3.46E-01 | 1.53E-03 | 3.25E-02 |
| *TRIB1* | 210.89 | 270.14 | 265.10 | 146.20 | 190.93 | 172.04 | -4.17E-01 | 1.54E-03 | 3.27E-02 |
| *RUNX3* | 30.44 | 31.77 | 35.44 | 25.72 | 24.94 | 24.17 | -4.42E-01 | 1.55E-03 | 3.28E-02 |
| *SCAMP5* | 58.09 | 45.61 | 41.22 | 74.15 | 74.10 | 59.08 | 5.62E-01 | 1.55E-03 | 3.28E-02 |
| *TMIGD2* | 99.14 | 98.61 | 95.18 | 105.21 | 122.44 | 122.94 | 3.67E-01 | 1.55E-03 | 3.28E-02 |
| *ZNF721* | 467.24 | 423.52 | 516.86 | 512.26 | 645.59 | 611.80 | 4.14E-01 | 1.55E-03 | 3.28E-02 |
| *TRAPPC3L* | 23.30 | 23.93 | 26.08 | 16.87 | 14.78 | 21.13 | -5.74E-01 | 1.56E-03 | 3.29E-02 |
| *CEACAM4* | 55.47 | 44.10 | 46.42 | 62.24 | 70.06 | 63.01 | 4.63E-01 | 1.57E-03 | 3.31E-02 |
| *DIPK1C* | 40.88 | 38.26 | 45.91 | 57.10 | 51.25 | 61.06 | 4.46E-01 | 1.58E-03 | 3.31E-02 |
| *FCSK* | 147.95 | 180.25 | 181.90 | 116.79 | 120.77 | 126.48 | -3.46E-01 | 1.57E-03 | 3.31E-02 |
| *LINC00052* | 46.54 | 41.13 | 36.72 | 32.61 | 27.44 | 30.09 | -4.95E-01 | 1.57E-03 | 3.31E-02 |
| *SLC34A3* | 290.59 | 179.00 | 228.32 | 341.36 | 318.95 | 294.45 | 6.09E-01 | 1.57E-03 | 3.31E-02 |
| *TDRG1* | 18.90 | 11.90 | 16.11 | 24.87 | 27.57 | 23.64 | 5.92E-01 | 1.57E-03 | 3.31E-02 |
| *ZNF827* | 33.78 | 43.38 | 40.39 | 27.20 | 24.94 | 31.46 | -5.30E-01 | 1.58E-03 | 3.31E-02 |
| *AK9* | 18.94 | 22.43 | 20.50 | 16.97 | 15.01 | 15.42 | -4.83E-01 | 1.59E-03 | 3.32E-02 |
| *C19orf84* | 39.36 | 28.72 | 25.35 | 51.48 | 48.85 | 42.02 | 5.95E-01 | 1.59E-03 | 3.32E-02 |
| *VAPB* | 53.54 | 58.29 | 56.16 | 39.60 | 42.06 | 44.39 | -4.07E-01 | 1.59E-03 | 3.32E-02 |
| *MAGOHB* | 209.08 | 142.94 | 141.60 | 226.66 | 219.00 | 207.27 | 5.64E-01 | 1.60E-03 | 3.33E-02 |
| *RREB1* | 345.15 | 408.45 | 466.62 | 249.89 | 293.39 | 296.08 | -4.08E-01 | 1.61E-03 | 3.35E-02 |
| *MAN1A1* | 104.94 | 89.29 | 66.59 | 118.71 | 111.41 | 117.27 | 5.45E-01 | 1.61E-03 | 3.35E-02 |
| *IL6* | 21.48 | 21.18 | 24.27 | 16.22 | 16.88 | 18.09 | -4.79E-01 | 1.61E-03 | 3.35E-02 |
| *LINC00705* | 26.30 | 18.29 | 20.20 | 36.07 | 34.89 | 29.08 | 5.46E-01 | 1.62E-03 | 3.36E-02 |
| *RAPGEF2* | 49.26 | 65.77 | 64.78 | 39.53 | 43.30 | 43.78 | -4.76E-01 | 1.62E-03 | 3.36E-02 |
| *TBC1D25* | 135.84 | 126.69 | 135.22 | 149.71 | 147.22 | 168.69 | 3.61E-01 | 1.62E-03 | 3.36E-02 |
| *PDZD8* | 236.85 | 236.71 | 229.96 | 258.06 | 256.07 | 282.27 | 3.16E-01 | 1.63E-03 | 3.37E-02 |
| *HSPE1* | 1676.84 | 1403.79 | 1411.67 | 1915.28 | 1857.27 | 1689.13 | 3.15E-01 | 1.63E-03 | 3.37E-02 |
| *SDHD* | 886.44 | 441.94 | 493.32 | 997.62 | 890.60 | 926.90 | 7.86E-01 | 1.63E-03 | 3.37E-02 |
| *ARMT1* | 420.76 | 305.22 | 313.60 | 471.38 | 422.87 | 445.60 | 5.00E-01 | 1.64E-03 | 3.39E-02 |
| *LCE1E* | 55.03 | 29.46 | 36.11 | 64.82 | 60.70 | 59.81 | 6.71E-01 | 1.65E-03 | 3.41E-02 |
| *CSNK1G1* | 14.46 | 19.54 | 23.34 | 13.69 | 12.59 | 11.97 | -6.42E-01 | 1.66E-03 | 3.42E-02 |
| *STYXL1* | 130.63 | 156.10 | 167.07 | 115.01 | 97.81 | 97.06 | -4.17E-01 | 1.67E-03 | 3.43E-02 |
| *H2AC17* | 98.98 | 95.11 | 89.19 | 110.17 | 107.95 | 116.73 | 3.54E-01 | 1.67E-03 | 3.44E-02 |
| *ADAMTS3* | 7.14 | 8.28 | 9.97 | 4.77 | 6.51 | 6.19 | -5.94E-01 | 1.68E-03 | 3.44E-02 |
| *H2BC21* | 175.31 | 202.05 | 181.35 | 219.22 | 195.56 | 258.68 | 3.96E-01 | 1.68E-03 | 3.44E-02 |
| *SPRED1* | 78.68 | 56.01 | 51.70 | 36.36 | 39.33 | 43.14 | -6.19E-01 | 1.68E-03 | 3.44E-02 |
| *SRPK1* | 93.30 | 88.15 | 69.33 | 118.86 | 102.12 | 102.47 | 4.80E-01 | 1.68E-03 | 3.44E-02 |
| *CTBP1* | 40.80 | 33.23 | 34.11 | 53.63 | 49.89 | 46.25 | 4.59E-01 | 1.69E-03 | 3.45E-02 |
| *LOC101928881* | 56.14 | 50.49 | 57.62 | 69.84 | 70.54 | 67.65 | 3.91E-01 | 1.69E-03 | 3.45E-02 |
| *PITPNA-AS1* | 307.99 | 258.88 | 274.67 | 311.35 | 338.11 | 347.22 | 3.73E-01 | 1.69E-03 | 3.45E-02 |
| *RETSAT* | 190.17 | 235.24 | 202.91 | 139.77 | 152.32 | 160.28 | -3.37E-01 | 1.69E-03 | 3.45E-02 |
| *ERN2* | 68.86 | 55.62 | 70.08 | 98.78 | 79.10 | 82.36 | 4.96E-01 | 1.70E-03 | 3.46E-02 |
| *GNAI2* | 225.74 | 229.70 | 213.77 | 264.64 | 270.08 | 236.81 | 3.37E-01 | 1.70E-03 | 3.46E-02 |
| *NLRP1* | 31.04 | 20.78 | 22.72 | 43.16 | 34.78 | 36.95 | 5.71E-01 | 1.71E-03 | 3.46E-02 |
| *PDE1A* | 479.41 | 552.52 | 514.35 | 655.00 | 576.84 | 568.19 | 3.07E-01 | 1.70E-03 | 3.46E-02 |
| *TRPM6* | 31.53 | 24.76 | 32.20 | 43.53 | 41.20 | 40.91 | 4.67E-01 | 1.71E-03 | 3.46E-02 |
| *SLC17A6* | 4.20 | 3.71 | 3.60 | 5.84 | 5.56 | 5.25 | 4.16E-01 | 1.71E-03 | 3.46E-02 |
| *FIGNL1* | 129.74 | 170.62 | 173.93 | 113.24 | 94.81 | 109.92 | -4.34E-01 | 1.71E-03 | 3.46E-02 |
| *LINC-PINT* | 68.04 | 79.67 | 81.27 | 57.72 | 42.89 | 56.59 | -4.95E-01 | 1.71E-03 | 3.46E-02 |
| *ANKRD29* | 6.65 | 7.24 | 6.11 | 4.74 | 4.68 | 5.49 | -4.72E-01 | 1.72E-03 | 3.46E-02 |
| *TTC30B* | 33.12 | 31.59 | 27.08 | 25.47 | 21.24 | 21.90 | -4.85E-01 | 1.71E-03 | 3.46E-02 |
| *MRPL16* | 581.82 | 685.94 | 674.71 | 480.75 | 525.36 | 478.13 | -3.02E-01 | 1.73E-03 | 3.48E-02 |
| *NFIL3* | 232.06 | 323.55 | 321.10 | 179.48 | 209.05 | 202.91 | -4.22E-01 | 1.74E-03 | 3.50E-02 |
| *DPH6* | 3.59 | 4.90 | 2.80 | 2.15 | 2.40 | 2.39 | -5.94E-01 | 1.75E-03 | 3.52E-02 |
| *MTERF1* | 55.55 | 66.08 | 66.23 | 47.14 | 46.64 | 45.17 | -3.98E-01 | 1.75E-03 | 3.52E-02 |
| *ZDHHC6* | 598.71 | 720.60 | 649.27 | 538.34 | 488.03 | 475.22 | -3.10E-01 | 1.76E-03 | 3.54E-02 |
| *ERGIC1* | 376.03 | 395.27 | 439.92 | 263.51 | 333.60 | 269.43 | -3.73E-01 | 1.77E-03 | 3.54E-02 |
| *GLRB* | 36.22 | 38.00 | 44.92 | 29.29 | 30.72 | 28.88 | -4.52E-01 | 1.77E-03 | 3.54E-02 |
| *TMEM260* | 69.65 | 76.27 | 85.29 | 58.08 | 56.59 | 52.60 | -4.05E-01 | 1.77E-03 | 3.54E-02 |
| *ADGRE2* | 23.69 | 32.39 | 29.71 | 21.71 | 20.25 | 20.75 | -5.16E-01 | 1.78E-03 | 3.54E-02 |
| *CDH22* | 60.37 | 48.68 | 61.21 | 80.56 | 74.41 | 68.98 | 4.59E-01 | 1.78E-03 | 3.54E-02 |
| *LRRC73* | 15.36 | 22.43 | 19.59 | 13.22 | 12.65 | 14.17 | -5.93E-01 | 1.78E-03 | 3.54E-02 |
| *LSS* | 397.98 | 449.04 | 467.77 | 456.10 | 564.77 | 535.94 | 3.44E-01 | 1.78E-03 | 3.54E-02 |
| *MCL1* | 44.10 | 51.43 | 51.52 | 36.46 | 34.49 | 39.10 | -4.27E-01 | 1.78E-03 | 3.54E-02 |
| *CCL28* | 3.30 | 2.73 | 3.40 | 4.95 | 4.42 | 4.25 | 4.32E-01 | 1.79E-03 | 3.56E-02 |
| *RTBDN* | 23.27 | 20.87 | 19.56 | 37.03 | 32.08 | 27.46 | 5.04E-01 | 1.79E-03 | 3.56E-02 |
| *LINC00467* | 77.23 | 64.44 | 68.10 | 53.94 | 48.92 | 47.44 | -4.36E-01 | 1.80E-03 | 3.57E-02 |
| *CYLD* | 60.00 | 78.49 | 70.73 | 51.83 | 45.93 | 52.56 | -4.30E-01 | 1.82E-03 | 3.57E-02 |
| *FAM111B* | 157.70 | 172.53 | 156.03 | 208.19 | 183.10 | 176.48 | 3.58E-01 | 1.82E-03 | 3.57E-02 |
| *FLNB* | 130.49 | 175.95 | 159.16 | 99.29 | 113.45 | 111.44 | -3.82E-01 | 1.81E-03 | 3.57E-02 |
| *LOC101929897* | 60.63 | 39.03 | 38.52 | 68.10 | 64.60 | 67.29 | 5.84E-01 | 1.82E-03 | 3.57E-02 |
| *LRCH4* | 87.30 | 84.17 | 77.21 | 112.78 | 102.87 | 93.12 | 4.18E-01 | 1.81E-03 | 3.57E-02 |
| *NUP153* | 53.74 | 55.74 | 51.74 | 39.14 | 41.77 | 41.48 | -3.93E-01 | 1.82E-03 | 3.57E-02 |
| *POFUT2* | 33.09 | 26.26 | 30.07 | 42.73 | 40.32 | 41.94 | 4.44E-01 | 1.82E-03 | 3.57E-02 |
| *TUBGCP3* | 66.29 | 53.52 | 59.75 | 73.09 | 79.61 | 76.82 | 4.20E-01 | 1.81E-03 | 3.57E-02 |
| *ADAM1A* | 27.30 | 19.93 | 19.40 | 15.46 | 14.02 | 17.71 | -5.85E-01 | 1.84E-03 | 3.58E-02 |
| *CXCR1* | 57.50 | 46.13 | 41.49 | 64.73 | 64.63 | 66.29 | 4.72E-01 | 1.83E-03 | 3.58E-02 |
| *GPCPD1* | 79.22 | 88.51 | 91.22 | 104.30 | 94.99 | 117.49 | 3.92E-01 | 1.83E-03 | 3.58E-02 |
| *LOC100506858* | 31.45 | 25.67 | 22.27 | 39.55 | 38.92 | 37.53 | 4.98E-01 | 1.83E-03 | 3.58E-02 |
| *LRRC41* | 90.85 | 103.22 | 97.75 | 68.75 | 69.83 | 76.47 | -3.52E-01 | 1.84E-03 | 3.58E-02 |
| *MRGPRX2* | 14.47 | 12.94 | 13.04 | 21.90 | 18.88 | 19.76 | 4.54E-01 | 1.84E-03 | 3.58E-02 |
| *RNF185-AS1* | 27.13 | 18.57 | 24.10 | 39.46 | 31.28 | 37.29 | 5.59E-01 | 1.84E-03 | 3.58E-02 |
| *RWDD2B* | 195.71 | 201.51 | 211.48 | 236.85 | 220.53 | 235.56 | 3.15E-01 | 1.83E-03 | 3.58E-02 |
| *SPC25* | 228.55 | 210.44 | 201.44 | 267.20 | 265.55 | 228.83 | 3.82E-01 | 1.84E-03 | 3.58E-02 |
| *SPINT3* | 5.58 | 4.06 | 4.31 | 8.18 | 6.78 | 6.82 | 5.15E-01 | 1.83E-03 | 3.58E-02 |
| *TST* | 364.87 | 442.71 | 471.16 | 293.67 | 301.06 | 323.21 | -3.52E-01 | 1.84E-03 | 3.58E-02 |
| *SPOCK1* | 13.60 | 15.28 | 16.80 | 10.64 | 11.97 | 11.81 | -5.05E-01 | 1.84E-03 | 3.58E-02 |
| *EPHX4* | 35.94 | 30.47 | 31.54 | 22.69 | 24.86 | 26.10 | -4.74E-01 | 1.85E-03 | 3.58E-02 |
| *TIGAR* | 274.66 | 331.46 | 313.51 | 349.61 | 376.52 | 323.33 | 3.17E-01 | 1.85E-03 | 3.59E-02 |
| *SIRT4* | 20.26 | 21.74 | 26.26 | 18.67 | 13.58 | 16.94 | -5.64E-01 | 1.85E-03 | 3.59E-02 |
| *PAQR4* | 121.93 | 134.45 | 111.18 | 149.48 | 130.50 | 169.06 | 4.14E-01 | 1.86E-03 | 3.59E-02 |
| *COX16* | 1290.54 | 1345.46 | 1263.82 | 1771.94 | 1508.88 | 1433.79 | 3.03E-01 | 1.86E-03 | 3.60E-02 |
| *TIMM17A* | 853.89 | 740.44 | 706.76 | 994.34 | 938.84 | 860.02 | 3.41E-01 | 1.87E-03 | 3.60E-02 |
| *TMEM120B* | 61.67 | 89.94 | 79.23 | 49.41 | 48.94 | 57.96 | -4.92E-01 | 1.87E-03 | 3.60E-02 |
| *RSPO3* | 11.34 | 15.70 | 11.01 | 8.12 | 7.24 | 10.10 | -6.56E-01 | 1.88E-03 | 3.61E-02 |
| *IFNA14* | 12.85 | 10.72 | 8.97 | 17.36 | 17.22 | 16.29 | 5.29E-01 | 1.89E-03 | 3.64E-02 |
| *SERINC5* | 62.04 | 57.36 | 51.98 | 44.47 | 40.29 | 41.46 | -4.25E-01 | 1.90E-03 | 3.65E-02 |
| *CDK5R1* | 9.57 | 9.26 | 8.26 | 6.81 | 6.27 | 7.48 | -4.74E-01 | 1.90E-03 | 3.65E-02 |
| *TFCP2L1* | 29.91 | 20.57 | 20.94 | 38.82 | 32.73 | 38.97 | 5.64E-01 | 1.91E-03 | 3.67E-02 |
| *LINC00473* | 425.94 | 525.73 | 474.94 | 361.86 | 333.42 | 367.91 | -3.15E-01 | 1.92E-03 | 3.67E-02 |
| *NBN* | 329.44 | 369.18 | 391.99 | 263.45 | 280.17 | 263.26 | -3.07E-01 | 1.93E-03 | 3.67E-02 |
| *RNF146* | 41.06 | 44.84 | 47.95 | 34.78 | 26.08 | 35.04 | -5.13E-01 | 1.93E-03 | 3.67E-02 |
| *SLC6A6* | 14.47 | 19.67 | 13.46 | 12.09 | 10.71 | 10.34 | -5.89E-01 | 1.93E-03 | 3.67E-02 |
| *ERMAP* | 42.20 | 48.16 | 60.14 | 33.87 | 28.97 | 39.40 | -5.69E-01 | 1.94E-03 | 3.69E-02 |
| *MMGT1* | 360.27 | 425.95 | 426.77 | 300.27 | 312.31 | 290.83 | -3.05E-01 | 1.94E-03 | 3.69E-02 |
| *ZNF624* | 6.54 | 6.75 | 6.33 | 4.61 | 4.24 | 5.61 | -4.82E-01 | 1.94E-03 | 3.69E-02 |
| *ACTRT3* | 24.49 | 36.22 | 25.43 | 22.48 | 18.42 | 18.72 | -5.87E-01 | 1.95E-03 | 3.70E-02 |
| *STARD13-AS* | 49.84 | 40.83 | 45.54 | 64.87 | 60.72 | 55.96 | 4.37E-01 | 1.95E-03 | 3.70E-02 |
| *ALDH1A3* | 61.78 | 81.60 | 90.49 | 50.73 | 48.48 | 57.77 | -5.08E-01 | 1.96E-03 | 3.71E-02 |
| *GSEC* | 87.60 | 110.33 | 101.58 | 76.70 | 59.84 | 71.20 | -4.34E-01 | 1.96E-03 | 3.72E-02 |
| *HNRNPLL* | 18.09 | 28.26 | 22.55 | 14.16 | 17.50 | 15.03 | -6.25E-01 | 1.97E-03 | 3.72E-02 |
| *GDI1* | 239.21 | 232.59 | 236.83 | 257.20 | 257.63 | 290.46 | 3.22E-01 | 1.97E-03 | 3.73E-02 |
| *C22orf42* | 12.15 | 9.31 | 9.19 | 18.39 | 13.91 | 17.11 | 5.54E-01 | 1.98E-03 | 3.73E-02 |
| *CCNJ* | 40.77 | 41.94 | 43.10 | 24.99 | 33.95 | 31.85 | -5.14E-01 | 1.98E-03 | 3.73E-02 |
| *GDA* | 189.84 | 219.45 | 209.35 | 148.90 | 143.06 | 160.77 | -3.12E-01 | 1.98E-03 | 3.73E-02 |
| *ACADM* | 675.10 | 424.30 | 488.64 | 809.97 | 657.32 | 734.91 | 5.82E-01 | 1.99E-03 | 3.74E-02 |
| *GRAMD1B* | 426.32 | 447.93 | 468.94 | 328.52 | 309.03 | 362.13 | -3.12E-01 | 2.00E-03 | 3.74E-02 |
| *MAMDC2* | 226.19 | 220.77 | 243.66 | 258.70 | 371.83 | 260.94 | 4.71E-01 | 1.99E-03 | 3.74E-02 |
| *NUP210* | 122.94 | 147.37 | 142.11 | 100.40 | 98.15 | 101.60 | -3.26E-01 | 2.00E-03 | 3.74E-02 |
| *ZNF426* | 38.42 | 39.87 | 38.90 | 28.95 | 31.26 | 30.31 | -4.10E-01 | 2.00E-03 | 3.74E-02 |
| *LSR* | 24.91 | 36.61 | 25.75 | 19.49 | 17.23 | 22.44 | -6.22E-01 | 2.00E-03 | 3.75E-02 |
| *SLC20A1* | 1123.60 | 1292.48 | 1333.37 | 885.10 | 1004.66 | 1016.91 | -3.17E-01 | 2.00E-03 | 3.75E-02 |
| *LINC00165* | 140.82 | 201.59 | 179.08 | 118.26 | 121.36 | 116.56 | -3.95E-01 | 2.01E-03 | 3.75E-02 |
| *INPP4A* | 59.13 | 78.60 | 79.77 | 46.60 | 51.78 | 53.53 | -4.58E-01 | 2.02E-03 | 3.77E-02 |
| *SPATA24* | 223.40 | 147.44 | 159.13 | 241.33 | 230.36 | 227.44 | 5.56E-01 | 2.02E-03 | 3.77E-02 |
| *C11orf72* | 77.94 | 45.92 | 54.36 | 85.93 | 87.47 | 79.21 | 5.98E-01 | 2.02E-03 | 3.77E-02 |
| *MAPK1* | 56.26 | 63.80 | 54.65 | 32.95 | 45.90 | 43.25 | -5.18E-01 | 2.03E-03 | 3.77E-02 |
| *OR51E1* | 10.65 | 10.34 | 8.83 | 18.49 | 14.35 | 14.08 | 5.16E-01 | 2.03E-03 | 3.77E-02 |
| *SEC24A* | 428.38 | 338.75 | 321.48 | 202.70 | 260.12 | 245.47 | -4.89E-01 | 2.03E-03 | 3.77E-02 |
| *CDSN* | 25.61 | 18.45 | 19.91 | 33.14 | 30.80 | 30.71 | 4.89E-01 | 2.04E-03 | 3.78E-02 |
| *MST1R* | 124.10 | 149.56 | 135.17 | 97.04 | 93.78 | 105.56 | -3.36E-01 | 2.04E-03 | 3.78E-02 |
| *ZNHIT6* | 360.09 | 351.00 | 356.10 | 239.50 | 291.27 | 247.44 | -3.35E-01 | 2.04E-03 | 3.79E-02 |
| *BIN3* | 66.61 | 56.27 | 53.01 | 76.93 | 71.61 | 75.32 | 4.18E-01 | 2.05E-03 | 3.79E-02 |
| *CIPC* | 94.82 | 120.60 | 99.82 | 78.19 | 76.40 | 70.70 | -3.77E-01 | 2.06E-03 | 3.79E-02 |
| *GAB4* | 46.20 | 36.45 | 32.21 | 50.89 | 64.52 | 53.11 | 5.53E-01 | 2.06E-03 | 3.79E-02 |
| *KRCC1* | 303.58 | 301.51 | 296.23 | 239.53 | 154.81 | 192.58 | -4.99E-01 | 2.05E-03 | 3.79E-02 |
| *MCOLN3* | 35.94 | 36.04 | 35.36 | 25.11 | 29.54 | 27.21 | -4.38E-01 | 2.05E-03 | 3.79E-02 |
| *KCNJ2* | 34.03 | 36.86 | 36.93 | 30.60 | 25.61 | 26.65 | -4.31E-01 | 2.06E-03 | 3.80E-02 |
| *OSCP1* | 26.67 | 34.00 | 37.55 | 22.71 | 23.87 | 24.24 | -5.22E-01 | 2.07E-03 | 3.80E-02 |
| *ZNF134* | 24.34 | 28.96 | 34.32 | 20.40 | 22.81 | 20.35 | -5.22E-01 | 2.07E-03 | 3.80E-02 |
| *HOXA11-AS* | 48.18 | 65.26 | 56.63 | 45.35 | 31.77 | 39.43 | -5.39E-01 | 2.07E-03 | 3.80E-02 |
| *DMAC2L* | 69.21 | 61.64 | 64.35 | 79.33 | 75.89 | 85.91 | 3.79E-01 | 2.08E-03 | 3.81E-02 |
| *DNM3* | 16.46 | 12.61 | 8.42 | 7.21 | 6.65 | 8.70 | -7.58E-01 | 2.08E-03 | 3.81E-02 |
| *SUSD2* | 174.32 | 166.83 | 147.89 | 111.79 | 111.43 | 121.02 | -3.66E-01 | 2.08E-03 | 3.81E-02 |
| *SYNE1* | 46.60 | 65.40 | 63.01 | 32.48 | 43.39 | 41.78 | -5.61E-01 | 2.08E-03 | 3.81E-02 |
| *NLRP4* | 5.38 | 4.40 | 4.65 | 8.02 | 7.06 | 6.48 | 4.49E-01 | 2.09E-03 | 3.82E-02 |
| *MESD* | 34.62 | 35.27 | 31.87 | 23.82 | 26.17 | 27.55 | -4.51E-01 | 2.10E-03 | 3.83E-02 |
| *PTGIR* | 72.22 | 49.05 | 47.83 | 83.84 | 73.59 | 76.39 | 5.53E-01 | 2.10E-03 | 3.83E-02 |
| *NUP58* | 107.74 | 114.05 | 120.27 | 83.44 | 83.20 | 85.36 | -3.27E-01 | 2.11E-03 | 3.84E-02 |
| *DOCK9-DT* | 35.24 | 34.09 | 33.61 | 51.16 | 48.78 | 41.53 | 4.35E-01 | 2.11E-03 | 3.84E-02 |
| *DTX3L* | 79.17 | 80.93 | 77.51 | 50.14 | 61.30 | 60.57 | -4.08E-01 | 2.11E-03 | 3.84E-02 |
| *EPHB2* | 67.20 | 48.14 | 51.15 | 72.23 | 81.47 | 70.42 | 4.98E-01 | 2.12E-03 | 3.84E-02 |
| *PAGE2B* | 3.90 | 4.87 | 4.41 | 3.40 | 3.38 | 3.12 | -4.10E-01 | 2.11E-03 | 3.84E-02 |
| *GABARAPL1* | 159.10 | 177.16 | 168.20 | 196.41 | 186.89 | 183.69 | 3.01E-01 | 2.13E-03 | 3.84E-02 |
| *HDGFL1* | 48.09 | 30.88 | 40.14 | 57.75 | 58.79 | 53.96 | 5.32E-01 | 2.12E-03 | 3.84E-02 |
| *MAP1LC3A* | 63.08 | 52.12 | 54.65 | 71.84 | 71.84 | 71.71 | 3.99E-01 | 2.13E-03 | 3.84E-02 |
| *MED1* | 126.15 | 158.63 | 140.37 | 92.04 | 108.47 | 103.77 | -3.57E-01 | 2.14E-03 | 3.84E-02 |
| *SCML1* | 717.77 | 607.60 | 640.26 | 844.95 | 763.73 | 732.22 | 3.37E-01 | 2.14E-03 | 3.84E-02 |
| *SCYL2* | 73.86 | 77.16 | 62.70 | 54.27 | 48.94 | 51.85 | -4.10E-01 | 2.14E-03 | 3.84E-02 |
| *ZNF45* | 51.52 | 50.45 | 46.79 | 30.23 | 39.43 | 37.83 | -4.87E-01 | 2.13E-03 | 3.84E-02 |
| *ZNF532* | 200.44 | 260.51 | 266.66 | 168.01 | 164.60 | 177.10 | -3.69E-01 | 2.12E-03 | 3.84E-02 |
| *DPYD* | 53.25 | 38.07 | 36.87 | 71.26 | 53.53 | 63.66 | 5.78E-01 | 2.14E-03 | 3.85E-02 |
| *SERPINI1* | 312.01 | 327.11 | 302.40 | 224.40 | 249.27 | 190.60 | -3.73E-01 | 2.16E-03 | 3.87E-02 |
| *CCDC142* | 27.48 | 33.85 | 28.65 | 22.24 | 21.88 | 24.04 | -4.69E-01 | 2.17E-03 | 3.88E-02 |
| *TM7SF3* | 70.17 | 76.20 | 73.30 | 51.60 | 56.99 | 56.27 | -3.62E-01 | 2.17E-03 | 3.88E-02 |
| *H2AC6* | 342.85 | 386.71 | 371.62 | 481.27 | 379.96 | 453.84 | 3.58E-01 | 2.17E-03 | 3.88E-02 |
| *OR7E12P* | 60.44 | 50.11 | 44.68 | 74.10 | 64.50 | 68.27 | 4.68E-01 | 2.18E-03 | 3.89E-02 |
| *GALR3* | 102.94 | 78.58 | 67.66 | 126.16 | 111.64 | 100.94 | 5.61E-01 | 2.18E-03 | 3.90E-02 |
| *ZNF527* | 51.74 | 40.09 | 44.62 | 69.41 | 59.15 | 57.69 | 4.70E-01 | 2.19E-03 | 3.90E-02 |
| *ADARB2-AS1* | 26.00 | 19.89 | 21.88 | 34.38 | 34.65 | 29.83 | 4.66E-01 | 2.21E-03 | 3.91E-02 |
| *ALDH3B1* | 136.59 | 102.06 | 120.27 | 146.94 | 142.70 | 153.17 | 4.41E-01 | 2.21E-03 | 3.91E-02 |
| *CDH15* | 35.53 | 27.69 | 37.46 | 51.64 | 50.09 | 42.22 | 5.00E-01 | 2.21E-03 | 3.91E-02 |
| *CDKN2D* | 46.53 | 50.02 | 55.11 | 65.32 | 63.54 | 63.15 | 3.77E-01 | 2.21E-03 | 3.91E-02 |
| *CEP120* | 12.54 | 13.96 | 11.70 | 11.15 | 8.73 | 8.87 | -5.02E-01 | 2.21E-03 | 3.91E-02 |
| *HSPB11* | 601.44 | 550.22 | 524.94 | 708.02 | 647.58 | 623.24 | 3.21E-01 | 2.21E-03 | 3.91E-02 |
| *LINC01806* | 37.91 | 39.57 | 34.99 | 30.42 | 23.19 | 29.47 | -4.87E-01 | 2.21E-03 | 3.91E-02 |
| *SEMA5B* | 70.19 | 45.88 | 50.56 | 78.90 | 71.99 | 76.39 | 5.27E-01 | 2.21E-03 | 3.91E-02 |
| *TYMP* | 67.02 | 60.28 | 60.79 | 79.90 | 80.34 | 72.71 | 3.80E-01 | 2.22E-03 | 3.91E-02 |
| *GDAP1* | 61.44 | 75.42 | 65.54 | 84.53 | 76.15 | 87.79 | 3.79E-01 | 2.22E-03 | 3.92E-02 |
| *IFT57* | 126.98 | 114.67 | 122.78 | 90.33 | 82.75 | 87.69 | -3.65E-01 | 2.22E-03 | 3.92E-02 |
| *LOC101928896* | 27.05 | 21.36 | 19.40 | 36.48 | 32.18 | 32.18 | 4.97E-01 | 2.23E-03 | 3.93E-02 |
| *NBDY* | 75.27 | 59.40 | 52.01 | 97.18 | 74.10 | 85.80 | 5.45E-01 | 2.23E-03 | 3.93E-02 |
| *TOGARAM1* | 170.26 | 165.16 | 137.56 | 99.41 | 100.46 | 121.53 | -4.25E-01 | 2.24E-03 | 3.94E-02 |
| *UBL7-AS1* | 47.98 | 49.27 | 48.33 | 39.13 | 36.42 | 36.76 | -3.80E-01 | 2.24E-03 | 3.94E-02 |
| *DLX4* | 86.30 | 97.27 | 85.74 | 101.44 | 101.10 | 125.77 | 3.88E-01 | 2.25E-03 | 3.94E-02 |
| *ELP2* | 192.27 | 244.72 | 237.33 | 161.34 | 141.44 | 171.71 | -3.72E-01 | 2.24E-03 | 3.94E-02 |
| *MLPH* | 63.37 | 37.38 | 45.23 | 75.72 | 72.11 | 63.61 | 6.04E-01 | 2.24E-03 | 3.94E-02 |
| *NSUN6* | 22.68 | 25.58 | 23.54 | 17.38 | 19.02 | 19.30 | -4.53E-01 | 2.25E-03 | 3.94E-02 |
| *GFRA4* | 18.80 | 14.25 | 14.25 | 25.48 | 27.15 | 21.02 | 5.27E-01 | 2.25E-03 | 3.94E-02 |
| *TAT* | 15.28 | 12.77 | 13.47 | 22.57 | 19.36 | 20.00 | 4.50E-01 | 2.26E-03 | 3.95E-02 |
| *NECAP1* | 240.88 | 210.40 | 211.63 | 261.40 | 255.44 | 251.35 | 3.50E-01 | 2.27E-03 | 3.96E-02 |
| *NUDT16* | 35.27 | 27.93 | 28.52 | 22.93 | 22.14 | 24.10 | -4.72E-01 | 2.27E-03 | 3.96E-02 |
| *MCM3* | 1567.44 | 1751.97 | 1653.27 | 1294.72 | 1188.76 | 1457.60 | -3.14E-01 | 2.28E-03 | 3.97E-02 |
| *OPHN1* | 723.43 | 613.55 | 614.41 | 747.90 | 755.79 | 929.10 | 3.95E-01 | 2.28E-03 | 3.97E-02 |
| *TFCP2* | 121.17 | 118.91 | 113.87 | 80.05 | 90.17 | 88.66 | -3.47E-01 | 2.28E-03 | 3.97E-02 |
| *ZNF385A* | 32.09 | 30.07 | 31.10 | 45.06 | 42.75 | 39.26 | 4.06E-01 | 2.28E-03 | 3.97E-02 |
| *ZNF582* | 49.04 | 34.29 | 39.09 | 57.24 | 54.21 | 57.79 | 4.80E-01 | 2.28E-03 | 3.97E-02 |
| *NUDT21* | 469.59 | 594.02 | 502.52 | 394.00 | 402.03 | 378.58 | -3.12E-01 | 2.29E-03 | 3.97E-02 |
| *DPYSL3* | 21.63 | 23.30 | 27.26 | 17.71 | 14.07 | 20.12 | -5.68E-01 | 2.30E-03 | 3.98E-02 |
| *EIF4ENIF1* | 195.04 | 239.10 | 236.85 | 146.57 | 163.91 | 172.93 | -3.36E-01 | 2.30E-03 | 3.99E-02 |
| *TSPYL2* | 185.52 | 161.00 | 172.40 | 188.40 | 204.50 | 214.77 | 3.62E-01 | 2.30E-03 | 3.99E-02 |
| *CHD2* | 166.47 | 171.27 | 149.11 | 120.56 | 112.10 | 116.13 | -3.40E-01 | 2.32E-03 | 4.01E-02 |
| *NT5C1A* | 42.47 | 25.77 | 30.44 | 49.59 | 49.16 | 47.44 | 5.65E-01 | 2.32E-03 | 4.01E-02 |
| *PARP4* | 373.66 | 464.07 | 428.29 | 325.89 | 305.16 | 313.31 | -3.03E-01 | 2.32E-03 | 4.01E-02 |
| *ANGPT4* | 32.71 | 25.77 | 28.13 | 46.27 | 38.95 | 38.27 | 4.70E-01 | 2.33E-03 | 4.02E-02 |
| *HGH1* | 142.13 | 151.60 | 150.27 | 169.55 | 169.33 | 159.25 | 3.04E-01 | 2.33E-03 | 4.02E-02 |
| *OLR1* | 339.82 | 286.46 | 321.47 | 399.46 | 467.45 | 334.15 | 4.46E-01 | 2.33E-03 | 4.02E-02 |
| *OSMR* | 379.08 | 475.28 | 455.53 | 306.02 | 343.44 | 318.36 | -3.15E-01 | 2.34E-03 | 4.02E-02 |
| *LTBP3* | 39.48 | 48.43 | 48.83 | 31.82 | 28.94 | 37.58 | -4.94E-01 | 2.35E-03 | 4.03E-02 |
| *TSACC* | 49.90 | 51.53 | 45.72 | 63.97 | 63.06 | 59.48 | 3.75E-01 | 2.35E-03 | 4.04E-02 |
| *CACNB1* | 16.73 | 12.63 | 13.81 | 24.24 | 20.11 | 21.59 | 4.87E-01 | 2.36E-03 | 4.04E-02 |
| *FAN1* | 108.56 | 126.59 | 115.67 | 83.31 | 87.45 | 89.44 | -3.20E-01 | 2.36E-03 | 4.05E-02 |
| *C5orf22* | 87.81 | 104.06 | 90.91 | 70.71 | 71.94 | 63.66 | -3.61E-01 | 2.37E-03 | 4.05E-02 |
| *SLC6A19* | 61.63 | 40.03 | 51.60 | 70.30 | 72.84 | 66.82 | 5.16E-01 | 2.37E-03 | 4.05E-02 |
| *RNF144A-AS1* | 28.94 | 24.21 | 17.27 | 36.60 | 38.39 | 34.20 | 5.86E-01 | 2.38E-03 | 4.07E-02 |
| *ARID1B* | 125.04 | 111.46 | 98.27 | 130.43 | 131.64 | 138.77 | 3.86E-01 | 2.39E-03 | 4.08E-02 |
| *GNA12* | 58.68 | 74.08 | 66.33 | 48.39 | 48.24 | 51.22 | -3.87E-01 | 2.39E-03 | 4.08E-02 |
| *ENKD1* | 49.91 | 67.56 | 64.58 | 42.29 | 44.40 | 44.40 | -4.38E-01 | 2.41E-03 | 4.09E-02 |
| *ALYREF* | 1096.12 | 970.11 | 1032.82 | 1196.27 | 1420.19 | 1143.67 | 3.25E-01 | 2.41E-03 | 4.10E-02 |
| *MLX* | 42.27 | 33.94 | 37.46 | 55.77 | 49.94 | 48.60 | 4.32E-01 | 2.41E-03 | 4.10E-02 |
| *RNF139-AS1* | 15.16 | 21.14 | 19.18 | 11.04 | 13.34 | 14.45 | -6.00E-01 | 2.41E-03 | 4.10E-02 |
| *LLGL1* | 55.14 | 37.68 | 40.59 | 66.31 | 60.15 | 59.39 | 5.08E-01 | 2.42E-03 | 4.10E-02 |
| *MON2* | 179.77 | 195.57 | 162.87 | 113.63 | 123.04 | 142.65 | -3.70E-01 | 2.42E-03 | 4.11E-02 |
| *NAA30* | 288.25 | 267.94 | 248.30 | 198.61 | 191.08 | 194.41 | -3.29E-01 | 2.43E-03 | 4.12E-02 |
| *STX1B* | 54.49 | 40.78 | 44.18 | 68.99 | 64.93 | 56.83 | 4.80E-01 | 2.43E-03 | 4.12E-02 |
| *SLC35E1* | 340.61 | 313.42 | 380.63 | 351.29 | 484.37 | 482.30 | 4.60E-01 | 2.44E-03 | 4.12E-02 |
| *HHLA3* | 70.50 | 82.37 | 78.28 | 56.65 | 51.37 | 61.34 | -3.81E-01 | 2.44E-03 | 4.13E-02 |
| *LUC7L* | 3.79 | 5.94 | 5.08 | 3.74 | 3.41 | 2.87 | -5.47E-01 | 2.46E-03 | 4.14E-02 |
| *MBNL2* | 37.77 | 45.35 | 35.02 | 29.64 | 28.51 | 29.84 | -4.47E-01 | 2.46E-03 | 4.14E-02 |
| *TPRA1* | 77.03 | 94.88 | 81.27 | 62.20 | 59.10 | 64.21 | -3.64E-01 | 2.46E-03 | 4.14E-02 |
| *TPD52L3* | 14.54 | 11.53 | 14.22 | 20.67 | 19.48 | 19.63 | 4.51E-01 | 2.47E-03 | 4.15E-02 |
| *ATXN1-AS1* | 15.46 | 19.47 | 16.84 | 14.61 | 12.42 | 12.16 | -4.96E-01 | 2.48E-03 | 4.16E-02 |
| *LIAS* | 128.61 | 163.70 | 183.24 | 97.48 | 107.74 | 116.25 | -4.20E-01 | 2.48E-03 | 4.16E-02 |
| *PODXL2* | 62.19 | 78.32 | 75.28 | 51.97 | 50.50 | 55.89 | -3.89E-01 | 2.49E-03 | 4.18E-02 |
| *STMN2* | 7.82 | 8.68 | 8.40 | 11.81 | 13.31 | 12.04 | 4.44E-01 | 2.49E-03 | 4.18E-02 |
| *POLR2K* | 303.94 | 234.86 | 204.78 | 366.08 | 296.36 | 303.56 | 5.20E-01 | 2.50E-03 | 4.18E-02 |
| *CDKN2C* | 1199.55 | 785.84 | 845.97 | 1588.66 | 1094.30 | 1387.27 | 5.73E-01 | 2.50E-03 | 4.18E-02 |
| *AKT3* | 36.86 | 42.25 | 30.80 | 30.80 | 25.13 | 22.40 | -5.31E-01 | 2.51E-03 | 4.19E-02 |
| *RHPN1-AS1* | 41.59 | 34.59 | 37.81 | 50.20 | 50.94 | 50.20 | 3.99E-01 | 2.51E-03 | 4.19E-02 |
| *TCEAL1* | 431.61 | 367.79 | 383.36 | 495.29 | 435.87 | 466.14 | 3.49E-01 | 2.51E-03 | 4.19E-02 |
| *FAM102A* | 89.76 | 124.05 | 109.46 | 77.40 | 68.41 | 79.77 | -4.08E-01 | 2.52E-03 | 4.20E-02 |
| *ZNF283* | 14.53 | 14.55 | 14.55 | 12.41 | 10.68 | 11.62 | -4.34E-01 | 2.52E-03 | 4.20E-02 |
| *FBN1* | 107.23 | 118.50 | 119.35 | 70.20 | 81.16 | 93.70 | -3.97E-01 | 2.52E-03 | 4.20E-02 |
| *LRTM1* | 25.99 | 21.00 | 24.96 | 41.51 | 31.25 | 33.72 | 4.93E-01 | 2.53E-03 | 4.21E-02 |
| *FGGY* | 42.97 | 50.98 | 48.95 | 34.62 | 33.24 | 39.58 | -4.29E-01 | 2.53E-03 | 4.21E-02 |
| *ZNF92* | 5.89 | 6.02 | 5.37 | 4.97 | 4.43 | 3.88 | -4.26E-01 | 2.53E-03 | 4.21E-02 |
| *MED11* | 310.37 | 387.66 | 359.41 | 460.32 | 415.08 | 373.75 | 3.49E-01 | 2.54E-03 | 4.21E-02 |
| *HHAT* | 57.56 | 85.67 | 80.44 | 48.33 | 49.29 | 54.84 | -4.82E-01 | 2.54E-03 | 4.21E-02 |
| *PCGF1* | 132.78 | 117.65 | 138.11 | 153.71 | 153.51 | 147.96 | 3.58E-01 | 2.54E-03 | 4.21E-02 |
| *PPRC1* | 205.04 | 261.59 | 279.61 | 165.05 | 180.21 | 177.10 | -3.67E-01 | 2.54E-03 | 4.21E-02 |
| *LINC01968* | 36.22 | 23.35 | 25.12 | 41.36 | 42.79 | 41.08 | 5.31E-01 | 2.55E-03 | 4.22E-02 |
| *NXNL1* | 100.54 | 65.22 | 86.01 | 119.28 | 101.60 | 115.50 | 5.38E-01 | 2.56E-03 | 4.23E-02 |
| *SLC39A5* | 41.84 | 31.77 | 34.20 | 54.49 | 46.94 | 48.27 | 4.62E-01 | 2.56E-03 | 4.23E-02 |
| *IFI6* | 96.01 | 105.77 | 96.84 | 128.81 | 106.05 | 121.03 | 3.64E-01 | 2.57E-03 | 4.24E-02 |
| *TAB3* | 6.50 | 7.66 | 6.97 | 5.64 | 5.61 | 5.28 | -4.15E-01 | 2.57E-03 | 4.24E-02 |
| *TIMM17B* | 29.00 | 20.19 | 22.47 | 41.76 | 34.72 | 31.89 | 5.33E-01 | 2.57E-03 | 4.24E-02 |
| *ZBTB38* | 79.05 | 87.61 | 76.71 | 62.02 | 60.92 | 57.92 | -3.49E-01 | 2.59E-03 | 4.26E-02 |
| *C9orf152* | 14.52 | 14.50 | 15.91 | 10.19 | 13.85 | 9.27 | -5.38E-01 | 2.60E-03 | 4.27E-02 |
| *RTL9* | 29.46 | 17.04 | 24.82 | 38.10 | 35.92 | 35.81 | 5.83E-01 | 2.60E-03 | 4.27E-02 |
| *TMEM184A* | 61.54 | 76.27 | 69.98 | 51.63 | 50.05 | 53.37 | -3.74E-01 | 2.60E-03 | 4.27E-02 |
| *CCR4* | 37.54 | 32.15 | 37.45 | 52.96 | 48.00 | 44.48 | 4.22E-01 | 2.60E-03 | 4.27E-02 |
| *PKIG* | 133.88 | 139.84 | 122.96 | 162.63 | 152.08 | 143.64 | 3.43E-01 | 2.61E-03 | 4.29E-02 |
| *NRSN2-AS1* | 12.76 | 11.84 | 15.65 | 11.05 | 7.82 | 10.26 | -5.60E-01 | 2.62E-03 | 4.29E-02 |
| *FAM76B* | 240.49 | 133.70 | 124.67 | 221.65 | 239.24 | 268.52 | 7.43E-01 | 2.62E-03 | 4.29E-02 |
| *MAP1A* | 22.05 | 19.60 | 17.73 | 28.67 | 29.94 | 27.08 | 4.36E-01 | 2.62E-03 | 4.29E-02 |
| *ZNF135* | 66.38 | 53.53 | 56.55 | 75.27 | 76.24 | 71.09 | 4.03E-01 | 2.63E-03 | 4.29E-02 |
| *ZNF75D* | 14.30 | 17.23 | 14.12 | 10.45 | 11.40 | 12.33 | -5.04E-01 | 2.65E-03 | 4.32E-02 |
| *PRKAG2-AS1* | 54.81 | 76.58 | 71.29 | 39.05 | 48.29 | 51.94 | -5.13E-01 | 2.65E-03 | 4.32E-02 |
| *ASPSCR1* | 181.94 | 203.37 | 183.70 | 129.44 | 147.36 | 141.24 | -3.04E-01 | 2.67E-03 | 4.34E-02 |
| *PLCD1* | 46.43 | 40.58 | 46.43 | 66.40 | 56.03 | 54.94 | 4.27E-01 | 2.67E-03 | 4.34E-02 |
| *BMPER* | 5.76 | 5.21 | 5.42 | 4.85 | 3.60 | 4.01 | -4.29E-01 | 2.68E-03 | 4.34E-02 |
| *RPL27A* | 543.12 | 454.35 | 441.22 | 593.86 | 541.88 | 601.22 | 3.69E-01 | 2.68E-03 | 4.34E-02 |
| *CENPH* | 168.27 | 154.56 | 160.62 | 184.18 | 176.27 | 192.65 | 3.29E-01 | 2.69E-03 | 4.35E-02 |
| *PPIL6* | 21.40 | 18.04 | 23.93 | 15.14 | 17.74 | 15.36 | -4.84E-01 | 2.69E-03 | 4.36E-02 |
| *TPP1* | 236.51 | 316.79 | 279.80 | 185.60 | 188.60 | 218.18 | -3.55E-01 | 2.70E-03 | 4.36E-02 |
| *CBS* | 117.69 | 134.87 | 120.44 | 78.78 | 95.41 | 95.17 | -3.58E-01 | 2.71E-03 | 4.37E-02 |
| *DLK2* | 149.75 | 140.94 | 144.54 | 177.95 | 162.28 | 160.76 | 3.34E-01 | 2.73E-03 | 4.39E-02 |
| *NUTM2B-AS1* | 82.60 | 106.54 | 100.08 | 113.14 | 114.88 | 110.29 | 3.47E-01 | 2.72E-03 | 4.39E-02 |
| *RSPH1* | 14.10 | 17.59 | 14.71 | 12.55 | 12.29 | 10.65 | -4.82E-01 | 2.72E-03 | 4.39E-02 |
| *ZDHHC12* | 90.50 | 90.93 | 81.28 | 106.77 | 106.29 | 97.83 | 3.48E-01 | 2.72E-03 | 4.39E-02 |
| *ZSCAN12* | 83.50 | 72.81 | 84.35 | 99.16 | 94.86 | 96.10 | 3.65E-01 | 2.73E-03 | 4.39E-02 |
| *ARHGAP32* | 123.76 | 175.88 | 159.94 | 89.82 | 121.27 | 91.84 | -4.76E-01 | 2.73E-03 | 4.39E-02 |
| *MAPK10* | 55.26 | 41.39 | 49.51 | 69.97 | 63.16 | 61.31 | 4.47E-01 | 2.74E-03 | 4.40E-02 |
| *ARF3* | 410.00 | 370.80 | 334.30 | 253.03 | 288.25 | 248.75 | -3.71E-01 | 2.75E-03 | 4.40E-02 |
| *CARHSP1* | 36.74 | 34.12 | 38.88 | 52.39 | 52.79 | 43.66 | 4.24E-01 | 2.75E-03 | 4.40E-02 |
| *PYROXD2* | 71.05 | 104.77 | 75.27 | 60.45 | 54.04 | 56.26 | -4.64E-01 | 2.75E-03 | 4.40E-02 |
| *CRELD2* | 557.05 | 632.33 | 602.74 | 629.20 | 795.95 | 692.43 | 3.11E-01 | 2.76E-03 | 4.42E-02 |
| *DISP2* | 14.57 | 10.94 | 11.22 | 21.20 | 18.28 | 16.89 | 4.89E-01 | 2.76E-03 | 4.42E-02 |
| *TNS3* | 893.86 | 1056.85 | 1106.74 | 746.60 | 828.11 | 798.98 | -3.10E-01 | 2.76E-03 | 4.42E-02 |
| *ERV3-2* | 24.51 | 27.51 | 22.99 | 15.98 | 18.01 | 21.74 | -5.27E-01 | 2.78E-03 | 4.43E-02 |
| *INSL6* | 12.32 | 12.19 | 13.66 | 20.48 | 18.80 | 17.36 | 4.32E-01 | 2.78E-03 | 4.43E-02 |
| *IGF1* | 4.58 | 3.67 | 4.20 | 6.32 | 6.46 | 5.29 | 4.25E-01 | 2.78E-03 | 4.43E-02 |
| *TFEB* | 70.49 | 69.43 | 64.59 | 78.98 | 85.36 | 81.60 | 3.41E-01 | 2.78E-03 | 4.43E-02 |
| *MRVI1-AS1* | 44.62 | 34.37 | 36.88 | 64.80 | 51.18 | 49.22 | 5.06E-01 | 2.79E-03 | 4.43E-02 |
| *CYP3A4* | 97.13 | 77.45 | 94.25 | 96.35 | 125.20 | 123.28 | 4.58E-01 | 2.81E-03 | 4.46E-02 |
| *LINC01537* | 3.10 | 3.29 | 3.43 | 2.68 | 2.34 | 2.55 | -3.46E-01 | 2.81E-03 | 4.47E-02 |
| *ZNF780A* | 29.31 | 25.69 | 23.95 | 21.68 | 20.22 | 20.02 | -4.33E-01 | 2.82E-03 | 4.47E-02 |
| *GNB5* | 49.94 | 39.88 | 40.08 | 58.90 | 62.60 | 53.45 | 4.40E-01 | 2.82E-03 | 4.47E-02 |
| *DESI1* | 189.94 | 183.65 | 180.97 | 191.94 | 234.15 | 213.26 | 3.29E-01 | 2.83E-03 | 4.47E-02 |
| *EPHX2* | 31.38 | 32.80 | 35.54 | 27.15 | 25.36 | 26.05 | -4.01E-01 | 2.83E-03 | 4.47E-02 |
| *PRELID2* | 81.69 | 69.10 | 70.31 | 95.10 | 90.36 | 86.71 | 3.84E-01 | 2.83E-03 | 4.47E-02 |
| *TMEM105* | 79.75 | 62.55 | 69.48 | 92.10 | 85.53 | 87.87 | 4.07E-01 | 2.83E-03 | 4.47E-02 |
| *CDK2AP2* | 128.94 | 153.60 | 137.31 | 144.96 | 173.96 | 158.77 | 3.23E-01 | 2.83E-03 | 4.47E-02 |
| *BHLHB9* | 40.18 | 41.36 | 42.52 | 33.85 | 26.56 | 33.31 | -4.44E-01 | 2.84E-03 | 4.48E-02 |
| *CA7* | 35.27 | 41.28 | 32.03 | 49.83 | 49.94 | 45.70 | 4.22E-01 | 2.84E-03 | 4.48E-02 |
| *FAM189A2* | 24.46 | 17.23 | 19.52 | 31.63 | 30.65 | 28.14 | 4.76E-01 | 2.84E-03 | 4.48E-02 |
| *CSF3R* | 35.31 | 32.43 | 30.30 | 46.48 | 45.08 | 40.97 | 4.07E-01 | 2.85E-03 | 4.48E-02 |
| *MT1HL1* | 1840.83 | 1716.09 | 1868.32 | 1305.00 | 1627.69 | 1395.65 | -3.01E-01 | 2.85E-03 | 4.48E-02 |
| *LOC101928343* | 45.79 | 30.55 | 34.56 | 53.74 | 49.94 | 52.67 | 4.99E-01 | 2.87E-03 | 4.50E-02 |
| *LOC101928697* | 4.30 | 4.79 | 4.75 | 2.67 | 3.49 | 3.85 | -4.69E-01 | 2.87E-03 | 4.50E-02 |
| *NMUR2* | 47.27 | 30.13 | 37.82 | 60.34 | 61.28 | 48.14 | 5.68E-01 | 2.87E-03 | 4.50E-02 |
| *ARHGEF9* | 52.62 | 52.39 | 52.62 | 44.90 | 35.12 | 38.11 | -4.16E-01 | 2.89E-03 | 4.52E-02 |
| *SERPINA9* | 44.60 | 38.99 | 36.62 | 55.18 | 56.83 | 49.09 | 4.21E-01 | 2.90E-03 | 4.53E-02 |
| *WHAMMP2* | 58.81 | 36.18 | 42.89 | 65.79 | 61.77 | 66.20 | 5.35E-01 | 2.91E-03 | 4.54E-02 |
| *SUMO4* | 551.37 | 431.51 | 406.48 | 585.31 | 537.08 | 613.73 | 4.25E-01 | 2.92E-03 | 4.56E-02 |
| *HS2ST1* | 81.81 | 70.37 | 58.15 | 47.20 | 47.34 | 51.94 | -4.75E-01 | 2.92E-03 | 4.56E-02 |
| *EP400* | 181.80 | 229.96 | 229.96 | 131.86 | 145.27 | 171.07 | -3.78E-01 | 2.93E-03 | 4.56E-02 |
| *CSF2RA* | 126.60 | 150.60 | 151.60 | 87.60 | 119.02 | 84.29 | -4.51E-01 | 2.93E-03 | 4.56E-02 |
| *TERF2* | 7.41 | 6.06 | 5.89 | 9.27 | 13.14 | 8.88 | 5.43E-01 | 2.93E-03 | 4.56E-02 |
| *DAPK1* | 296.78 | 343.36 | 326.77 | 221.17 | 263.95 | 225.55 | -3.11E-01 | 2.95E-03 | 4.56E-02 |
| *KIF9* | 36.67 | 24.60 | 23.17 | 41.50 | 43.55 | 40.31 | 5.45E-01 | 2.94E-03 | 4.56E-02 |
| *KTN1* | 1171.29 | 661.46 | 722.64 | 1183.89 | 1148.78 | 1240.81 | 5.91E-01 | 2.95E-03 | 4.56E-02 |
| *NAT2* | 16.18 | 14.02 | 15.35 | 23.92 | 21.76 | 20.79 | 4.17E-01 | 2.95E-03 | 4.56E-02 |
| *TOPORS* | 329.25 | 240.73 | 269.68 | 412.56 | 343.43 | 317.80 | 4.83E-01 | 2.95E-03 | 4.56E-02 |
| *WDR72* | 9.17 | 7.31 | 8.58 | 12.58 | 12.16 | 11.94 | 4.27E-01 | 2.95E-03 | 4.56E-02 |
| *FKBP7* | 6.21 | 4.50 | 6.26 | 4.18 | 4.41 | 3.84 | -4.62E-01 | 2.96E-03 | 4.57E-02 |
| *PRELID3B* | 658.24 | 211.82 | 321.78 | 798.07 | 614.87 | 686.04 | 1.07E+00 | 2.97E-03 | 4.58E-02 |
| *THSD4* | 3.06 | 3.62 | 3.92 | 2.52 | 2.72 | 2.67 | -3.79E-01 | 2.98E-03 | 4.59E-02 |
| *APBA2* | 60.85 | 53.95 | 62.81 | 70.85 | 75.30 | 73.10 | 3.66E-01 | 2.99E-03 | 4.59E-02 |
| *CHST5* | 47.98 | 40.12 | 47.41 | 55.32 | 59.50 | 62.75 | 4.05E-01 | 2.98E-03 | 4.59E-02 |
| *ERO1B* | 7.28 | 8.93 | 8.75 | 6.27 | 6.22 | 6.57 | -4.44E-01 | 3.00E-03 | 4.59E-02 |
| *IL2* | 4.11 | 4.34 | 4.29 | 3.48 | 3.29 | 3.25 | -3.48E-01 | 2.98E-03 | 4.59E-02 |
| *LRPAP1* | 296.11 | 388.78 | 374.06 | 247.26 | 270.18 | 243.85 | -3.42E-01 | 2.99E-03 | 4.59E-02 |
| *PLAC1* | 37.34 | 36.37 | 35.21 | 48.93 | 47.16 | 45.85 | 3.69E-01 | 3.00E-03 | 4.59E-02 |
| *PRDM4* | 175.60 | 216.60 | 224.93 | 132.97 | 153.25 | 155.27 | -3.47E-01 | 2.99E-03 | 4.59E-02 |
| *PRKD1* | 33.33 | 31.06 | 23.34 | 40.13 | 40.38 | 51.57 | 5.46E-01 | 3.04E-03 | 4.63E-02 |
| *TMEM14B* | 2485.28 | 1960.96 | 2008.35 | 2906.98 | 2593.21 | 2518.42 | 3.06E-01 | 3.03E-03 | 4.63E-02 |
| *TXNDC2* | 28.38 | 21.17 | 29.50 | 37.03 | 41.41 | 35.78 | 4.80E-01 | 3.04E-03 | 4.63E-02 |
| *ZMAT4* | 39.23 | 25.96 | 27.94 | 49.17 | 50.79 | 39.31 | 5.63E-01 | 3.04E-03 | 4.63E-02 |
| *B3GAT3* | 74.78 | 82.21 | 89.01 | 58.29 | 59.26 | 64.79 | -3.62E-01 | 3.05E-03 | 4.64E-02 |
| *EMILIN3* | 40.77 | 35.06 | 36.40 | 50.40 | 48.38 | 48.64 | 3.82E-01 | 3.06E-03 | 4.64E-02 |
| *MANBA* | 129.02 | 171.80 | 143.10 | 94.61 | 101.64 | 116.69 | -3.73E-01 | 3.07E-03 | 4.65E-02 |
| *MEIS1* | 42.27 | 50.91 | 45.51 | 55.70 | 59.48 | 62.65 | 3.81E-01 | 3.07E-03 | 4.65E-02 |
| *C12orf75* | 251.72 | 230.95 | 221.44 | 300.62 | 282.49 | 249.17 | 3.59E-01 | 3.09E-03 | 4.67E-02 |
| *NEURL1* | 16.95 | 23.42 | 21.11 | 15.21 | 15.67 | 15.10 | -5.01E-01 | 3.09E-03 | 4.67E-02 |
| *CBX5* | 237.26 | 294.73 | 292.42 | 281.99 | 334.79 | 321.50 | 3.22E-01 | 3.10E-03 | 4.67E-02 |
| *KBTBD11* | 7.55 | 9.68 | 10.33 | 6.49 | 7.34 | 6.63 | -4.94E-01 | 3.10E-03 | 4.67E-02 |
| *YBX2* | 72.27 | 55.27 | 59.22 | 86.74 | 77.27 | 75.19 | 4.35E-01 | 3.10E-03 | 4.67E-02 |
| *STK25* | 130.49 | 132.91 | 112.80 | 135.07 | 144.59 | 153.34 | 3.35E-01 | 3.11E-03 | 4.69E-02 |
| *PCOLCE-AS1* | 62.51 | 59.25 | 49.58 | 81.32 | 71.08 | 67.82 | 4.23E-01 | 3.12E-03 | 4.69E-02 |
| *PADI1* | 87.71 | 81.32 | 80.39 | 108.52 | 96.94 | 94.22 | 3.65E-01 | 3.13E-03 | 4.70E-02 |
| *GPA33* | 21.90 | 18.19 | 18.94 | 29.60 | 27.35 | 26.95 | 4.14E-01 | 3.13E-03 | 4.70E-02 |
| *IHH* | 26.60 | 19.94 | 18.83 | 33.80 | 30.92 | 31.14 | 4.79E-01 | 3.13E-03 | 4.70E-02 |
| *B4GALNT2* | 10.77 | 10.07 | 10.49 | 16.88 | 16.15 | 13.42 | 4.31E-01 | 3.15E-03 | 4.72E-02 |
| *ADIRF* | 33.95 | 40.92 | 39.04 | 47.76 | 48.76 | 58.31 | 4.33E-01 | 3.16E-03 | 4.72E-02 |
| *ISCA2* | 264.28 | 327.07 | 317.97 | 370.28 | 336.59 | 322.37 | 3.14E-01 | 3.16E-03 | 4.72E-02 |
| *ISOC2* | 154.90 | 200.01 | 175.36 | 191.30 | 222.43 | 192.35 | 3.28E-01 | 3.15E-03 | 4.72E-02 |
| *MIS18BP1* | 312.64 | 261.33 | 248.65 | 325.09 | 306.44 | 343.82 | 3.74E-01 | 3.15E-03 | 4.72E-02 |
| *PCNP* | 590.32 | 545.56 | 504.27 | 684.66 | 641.91 | 591.82 | 3.15E-01 | 3.18E-03 | 4.74E-02 |
| *SOCS1* | 18.29 | 11.41 | 11.93 | 24.00 | 20.73 | 21.61 | 5.69E-01 | 3.18E-03 | 4.74E-02 |
| *ELAC1* | 10.29 | 10.47 | 9.94 | 6.10 | 8.54 | 8.13 | -5.12E-01 | 3.20E-03 | 4.76E-02 |
| *PEAR1* | 43.69 | 51.94 | 45.37 | 36.83 | 37.04 | 34.54 | -3.82E-01 | 3.20E-03 | 4.76E-02 |
| *SHISAL1* | 67.34 | 88.82 | 76.55 | 51.64 | 60.78 | 55.48 | -3.99E-01 | 3.19E-03 | 4.76E-02 |
| *FBXO5* | 215.99 | 193.75 | 153.82 | 246.97 | 211.53 | 233.43 | 4.44E-01 | 3.21E-03 | 4.77E-02 |
| *NFATC4* | 42.46 | 28.07 | 34.66 | 55.75 | 50.94 | 44.60 | 5.19E-01 | 3.21E-03 | 4.77E-02 |
| *SNX22* | 9.66 | 6.81 | 8.25 | 12.43 | 12.18 | 12.72 | 4.75E-01 | 3.21E-03 | 4.77E-02 |
| *GMFB* | 181.85 | 171.60 | 155.62 | 215.60 | 196.19 | 182.63 | 3.64E-01 | 3.21E-03 | 4.77E-02 |
| *CFL2* | 654.60 | 563.84 | 573.08 | 827.26 | 661.27 | 695.64 | 3.63E-01 | 3.22E-03 | 4.78E-02 |
| *KCNN3* | 10.63 | 8.65 | 8.41 | 15.63 | 11.86 | 16.46 | 5.30E-01 | 3.24E-03 | 4.79E-02 |
| *CFAP54* | 5.59 | 6.20 | 5.29 | 4.44 | 3.93 | 4.77 | -4.20E-01 | 3.24E-03 | 4.79E-02 |
| *AGAP1* | 224.00 | 266.49 | 268.46 | 156.04 | 204.01 | 184.03 | -3.52E-01 | 3.26E-03 | 4.81E-02 |
| *BHLHE40* | 41.81 | 53.34 | 48.32 | 39.68 | 28.13 | 35.15 | -4.93E-01 | 3.26E-03 | 4.81E-02 |
| *LOC100505918* | 32.78 | 27.41 | 31.58 | 49.53 | 37.27 | 43.96 | 4.65E-01 | 3.27E-03 | 4.81E-02 |
| *CACNG6* | 139.60 | 95.74 | 119.27 | 153.96 | 151.65 | 141.00 | 4.76E-01 | 3.27E-03 | 4.82E-02 |
| *FAM3C* | 386.38 | 310.15 | 320.37 | 401.27 | 382.27 | 451.80 | 4.02E-01 | 3.28E-03 | 4.82E-02 |
| *RAD54L2* | 49.94 | 37.68 | 43.47 | 60.38 | 60.74 | 53.94 | 4.32E-01 | 3.28E-03 | 4.82E-02 |
| *NIPA1* | 26.03 | 28.05 | 28.94 | 21.77 | 23.41 | 20.77 | -4.09E-01 | 3.28E-03 | 4.82E-02 |
| *RHOU* | 226.09 | 233.14 | 222.29 | 251.01 | 239.04 | 302.36 | 3.40E-01 | 3.29E-03 | 4.82E-02 |
| *PNMA2* | 18.36 | 18.94 | 18.82 | 15.28 | 14.69 | 15.50 | -4.08E-01 | 3.30E-03 | 4.83E-02 |
| *ITGA5* | 87.07 | 103.44 | 103.33 | 63.61 | 76.01 | 73.44 | -3.69E-01 | 3.31E-03 | 4.84E-02 |
| *KCTD18* | 72.37 | 53.94 | 62.76 | 83.39 | 78.21 | 77.53 | 4.20E-01 | 3.31E-03 | 4.84E-02 |
| *LDLRAP1* | 88.95 | 102.22 | 107.10 | 73.29 | 72.17 | 75.25 | -3.29E-01 | 3.32E-03 | 4.85E-02 |
| *TNFRSF14-AS1* | 71.85 | 56.48 | 77.27 | 79.61 | 86.88 | 101.31 | 4.61E-01 | 3.35E-03 | 4.88E-02 |
| *ARRDC3* | 110.78 | 63.64 | 91.91 | 135.97 | 105.12 | 133.79 | 6.25E-01 | 3.36E-03 | 4.89E-02 |
| *BRAP* | 20.06 | 27.80 | 18.03 | 16.32 | 15.97 | 14.86 | -5.45E-01 | 3.36E-03 | 4.89E-02 |
| *CALM3* | 138.26 | 92.02 | 105.55 | 139.56 | 136.87 | 151.53 | 4.91E-01 | 3.36E-03 | 4.89E-02 |
| *KRAS* | 8.23 | 8.05 | 6.94 | 5.94 | 6.26 | 6.09 | -4.08E-01 | 3.36E-03 | 4.89E-02 |
| *ULK1* | 108.24 | 131.71 | 129.05 | 85.34 | 88.68 | 96.64 | -3.27E-01 | 3.37E-03 | 4.90E-02 |
| *TBC1D13* | 88.18 | 108.51 | 88.84 | 62.96 | 72.18 | 72.38 | -3.65E-01 | 3.39E-03 | 4.91E-02 |
| *LARS1* | 353.77 | 285.19 | 341.87 | 344.41 | 420.47 | 427.68 | 4.02E-01 | 3.39E-03 | 4.92E-02 |
| *ANK2* | 20.48 | 20.37 | 19.73 | 17.37 | 15.25 | 16.28 | -4.08E-01 | 3.41E-03 | 4.94E-02 |
| *AKAP11* | 216.09 | 239.52 | 219.46 | 146.68 | 159.44 | 186.72 | -3.25E-01 | 3.42E-03 | 4.95E-02 |
| *LINC01588* | 20.38 | 18.61 | 17.38 | 14.70 | 16.06 | 14.37 | -4.19E-01 | 3.42E-03 | 4.95E-02 |
| *PLS1* | 279.10 | 224.26 | 236.39 | 362.05 | 269.68 | 292.02 | 4.49E-01 | 3.43E-03 | 4.95E-02 |
| *ZNF202* | 85.25 | 118.06 | 110.61 | 71.24 | 80.68 | 64.94 | -4.26E-01 | 3.43E-03 | 4.95E-02 |
| *KCTD3* | 704.35 | 364.00 | 418.10 | 689.05 | 648.65 | 868.74 | 7.16E-01 | 3.44E-03 | 4.96E-02 |
| Total RNAs were harvested from HepG2 cells stably transfected with scramble control or sh*MYH10* (sh*MYH10*-1 and sh*MYH10*-2 were pooled as 1:1) using the RNeasy mini kit (QIAGEN, Germany). The Affymetrix GeneChip Human Gene U133 Arrays were used for gene expression profiling, which was performed by CapitalBio Corporation (Beijing, China). The difference of gene expression levels between the sh*MYH10* and shCtrl was assessed by linear model limma library. Adjusted *P* (FDR) < 0.05 was considered to be statistically significant. FDR, false discovery rate. | | | | | | | | | |

**Supplementary Table S5: GSEA results in HepG2 cells upon knockdown of MYH10.**

| **Gene sets** | **Size** | **ES** | **NES** | ***P*** | **FDR** |
| --- | --- | --- | --- | --- | --- |
| **EGFR_UP.V1_UP** | **97** | **-7.10E-01** | **-2.619416** | **0.00E+00** | **0.00E+00** |
| **AMIT_EGF_RESPONSE_480_HELA** | **59** | **-7.26E-01** | **-2.4697824** | **0.00E+00** | **0.00E+00** |
| CHARAFE_BREAST_CANCER_LUMINAL_VS_BASAL_DN | 172 | -6.27E-01 | -2.455757 | 0.00E+00 | 0.00E+00 |
| **ZWANG_CLASS_3_TRANSIENTLY_INDUCED_BY_EGF** | **88** | **-6.75E-01** | **-2.427204** | **0.00E+00** | **0.00E+00** |
| GSE36891_POLYIC_TLR3_VS_PAM_TLR2_STIM_PERITONEAL_MACROPHAGE_UP | 49 | -7.34E-01 | -2.4268599 | 0.00E+00 | 0.00E+00 |
| CHARAFE_BREAST_CANCER_LUMINAL_VS_MESENCHYMAL_DN | 181 | -6.03E-01 | -2.3928106 | 0.00E+00 | 0.00E+00 |
| **MEK_UP.V1_UP** | **92** | **-6.53E-01** | **-2.389191** | **0.00E+00** | **0.00E+00** |
| AZARE_NEOPLASTIC_TRANSFORMATION_BY_STAT3_UP | 40 | -7.50E-01 | -2.3618815 | 0.00E+00 | 0.00E+00 |
| **PEDERSEN_METASTASIS_BY_ERBB2_ISOFORM_1** | **22** | **-8.33E-01** | **-2.3400514** | **0.00E+00** | **0.00E+00** |
| PID_INTEGRIN1_PATHWAY | 28 | -7.96E-01 | -2.337012 | 0.00E+00 | 0.00E+00 |
| MANALO_HYPOXIA_UP | 79 | -6.58E-01 | -2.336373 | 0.00E+00 | 0.00E+00 |
| WINZEN_DEGRADED_VIA_KHSRP | 49 | -6.89E-01 | -2.323646 | 0.00E+00 | 0.00E+00 |
| BROWNE_HCMV_INFECTION_24HR_DN | 52 | -6.92E-01 | -2.3220656 | 0.00E+00 | 0.00E+00 |
| WANG_SMARCE1_TARGETS_UP | 92 | -6.37E-01 | -2.3176267 | 0.00E+00 | 0.00E+00 |
| **AMIT_EGF_RESPONSE_120_HELA** | **32** | **-7.56E-01** | **-2.3018289** | **0.00E+00** | **0.00E+00** |
| LINDSTEDT_DENDRITIC_CELL_MATURATION_B | 25 | -7.95E-01 | -2.299335 | 0.00E+00 | 0.00E+00 |
| HALLMARK_TNFA_SIGNALING_VIA_NFKB | 88 | -6.30E-01 | -2.2875042 | 0.00E+00 | 6.87E-05 |
| GSE42021_TREG_PLN_VS_CD24INT_TREG_THYMUS_DN | 75 | -6.40E-01 | -2.264978 | 0.00E+00 | 1.96E-04 |
| LTE2_UP.V1_UP | 85 | -6.33E-01 | -2.2578902 | 0.00E+00 | 1.86E-04 |
| NAKAMURA_TUMOR_ZONE_PERIPHERAL_VS_CENTRAL_DN | 217 | -5.45E-01 | -2.2417293 | 0.00E+00 | 2.94E-04 |
| VECCHI_GASTRIC_CANCER_ADVANCED_VS_EARLY_UP | 53 | -6.92E-01 | -2.2415318 | 0.00E+00 | 2.80E-04 |
| **BILD_HRAS_ONCOGENIC_SIGNATURE** | **99** | **-6.09E-01** | **-2.2292604** | **0.00E+00** | **2.67E-04** |
| GSE14769_UNSTIM_VS_60MIN_LPS_BMDM_DN | 79 | -6.28E-01 | -2.2270849 | 0.00E+00 | 2.56E-04 |
| GSE9960_HEALTHY_VS_GRAM_NEG_SEPSIS_PBMC_DN | 76 | -6.24E-01 | -2.216738 | 0.00E+00 | 2.45E-04 |
| PHONG_TNF_RESPONSE_VIA_P38_PARTIAL | 66 | -6.39E-01 | -2.2048032 | 0.00E+00 | 3.76E-04 |
| HORIUCHI_WTAP_TARGETS_UP | 120 | -5.86E-01 | -2.202321 | 0.00E+00 | 4.07E-04 |
| SCHUETZ_BREAST_CANCER_DUCTAL_INVASIVE_UP | 122 | -5.77E-01 | -2.2020447 | 0.00E+00 | 3.91E-04 |
| MODULE_357 | 33 | -7.19E-01 | -2.2008011 | 0.00E+00 | 3.77E-04 |
| DELYS_THYROID_CANCER_UP | 194 | -5.49E-01 | -2.1910036 | 0.00E+00 | 4.05E-04 |
| PRAMOONJAGO_SOX4_TARGETS_UP | 24 | -7.71E-01 | -2.190781 | 0.00E+00 | 3.91E-04 |
| **ERB2_UP.V1_UP** | **84** | **-5.98E-01** | **-2.1855288** | **0.00E+00** | **4.54E-04** |
| GSE9988_ANTI_TREM1_VS_VEHICLE_TREATED_MONOCYTES_UP | 90 | -5.98E-01 | -2.1819074 | 0.00E+00 | 4.77E-04 |
| HUANG_DASATINIB_RESISTANCE_UP | 37 | -7.06E-01 | -2.181118 | 0.00E+00 | 4.62E-04 |
| PHONG_TNF_RESPONSE_NOT_VIA_P38 | 125 | -5.69E-01 | -2.1756113 | 0.00E+00 | 4.49E-04 |
| MODULE_297 | 33 | -7.19E-01 | -2.1749544 | 0.00E+00 | 4.36E-04 |
| DUTERTRE_ESTRADIOL_RESPONSE_24HR_DN | 228 | -5.30E-01 | -2.1739218 | 0.00E+00 | 4.89E-04 |
| SENESE_HDAC1_TARGETS_UP | 92 | -5.94E-01 | -2.1723754 | 0.00E+00 | 5.39E-04 |
| GSE37605_TREG_VS_TCONV_NOD_FOXP3_FUSION_GFP_UP | 49 | -6.55E-01 | -2.1686783 | 0.00E+00 | 6.49E-04 |
| GO_MULTICELLULAR_ORGANISMAL_MACROMOLECULE_METABOLIC_PROCESS | 23 | -7.62E-01 | -2.1666625 | 0.00E+00 | 6.32E-04 |
| GSE14769_UNSTIM_VS_40MIN_LPS_BMDM_DN | 88 | -6.05E-01 | -2.16461 | 0.00E+00 | 6.46E-04 |
| ZHAN_MULTIPLE_MYELOMA_CD1_VS_CD2_UP | 26 | -7.40E-01 | -2.1643605 | 0.00E+00 | 6.30E-04 |
| REN_ALVEOLAR_RHABDOMYOSARCOMA_DN | 174 | -5.41E-01 | -2.146429 | 0.00E+00 | 9.50E-04 |
| KEGG_SMALL_CELL_LUNG_CANCER | 37 | -6.94E-01 | -2.1463566 | 0.00E+00 | 9.28E-04 |
| GSE42021_TREG_PLN_VS_CD24LO_TREG_THYMUS_DN | 90 | -5.87E-01 | -2.1461203 | 0.00E+00 | 9.07E-04 |
| GSE9988_ANTI_TREM1_VS_CTRL_TREATED_MONOCYTES_UP | 84 | -6.03E-01 | -2.1458051 | 0.00E+00 | 9.39E-04 |
| SMITH_TERT_TARGETS_UP | 78 | -5.98E-01 | -2.144342 | 0.00E+00 | 9.70E-04 |
| **GO_INACTIVATION_OF_MAPK_ACTIVITY** | **13** | **-8.55E-01** | **-2.142561** | **0.00E+00** | **1.05E-03** |
| JI_METASTASIS_REPRESSED_BY_STK11 | 11 | -9.16E-01 | -2.1374252 | 0.00E+00 | 1.17E-03 |
| LTE2_UP.V1_DN | 86 | -5.93E-01 | -2.1353471 | 0.00E+00 | 1.29E-03 |
| GHANDHI_DIRECT_IRRADIATION_UP | 45 | -6.60E-01 | -2.1345205 | 0.00E+00 | 1.31E-03 |
| GROSS_HYPOXIA_VIA_ELK3_DN | 74 | -6.11E-01 | -2.1295524 | 0.00E+00 | 1.40E-03 |
| GO_MULTICELLULAR_ORGANISM_METABOLIC_PROCESS | 30 | -7.06E-01 | -2.1294906 | 0.00E+00 | 1.38E-03 |
| MODULE_47 | 82 | -5.91E-01 | -2.1266594 | 0.00E+00 | 1.40E-03 |
| TSUNODA_CISPLATIN_RESISTANCE_DN | 27 | -7.39E-01 | -2.1256578 | 0.00E+00 | 1.37E-03 |
| DACOSTA_UV_RESPONSE_VIA_ERCC3_DN | 293 | -5.07E-01 | -2.125579 | 0.00E+00 | 1.35E-03 |
| GSE30971_WBP7_HET_VS_KO_MACROPHAGE_2H_LPS_STIM_DN | 69 | -6.12E-01 | -2.1253176 | 0.00E+00 | 1.34E-03 |
| REACTOME_NFKB_AND_MAP_KINASES_ACTIVATION_MEDIATED_BY_TLR4_SIGNALING_REPERTOIRE | 21 | -7.72E-01 | -2.1248145 | 0.00E+00 | 1.34E-03 |
| FRASOR_RESPONSE_TO_ESTRADIOL_DN | 44 | -6.56E-01 | -2.1238256 | 0.00E+00 | 1.36E-03 |
| KRIGE_RESPONSE_TO_TOSEDOSTAT_24HR_UP | 283 | -5.14E-01 | -2.122963 | 0.00E+00 | 1.37E-03 |
| DACOSTA_UV_RESPONSE_VIA_ERCC3_COMMON_DN | 164 | -5.39E-01 | -2.1195388 | 0.00E+00 | 1.41E-03 |
| GSE30971_CTRL_VS_LPS_STIM_MACROPHAGE_WBP7_KO_4H_UP | 72 | -6.17E-01 | -2.1189456 | 0.00E+00 | 1.42E-03 |
| GABRIELY_MIR21_TARGETS | 73 | -5.98E-01 | -2.115926 | 0.00E+00 | 1.44E-03 |
| ENK_UV_RESPONSE_EPIDERMIS_DN | 228 | -5.19E-01 | -2.1150188 | 0.00E+00 | 1.44E-03 |
| MODULE_154 | 30 | -7.12E-01 | -2.1130693 | 0.00E+00 | 1.50E-03 |
| ELVIDGE_HYPOXIA_BY_DMOG_UP | 57 | -6.28E-01 | -2.1103115 | 0.00E+00 | 1.59E-03 |
| **AMIT_EGF_RESPONSE_60_MCF10A** | **19** | **-7.87E-01** | **-2.10977** | **0.00E+00** | **1.58E-03** |
| GTACTGT_MIR101 | 82 | -5.95E-01 | -2.1068265 | 0.00E+00 | 1.67E-03 |
| FOURNIER_ACINAR_DEVELOPMENT_LATE_DN | 13 | -8.64E-01 | -2.1059403 | 0.00E+00 | 1.68E-03 |
| FORTSCHEGGER_PHF8_TARGETS_UP | 113 | -5.61E-01 | -2.1058607 | 0.00E+00 | 1.65E-03 |
| P53_DN.V1_UP | 95 | -5.82E-01 | -2.1042206 | 0.00E+00 | 1.69E-03 |
| ONDER_CDH1_TARGETS_1_UP | 52 | -6.27E-01 | -2.1038218 | 0.00E+00 | 1.67E-03 |
| GO_PROTEIN_TYROSINE_SERINE_THREONINE_PHOSPHATASE_ACTIVITY | 18 | -8.03E-01 | -2.1028154 | 0.00E+00 | 1.73E-03 |
| STK33_NOMO_UP | 108 | -5.63E-01 | -2.0983543 | 0.00E+00 | 1.91E-03 |
| GSE41176_UNSTIM_VS_ANTI_IGM_STIM_BCELL_1H_UP | 70 | -6.12E-01 | -2.098044 | 0.00E+00 | 1.90E-03 |
| PEDERSEN_TARGETS_OF_611CTF_ISOFORM_OF_ERBB2 | 30 | -7.03E-01 | -2.0939302 | 0.00E+00 | 2.08E-03 |
| REACTOME_TRIF_MEDIATED_TLR3_SIGNALING | 25 | -7.18E-01 | -2.092517 | 0.00E+00 | 2.07E-03 |
| **RAF_UP.V1_UP** | **74** | **-5.89E-01** | **-2.091384** | **0.00E+00** | **2.09E-03** |
| GSE34392_ST2_KO_VS_WT_DAY8_LCMV_EFFECTOR_CD8_TCELL_DN | 86 | -5.75E-01 | -2.0912395 | 0.00E+00 | 2.06E-03 |
| NAGASHIMA_NRG1_SIGNALING_UP | 83 | -5.84E-01 | -2.0903773 | 0.00E+00 | 2.05E-03 |
| BOQUEST_STEM_CELL_CULTURED_VS_FRESH_UP | 148 | -5.35E-01 | -2.0892878 | 0.00E+00 | 2.02E-03 |
| WEINMANN_ADAPTATION_TO_HYPOXIA_DN | 15 | -8.01E-01 | -2.089131 | 0.00E+00 | 2.01E-03 |
| **SWEET_KRAS_TARGETS_UP** | **39** | **-6.66E-01** | **-2.0890734** | **0.00E+00** | **1.99E-03** |
| DORN_ADENOVIRUS_INFECTION_32HR_DN | 19 | -7.70E-01 | -2.08809 | 0.00E+00 | 2.04E-03 |
| EIF4E_DN | 40 | -6.49E-01 | -2.0875587 | 0.00E+00 | 2.04E-03 |
| KEGG_PATHWAYS_IN_CANCER | 122 | -5.53E-01 | -2.0856404 | 0.00E+00 | 2.10E-03 |
| GROSS_HYPOXIA_VIA_ELK3_AND_HIF1A_UP | 73 | -5.96E-01 | -2.0845256 | 0.00E+00 | 2.14E-03 |
| PID_AVB3_INTEGRIN_PATHWAY | 29 | -7.09E-01 | -2.0822396 | 0.00E+00 | 2.25E-03 |
| SABATES_COLORECTAL_ADENOMA_UP | 52 | -6.27E-01 | -2.0810378 | 0.00E+00 | 2.25E-03 |
| GSE27434_WT_VS_DNMT1_KO_TREG_DN | 76 | -5.91E-01 | -2.080593 | 0.00E+00 | 2.24E-03 |
| SENESE_HDAC3_TARGETS_UP | 118 | -5.52E-01 | -2.0799263 | 0.00E+00 | 2.28E-03 |
| KIM_WT1_TARGETS_12HR_DN | 72 | -5.94E-01 | -2.0792377 | 0.00E+00 | 2.28E-03 |
| CROONQUIST_STROMAL_STIMULATION_UP | 26 | -7.18E-01 | -2.0776243 | 0.00E+00 | 2.34E-03 |
| TURASHVILI_BREAST_LOBULAR_CARCINOMA_VS_DUCTAL_NORMAL_DN | 23 | -7.39E-01 | -2.0773926 | 0.00E+00 | 2.32E-03 |
| ELVIDGE_HYPOXIA_UP | 71 | -5.96E-01 | -2.071046 | 0.00E+00 | 2.57E-03 |
| TIEN_INTESTINE_PROBIOTICS_24HR_DN | 63 | -6.02E-01 | -2.0678272 | 0.00E+00 | 2.72E-03 |
| TATTATA_MIR374 | 91 | -5.68E-01 | -2.065654 | 0.00E+00 | 2.91E-03 |
| GSE22886_DAY0_VS_DAY1_MONOCYTE_IN_CULTURE_DN | 81 | -5.77E-01 | -2.06204 | 0.00E+00 | 3.04E-03 |
| NUYTTEN_EZH2_TARGETS_UP | 429 | -4.78E-01 | -2.0615337 | 0.00E+00 | 3.05E-03 |
| HALLMARK_EPITHELIAL_MESENCHYMAL_TRANSITION | 71 | -5.78E-01 | -2.059778 | 0.00E+00 | 3.14E-03 |
| MEL18_DN.V1_UP | 63 | -5.97E-01 | -2.0594335 | 0.00E+00 | 3.12E-03 |
| OSWALD_HEMATOPOIETIC_STEM_CELL_IN_COLLAGEN_GEL_UP | 75 | -5.83E-01 | -2.058758 | 0.00E+00 | 3.10E-03 |
| GSE35825_UNTREATED_VS_IFNG_STIM_MACROPHAGE_UP | 76 | -5.77E-01 | -2.0577562 | 0.00E+00 | 3.14E-03 |
| GO_SENSORY_PERCEPTION_OF_MECHANICAL_STIMULUS | 45 | -6.34E-01 | -2.0573573 | 0.00E+00 | 3.15E-03 |
| GSE24671_CTRL_VS_BAKIMULC_INFECTED_MOUSE_SPLENOCYTES_DN | 62 | -5.95E-01 | -2.0567062 | 0.00E+00 | 3.15E-03 |
| GSE21546_UNSTIM_VS_ANTI_CD3_STIM_SAP1A_KO_DP_THYMOCYTES_UP | 83 | -5.76E-01 | -2.0553944 | 0.00E+00 | 3.15E-03 |
| CHEN_HOXA5_TARGETS_9HR_UP | 98 | -5.56E-01 | -2.0549805 | 0.00E+00 | 3.18E-03 |
| LEE_NEURAL_CREST_STEM_CELL_DN | 49 | -6.39E-01 | -2.0546405 | 0.00E+00 | 3.18E-03 |
| BLUM_RESPONSE_TO_SALIRASIB_UP | 105 | -5.54E-01 | -2.054035 | 0.00E+00 | 3.17E-03 |
| ZHU_CMV_24_HR_DN | 36 | -6.67E-01 | -2.052 | 0.00E+00 | 3.30E-03 |
| TAKEDA_TARGETS_OF_NUP98_HOXA9_FUSION_10D_UP | 54 | -6.14E-01 | -2.0490134 | 0.00E+00 | 3.48E-03 |
| BRUECKNER_TARGETS_OF_MIRLET7A3_UP | 48 | -6.19E-01 | -2.0463996 | 0.00E+00 | 3.66E-03 |
| GSE29617_CTRL_VS_DAY7_TIV_FLU_VACCINE_PBMC_2008_UP | 72 | -5.81E-01 | -2.0449035 | 0.00E+00 | 3.76E-03 |
| HOFMANN_MYELODYSPLASTIC_SYNDROM_RISK_UP | 15 | -8.09E-01 | -2.0440662 | 0.00E+00 | 3.81E-03 |
| **KOBAYASHI_EGFR_SIGNALING_6HR_DN** | **11** | **-8.88E-01** | **-2.043417** | **0.00E+00** | **3.80E-03** |
| STAMBOLSKY_RESPONSE_TO_VITAMIN_D3_UP | 26 | -7.09E-01 | -2.032937 | 1.54E-03 | 4.69E-03 |
| STK33_UP | 114 | -5.40E-01 | -2.0325387 | 0.00E+00 | 4.68E-03 |
| GALINDO_IMMUNE_RESPONSE_TO_ENTEROTOXIN | 34 | -6.69E-01 | -2.0317783 | 0.00E+00 | 4.70E-03 |
| GSE18791_CTRL_VS_NEWCASTLE_VIRUS_DC_12H_DN | 89 | -5.68E-01 | -2.0314832 | 0.00E+00 | 4.67E-03 |
| GO_CELLULAR_RESPONSE_TO_AMINO_ACID_STIMULUS | 20 | -7.41E-01 | -2.0300496 | 0.00E+00 | 4.74E-03 |
| WANG_SMARCE1_TARGETS_DN | 153 | -5.19E-01 | -2.029945 | 0.00E+00 | 4.70E-03 |
| **AMIT_EGF_RESPONSE_40_HELA** | **16** | **-7.93E-01** | **-2.0289395** | **0.00E+00** | **4.78E-03** |
| PICCALUGA_ANGIOIMMUNOBLASTIC_LYMPHOMA_UP | 62 | -5.89E-01 | -2.0288749 | 0.00E+00 | 4.74E-03 |
| SCHAEFFER_PROSTATE_DEVELOPMENT_12HR_UP | 45 | -6.39E-01 | -2.0287187 | 0.00E+00 | 4.71E-03 |
| GSE42021_TCONV_PLN_VS_TREG_PRECURSORS_THYMUS_UP | 89 | -5.57E-01 | -2.0267518 | 0.00E+00 | 4.88E-03 |
| GSE43955_1H_VS_20H_ACT_CD4_TCELL_WITH_TGFB_IL6_DN | 68 | -5.85E-01 | -2.0251055 | 0.00E+00 | 4.96E-03 |
| GSE13485_CTRL_VS_DAY21_YF17D_VACCINE_PBMC_UP | 88 | -5.64E-01 | -2.0242867 | 0.00E+00 | 5.03E-03 |
| HIRSCH_CELLULAR_TRANSFORMATION_SIGNATURE_UP | 110 | -5.37E-01 | -2.0234127 | 0.00E+00 | 5.07E-03 |
| GSE4748_CTRL_VS_LPS_AND_CYANOBACTERIUM_LPSLIKE_STIM_DC_3H_UP | 80 | -5.67E-01 | -2.0217385 | 0.00E+00 | 5.24E-03 |
| MODULE_1 | 140 | -5.26E-01 | -2.0216038 | 0.00E+00 | 5.21E-03 |
| KRIGE_RESPONSE_TO_TOSEDOSTAT_6HR_UP | 341 | -4.78E-01 | -2.0208242 | 0.00E+00 | 5.23E-03 |
| KEGG_ECM_RECEPTOR_INTERACTION | 34 | -6.63E-01 | -2.0206664 | 0.00E+00 | 5.20E-03 |
| GHANDHI_BYSTANDER_IRRADIATION_UP | 30 | -6.80E-01 | -2.0201843 | 0.00E+00 | 5.22E-03 |
| GAJATE_RESPONSE_TO_TRABECTEDIN_UP | 36 | -6.47E-01 | -2.0181067 | 0.00E+00 | 5.34E-03 |
| COULOUARN_TEMPORAL_TGFB1_SIGNATURE_UP | 41 | -6.38E-01 | -2.0166104 | 0.00E+00 | 5.48E-03 |
| **REACTOME_MAP_KINASE_ACTIVATION_IN_TLR_CASCADE** | **14** | **-8.22E-01** | **-2.016124** | **0.00E+00** | **5.50E-03** |
| JOHNSTONE_PARVB_TARGETS_3_UP | 167 | -5.12E-01 | -2.0153408 | 0.00E+00 | 5.55E-03 |
| MILI_PSEUDOPODIA_CHEMOTAXIS_DN | 188 | -5.04E-01 | -2.0136812 | 0.00E+00 | 5.67E-03 |
| BURTON_ADIPOGENESIS_PEAK_AT_2HR | 25 | -6.99E-01 | -2.0109274 | 0.00E+00 | 5.85E-03 |
| DOUGLAS_BMI1_TARGETS_DN | 128 | -5.23E-01 | -2.0104027 | 0.00E+00 | 5.89E-03 |
| WU_CELL_MIGRATION | 89 | -5.49E-01 | -2.0095255 | 0.00E+00 | 5.95E-03 |
| REACTOME_MYD88_MAL_CASCADE_INITIATED_ON_PLASMA_MEMBRANE | 25 | -7.07E-01 | -2.009142 | 0.00E+00 | 5.96E-03 |
| PASINI_SUZ12_TARGETS_DN | 122 | -5.31E-01 | -2.0083838 | 0.00E+00 | 5.99E-03 |
| GO_EXTRACELLULAR_MATRIX_COMPONENT | 53 | -6.12E-01 | -2.0080695 | 0.00E+00 | 5.97E-03 |
| SENESE_HDAC1_AND_HDAC2_TARGETS_UP | 57 | -6.01E-01 | -2.0064034 | 0.00E+00 | 6.06E-03 |
| NAGASHIMA_EGF_SIGNALING_UP | 30 | -6.65E-01 | -1.9992585 | 0.00E+00 | 6.72E-03 |
| BMI1_DN_MEL18_DN.V1_UP | 61 | -5.91E-01 | -1.9984872 | 0.00E+00 | 6.79E-03 |
| NGUYEN_NOTCH1_TARGETS_DN | 34 | -6.63E-01 | -1.9978962 | 0.00E+00 | 6.82E-03 |
| GSE13522_CTRL_VS_T_CRUZI_Y_STRAIN_INF_SKIN_IFNG_KO_UP | 71 | -5.69E-01 | -1.9945784 | 0.00E+00 | 7.15E-03 |
| BMI1_DN.V1_UP | 73 | -5.72E-01 | -1.9920434 | 0.00E+00 | 7.45E-03 |
| GSE9037_CTRL_VS_LPS_4H_STIM_IRAK4_KO_BMDM_DN | 74 | -5.69E-01 | -1.9910022 | 0.00E+00 | 7.57E-03 |
| REACTOME_ACTIVATED_TLR4_SIGNALLING | 29 | -6.72E-01 | -1.9899999 | 0.00E+00 | 7.67E-03 |
| GSE2770_TGFB_AND_IL4_VS_IL4_TREATED_ACT_CD4_TCELL_48H_UP | 81 | -5.53E-01 | -1.9889933 | 0.00E+00 | 7.75E-03 |
| TURASHVILI_BREAST_DUCTAL_CARCINOMA_VS_DUCTAL_NORMAL_DN | 53 | -5.95E-01 | -1.988114 | 0.00E+00 | 7.77E-03 |
| DORN_ADENOVIRUS_INFECTION_48HR_DN | 19 | -7.37E-01 | -1.9860537 | 0.00E+00 | 7.97E-03 |
| GOTZMANN_EPITHELIAL_TO_MESENCHYMAL_TRANSITION_UP | 29 | -6.76E-01 | -1.9850289 | 0.00E+00 | 8.01E-03 |
| **SWEET_KRAS_TARGETS_DN** | **31** | **-6.67E-01** | **-1.9841075** | **0.00E+00** | **8.07E-03** |
| GO_REGULATION_OF_EMBRYONIC_DEVELOPMENT | 33 | -6.52E-01 | -1.9838917 | 0.00E+00 | 8.06E-03 |
| GSE42021_TREG_PLN_VS_CD24HI_TREG_THYMUS_UP | 70 | -5.66E-01 | -1.9834498 | 0.00E+00 | 8.09E-03 |
| GSE30971_WBP7_HET_VS_KO_MACROPHAGE_DN | 76 | -5.60E-01 | -1.9829332 | 0.00E+00 | 8.11E-03 |
| JAEGER_METASTASIS_DN | 82 | -5.62E-01 | -1.9803647 | 0.00E+00 | 8.44E-03 |
| RIGGI_EWING_SARCOMA_PROGENITOR_UP | 142 | -5.14E-01 | -1.9800698 | 0.00E+00 | 8.46E-03 |
| PROVENZANI_METASTASIS_UP | 87 | -5.52E-01 | -1.979114 | 0.00E+00 | 8.62E-03 |
| KARLSSON_TGFB1_TARGETS_UP | 43 | -6.11E-01 | -1.9786313 | 0.00E+00 | 8.66E-03 |
| LI_WILMS_TUMOR_VS_FETAL_KIDNEY_2_DN | 20 | -7.20E-01 | -1.9768978 | 0.00E+00 | 8.85E-03 |
| REACTOME_TRAF6_MEDIATED_INDUCTION_OF_NFKB_AND_MAP_KINASES_UPON_TLR7_8_OR_9_ACTIVATION | 25 | -7.07E-01 | -1.9735858 | 0.00E+00 | 9.32E-03 |
| PLASARI_TGFB1_TARGETS_10HR_UP | 78 | -5.56E-01 | -1.9725668 | 0.00E+00 | 9.42E-03 |
| GSE3982_MEMORY_CD4_TCELL_VS_TH2_DN | 88 | -5.42E-01 | -1.9713644 | 0.00E+00 | 9.55E-03 |
| GRUETZMANN_PANCREATIC_CANCER_UP | 160 | -5.00E-01 | -1.9712036 | 0.00E+00 | 9.52E-03 |
| GO_REGULATION_OF_PROTEIN_SERINE_THREONINE_KINASE_ACTIVITY | 182 | -4.93E-01 | -1.9711611 | 0.00E+00 | 9.47E-03 |
| GSE7852_TREG_VS_TCONV_FAT_UP | 85 | -5.45E-01 | -1.9710158 | 0.00E+00 | 9.43E-03 |
| SUNG_METASTASIS_STROMA_UP | 44 | -6.21E-01 | -1.9699643 | 0.00E+00 | 9.58E-03 |
| NABA_COLLAGENS | 14 | -7.93E-01 | -1.9699576 | 0.00E+00 | 9.53E-03 |
| GSE43955_10H_VS_60H_ACT_CD4_TCELL_DN | 75 | -5.60E-01 | -1.9684954 | 0.00E+00 | 9.68E-03 |
| TAKEDA_TARGETS_OF_NUP98_HOXA9_FUSION_16D_UP | 52 | -5.84E-01 | -1.9682555 | 0.00E+00 | 9.67E-03 |
| ZHU_CMV_ALL_DN | 46 | -6.16E-01 | -1.9681228 | 0.00E+00 | 9.64E-03 |
| CHIARADONNA_NEOPLASTIC_TRANSFORMATION_CDC25_DN | 70 | -5.60E-01 | -1.9662168 | 0.00E+00 | 9.92E-03 |
| GSE18791_CTRL_VS_NEWCASTLE_VIRUS_DC_2H_DN | 63 | -5.74E-01 | -1.9661175 | 0.00E+00 | 9.89E-03 |
| GSE17301_ACD3_ACD28_VS_ACD3_ACD28_AND_IFNA2_STIM_CD8_TCELL_DN | 76 | -5.60E-01 | -1.964332 | 0.00E+00 | 1.01E-02 |
| REACTOME_COLLAGEN_FORMATION | 17 | -7.61E-01 | -1.9638306 | 1.63E-03 | 1.01E-02 |
| GO_ENDOPLASMIC_RETICULUM_LUMEN | 67 | -5.67E-01 | -1.962248 | 0.00E+00 | 1.04E-02 |
| PID_A6B1_A6B4_INTEGRIN_PATHWAY | 16 | -7.58E-01 | -1.9603727 | 0.00E+00 | 1.07E-02 |
| BUYTAERT_PHOTODYNAMIC_THERAPY_STRESS_UP | 331 | -4.63E-01 | -1.95871 | 0.00E+00 | 1.09E-02 |
| PHONG_TNF_RESPONSE_VIA_P38_COMPLETE | 72 | -5.55E-01 | -1.9581988 | 0.00E+00 | 1.09E-02 |
| AKL_HTLV1_INFECTION_DN | 25 | -6.70E-01 | -1.9577818 | 0.00E+00 | 1.09E-02 |
| RODRIGUES_THYROID_CARCINOMA_ANAPLASTIC_UP | 213 | -4.81E-01 | -1.9564034 | 0.00E+00 | 1.11E-02 |
| GO_PROTEIN_TYROSINE_PHOSPHATASE_ACTIVITY | 38 | -6.26E-01 | -1.9559362 | 0.00E+00 | 1.11E-02 |
| UZONYI_RESPONSE_TO_LEUKOTRIENE_AND_THROMBIN | 15 | -7.62E-01 | -1.9555289 | 0.00E+00 | 1.11E-02 |
| GSE9988_ANTI_TREM1_AND_LPS_VS_CTRL_TREATED_MONOCYTES_UP | 93 | -5.29E-01 | -1.9551516 | 0.00E+00 | 1.11E-02 |
| GNF2_PTX3 | 12 | -8.10E-01 | -1.9548743 | 0.00E+00 | 1.12E-02 |
| MASSARWEH_TAMOXIFEN_RESISTANCE_UP | 227 | -4.84E-01 | -1.9539521 | 0.00E+00 | 1.13E-02 |
| HALLMARK_ANGIOGENESIS | 13 | -8.00E-01 | -1.9535236 | 1.68E-03 | 1.13E-02 |
| GSE16385_ROSIGLITAZONE_IL4_VS_IFNG_TNF_STIM_MACROPHAGE_UP | 82 | -5.45E-01 | -1.9534098 | 0.00E+00 | 1.12E-02 |
| WOO_LIVER_CANCER_RECURRENCE_UP | 40 | -6.15E-01 | -1.9529859 | 0.00E+00 | 1.13E-02 |
| CHICAS_RB1_TARGETS_SENESCENT | 201 | -4.85E-01 | -1.9506265 | 0.00E+00 | 1.16E-02 |
| KOINUMA_TARGETS_OF_SMAD2_OR_SMAD3 | 354 | -4.63E-01 | -1.9493462 | 0.00E+00 | 1.18E-02 |
| GO_BASEMENT_MEMBRANE | 36 | -6.39E-01 | -1.9493105 | 0.00E+00 | 1.18E-02 |
| HAN_SATB1_TARGETS_DN | 180 | -4.88E-01 | -1.946508 | 0.00E+00 | 1.22E-02 |
| PID_SYNDECAN_1_PATHWAY | 17 | -7.43E-01 | -1.9452662 | 0.00E+00 | 1.25E-02 |
| MODULE_12 | 128 | -5.08E-01 | -1.9444324 | 0.00E+00 | 1.25E-02 |
| LIN_NPAS4_TARGETS_DN | 25 | -6.92E-01 | -1.9442071 | 1.63E-03 | 1.25E-02 |
| DAVICIONI_TARGETS_OF_PAX_FOXO1_FUSIONS_UP | 108 | -5.26E-01 | -1.943915 | 0.00E+00 | 1.25E-02 |
| GARGALOVIC_RESPONSE_TO_OXIDIZED_PHOSPHOLIPIDS_BLUE_UP | 54 | -5.85E-01 | -1.9434004 | 1.47E-03 | 1.25E-02 |
| GO_NEGATIVE_REGULATION_OF_INNATE_IMMUNE_RESPONSE | 14 | -7.74E-01 | -1.9433378 | 0.00E+00 | 1.25E-02 |
| HALLMARK_UV_RESPONSE_DN | 50 | -5.78E-01 | -1.9395462 | 0.00E+00 | 1.32E-02 |
| GSE36891_UNSTIM_VS_POLYIC_TLR3_STIM_PERITONEAL_MACROPHAGE_UP | 58 | -5.78E-01 | -1.9367961 | 0.00E+00 | 1.36E-02 |
| GSE2706_UNSTIM_VS_2H_LPS_AND_R848_DC_DN | 82 | -5.41E-01 | -1.9360824 | 0.00E+00 | 1.37E-02 |
| HOELZEL_NF1_TARGETS_UP | 36 | -6.25E-01 | -1.9359726 | 0.00E+00 | 1.36E-02 |
| GSE24142_ADULT_VS_FETAL_DN3_THYMOCYTE_UP | 66 | -5.58E-01 | -1.9357204 | 0.00E+00 | 1.36E-02 |
| GSE9988_ANTI_TREM1_AND_LPS_VS_VEHICLE_TREATED_MONOCYTES_UP | 85 | -5.43E-01 | -1.9348235 | 0.00E+00 | 1.36E-02 |
| GSE42088_UNINF_VS_LEISHMANIA_INF_DC_24H_DN | 77 | -5.42E-01 | -1.9342595 | 0.00E+00 | 1.37E-02 |
| MILI_PSEUDOPODIA_HAPTOTAXIS_DN | 333 | -4.57E-01 | -1.9313148 | 0.00E+00 | 1.43E-02 |
| HALLMARK_NOTCH_SIGNALING | 17 | -7.32E-01 | -1.9308811 | 0.00E+00 | 1.43E-02 |
| GSE11057_NAIVE_VS_MEMORY_CD4_TCELL_UP | 76 | -5.39E-01 | -1.9294126 | 0.00E+00 | 1.45E-02 |
| **CROONQUIST_NRAS_VS_STROMAL_STIMULATION_DN** | **47** | **-5.87E-01** | **-1.9286202** | **0.00E+00** | **1.46E-02** |
| GSE9960_HEALTHY_VS_GRAM_POS_SEPSIS_PBMC_DN | 79 | -5.48E-01 | -1.9272847 | 0.00E+00 | 1.48E-02 |
| LU_EZH2_TARGETS_DN | 138 | -4.95E-01 | -1.9269087 | 0.00E+00 | 1.48E-02 |
| GNF2_CDH3 | 15 | -7.55E-01 | -1.9266591 | 4.84E-03 | 1.48E-02 |
| NABA_BASEMENT_MEMBRANES | 19 | -7.16E-01 | -1.9262285 | 1.60E-03 | 1.48E-02 |
| WANG_HCP_PROSTATE_CANCER | 44 | -6.03E-01 | -1.9257815 | 0.00E+00 | 1.48E-02 |
| **ACEVEDO_FGFR1_TARGETS_IN_PROSTATE_CANCER_MODEL_UP** | **110** | **-5.10E-01** | **-1.9235654** | **0.00E+00** | **1.53E-02** |
| **CHIARADONNA_NEOPLASTIC_TRANSFORMATION_KRAS_CDC25_UP** | **24** | **-6.74E-01** | **-1.9229906** | **0.00E+00** | **1.54E-02** |
| CLASPER_LYMPHATIC_VESSELS_DURING_METASTASIS_DN | 13 | -7.88E-01 | -1.922336 | 0.00E+00 | 1.55E-02 |
| **GO_REGULATION_OF_MAP_KINASE_ACTIVITY** | **117** | **-5.09E-01** | **-1.9222687** | **0.00E+00** | **1.54E-02** |
| GSE21546_WT_VS_SAP1A_KO_AND_ELK1_KO_ANTI_CD3_STIM_DP_THYMOCYTES_UP | 75 | -5.40E-01 | -1.9219257 | 0.00E+00 | 1.54E-02 |
| FRIDMAN_IMMORTALIZATION_DN | 16 | -7.49E-01 | -1.9208326 | 0.00E+00 | 1.57E-02 |
| LINDGREN_BLADDER_CANCER_HIGH_RECURRENCE | 19 | -7.15E-01 | -1.920578 | 1.64E-03 | 1.57E-02 |
| GSE17301_IFNA2_VS_IFNA2_AND_ACD3_ACD28_STIM_CD8_TCELL_UP | 81 | -5.37E-01 | -1.919814 | 0.00E+00 | 1.57E-02 |
| **GO_NEGATIVE_REGULATION_OF_TRANSFORMING_GROWTH_FACTOR_BETA_RECEPTOR_SIGNALING_PATHWAY** | **24** | **-6.69E-01** | **-1.9195234** | **1.55E-03** | **1.57E-02** |
| ONDER_CDH1_TARGETS_2_DN | 205 | -4.79E-01 | -1.9184089 | 0.00E+00 | 1.59E-02 |
| CROMER_TUMORIGENESIS_UP | 23 | -6.74E-01 | -1.9183325 | 1.53E-03 | 1.59E-02 |
| SEKI_INFLAMMATORY_RESPONSE_LPS_UP | 32 | -6.38E-01 | -1.9162153 | 0.00E+00 | 1.63E-02 |
| **GO_POSITIVE_REGULATION_OF_PROTEIN_SERINE_THREONINE_KINASE_ACTIVITY** | **110** | **-5.09E-01** | **-1.9150914** | **0.00E+00** | **1.65E-02** |
| BOQUEST_STEM_CELL_UP | 89 | -5.30E-01 | -1.9146782 | 0.00E+00 | 1.66E-02 |
| MANN_RESPONSE_TO_AMIFOSTINE_UP | 15 | -7.42E-01 | -1.9141084 | 1.64E-03 | 1.67E-02 |
| TBK1.DF_DN | 103 | -5.20E-01 | -1.9123099 | 0.00E+00 | 1.71E-02 |
| GSE7509_DC_VS_MONOCYTE_DN | 81 | -5.34E-01 | -1.9120129 | 0.00E+00 | 1.70E-02 |
| GSE36888_UNTREATED_VS_IL2_TREATED_STAT5_AB_KNOCKIN_TCELL_2H_UP | 69 | -5.45E-01 | -1.9114708 | 0.00E+00 | 1.71E-02 |
| ATF2_S_UP.V1_DN | 72 | -5.41E-01 | -1.9103304 | 0.00E+00 | 1.73E-02 |
| KAAB_FAILED_HEART_ATRIUM_DN | 49 | -5.77E-01 | -1.9100262 | 0.00E+00 | 1.73E-02 |
| SMIRNOV_CIRCULATING_ENDOTHELIOCYTES_IN_CANCER_UP | 54 | -5.79E-01 | -1.9098544 | 0.00E+00 | 1.73E-02 |
| BOYAULT_LIVER_CANCER_SUBCLASS_G12_UP | 17 | -7.20E-01 | -1.9050456 | 1.65E-03 | 1.85E-02 |
| AMIT_SERUM_RESPONSE_60_MCF10A | 17 | -7.27E-01 | -1.9046509 | 0.00E+00 | 1.85E-02 |
| GSE14769_UNSTIM_VS_80MIN_LPS_BMDM_DN | 75 | -5.38E-01 | -1.9044226 | 1.45E-03 | 1.85E-02 |
| BILANGES_SERUM_SENSITIVE_VIA_TSC1 | 13 | -7.67E-01 | -1.9012537 | 0.00E+00 | 1.92E-02 |
| MODULE_105 | 92 | -5.25E-01 | -1.9010109 | 0.00E+00 | 1.92E-02 |
| ATF2_UP.V1_DN | 66 | -5.40E-01 | -1.8991714 | 0.00E+00 | 1.97E-02 |
| CUI_TCF21_TARGETS_2_DN | 282 | -4.58E-01 | -1.8990873 | 0.00E+00 | 1.96E-02 |
| GSE36888_UNTREATED_VS_IL2_TREATED_TCELL_17H_DN | 73 | -5.32E-01 | -1.8978295 | 0.00E+00 | 1.99E-02 |
| MCBRYAN_PUBERTAL_TGFB1_TARGETS_UP | 65 | -5.50E-01 | -1.8976393 | 0.00E+00 | 1.99E-02 |
| GU_PDEF_TARGETS_UP | 29 | -6.43E-01 | -1.8971825 | 0.00E+00 | 1.99E-02 |
| JACKSON_DNMT1_TARGETS_UP | 31 | -6.35E-01 | -1.8963187 | 0.00E+00 | 2.01E-02 |
| HALLMARK_UNFOLDED_PROTEIN_RESPONSE | 48 | -5.84E-01 | -1.8960046 | 0.00E+00 | 2.01E-02 |
| IGARASHI_ATF4_TARGETS_DN | 39 | -6.17E-01 | -1.8950919 | 1.44E-03 | 2.04E-02 |
| BONOME_OVARIAN_CANCER_SURVIVAL_OPTIMAL_DEBULKING | 81 | -5.30E-01 | -1.8948568 | 0.00E+00 | 2.04E-02 |
| GSE42021_TCONV_PLN_VS_CD24HI_TCONV_THYMUS_UP | 68 | -5.48E-01 | -1.8937109 | 0.00E+00 | 2.06E-02 |
| DAUER_STAT3_TARGETS_UP | 19 | -7.07E-01 | -1.893195 | 3.13E-03 | 2.06E-02 |
| GSE37605_C57BL6_VS_NOD_FOXP3_FUSION_GFP_TREG_DN | 78 | -5.31E-01 | -1.8906085 | 0.00E+00 | 2.12E-02 |
| GSE29617_CTRL_VS_TIV_FLU_VACCINE_PBMC_2008_UP | 71 | -5.43E-01 | -1.8903688 | 0.00E+00 | 2.12E-02 |
| PID_WNT_SIGNALING_PATHWAY | 13 | -7.72E-01 | -1.8900816 | 0.00E+00 | 2.12E-02 |
| GO_PROTEIN_DEPHOSPHORYLATION | 71 | -5.34E-01 | -1.8900659 | 0.00E+00 | 2.12E-02 |
| TURASHVILI_BREAST_LOBULAR_CARCINOMA_VS_LOBULAR_NORMAL_UP | 21 | -6.90E-01 | -1.8892457 | 0.00E+00 | 2.13E-02 |
| CTTTAAR_UNKNOWN | 21 | -6.84E-01 | -1.8866581 | 0.00E+00 | 2.21E-02 |
| GO_BASAL_LAMINA | 12 | -7.62E-01 | -1.88665 | 3.34E-03 | 2.20E-02 |
| YAO_TEMPORAL_RESPONSE_TO_PROGESTERONE_CLUSTER_9 | 32 | -6.22E-01 | -1.8864957 | 0.00E+00 | 2.20E-02 |
| PICCALUGA_ANGIOIMMUNOBLASTIC_LYMPHOMA_DN | 48 | -5.78E-01 | -1.8859655 | 1.46E-03 | 2.21E-02 |
| DAZARD_RESPONSE_TO_UV_NHEK_DN | 111 | -5.00E-01 | -1.8859016 | 0.00E+00 | 2.20E-02 |
| **CROONQUIST_NRAS_VS_STROMAL_STIMULATION_UP** | **16** | **-7.24E-01** | **-1.8847301** | **3.36E-03** | **2.23E-02** |
| GSE37605_FOXP3_FUSION_GFP_VS_IRES_GFP_TREG_C57BL6_UP | 82 | -5.26E-01 | -1.8846647 | 0.00E+00 | 2.22E-02 |
| GO_COATED_PIT | 21 | -6.74E-01 | -1.8804394 | 0.00E+00 | 2.35E-02 |
| DAVICIONI_MOLECULAR_ARMS_VS_ERMS_DN | 77 | -5.35E-01 | -1.8788512 | 0.00E+00 | 2.39E-02 |
| MANTOVANI_NFKB_TARGETS_UP | 16 | -7.46E-01 | -1.8788508 | 0.00E+00 | 2.38E-02 |
| GO_REGULATION_OF_CELLULAR_RESPONSE_TO_TRANSFORMING_GROWTH_FACTOR_BETA_STIMULUS | 39 | -5.99E-01 | -1.8781945 | 0.00E+00 | 2.40E-02 |
| GSE41176_UNSTIM_VS_ANTI_IGM_STIM_BCELL_3H_UP | 79 | -5.34E-01 | -1.8778734 | 0.00E+00 | 2.40E-02 |
| PETROVA_ENDOTHELIUM_LYMPHATIC_VS_BLOOD_DN | 75 | -5.26E-01 | -1.8777816 | 1.41E-03 | 2.39E-02 |
| GSE3982_MEMORY_CD4_TCELL_VS_TH1_DN | 96 | -5.13E-01 | -1.8772459 | 0.00E+00 | 2.40E-02 |
| GSE36476_YOUNG_VS_OLD_DONOR_MEMORY_CD4_TCELL_72H_TSST_ACT_DN | 72 | -5.36E-01 | -1.8753463 | 1.40E-03 | 2.46E-02 |
| GU_PDEF_TARGETS_DN | 17 | -7.11E-01 | -1.8752224 | 0.00E+00 | 2.45E-02 |
| VANTVEER_BREAST_CANCER_METASTASIS_DN | 57 | -5.54E-01 | -1.8746576 | 0.00E+00 | 2.46E-02 |
| KAN_RESPONSE_TO_ARSENIC_TRIOXIDE | 54 | -5.52E-01 | -1.8736297 | 0.00E+00 | 2.49E-02 |
| **KOBAYASHI_EGFR_SIGNALING_24HR_DN** | **121** | **-4.92E-01** | **-1.8734758** | **0.00E+00** | **2.48E-02** |
| CHIANG_LIVER_CANCER_SUBCLASS_CTNNB1_DN | 60 | -5.52E-01 | -1.8731779 | 0.00E+00 | 2.49E-02 |
| GSE19923_WT_VS_HEB_AND_E2A_KO_DP_THYMOCYTE_DN | 83 | -5.25E-01 | -1.8726771 | 0.00E+00 | 2.50E-02 |
| GSE41978_ID2_KO_AND_BIM_KO_VS_BIM_KO_KLRG1_LOW_EFFECTOR_CD8_TCELL_DN | 84 | -5.25E-01 | -1.8717263 | 0.00E+00 | 2.52E-02 |
| SMID_BREAST_CANCER_LUMINAL_B_DN | 175 | -4.68E-01 | -1.8716303 | 0.00E+00 | 2.52E-02 |
| LEF1_UP.V1_DN | 97 | -5.15E-01 | -1.8713955 | 0.00E+00 | 2.52E-02 |
| GSE22313_HEALTHY_VS_SLE_MOUSE_CD4_TCELL_UP | 83 | -5.22E-01 | -1.8713686 | 0.00E+00 | 2.51E-02 |
| WINTER_HYPOXIA_METAGENE | 103 | -5.02E-01 | -1.8705056 | 0.00E+00 | 2.53E-02 |
| **ST_ERK1_ERK2_MAPK_PATHWAY** | **13** | **-7.78E-01** | **-1.8694057** | **3.12E-03** | **2.56E-02** |
| BERENJENO_ROCK_SIGNALING_NOT_VIA_RHOA_DN | 18 | -7.01E-01 | -1.8691033 | 1.64E-03 | 2.56E-02 |
| GSE21546_ELK1_KO_VS_SAP1A_KO_AND_ELK1_KO_ANTI_CD3_STIM_DP_THYMOCYTES_DN | 83 | -5.30E-01 | -1.8678399 | 0.00E+00 | 2.60E-02 |
| KRIEG_HYPOXIA_VIA_KDM3A | 20 | -6.93E-01 | -1.8671149 | 3.23E-03 | 2.61E-02 |
| GO_SULFUR_COMPOUND_TRANSPORT | 13 | -7.67E-01 | -1.8668351 | 3.42E-03 | 2.61E-02 |
| GGGNRMNNYCAT_UNKNOWN | 21 | -6.69E-01 | -1.8662786 | 1.58E-03 | 2.62E-02 |
| GSE30971_CTRL_VS_LPS_STIM_MACROPHAGE_WBP7_KO_2H_UP | 68 | -5.40E-01 | -1.8658422 | 0.00E+00 | 2.63E-02 |
| GO_NEGATIVE_REGULATION_OF_MAP_KINASE_ACTIVITY | 30 | -6.23E-01 | -1.8648443 | 0.00E+00 | 2.65E-02 |
| ZHAN_MULTIPLE_MYELOMA_MF_DN | 13 | -7.48E-01 | -1.8646778 | 1.70E-03 | 2.65E-02 |
| GSE9988_LOW_LPS_VS_VEHICLE_TREATED_MONOCYTE_UP | 81 | -5.28E-01 | -1.8645196 | 0.00E+00 | 2.65E-02 |
| P53_DN.V1_DN | 64 | -5.36E-01 | -1.8643394 | 0.00E+00 | 2.64E-02 |
| RUIZ_TNC_TARGETS_UP | 61 | -5.49E-01 | -1.8642286 | 0.00E+00 | 2.64E-02 |
| GSE2706_UNSTIM_VS_8H_LPS_AND_R848_DC_DN | 86 | -5.20E-01 | -1.8639196 | 0.00E+00 | 2.64E-02 |
| GO_POSITIVE_REGULATION_OF_PROTEIN_TARGETING_TO_MEMBRANE | 10 | -8.11E-01 | -1.8627448 | 0.00E+00 | 2.67E-02 |
| CREIGHTON_ENDOCRINE_THERAPY_RESISTANCE_5 | 198 | -4.64E-01 | -1.8622965 | 0.00E+00 | 2.68E-02 |
| **HALLMARK_KRAS_SIGNALING_UP** | **63** | **-5.40E-01** | **-1.8621234** | **0.00E+00** | **2.68E-02** |
| GSE42088_UNINF_VS_LEISHMANIA_INF_DC_2H_UP | 80 | -5.22E-01 | -1.861883 | 0.00E+00 | 2.68E-02 |
| GSE25085_FETAL_BM_VS_ADULT_BM_SP4_THYMIC_IMPLANT_DN | 79 | -5.24E-01 | -1.8618162 | 0.00E+00 | 2.67E-02 |
| AATGTGA_MIR23A_MIR23B | 127 | -4.86E-01 | -1.8616685 | 0.00E+00 | 2.67E-02 |
| GO_REGULATION_OF_PROTEIN_TARGETING_TO_MEMBRANE | 13 | -7.53E-01 | -1.8609992 | 0.00E+00 | 2.67E-02 |
| DORN_ADENOVIRUS_INFECTION_24HR_DN | 22 | -6.68E-01 | -1.86022 | 1.56E-03 | 2.69E-02 |
| DELPUECH_FOXO3_TARGETS_UP | 26 | -6.51E-01 | -1.8593423 | 3.11E-03 | 2.71E-02 |
| GSE9988_LPS_VS_VEHICLE_TREATED_MONOCYTE_UP | 79 | -5.19E-01 | -1.8591198 | 0.00E+00 | 2.71E-02 |
| GSE4748_LPS_VS_LPS_AND_CYANOBACTERIUM_LPSLIKE_STIM_DC_3H_UP | 73 | -5.28E-01 | -1.8586795 | 0.00E+00 | 2.72E-02 |
| GSE3982_DC_VS_TH2_DN | 80 | -5.23E-01 | -1.8586453 | 0.00E+00 | 2.71E-02 |
| GSE2706_R848_VS_R848_AND_LPS_2H_STIM_DC_DN | 73 | -5.20E-01 | -1.8584412 | 0.00E+00 | 2.71E-02 |
| GSE42021_TCONV_PLN_VS_CD24HI_TCONV_THYMUS_DN | 96 | -5.03E-01 | -1.8583239 | 0.00E+00 | 2.71E-02 |
| GO_RESPONSE_TO_AMINO_ACID | 45 | -5.76E-01 | -1.8580729 | 0.00E+00 | 2.71E-02 |
| LINDGREN_BLADDER_CANCER_CLUSTER_3_DN | 102 | -5.05E-01 | -1.8577048 | 0.00E+00 | 2.71E-02 |
| REACTOME_TOLL_RECEPTOR_CASCADES | 35 | -6.03E-01 | -1.8569748 | 1.54E-03 | 2.74E-02 |
| GSE24081_CONTROLLER_VS_PROGRESSOR_HIV_SPECIFIC_CD8_TCELL_UP | 73 | -5.21E-01 | -1.8565245 | 0.00E+00 | 2.75E-02 |
| GO_CELLULAR_RESPONSE_TO_ACID_CHEMICAL | 63 | -5.43E-01 | -1.8556062 | 0.00E+00 | 2.77E-02 |
| GSE24726_WT_VS_E2-2_KO_PDC_DAY4_POST_DELETION_DN | 81 | -5.20E-01 | -1.8543712 | 0.00E+00 | 2.81E-02 |
| RODWELL_AGING_KIDNEY_NO_BLOOD_UP | 77 | -5.24E-01 | -1.8533973 | 0.00E+00 | 2.84E-02 |
| STK33_NOMO_DN | 109 | -4.99E-01 | -1.852945 | 0.00E+00 | 2.84E-02 |
| KIM_RESPONSE_TO_TSA_AND_DECITABINE_UP | 56 | -5.51E-01 | -1.852624 | 0.00E+00 | 2.85E-02 |
| PAX2_02 | 101 | -5.04E-01 | -1.8523165 | 0.00E+00 | 2.85E-02 |
| WAMUNYOKOLI_OVARIAN_CANCER_LMP_DN | 71 | -5.27E-01 | -1.8523139 | 0.00E+00 | 2.84E-02 |
| HUANG_FOXA2_TARGETS_UP | 20 | -6.89E-01 | -1.8522155 | 4.77E-03 | 2.84E-02 |
| FRIDMAN_SENESCENCE_UP | 28 | -6.27E-01 | -1.8511561 | 0.00E+00 | 2.87E-02 |
| GO_VASCULATURE_DEVELOPMENT | 177 | -4.65E-01 | -1.849999 | 0.00E+00 | 2.91E-02 |
| GO_EXTRACELLULAR_MATRIX_STRUCTURAL_CONSTITUENT | 28 | -6.29E-01 | -1.8498375 | 3.13E-03 | 2.90E-02 |
| LIN_SILENCED_BY_TUMOR_MICROENVIRONMENT | 33 | -5.99E-01 | -1.8498244 | 0.00E+00 | 2.89E-02 |
| GSE17974_2H_VS_72H_UNTREATED_IN_VITRO_CD4_TCELL_UP | 92 | -5.07E-01 | -1.8496776 | 0.00E+00 | 2.89E-02 |
| **RAF_UP.V1_DN** | **68** | **-5.35E-01** | **-1.8489525** | **0.00E+00** | **2.91E-02** |
| CAMP_UP.V1_DN | 72 | -5.28E-01 | -1.8488386 | 1.37E-03 | 2.90E-02 |
| CHR11Q22 | 13 | -7.48E-01 | -1.8484707 | 1.67E-03 | 2.91E-02 |
| REACTOME_EXTRACELLULAR_MATRIX_ORGANIZATION | 27 | -6.37E-01 | -1.847815 | 0.00E+00 | 2.93E-02 |
| RAO_BOUND_BY_SALL4_ISOFORM_A | 51 | -5.53E-01 | -1.8472518 | 1.46E-03 | 2.94E-02 |
| PAX4_04 | 61 | -5.38E-01 | -1.846983 | 0.00E+00 | 2.94E-02 |
| ENK_UV_RESPONSE_KERATINOCYTE_DN | 186 | -4.61E-01 | -1.8460994 | 0.00E+00 | 2.97E-02 |
| CHIARADONNA_NEOPLASTIC_TRANSFORMATION_KRAS_DN | 66 | -5.31E-01 | -1.845954 | 0.00E+00 | 2.96E-02 |
| SINGH_KRAS_DEPENDENCY_SIGNATURE_ | 13 | -7.68E-01 | -1.844599 | 0.00E+00 | 3.01E-02 |
| GO_RESPONSE_TO_ENDOPLASMIC_RETICULUM_STRESS | 92 | -5.04E-01 | -1.8431423 | 0.00E+00 | 3.06E-02 |
| GILDEA_METASTASIS | 15 | -7.29E-01 | -1.8431053 | 3.36E-03 | 3.05E-02 |
| CORDENONSI_YAP_CONSERVED_SIGNATURE | 23 | -6.64E-01 | -1.8425796 | 1.55E-03 | 3.06E-02 |
| LEONARD_HYPOXIA | 24 | -6.54E-01 | -1.8417861 | 3.08E-03 | 3.08E-02 |
| GO_REGULATION_OF_PEPTIDYL_THREONINE_PHOSPHORYLATION | 15 | -7.24E-01 | -1.8416866 | 4.93E-03 | 3.08E-02 |
| MARZEC_IL2_SIGNALING_UP | 51 | -5.60E-01 | -1.841651 | 1.47E-03 | 3.07E-02 |
| GO_COLLAGEN_FIBRIL_ORGANIZATION | 12 | -7.69E-01 | -1.8414199 | 0.00E+00 | 3.07E-02 |
| SMID_BREAST_CANCER_RELAPSE_IN_BONE_UP | 39 | -5.86E-01 | -1.8410891 | 1.50E-03 | 3.07E-02 |
| BROWNE_HCMV_INFECTION_12HR_DN | 37 | -5.96E-01 | -1.8403472 | 1.61E-03 | 3.09E-02 |
| GO_EMBRYONIC_MORPHOGENESIS | 207 | -4.63E-01 | -1.8401253 | 0.00E+00 | 3.09E-02 |
| GSE42021_CD24HI_VS_CD24LOW_TCONV_THYMUS_DN | 76 | -5.24E-01 | -1.83999 | 1.37E-03 | 3.09E-02 |
| RHEIN_ALL_GLUCOCORTICOID_THERAPY_UP | 20 | -6.78E-01 | -1.8396034 | 0.00E+00 | 3.10E-02 |
| REACTOME_MAPK_TARGETS_NUCLEAR_EVENTS_MEDIATED_BY_MAP_KINASES | 10 | -8.12E-01 | -1.8379596 | 0.00E+00 | 3.15E-02 |
| GSE45365_WT_VS_IFNAR_KO_CD8A_DC_DN | 81 | -5.07E-01 | -1.8374728 | 0.00E+00 | 3.16E-02 |
| RODRIGUES_THYROID_CARCINOMA_POORLY_DIFFERENTIATED_DN | 261 | -4.46E-01 | -1.837253 | 0.00E+00 | 3.15E-02 |
| BERENJENO_TRANSFORMED_BY_RHOA_FOREVER_DN | 10 | -8.10E-01 | -1.8370922 | 1.71E-03 | 3.15E-02 |
| GSE2770_TGFB_AND_IL4_VS_TGFB_AND_IL12_TREATED_ACT_CD4_TCELL_6H_DN | 71 | -5.13E-01 | -1.8368082 | 0.00E+00 | 3.15E-02 |
| ALCALAY_AML_BY_NPM1_LOCALIZATION_UP | 53 | -5.51E-01 | -1.8349954 | 1.46E-03 | 3.22E-02 |
| GSE25123_CTRL_VS_IL4_STIM_MACROPHAGE_DN | 90 | -5.02E-01 | -1.8339843 | 0.00E+00 | 3.26E-02 |
| GSE19923_HEB_KO_VS_HEB_AND_E2A_KO_DP_THYMOCYTE_DN | 77 | -5.16E-01 | -1.8330375 | 0.00E+00 | 3.29E-02 |
| STK33_SKM_UP | 110 | -4.96E-01 | -1.8320887 | 1.32E-03 | 3.32E-02 |
| HALLMARK_ESTROGEN_RESPONSE_EARLY | 71 | -5.24E-01 | -1.8314571 | 0.00E+00 | 3.34E-02 |
| KRIGE_AMINO_ACID_DEPRIVATION | 14 | -7.06E-01 | -1.8303016 | 3.23E-03 | 3.39E-02 |
| RODRIGUES_NTN1_TARGETS_DN | 52 | -5.58E-01 | -1.8300152 | 2.94E-03 | 3.39E-02 |
| HALLMARK_P53_PATHWAY | 97 | -5.03E-01 | -1.8296258 | 0.00E+00 | 3.40E-02 |
| IZADPANAH_STEM_CELL_ADIPOSE_VS_BONE_DN | 40 | -5.81E-01 | -1.8293355 | 1.53E-03 | 3.40E-02 |
| CREIGHTON_ENDOCRINE_THERAPY_RESISTANCE_3 | 273 | -4.38E-01 | -1.8292496 | 0.00E+00 | 3.40E-02 |
| KEGG_PHENYLALANINE_METABOLISM | 10 | -8.09E-01 | -1.8290017 | 5.27E-03 | 3.40E-02 |
| GO_BRANCHING_MORPHOGENESIS_OF_AN_EPITHELIAL_TUBE | 51 | -5.51E-01 | -1.8289608 | 2.97E-03 | 3.39E-02 |
| GSE6674_CPG_VS_CPG_AND_ANTI_IGM_STIM_BCELL_DN | 90 | -4.99E-01 | -1.8289337 | 0.00E+00 | 3.38E-02 |
| MIYAGAWA_TARGETS_OF_EWSR1_ETS_FUSIONS_DN | 83 | -5.13E-01 | -1.8288581 | 1.43E-03 | 3.38E-02 |
| NAKAMURA_ADIPOGENESIS_EARLY_DN | 13 | -7.36E-01 | -1.8286185 | 4.78E-03 | 3.38E-02 |
| DAVICIONI_RHABDOMYOSARCOMA_PAX_FOXO1_FUSION_UP | 28 | -6.23E-01 | -1.8283902 | 1.54E-03 | 3.38E-02 |
| KINSEY_TARGETS_OF_EWSR1_FLII_FUSION_DN | 117 | -4.82E-01 | -1.8277583 | 1.33E-03 | 3.40E-02 |
| WU_SILENCED_BY_METHYLATION_IN_BLADDER_CANCER | 17 | -6.97E-01 | -1.8273699 | 0.00E+00 | 3.41E-02 |
| GSE5455_HEALTHY_VS_TUMOR_BEARING_MOUSE_SPLEEN_MONOCYTE_24H_INCUBATION_DN | 79 | -5.09E-01 | -1.8273685 | 0.00E+00 | 3.40E-02 |
| GSE6259_33D1_POS_VS_DEC205_POS_FLT3L_INDUCED_SPLENIC_DC_UP | 60 | -5.32E-01 | -1.8272513 | 0.00E+00 | 3.39E-02 |
| GO_REPRODUCTIVE_SYSTEM_DEVELOPMENT | 142 | -4.69E-01 | -1.8268926 | 0.00E+00 | 3.40E-02 |
| ZHANG_PROLIFERATING_VS_QUIESCENT | 23 | -6.37E-01 | -1.8265634 | 0.00E+00 | 3.40E-02 |
| GO_RESPONSE_TO_ACID_CHEMICAL | 123 | -4.77E-01 | -1.8252336 | 0.00E+00 | 3.45E-02 |
| PETROVA_ENDOTHELIUM_LYMPHATIC_VS_BLOOD_UP | 50 | -5.56E-01 | -1.8251162 | 2.97E-03 | 3.45E-02 |
| GSE360_L_DONOVANI_VS_M_TUBERCULOSIS_MAC_DN | 80 | -5.18E-01 | -1.824915 | 0.00E+00 | 3.45E-02 |
| SMIRNOV_RESPONSE_TO_IR_6HR_UP | 83 | -5.06E-01 | -1.8240513 | 0.00E+00 | 3.47E-02 |
| RADMACHER_AML_PROGNOSIS | 25 | -6.42E-01 | -1.8238732 | 4.53E-03 | 3.47E-02 |
| GSE37301_PRO_BCELL_VS_GRANULOCYTE_MONOCYTE_PROGENITOR_UP | 59 | -5.35E-01 | -1.8238547 | 0.00E+00 | 3.46E-02 |
| GSE2706_UNSTIM_VS_2H_LPS_DC_DN | 78 | -5.08E-01 | -1.8237356 | 1.38E-03 | 3.46E-02 |
| GO_WNT_PROTEIN_BINDING | 14 | -7.26E-01 | -1.8233078 | 3.41E-03 | 3.47E-02 |
| CYCLIN_D1_KE_.V1_UP | 96 | -4.95E-01 | -1.8228528 | 0.00E+00 | 3.48E-02 |
| GO_CANONICAL_WNT_SIGNALING_PATHWAY | 34 | -5.92E-01 | -1.8227869 | 1.59E-03 | 3.47E-02 |
| DURCHDEWALD_SKIN_CARCINOGENESIS_DN | 92 | -5.03E-01 | -1.8222175 | 0.00E+00 | 3.49E-02 |
| GO_EXTRACELLULAR_STRUCTURE_ORGANIZATION | 130 | -4.71E-01 | -1.8219006 | 0.00E+00 | 3.50E-02 |
| ZHANG_RESPONSE_TO_IKK_INHIBITOR_AND_TNF_UP | 90 | -5.04E-01 | -1.8214968 | 0.00E+00 | 3.51E-02 |
| GO_SUBSTRATE_DEPENDENT_CELL_MIGRATION | 15 | -7.12E-01 | -1.8213885 | 1.62E-03 | 3.51E-02 |
| GSE42021_CD24HI_TREG_VS_CD24HI_TCONV_THYMUS_DN | 78 | -5.12E-01 | -1.8213313 | 0.00E+00 | 3.50E-02 |
| MODULE_122 | 60 | -5.30E-01 | -1.8205646 | 1.43E-03 | 3.52E-02 |
| VANHARANTA_UTERINE_FIBROID_UP | 21 | -6.55E-01 | -1.8202906 | 0.00E+00 | 3.53E-02 |
| WONG_ADULT_TISSUE_STEM_MODULE | 268 | -4.37E-01 | -1.8194582 | 0.00E+00 | 3.55E-02 |
| GSE42021_CD24HI_VS_CD24INT_TCONV_THYMUS_UP | 83 | -5.02E-01 | -1.819424 | 0.00E+00 | 3.55E-02 |
| LU_TUMOR_VASCULATURE_UP | 13 | -7.47E-01 | -1.8194122 | 0.00E+00 | 3.54E-02 |
| ONDER_CDH1_SIGNALING_VIA_CTNNB1 | 45 | -5.65E-01 | -1.8186944 | 5.55E-03 | 3.56E-02 |
| GSE3982_CTRL_VS_LPS_4H_MAC_DN | 79 | -5.05E-01 | -1.8186587 | 1.40E-03 | 3.55E-02 |
| BURTON_ADIPOGENESIS_9 | 35 | -5.93E-01 | -1.8184685 | 4.56E-03 | 3.55E-02 |
| GSE10422_WT_VS_BAFF_TRANSGENIC_LN_BCELL_UP | 66 | -5.23E-01 | -1.8181921 | 0.00E+00 | 3.55E-02 |
| GSE36476_YOUNG_VS_OLD_DONOR_MEMORY_CD4_TCELL_40H_TSST_ACT_DN | 94 | -5.02E-01 | -1.817188 | 0.00E+00 | 3.58E-02 |
| NABA_CORE_MATRISOME | 102 | -4.88E-01 | -1.817131 | 0.00E+00 | 3.58E-02 |
| SOTIRIOU_BREAST_CANCER_GRADE_1_VS_3_DN | 20 | -6.77E-01 | -1.8169848 | 3.22E-03 | 3.58E-02 |
| JOHANSSON_GLIOMAGENESIS_BY_PDGFB_UP | 20 | -6.81E-01 | -1.8167166 | 6.38E-03 | 3.58E-02 |
| AACTGGA_MIR145 | 75 | -5.08E-01 | -1.8166056 | 1.40E-03 | 3.58E-02 |
| BERENJENO_TRANSFORMED_BY_RHOA_REVERSIBLY_DN | 16 | -7.09E-01 | -1.8159282 | 3.20E-03 | 3.60E-02 |
| RUTELLA_RESPONSE_TO_CSF2RB_AND_IL4_UP | 136 | -4.68E-01 | -1.8150007 | 1.30E-03 | 3.64E-02 |
| PLASARI_TGFB1_TARGETS_1HR_UP | 16 | -7.07E-01 | -1.8139787 | 4.89E-03 | 3.69E-02 |
| GO_COLLAGEN_TRIMER | 25 | -6.45E-01 | -1.8138741 | 1.61E-03 | 3.68E-02 |
| CHICAS_RB1_TARGETS_CONFLUENT | 229 | -4.43E-01 | -1.8137722 | 0.00E+00 | 3.68E-02 |
| WATANABE_RECTAL_CANCER_RADIOTHERAPY_RESPONSIVE_DN | 35 | -5.82E-01 | -1.8130109 | 6.07E-03 | 3.71E-02 |
| GSE10325_CD4_TCELL_VS_LUPUS_CD4_TCELL_DN | 75 | -5.14E-01 | -1.8129287 | 0.00E+00 | 3.70E-02 |
| **PEDERSEN_METASTASIS_BY_ERBB2_ISOFORM_7** | **142** | **-4.69E-01** | **-1.8124276** | **0.00E+00** | **3.72E-02** |
| ZWANG_CLASS_1_TRANSIENTLY_INDUCED_BY_EGF | 209 | -4.49E-01 | -1.8123295 | 0.00E+00 | 3.71E-02 |
| KIM_GLIS2_TARGETS_UP | 38 | -5.72E-01 | -1.8111308 | 4.43E-03 | 3.76E-02 |
| BROWNE_HCMV_INFECTION_6HR_DN | 76 | -5.10E-01 | -1.8106319 | 1.35E-03 | 3.77E-02 |
| RIGGI_EWING_SARCOMA_PROGENITOR_DN | 69 | -5.24E-01 | -1.810588 | 0.00E+00 | 3.77E-02 |
| GSE19401_NAIVE_VS_IMMUNIZED_MOUSE_PLN_FOLLICULAR_DC_UP | 79 | -5.11E-01 | -1.8105159 | 0.00E+00 | 3.76E-02 |
| GSE18804_SPLEEN_MACROPHAGE_VS_BRAIN_TUMORAL_MACROPHAGE_DN | 87 | -4.98E-01 | -1.8100959 | 0.00E+00 | 3.77E-02 |
| GRAESSMANN_APOPTOSIS_BY_SERUM_DEPRIVATION_UP | 226 | -4.43E-01 | -1.8100189 | 0.00E+00 | 3.77E-02 |
| GNF2_CDH11 | 10 | -7.95E-01 | -1.8099741 | 3.43E-03 | 3.76E-02 |
| GSE6674_CPG_VS_PL2_3_STIM_BCELL_DN | 83 | -5.05E-01 | -1.8092468 | 0.00E+00 | 3.79E-02 |
| GSE24634_TREG_VS_TCONV_POST_DAY7_IL4_CONVERSION_DN | 74 | -5.19E-01 | -1.8091352 | 1.38E-03 | 3.78E-02 |
| GSE19888_CTRL_VS_A3R_INHIBITOR_TREATED_MAST_CELL_DN | 76 | -5.13E-01 | -1.8090942 | 0.00E+00 | 3.77E-02 |
| REACTOME_NCAM1_INTERACTIONS | 18 | -6.90E-01 | -1.8090185 | 4.84E-03 | 3.77E-02 |
| GO_ENDODERM_DEVELOPMENT | 31 | -5.97E-01 | -1.8088409 | 6.31E-03 | 3.77E-02 |
| TIEN_INTESTINE_PROBIOTICS_6HR_UP | 13 | -7.23E-01 | -1.8086014 | 3.29E-03 | 3.77E-02 |
| TAKEDA_TARGETS_OF_NUP98_HOXA9_FUSION_8D_UP | 56 | -5.40E-01 | -1.8085185 | 1.46E-03 | 3.76E-02 |
| GSE27670_CTRL_VS_BLIMP1_TRANSDUCED_GC_BCELL_DN | 73 | -5.13E-01 | -1.8082392 | 1.38E-03 | 3.77E-02 |
| VANHARANTA_UTERINE_FIBROID_DN | 26 | -6.26E-01 | -1.808146 | 3.12E-03 | 3.76E-02 |
| GSE3982_BASOPHIL_VS_EFF_MEMORY_CD4_TCELL_UP | 82 | -5.09E-01 | -1.8071668 | 0.00E+00 | 3.80E-02 |
| REACTOME_NCAM_SIGNALING_FOR_NEURITE_OUT_GROWTH | 24 | -6.44E-01 | -1.8070529 | 4.82E-03 | 3.79E-02 |
| KEGG_FOCAL_ADHESION | 77 | -5.03E-01 | -1.8068054 | 0.00E+00 | 3.80E-02 |
| GSE339_EX_VIVO_VS_IN_CULTURE_CD8POS_DC_DN | 85 | -5.05E-01 | -1.8056785 | 0.00E+00 | 3.84E-02 |
| GSE18893_TCONV_VS_TREG_2H_TNF_STIM_DN | 75 | -5.14E-01 | -1.8054849 | 0.00E+00 | 3.83E-02 |
| GSE3982_MAC_VS_TH2_UP | 78 | -5.13E-01 | -1.804905 | 0.00E+00 | 3.86E-02 |
| GSE14000_4H_VS_16H_LPS_DC_TRANSLATED_RNA_UP | 86 | -4.90E-01 | -1.8048954 | 0.00E+00 | 3.85E-02 |
| ATATGCA_MIR448 | 51 | -5.46E-01 | -1.8045343 | 0.00E+00 | 3.86E-02 |
| GO_WNT_ACTIVATED_RECEPTOR_ACTIVITY | 10 | -7.83E-01 | -1.8044026 | 1.77E-03 | 3.86E-02 |
| GSE3203_INFLUENZA_INF_VS_IFNB_TREATED_LN_BCELL_DN | 82 | -5.02E-01 | -1.8035941 | 1.40E-03 | 3.89E-02 |
| KOYAMA_SEMA3B_TARGETS_UP | 114 | -4.77E-01 | -1.8035889 | 0.00E+00 | 3.88E-02 |
| KRAS.LUNG.BREAST_UP.V1_UP | 36 | -5.84E-01 | -1.8015193 | 3.13E-03 | 3.97E-02 |
| MODULE_19 | 113 | -4.77E-01 | -1.8012781 | 0.00E+00 | 3.97E-02 |
| ONDER_CDH1_TARGETS_2_UP | 98 | -4.95E-01 | -1.8005657 | 0.00E+00 | 4.00E-02 |
| **NAKAYAMA_FGF2_TARGETS** | **11** | **-7.75E-01** | **-1.8001707** | **3.37E-03** | **4.01E-02** |
| GSE17974_0.5H_VS_72H_IL4_AND_ANTI_IL12_ACT_CD4_TCELL_UP | 91 | -4.93E-01 | -1.800141 | 0.00E+00 | 4.00E-02 |
| TIAN_TNF_SIGNALING_VIA_NFKB | 15 | -7.07E-01 | -1.7995383 | 1.72E-03 | 4.02E-02 |
| RAO_BOUND_BY_SALL4 | 73 | -5.14E-01 | -1.7993175 | 0.00E+00 | 4.03E-02 |
| DAZARD_RESPONSE_TO_UV_SCC_DN | 45 | -5.60E-01 | -1.7990885 | 1.48E-03 | 4.03E-02 |
| GSE13485_PRE_VS_POST_YF17D_VACCINATION_PBMC_UP | 65 | -5.17E-01 | -1.7988871 | 1.42E-03 | 4.03E-02 |
| CASORELLI_APL_SECONDARY_VS_DE_NOVO_UP | 18 | -6.84E-01 | -1.7985197 | 3.26E-03 | 4.04E-02 |
| GSE11057_EFF_MEM_VS_CENT_MEM_CD4_TCELL_UP | 81 | -4.94E-01 | -1.7981842 | 0.00E+00 | 4.05E-02 |
| GSE6259_33D1_POS_VS_DEC205_POS_SPLENIC_DC_DN | 78 | -5.05E-01 | -1.7965703 | 2.77E-03 | 4.12E-02 |
| GSE360_T_GONDII_VS_M_TUBERCULOSIS_MAC_UP | 93 | -4.95E-01 | -1.7954803 | 0.00E+00 | 4.17E-02 |
| SENGUPTA_NASOPHARYNGEAL_CARCINOMA_UP | 97 | -4.90E-01 | -1.7945255 | 0.00E+00 | 4.21E-02 |
| TURASHVILI_BREAST_LOBULAR_CARCINOMA_VS_LOBULAR_NORMAL_DN | 20 | -6.54E-01 | -1.794343 | 4.76E-03 | 4.21E-02 |
| BLALOCK_ALZHEIMERS_DISEASE_INCIPIENT_DN | 65 | -5.15E-01 | -1.7939992 | 0.00E+00 | 4.22E-02 |
| GSE16755_CTRL_VS_IFNA_TREATED_MAC_DN | 76 | -5.03E-01 | -1.7936398 | 1.37E-03 | 4.23E-02 |
| FULCHER_INFLAMMATORY_RESPONSE_LECTIN_VS_LPS_UP | 212 | -4.44E-01 | -1.7934296 | 0.00E+00 | 4.23E-02 |
| GSE10094_LCMV_VS_LISTERIA_IND_EFF_CD4_TCELL_UP | 80 | -5.11E-01 | -1.7931912 | 1.39E-03 | 4.23E-02 |
| RICKMAN_TUMOR_DIFFERENTIATED_WELL_VS_POORLY_UP | 81 | -5.02E-01 | -1.793062 | 0.00E+00 | 4.22E-02 |
| LIM_MAMMARY_STEM_CELL_UP | 193 | -4.46E-01 | -1.7928922 | 0.00E+00 | 4.23E-02 |
| TBK1.DF_UP | 147 | -4.56E-01 | -1.792095 | 0.00E+00 | 4.26E-02 |
| CAGTGTT_MIR141_MIR200A | 90 | -4.92E-01 | -1.7916492 | 0.00E+00 | 4.27E-02 |
| GO_POSITIVE_REGULATION_OF_RELEASE_OF_SEQUESTERED_CALCIUM_ION_INTO_CYTOSOL | 17 | -6.79E-01 | -1.7912273 | 4.72E-03 | 4.28E-02 |
| TACTTGA_MIR26A_MIR26B | 107 | -4.79E-01 | -1.790909 | 0.00E+00 | 4.29E-02 |
| GSE43955_1H_VS_60H_ACT_CD4_TCELL_DN | 77 | -5.08E-01 | -1.790454 | 0.00E+00 | 4.30E-02 |
| GSE2770_UNTREATED_VS_ACT_CD4_TCELL_48H_DN | 95 | -4.84E-01 | -1.7902282 | 0.00E+00 | 4.31E-02 |
| GSE37605_C57BL6_VS_NOD_FOXP3_IRES_GFP_TCONV_DN | 66 | -5.12E-01 | -1.7899926 | 1.43E-03 | 4.31E-02 |
| SESTO_RESPONSE_TO_UV_C2 | 19 | -6.61E-01 | -1.7897319 | 3.28E-03 | 4.31E-02 |
| GSE36392_TYPE_2_MYELOID_VS_MAC_IL25_TREATED_LUNG_UP | 63 | -5.24E-01 | -1.7894789 | 1.43E-03 | 4.32E-02 |
| PEDERSEN_METASTASIS_BY_ERBB2_ISOFORM_4 | 43 | -5.55E-01 | -1.7894019 | 0.00E+00 | 4.31E-02 |
| SENESE_HDAC2_TARGETS_DN | 52 | -5.32E-01 | -1.7891601 | 0.00E+00 | 4.32E-02 |
| SNIJDERS_AMPLIFIED_IN_HEAD_AND_NECK_TUMORS | 10 | -7.81E-01 | -1.7889296 | 3.37E-03 | 4.32E-02 |
| PHONG_TNF_TARGETS_UP | 31 | -6.03E-01 | -1.7888192 | 4.67E-03 | 4.32E-02 |
| GSE41176_WT_VS_TAK1_KO_ANTI_IGM_STIM_BCELL_24H_UP | 81 | -5.02E-01 | -1.7880762 | 0.00E+00 | 4.34E-02 |
| GSE40274_CTRL_VS_FOXP3_TRANSDUCED_ACTIVATED_CD4_TCELL_DN | 81 | -5.00E-01 | -1.7867956 | 0.00E+00 | 4.40E-02 |
| MANTOVANI_VIRAL_GPCR_SIGNALING_UP | 29 | -6.14E-01 | -1.786175 | 0.00E+00 | 4.42E-02 |
| GSE24142_EARLY_THYMIC_PROGENITOR_VS_DN3_THYMOCYTE_FETAL_UP | 81 | -4.95E-01 | -1.7861221 | 0.00E+00 | 4.42E-02 |
| GSE17721_LPS_VS_PAM3CSK4_1H_BMDC_DN | 78 | -5.10E-01 | -1.7857301 | 2.81E-03 | 4.43E-02 |
| GSE14769_UNSTIM_VS_20MIN_LPS_BMDM_DN | 73 | -5.18E-01 | -1.7854654 | 0.00E+00 | 4.43E-02 |
| GOZGIT_ESR1_TARGETS_DN | 259 | -4.33E-01 | -1.7851847 | 0.00E+00 | 4.44E-02 |
| GSE37301_MULTIPOTENT_PROGENITOR_VS_PRO_BCELL_DN | 70 | -5.05E-01 | -1.7848586 | 0.00E+00 | 4.45E-02 |
| HNF1_C | 66 | -5.09E-01 | -1.7840631 | 0.00E+00 | 4.48E-02 |
| GSE42021_TREG_PLN_VS_CD24INT_TREG_THYMUS_UP | 66 | -5.17E-01 | -1.783947 | 0.00E+00 | 4.48E-02 |
| GO_AORTA_DEVELOPMENT | 14 | -7.27E-01 | -1.7839305 | 6.44E-03 | 4.47E-02 |
| GSE37336_LY6C_POS_VS_NEG_NAIVE_CD4_TCELL_UP | 62 | -5.23E-01 | -1.7830632 | 2.85E-03 | 4.50E-02 |
| BASAKI_YBX1_TARGETS_DN | 127 | -4.71E-01 | -1.7830325 | 0.00E+00 | 4.50E-02 |
| DAVICIONI_PAX_FOXO1_SIGNATURE_IN_ARMS_UP | 23 | -6.25E-01 | -1.7825857 | 6.23E-03 | 4.51E-02 |
| GSE3920_UNTREATED_VS_IFNA_TREATED_FIBROBLAST_UP | 76 | -5.02E-01 | -1.7825593 | 0.00E+00 | 4.50E-02 |
| GSE37533_UNTREATED_VS_PIOGLIZATONE_TREATED_CD4_TCELL_PPARG1_AND_FOXP3_TRASDUCED_UP | 71 | -5.12E-01 | -1.782159 | 2.82E-03 | 4.52E-02 |
| GO_NOTCH_BINDING | 10 | -7.87E-01 | -1.7819016 | 6.71E-03 | 4.52E-02 |
| MODULE_5 | 159 | -4.51E-01 | -1.7808185 | 0.00E+00 | 4.56E-02 |
| ZHANG_GATA6_TARGETS_DN | 25 | -6.21E-01 | -1.7797071 | 7.58E-03 | 4.62E-02 |
| GO_SENSORY_ORGAN_MORPHOGENESIS | 85 | -5.00E-01 | -1.7796088 | 1.37E-03 | 4.61E-02 |
| ONGUSAHA_TP53_TARGETS | 18 | -6.64E-01 | -1.7792788 | 1.15E-02 | 4.62E-02 |
| ESC_V6.5_UP_EARLY.V1_DN | 63 | -5.22E-01 | -1.7790167 | 1.43E-03 | 4.62E-02 |
| CGTSACG_PAX3_B | 25 | -6.15E-01 | -1.7790054 | 1.50E-03 | 4.61E-02 |
| GSE17974_IL4_AND_ANTI_IL12_VS_UNTREATED_48H_ACT_CD4_TCELL_DN | 59 | -5.22E-01 | -1.7784505 | 2.95E-03 | 4.64E-02 |
| GSE32986_UNSTIM_VS_GMCSF_AND_CURDLAN_LOWDOSE_STIM_DC_DN | 74 | -5.01E-01 | -1.7782584 | 1.36E-03 | 4.64E-02 |
| GSE23502_BM_VS_COLON_TUMOR_MYELOID_DERIVED_SUPPRESSOR_CELL_UP | 73 | -5.07E-01 | -1.777559 | 0.00E+00 | 4.67E-02 |
| GSE23502_BM_VS_COLON_TUMOR_MYELOID_DERIVED_SUPPRESSOR_CELL_DN | 59 | -5.27E-01 | -1.7768465 | 1.40E-03 | 4.70E-02 |
| NOUSHMEHR_GBM_SILENCED_BY_METHYLATION | 17 | -6.63E-01 | -1.7767974 | 4.97E-03 | 4.69E-02 |
| MARTORIATI_MDM4_TARGETS_NEUROEPITHELIUM_UP | 80 | -4.98E-01 | -1.776744 | 1.41E-03 | 4.69E-02 |
| GSE26343_UNSTIM_VS_LPS_STIM_NFAT5_KO_MACROPHAGE_DN | 90 | -4.88E-01 | -1.7766463 | 0.00E+00 | 4.68E-02 |
| **RUTELLA_RESPONSE_TO_HGF_UP** | **156** | **-4.53E-01** | **-1.776463** | **0.00E+00** | **4.68E-02** |
| **BORLAK_LIVER_CANCER_EGF_UP** | **20** | **-6.48E-01** | **-1.77643** | **6.62E-03** | **4.68E-02** |
| GSE2585_CD80_HIGH_VS_LOW_AIRE_KO_MTEC_UP | 61 | -5.21E-01 | -1.776412 | 0.00E+00 | 4.67E-02 |
| GO_BLOOD_VESSEL_MORPHOGENESIS | 142 | -4.60E-01 | -1.7761611 | 0.00E+00 | 4.67E-02 |
| GSE2706_UNSTIM_VS_2H_R848_DC_DN | 87 | -4.94E-01 | -1.7761465 | 0.00E+00 | 4.67E-02 |
| RICKMAN_HEAD_AND_NECK_CANCER_A | 29 | -6.04E-01 | -1.7756692 | 8.14E-03 | 4.68E-02 |
| TOOKER_GEMCITABINE_RESISTANCE_DN | 47 | -5.46E-01 | -1.7746089 | 1.48E-03 | 4.73E-02 |
| KIM_WT1_TARGETS_UP | 89 | -4.80E-01 | -1.77454 | 0.00E+00 | 4.73E-02 |
| GO_NERVE_DEVELOPMENT | 21 | -6.63E-01 | -1.7745028 | 4.82E-03 | 4.72E-02 |
| GNF2_SERPINB5 | 14 | -7.21E-01 | -1.7742528 | 6.56E-03 | 4.72E-02 |
| AMIT_SERUM_RESPONSE_240_MCF10A | 25 | -6.22E-01 | -1.773414 | 1.60E-03 | 4.76E-02 |
| GSE41867_DAY6_EFFECTOR_VS_DAY30_EXHAUSTED_CD8_TCELL_LCMV_CLONE13_UP | 78 | -5.01E-01 | -1.7728027 | 1.37E-03 | 4.78E-02 |
| GSE360_T_GONDII_VS_M_TUBERCULOSIS_DC_DN | 75 | -4.96E-01 | -1.7725568 | 0.00E+00 | 4.78E-02 |
| **CHIARADONNA_NEOPLASTIC_TRANSFORMATION_KRAS_CDC25_DN** | **24** | **-6.15E-01** | **-1.7714686** | **1.53E-03** | **4.83E-02** |
| MASRI_RESISTANCE_TO_TAMOXIFEN_AND_AROMATASE_INHIBITORS_DN | 10 | -7.75E-01 | -1.771368 | 5.39E-03 | 4.83E-02 |
| GO_ANGIOGENESIS | 115 | -4.66E-01 | -1.7713015 | 0.00E+00 | 4.83E-02 |
| GSE25123_ROSIGLITAZONE_VS_IL4_AND_ROSIGLITAZONE_STIM_PPARG_KO_MACROPHAGE_DAY10_DN | 86 | -4.91E-01 | -1.7711408 | 0.00E+00 | 4.83E-02 |
| SCHOEN_NFKB_SIGNALING | 16 | -7.03E-01 | -1.7707393 | 6.51E-03 | 4.84E-02 |
| GSE36009_WT_VS_NLRP10_KO_DC_DN | 93 | -4.82E-01 | -1.7700554 | 0.00E+00 | 4.86E-02 |
| LI_PROSTATE_CANCER_EPIGENETIC | 10 | -7.78E-01 | -1.7698257 | 1.73E-03 | 4.87E-02 |
| RASHI_RESPONSE_TO_IONIZING_RADIATION_2 | 43 | -5.49E-01 | -1.7698079 | 4.46E-03 | 4.86E-02 |
| GSE19923_E2A_KO_VS_HEB_AND_E2A_KO_DP_THYMOCYTE_UP | 71 | -5.00E-01 | -1.7697179 | 1.37E-03 | 4.86E-02 |
| GSE42021_CD24HI_VS_CD24INT_TREG_THYMUS_DN | 89 | -4.93E-01 | -1.7695318 | 1.34E-03 | 4.86E-02 |
| GSE3982_EFF_MEMORY_CD4_TCELL_VS_TH2_DN | 85 | -4.90E-01 | -1.7693096 | 0.00E+00 | 4.86E-02 |
| AMIT_EGF_RESPONSE_240_HELA | 20 | -6.62E-01 | -1.7687366 | 1.55E-03 | 4.88E-02 |
| GSE30971_CTRL_VS_LPS_STIM_MACROPHAGE_WBP7_HET_2H_UP | 61 | -5.18E-01 | -1.7685853 | 0.00E+00 | 4.88E-02 |
| ZHENG_GLIOBLASTOMA_PLASTICITY_UP | 111 | -4.73E-01 | -1.7679074 | 0.00E+00 | 4.91E-02 |
| GSE1460_INTRATHYMIC_T_PROGENITOR_VS_DP_THYMOCYTE_DN | 76 | -4.96E-01 | -1.7674905 | 0.00E+00 | 4.93E-02 |
| GSE25088_ROSIGLITAZONE_VS_IL4_AND_ROSIGLITAZONE_STIM_STAT6_KO_MACROPHAGE_DAY10_DN | 89 | -4.86E-01 | -1.7674354 | 0.00E+00 | 4.92E-02 |
| RODRIGUES_DCC_TARGETS_DN | 34 | -5.79E-01 | -1.767119 | 3.09E-03 | 4.94E-02 |
| HUMMERICH_SKIN_CANCER_PROGRESSION_UP | 38 | -5.76E-01 | -1.7666966 | 4.63E-03 | 4.95E-02 |
| GARGALOVIC_RESPONSE_TO_OXIDIZED_PHOSPHOLIPIDS_GREY_DN | 20 | -6.54E-01 | -1.7665974 | 6.43E-03 | 4.95E-02 |
| GSE2128_C57BL6_VS_NOD_CD4CD8_DP_THYMOCYTE_UP | 73 | -5.00E-01 | -1.7664688 | 0.00E+00 | 4.95E-02 |
| GSE21063_CTRL_VS_ANTI_IGM_STIM_BCELL_NFATC1_KO_8H_UP | 86 | -4.83E-01 | -1.7663945 | 1.36E-03 | 4.94E-02 |
| RODRIGUES_THYROID_CARCINOMA_ANAPLASTIC_DN | 191 | -4.41E-01 | -1.7662091 | 0.00E+00 | 4.94E-02 |
| CERVERA_SDHB_TARGETS_1_UP | 40 | -5.70E-01 | -1.7661994 | 4.51E-03 | 4.93E-02 |
| TBK1.DN.48HRS_DN | 21 | -6.39E-01 | -1.7657793 | 3.23E-03 | 4.95E-02 |
| HALLMARK_INTERFERON_ALPHA_RESPONSE | 48 | -5.38E-01 | -1.7654287 | 1.45E-03 | 4.95E-02 |
| PID_IL6_7_PATHWAY | 14 | -7.01E-01 | -1.7653043 | 1.42E-02 | 4.95E-02 |
| HENDRICKS_SMARCA4_TARGETS_UP | 25 | -6.15E-01 | -1.7644764 | 6.06E-03 | 4.99E-02 |
| GSE13485_DAY3_VS_DAY21_YF17D_VACCINE_PBMC_DN | 54 | 6.71E-01 | 2.6120307 | 0.00E+00 | 0.00E+00 |
| SHEN_SMARCA2_TARGETS_DN | 115 | 5.34E-01 | 2.33835 | 0.00E+00 | 6.24E-04 |
| GSE13485_DAY1_VS_DAY7_YF17D_VACCINE_PBMC_UP | 54 | 6.14E-01 | 2.3298433 | 0.00E+00 | 4.16E-04 |
| GSE13485_DAY1_VS_DAY3_YF17D_VACCINE_PBMC_UP | 62 | 5.92E-01 | 2.3053858 | 0.00E+00 | 4.67E-04 |
| REACTOME_DEPOSITION_OF_NEW_CENPA_CONTAINING_NUCLEOSOMES_AT_THE_CENTROMERE | 25 | 7.18E-01 | 2.2932234 | 0.00E+00 | 4.98E-04 |
| KONDO_PROSTATE_CANCER_WITH_H3K27ME3 | 27 | 7.09E-01 | 2.2903006 | 0.00E+00 | 5.19E-04 |
| KEGG_OLFACTORY_TRANSDUCTION | 19 | 7.65E-01 | 2.2579937 | 0.00E+00 | 7.96E-04 |
| GO_DNA_PACKAGING_COMPLEX | 34 | 6.48E-01 | 2.2407036 | 0.00E+00 | 1.01E-03 |
| REACTOME_RNA_POL_I_PROMOTER_OPENING | 21 | 7.17E-01 | 2.1965733 | 0.00E+00 | 2.68E-03 |
| GSE18791_UNSTIM_VS_NEWCATSLE_VIRUS_DC_2H_UP | 47 | 5.95E-01 | 2.184505 | 0.00E+00 | 2.90E-03 |
| DCA_UP.V1_UP | 72 | 5.39E-01 | 2.1566927 | 0.00E+00 | 5.27E-03 |
| GSE13485_DAY7_VS_DAY21_YF17D_VACCINE_PBMC_DN | 61 | 5.47E-01 | 2.1527326 | 0.00E+00 | 5.09E-03 |
| REACTOME_MEIOTIC_RECOMBINATION | 29 | 6.30E-01 | 2.1399395 | 0.00E+00 | 6.07E-03 |
| GSE13485_CTRL_VS_DAY1_YF17D_VACCINE_PBMC_DN | 51 | 5.64E-01 | 2.1287868 | 0.00E+00 | 7.04E-03 |
| GGAMTNNNNNTCCY_UNKNOWN | 26 | 6.52E-01 | 2.1126797 | 0.00E+00 | 8.38E-03 |
| GO_NUCLEAR_NUCLEOSOME | 13 | 7.61E-01 | 2.104548 | 0.00E+00 | 9.13E-03 |
| MIKKELSEN_MEF_HCP_WITH_H3K27ME3 | 177 | 4.53E-01 | 2.0972004 | 0.00E+00 | 9.57E-03 |
| GO_DEFENSE_RESPONSE_TO_BACTERIUM | 58 | 5.45E-01 | 2.0925395 | 0.00E+00 | 1.00E-02 |
| KEGG_SYSTEMIC_LUPUS_ERYTHEMATOSUS | 42 | 5.57E-01 | 2.0535767 | 0.00E+00 | 1.79E-02 |
| KEGG_PRIMARY_IMMUNODEFICIENCY | 14 | 7.28E-01 | 2.0431902 | 0.00E+00 | 2.00E-02 |
| GO_DRUG_METABOLIC_PROCESS | 10 | 7.98E-01 | 2.0052402 | 0.00E+00 | 3.31E-02 |
| KEGG_NATURAL_KILLER_CELL_MEDIATED_CYTOTOXICITY | 38 | 5.69E-01 | 2.0040126 | 0.00E+00 | 3.20E-02 |
| GO_SENSORY_PERCEPTION_OF_CHEMICAL_STIMULUS | 26 | 6.22E-01 | 2.0022624 | 0.00E+00 | 3.13E-02 |
| BRCA1_DN.V1_UP | 34 | 5.64E-01 | 2.0003946 | 0.00E+00 | 3.08E-02 |
| KEGG_AUTOIMMUNE_THYROID_DISEASE | 10 | 8.03E-01 | 1.9954278 | 0.00E+00 | 3.14E-02 |
| GO_DEFENSE_RESPONSE_TO_GRAM_POSITIVE_BACTERIUM | 21 | 6.42E-01 | 1.994438 | 0.00E+00 | 3.05E-02 |
| GSE32423_MEMORY_VS_NAIVE_CD8_TCELL_IL7_IL4_UP | 68 | 4.80E-01 | 1.9741545 | 0.00E+00 | 3.83E-02 |
| GO_SYNAPTIC_VESICLE_CYCLE | 27 | 5.90E-01 | 1.956188 | 0.00E+00 | 4.64E-02 |
| MARTENS_TRETINOIN_RESPONSE_UP | 286 | 4.03E-01 | 1.955481 | 0.00E+00 | 4.52E-02 |
| GO_NEURAL_RETINA_DEVELOPMENT | 14 | 6.86E-01 | 1.9486573 | 2.52E-03 | 4.72E-02 |
| GSE19923_E2A_KO_VS_HEB_AND_E2A_KO_DP_THYMOCYTE_DN | 58 | 4.99E-01 | 1.9485862 | 0.00E+00 | 4.57E-02 |
| KEGG_CALCIUM_SIGNALING_PATHWAY | 60 | 5.00E-01 | 1.9438828 | 0.00E+00 | 4.69E-02 |
| REACTOME_PACKAGING_OF_TELOMERE_ENDS | 19 | 6.58E-01 | 1.9411077 | 5.45E-03 | 4.70E-02 |
| The enrichment of the underlying molecular mechanism was assessed by GSEA based on the gene expression data in HepG2 cells transfected with scramble control or shRNAs targeting *MYH10* (GEO Accession No.: GSE109358). The GSEA was performed to determine the biological pathways documented in the Molecular Signatures Database (MSigDB, v6) that are altered after knockdown of *MYH10.* ES, enrichment score; NES, normalized enrichment score; FDR, false discovery rate. | | | | | |
|  |  |  |  |  |  |
|  |  |  |  |  |  |
|  |  |  |  |  |  |

**Supplementary Table S6: Probes, primers and shRNAs used in this study.**

| **Names** | **Sequences (5’→3’)** |
| --- | --- |
| **Primers for CNA analyses** | |
| MYH10-F | ATAAGGAAGCCAGTCTACTCTCC |
| MYH10-R | TGTTCATTACAAACAGACAAATGCA |
| SATB-F | TGGCTCAGACACACATCC |
| SATB-R | AATACAGTTGGCTATCTCTACACT |
| ANO3-F | GCAGTGTGTTCCAGATGTTAC |
| ANO3-R | TGGTGTCCTCCTTGTTCAAT |
| LTBP1-F | AGAATGAAAGAGAGGAGAGTG |
| LTBP1-R | GCTAAACAGTAAATGAAAGAACC |
| **Probes for CNVplex assays** | |
| MYH10 probe-1 | CCCGACCCGTGGGTGGAGGGACAGGAGGGCATTGGCTCTGCGCGGCCGGG |
| MYH10 probe-2 | CCAGGCCTGTTTGGAACTAGGGCCTTAGGAAGGAGGTTGGCGACTCGGCGGGT |
| MYH10 probe-3 | ATCTTAAATTCAGTTCTTCCCCGTCCTTATCTGGAGAAAGCATATGGTGT |
| POLR2A | CCAGATGGAAGTCTGGGGTTCATGTCCCAGTAGCAGGTGATTTGTATGGA |
| POP1 | TCTCTCTTTACGCCTTCTTGGTGGAGGAATTCAGTTAAGTTTCCCCAGCAG |
| RPP14 | TGGACTGAATGCTGCACAGTTCAAACAGCTGCTTATTTCGGCTGTGA |
| TBX15 | CCTTGAAGCCTGGAAGACTCTTTCAAGTCAGAGTCTGTTTGTCAGAATCTCACATTGC |
| **Primers for RT-qPCR assays** | |
| *MYH10*-F | GACTGAGGCGCTGGATCTG |
| *MYH10*-R | AAGCAATTGCCTCTTCAGCC |
| *SPRY2*-F | CTCCGTGTGGACAGTCTAGC |
| *SPRY2*-R | GAAGTGTGGTCACTCCAGCA |
| *DUSP6*-F | CGGAAATGGCGATCAGCAAG |
| *DUSP6*-R | TTACTGAAGCCACCTTCCAGG |
| *DUSP4*-F | GGTGACGATGGAGGAGCTG |
| *DUSP4*-R | TCATAGCCGCCTTTGAGC |
| *SPRED1*-F | CAGATCGGATCACGGTGAGG |
| *SPRED1*-R | GTGCATAACTATTATCGTTGTCAGA |
| *GAPDH*-F | CGGAGTCAACGGATTTGGTCGT |
| *GAPDH*-R | TCTCAGCCTTGACGGTGCCA |
| **shRNAs** | |
| sh*MYH10*-1 | GAGATTCTGTCAATGCTTA |
| sh*MYH10*-2 | CAAGTAAGCTGCAGAATGA |
| shCtrl | TTCTCCGAACGTGTCACGT |
| F, forward primer; R, reverse primer; RT-qPCR, real-time quantitative polymerase chain reaction. | |
